# Phenological Shifts Since 1830 in 29 Native Plant Species of California and Their Responses to Historical Climate Change

**DOI:** 10.3390/plants14060843

**Published:** 2025-03-07

**Authors:** Andros Solakis-Tena, Noelia Hidalgo-Triana, Ryan Boynton, James H. Thorne

**Affiliations:** 1 Department of Botany and Plant Physiology (Botany Area), Faculty of Science, University of Málaga, 29010 Málaga, Spain; andros@uma.es; 2 Department of Environmental Science and Policy, University of California, Davis, CA 95616, USA; rmboynton@ucdavis.edu (R.B.); jhthorne@ucdavis.edu (J.H.T.)

**Keywords:** advance, day of year, flowering, fruiting, global warming, growth, Mediterranean, preserved specimens, retrospective phenology

## Abstract

Climate change is affecting Mediterranean climate regions, such as California. Retrospective phenological studies are a useful tool to track biological response to these impacts through the use of herbarium-preserved specimens. We used data from more than 12,000 herbarium specimens of 29 dominant native plant species that are characteristic of 12 broadly distributed vegetation types to investigate phenological patterns in response to climate change. We analyzed the trends of four phenophases: preflowering (FBF), flowering (F), fruiting (FS) and growth (DVG), over time (from 1830 to 2023) and through changes in climate variables (from 1896 to 2023). We also examined these trends within California’s 10 ecoregions. Among the four phenophases, the strongest response was found in the timing of flowering, which showed an advance in 28 species. Furthermore, 21 species showed sequencing in the advance of two or more phenophases. We highlight the advances found over temperature variables: 10 in FBF, 28 in F, 17 in FS and 18 in DVG. Diverse and less-consistent results were found for water-related variables with 15 species advancing and 11 delaying various phenophases in response to decreasing precipitation and increasing evapotranspiration. Jepson ecoregions displayed a more pronounced advance in F related to time and mean annual temperature in the three of the southern regions compared to the northern ones. This study underscores the role of temperature in driving phenological change, demonstrating how rising temperatures have predominantly advanced phenophase timing. These findings highlight potential threats, including risks of climatic, ecological, and biological imbalances.

## 1. Introduction

California encompasses remarkable biodiversity in an intricate landscape shaped by varied environmental conditions. These conditions have driven the development of high levels of endemism, making California the most floristically diverse state in temperate North America [1]. The state’s climate is characterized by a Mediterranean climate according to Köppen–Geiger classification [2], with winter rainfall and warm, dry summers, though with significant climatic variation. While much of California shares the arid characteristics of southwestern North America, the northwestern coastal areas support temperate rainforests. This regional climatic diversity, combined with complex topography and hydrological networks, defined by mountain ranges surrounding central and coastal valleys, generates a broad array of habitats [3,4] clearly represented by the Jepson ecoregions. California’s flora, the Jepson Manual [5], classifies the state into ten ecoregions that represent different geographic divisions based on distinctive types of dominant vegetation, topographic, geologic and climatic conditions.

Each ecoregion has a distinctive set of endemic species and communities adapted to specific climatic conditions [5,6] (see File S1 for a more detailed description). The ecoregions reveal the great vegetative diversity and climate variability that encompasses the state of California. This vegetative diversity is organized in a mid-level class of the National Vegetation Classification System (NVCS; [7]), called macrogroups. They are defined as an array of diagnostic species and growth forms, characterized by variations in composition, biogeography, geology, substrate, hydrology, disturbance regimes and mesoclimate. Essentially, a macrogroup identifies the floristic, growth forms and ecological characteristics that differentiate vegetation within a division [8,9]. In California, there are 31 terrestrial vegetation macrogroups [10], which serve as units for terrestrial conservation strategies developed as part of the State’s Wildlife Action Plan [11].

Global warming has raised the average global surface temperature by 1.1 °C [12], with pronounced impacts on climate, particularly in Mediterranean regions [13,14]. Furthermore, projections of future climate scenarios suggest that these impacts are likely to intensify [3,15]. California has experienced changes to its climate over the last century, with warming of surface temperatures by approximately 1.4 °C since 1895 and change its precipitation regime with increasing droughts, torrential rains, and decreasing snowfall [16,17,18]. Moreover, California’s climate has undergone changes in recent decades, with a reduction in the extent of temperate, oceanic and cold-type climates, giving way to more arid and desert-like conditions [19,20].

Phenology, as a bioindicator of climate change, has been instrumental in detecting ecological responses to changing environmental conditions, as shown in foundational studies [21,22,23]. Consequently, retrospective phenology studies have become essential for predicting species responses to climate change and monitoring these shifts over time [24,25]. Plant phenology is closely linked to climatic variables, and climatic changes over the past century correlate with significant shifts in the timing of yearly plant phenophases. For example, a review published by Bertin [26] compiled several studies from Europe, North America, Australasia, and other regions worldwide. They found that spring phenophases (mainly leafing and flowering) have advanced by approximately 4–5 days per °C, and a study by Peñuelas et al. [27] revealed that, in representative Mediterranean plant species, leaves unfolded 16 days earlier in the 2000s than in the 1950s on average, leaves fell 13 days later, flowering occurred 6 days earlier, and fruiting occurred 9 days earlier.

Phenological timing is critical for species, as it determines the optimal periods for the development of reproductive (e.g., flower buds, flowers, pollen, fruits) and vegetative structures essential for survival [28,29]. Shifts in phenology can also disrupt ecological interactions, such as those between leafing periods and herbivory, flowering times and pollinators, or fruiting periods and frugivorous species [30]. Furthermore, the advancement or compression of life cycles under climate change may reduce reproductive success, with significant implications for the long-term persistence and evolutionary potential of populations [31]. Vegetative and reproductive phenophases are often interlinked and interdependent; for example, preflowering buds cannot develop without preceding plant growth in some species [32,33]. The relation of phenophases with temperature can be seen in Mediterranean regions, where rising spring temperatures initiate the development of reproductive and vegetative structures, provided that chilling and forcing requirements have been met [34,35,36]. Additionally, the timing of phenological events can be influenced by annual precipitation patterns, with shifts occurring in response to drought and rainy periods throughout the year [37,38].

Since climate change has affected many plant species, herbarium specimens have been used to track its effects on plant phenology [24,39,40,41]. However, many of these studies primarily focus on a single phenophase (typically flowering) and often examine only annual or spring climate variables. Additionally, most research focuses on woody species, leaving herbaceous species understudied [42]. Regarding conservation, changes in the floristic composition of the communities, based mainly on dominant species, can also lead to a loss of the plant communities [43,44], which highlights the importance of a more comprehensive phenological analysis. We aimed to address these gaps by using herbarium specimens to track phenological changes in four distinct phenophases across a diverse range of woody and herbaceous annual species: the presence of flowering buds, opening of flowers, the onset of fruiting, and the presence of growth activity.

The observed climate changes may have left measurable impacts on the phenology of Mediterranean plant species across California. Therefore, the objective of this study was to investigate phenological trends over the past century in dominant plant species representing California’s primary vegetation macrogroups. Additionally, as a novel approach, we analyzed phenological trends within the Jepson ecoregions [5]. The selected species are dominant and native to 12 of California’s vegetation macrogroups.

This research addresses the following questions:

1. Have the selected plant species changed their day of year (DOY) in the preflowering, flowering, fruiting and growth phenophases due to the effects of climate change in California?
2. Has the flowering DOY changed across the different Jepson ecoregions due to the effects of climate change based on the selected species?

## 2. Materials and Methods

### 2.1. Study Area

**Figure 1:**
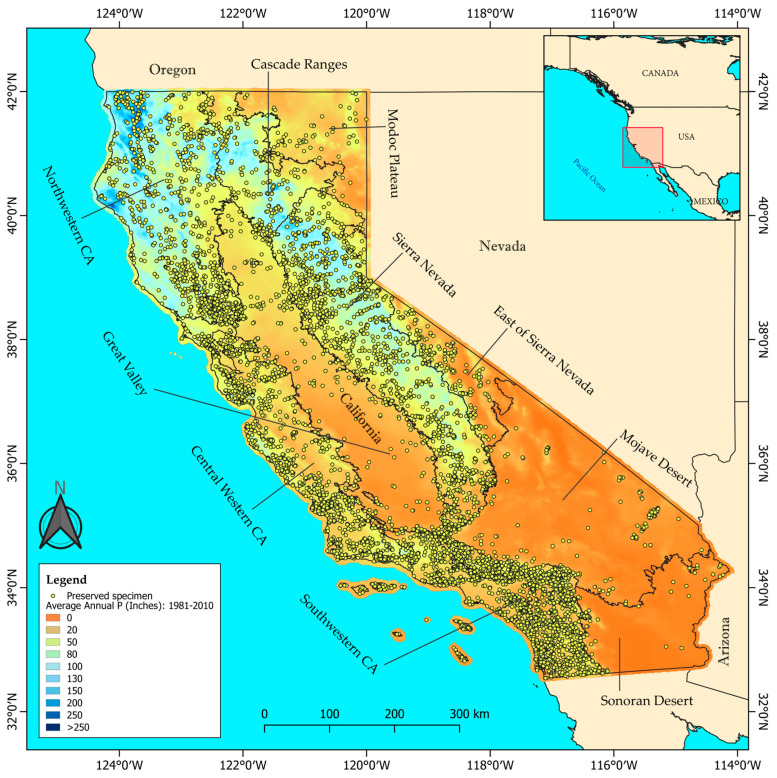
Study area, distribution of the sampled preserved specimens (yellow dots) and division of the Jepson ecoregions. The precipitation across California is based on the average annual precipitation levels in inches for California from 1981 to 2010 (California Department of Fish and Wildlife [47]).

The study spans all of California covering an area of 423,970 km^2^. We examined species phenological trends in the 10 Jepson ecoregions [5], which represent different plant floras (Figure 1; File S1), from north to south: the Cascade Ranges, the Modoc Plateau, northwestern CA, Sierra Nevada, Great Valley, the east of Sierra Nevada, central western CA, Mojave Desert, Sonoran Desert and southwestern CA. California has a water year running from October to September, with varied precipitation across the state: northern California experiencing wetter, more temperate conditions, while southern regions have drier, more desert-like conditions (Figure 1). The state has a wide variety of climate types: a hot-summer and a warm-summer Mediterranean climates in most of the state, mainly northwestern and central western CA, Sacramento Valley (Great Valley), Modoc Plateau and the Cascade Ranges ecoregions; a cold and a hot semi-arid climates, mainly in the San Joaquin Valley (Great Valley) ecoregion; a cold and a hot desert climates in the Mojave and Sonoran Desert ecoregions; a warm-summer Mediterranean continental and a dry-summer subarctic climate in the east of Sierra Nevada and small areas of the northern Modoc Plateau; and also alpine conditions in Mt. Shasta (Cascade Ranges ecoregion) [45].

### 2.2. Target Species and Phenological Data

We selected 30 species from 13 families and six growth forms [46] (File S1, Table S1). These species were dominant members of 12 broadly distributed macrogroups. The macrogroup vegetation classes were obtained from the NVCS [7] and the climate change vulnerability assessment of California’s terrestrial vegetation report [10]. We studied four phenological phases (phenophases) of the selected species to search for trends of changes through time and climate change. Each phenophase was visually determined from the herbarium specimens, observing the presence of flowering buds (i.e., preflowering, also known as flower bud formation = FBF), open flowers (blooming or flowering = F) and the onset of fruiting (fruiting or fruit setting = FS) for the three reproductive phenophases analyzed here, and the presence of growth activity (growing or dolichoblast vegetative growth = DVG) as a vegetative phenophase. Phenology can be studied in a binary way, meaning that a specimen is either in a specific phenophase or not. In this study, specimens with 50% or more of flower buds in anthesis at the time of collection were considered to be in the flowering phase, while those with less than 50% in anthesis were in the preflowering bud phase [40,47].

Unlike other studies that distinguish between initial, peak, or final flowering phases [48,49,50], we did not separate flowering into these sub-phases but instead considered them all as part of the full flowering phase (F). The development of flower buds occurs before flowering and is referred to as preflowering (FBF). Importantly, this does not imply the absence of buds in anthesis but rather indicates that the plant was closer to the preflowering stage than to the full flowering phase [47,51,52]. The third phenophase studied was fruiting (FS), which occurs after flowering. Fruiting sometimes follows a latency period before active fruit development or occurs after ovary development post-anthesis and prior to fertilization. If more than 50% of flowers were in anthesis but fruit development had already begun, the plant was classified as being in the fruit setting phase (FS), not in the flowering phase. Although some flowers remained open during the fruiting stage, signs of senescence, discoloration, stamen drop or ovary enlargement were used to confirm the transition to fruiting [53]. Fruiting was recorded at its onset, during its growth and at maturation, but not when seed dispersal had begun. In addition to these three phenophases, specific vegetative structures were also considered. The vegetative phase was recorded when growth activity was observed in dolichoblast branches (DVG; branches with large internodes), which habitually serves to expand the crown and explore favorable nearby microenvironments. Dolichoblasts mainly grow in spring and typically end their growth with an inflorescence [33]. Additionally, we examined whether phenophases exhibited consistent change patterns, meaning that if one phenophase advanced or was delayed, the others followed the same trend. From now on we will refer to this effect as “sequencing” [54,55].

We used specimens from the herbariums of the UC Davis Center for Plant Diversity and the Jepson and University Herbaria at UC Berkeley to visually examine 10,350 herbarium sheets, collected between 1830 and 2023 for 30 different species. From these preserved specimens, 5742 were in at least one phenophase of interest. We enriched our database by taking advantage of the Consortium of California Herbaria [56] and the specimens examined in Ramirez-Parada et al. [41], which both contributed to the database with 10,548 registers. We cleaned the database by removing duplicate records and specimens which were clearly indicating a second flowering and removed the specimens indicated as “in fruit”, since they do not differentiate between a mature fruit, ready to disperse or the starting fruiting phase, the latter being a focus for this study. Our final database was made up of 12,010 registers that were in at least one phenophase of interest. We also developed phenological calendars, which are graphical representations detailing the timing of phenophases occurring in each plant species throughout the year. To analyze the DOY changes through time, we had data from 1830 to 2023 (193 years), but when analyzing DOY changes over climate variables, we had to remove the registers with no climatic data, resulting in a database with 11,366 registers and data from 1896 to 2023 (127 years). Each register was reviewed, and its location, coordinates and day of year (DOY) of a phenophase from the date of sampling were recorded from the preserved specimens’ label.

### 2.3. Climate Data Treatment

We used the Basin Characterization Model (BCM) [57] to extract monthly and annual climatic data from 1896 to 2023 for all of the specimen locations and for the spatial mean through time within the 10 Jepson ecoregions. This allowed us to examine changes in climatic conditions between historical and current time periods. The BCM is a process-based model that balances the hydrologic cycle for each grid cell in the area modeled, where historical climate is derived from the downscaled version (270 m grids) of the empirically based PRISM monthly precipitation and air temperature database [58,59].

The BCM extractions provided us with temperature and precipitation-related variables, calculated on monthly and annual time steps (File S1, Table S2). In addition, we obtained the mean spring and autumn temperatures, by calculating the average of March, April and May monthly temperatures of the same year for spring T, and the average of September, October and November of the previous year for autumn T. The total spring and autumn precipitation were also calculated by the sum of March, April and May monthly P for spring P, and the sum of September, October and November of the previous year for autumn P.

Subsequently, we reduced the number of variables from 42 to 8 when they were highly correlated (>0.75) using the corrplot function in R software (v4.4.0) [60]. This resulted in 8 variables being selected for the analysis; three yearly climatic variables: the mean annual temperature (annual T), the sum of annual precipitation (annual P), and actual evapotranspiration (AET); three seasonal climatic variables: spring temperature and precipitation (spring T and spring P, respectively) and autumn precipitation (autumn P of the previous year); and two monthly variables: mean monthly temperature (monthly T) and total monthly precipitation (monthly P) (File S1, Figures S1 and S2). The monthly T and P were considered in the analysis because they previously proved to have a high explanatory power over the trends of the DOY [61].

### 2.4. Statistical Analysis

First, we constructed time and climatic models for each selected plant species (question 1), where the DOY was compared to its corresponding decimal year and to the climatic variables. We set a minimum of 10 independent specimens records (i.e., records in different locations and dates) in the phenophase of study following Gallagher et al. [62] and Calinger et al. [51], in order to be able to run the models. The sample size for phenophase and species ranged from 1 (DVG in *Sequoia sempervirens* (D.Don) Endl.) to 1206 (F in *Salvia mellifera* Greene) (File S1, Table S3). Regarding ecoregions, the sample size ranged from 96 (Modoc Plateau) to 5609 (southwestern CA) (File S1, Table S4). To avoid problems related with second flowering times and outliers we applied the data correction method of Menzel et al. [63] and Picornell et al. [64], where DOYs out of the median ± 3.5 × MAD (median absolute deviation) were discarded in the models.

We used linear regression models (LM) to analyze the DOY trends, but when model assumptions were not met, we used generalized linear models (GLM [65]) or generalized additive models (GAM [66]). We applied the Shapiro–Wilk (*n* < 50) or the Kolmogorov–Smirnoff (*n* ≥ 50) tests for normality. The implemented GLM were of Gaussian family and logarithmic link function. Parallelly, a series of GAM were employed given their increased flexibility in capturing nonlinear relationships. The GAM used were Gaussian, with an identity link function, and included five bases (k = 5) smoothed with the thin plate regression spline (bs = “tp”) for the time models, and the shrinkage version (bs = “ts”) for the climatic models [67]. The restricted maximum likelihood method (REML) was used to fit the GAM.

For the models through time, we ran the DOY over the years, while for the climatic models, a set of different models were evaluated in a stepwise manner for each case (i.e., phenophase and species). The set of models varied in the number of independent variables introduced, and the higher the parsimony the better information contributed to the model [68]. We first tested individual variables, then progressively added more temperature-related variables, including monthly temperatures and spring averages. Subsequently, precipitation variables were introduced, including monthly and spring precipitation, as well as autumn precipitation from the previous year. We also constructed models containing only temperature variables or only precipitation and evapotranspiration variables to explore their isolated effects. Finally, from this comprehensive set of models, the one with the highest parsimony, as indicated by the Akaike Information Criterion (AIC) [68], was selected. The variance inflation factor (VIF) for the climatic variables were also calculated, and the AIC was also compared between the GLM and GAM to select the best model between them.

Secondly, we conducted models for each of the 10 Jepson ecoregions (question 2). The DOY was modeled against time and climatic variables. Considering the large extents of the ecoregions, the low data of FBF and DVG for these extensions and the high FS variance when mixing species, we decided to run the models for just the F phenophase. We focused the climatic variables analysis on long-term indicators, specifically annual temperature, annual precipitation and time. This approach allowed us to provide a general overview of the main phenological changes by examining the combined phenological patterns observed across species within each ecoregion.

## 3. Results

### 3.1. Phenological Trends by Species

Twenty nine species had enough samples to model against temporal and climatic variables. The species displayed different number of samples depending on the phenological phase being analyzed: 17 species in preflowering (FBF), 29 species in flowering (F), 26 species in fruiting (FS) and 21 species in growth (DVG) had sufficient samples (File S1, Table S3). The phenological calendars of each studied species by phenophase are available in File S1, Figure S3. The number of species showing changes (i.e., advances or delays) in DOY, in relation to at least one variable, varied by phenophase: 14 species exhibited changes in FBF, while no changes were observed in *Juniperus californica* Carrière, *Quercus chrysolepis* Liebm. and *Quercus durata* Jeps.; 29 species showed changes in F, with all species exhibiting significant shifts; 19 species exhibited changes in FS, but no changes were detected in *Arbutus menziesii* Pursh, *Heteromeles arbutifolia* (Lindl.) M.Roem., *Pinus attenuata* Lemmon, *Pinus ponderosa* Douglas ex C.Lawson, *Quercus agrifolia* Née, *Q. durata*, *Quercus lobata* Née and *Quercus vaccinifolia* Hittell; 20 species exhibited changes in DVG, except for *Arctostaphylos patula* Greene, which showed no significant changes. Most species were consistent within changes by sequencing their phenophases. To examine the outcomes of all the models conducted in detail for each of the species, as well as the results and graphs of the model validation, see File S2.

#### 3.1.1. Phenological Trends by Species over Years

**Figure 2:**
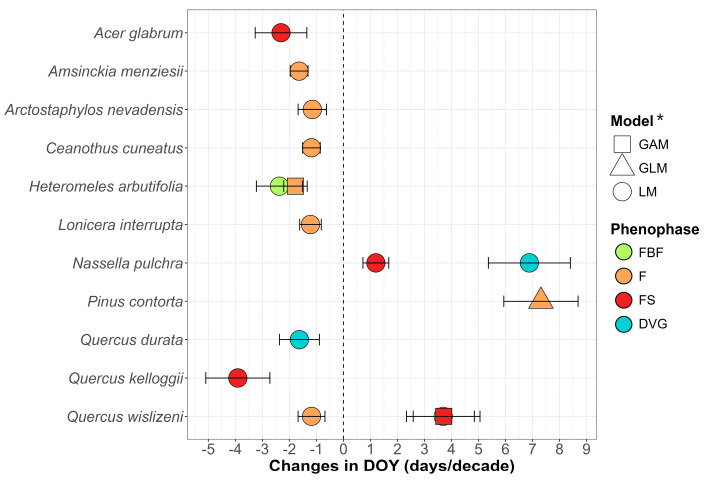
Trends of the DOY against years by phenophase (FBF: preflowering; F: flowering; FS: fruiting; DVG: growth). Only species with significant trends are shown, all trends are in File S2.1. The results show the slopes of the DOY over the years, which can be expressed in days in advance (to the left of 0) or delay (to the right of 0) per decade. It should be noted that FBF in *Q. wislizeni* delayed despite being overlapped by FS in the figure. * To maintain plot uniformity and facilitate comparison, all slopes shown are from LM results. However, for species marked with triangles (GLM) and squares (GAM), the final interpretation is based on these models, readers should refer to the Supplementary Material File S2.2 for the accurate model results.

Over time, 11 species exhibited phenological shifts in one or more of their phenophases, with 9 showing advances: one (*H. arbutifolia*) in FBF, six in F, two in FS (*Acer glabrum* Torr. and *Quercus kelloggii* Newb.) and one (*Q. durata*) in DVG (Figure 2). Two species were delayed significantly: *Quercus wislizeni* A.DC. in FBF, *Pinus contorta* Douglas ex Loudon in F and *Nassella pulchra* (Hitchc.) Barkworth in FS and DVG. The average advance in reproductive phenophases (FBF, F and FS) was −1.86 days/decade, and *Q. durata* alone showed an advance of −1.63 days/decade in DVG, while the average delay was 3.98 days/decade in reproductive phenophases and *N. pulchra* alone showed 6.88 days/decade in the DVG. To ensure visual comparability of trends across all species, slopes from LM were used in the figure, even for species where GLM or GAM provided a better model fit. Symbols (triangles and squares) indicate these cases and full model details are provided in File S2.1.

Two species showed sequencing of phenophases: *H. arbutifolia*, advancing both FBF and F, and *N. pulchra*, delaying FS and DVG. *Q. wislizeni* showed no sequencing in F compared to FBF and FS, since the first showed advance while the others showed delays (Figure 2). The majority of the models were LM type, but in the cases of F in *H. arbutifolia*, F in *P. contorta* and FS in *Q. wislizeni*, GLM and GAM type models were implemented (File S2.1). The time models presented an R^2^ range of ≈0 (low explained variance)—0.63 (high explained variance); for more information, see File S2.1.

#### 3.1.2. Phenological Trends by Species Through Climate Variables

The climatic models show more significant phenological change than when we consider only the passage of time. The R^2^ in these models were higher than in the time models, reaching values of 0.93 (File S2.2). All 29 analyzed species showed significant trends with respect to long-term climatic variables: 29 species against temperature variables and 18 species against the water-related variables of precipitation (annual P, spring P and autumn P of the previous year) and actual evapotranspiration (AET). See File S1, Figure S4 and Tables S5 and S6 to check the main climatic variables trends across California since 1896.

**Figure 3:**
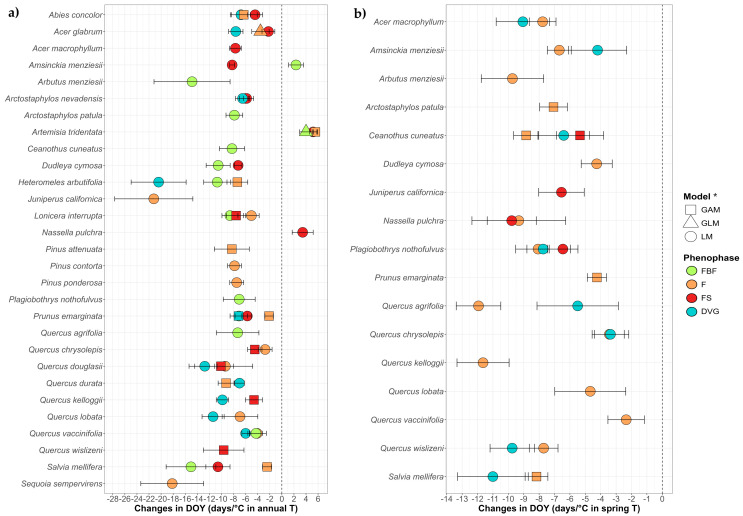
Trends of the DOY in the climatic models for 29 species and four phenophases (FBF: preflowering; F: flowering; FS: fruiting; DVG: growth)over the increase in mean annual temperature (**a**) and spring temperature (**b**). Only species with significant trends are shown, all trends are in File S2.2. It should be noted that in (**a**), despite being overlapped by other phenophases, *A. nevadensis* and *Q. vaccinifolia* show advances in F and *P. emarginata* in FBF as well, while in (**b**), F advanced in *Q. chrysolepis*. * To maintain plot uniformity and facilitate comparison, all slopes shown are from LM results. However, for species marked with triangles (GLM) and squares (GAM), the final interpretation is based on these models, and readers should refer to the Supplementary Material File S2.2 for the accurate model results.

The main tendency found against annual or spring T was the advance of the phenophases (Figure 3), with 79 models showing significant advances against five models that show delays. The average advance for the reproductive phenophases (FBF, F and FS) against the increase in temperature-related variables was −7.76 days/°C in annual T and −7.2 days/°C in spring T, while the average advance regarding DVG was −9.55 days/°C in annual T and −7.12 days/°C in spring T. On the other hand, the average delay was 4.14 days/°C in annual T regarding the reproductive phenophases (F and FS), with no delays against spring T and no delays in DVG in both variables.

Regarding the mean annual T (Figure 3a), advances were found for 11 species in FBF, 17 species in F, 13 in FS and 10 in DVG. Almost all advances in one phenophase presented sequencing with others, highlighting *Abies concolor* (Gordon & Glend.) Lindl. ex Hildebr., *A. glabrum*, *Arctostaphylos nevadensis* A.Gray, *H. arbutifolia*, *Lonicera interrupta* Benth., *Prunus emarginata* (Douglas ex Hook.) Eaton, *Quercus douglasii* Hook. & Arn., *Q. durata*, *Q. vaccinifolia* and *S. mellifera*, which were species that showed sequencing in three or more of their phenophases. The delays were found in: *Amsinckia menziesii* (Lehm.) A.Nelson & J.F.Macbr. in FBF, *Artemisia tridentata* Nutt. for all the studied phenophases, and *N. pulchra* in FS. In these cases, *A. tridentata* presented sequencing in the delays of all the reproductive phenophases, but *Amsinckia menziesii* showed no sequencing since F advanced while FBF delayed.

Regarding the increase in spring T (Figure 3b), all findings were of advance, 16 species in F, four in FS and eight in DVG. Furthermore, 9 species showed sequenced advances, highlighting *Ceanothus cuneatus* (Hook.) Nutt. and *Plagiobothrys nothofulvus* (A.Gray) A.Gray that showed advances in three of their phenophases.

**Figure 4:**
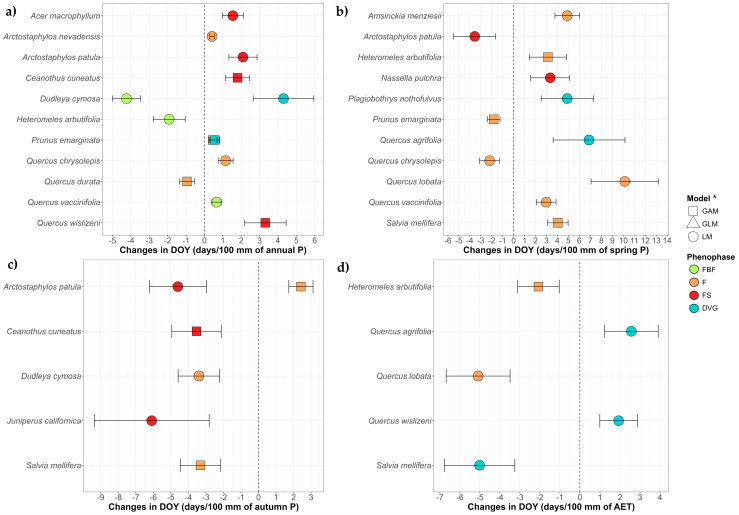
Trends of the DOY in the climatic models for every species and phenophase (FBF: preflowering; F: flowering; FS: fruiting; DVG: growth) over the increase in annual precipitation (**a**), spring precipitation (**b**), autumn precipitation of the previous year (**c**) and actual evapotranspiration (**d**). Only species with significant trends are shown, all trends are in File S2.2. * To maintain plot uniformity and facilitate comparison, all slopes shown are from LM results. However, for species marked with triangles (GLM) and squares (GAM), the final interpretation is based on these models, readers should refer to the Supplementary Material File S2.2 for the accurate model results.

As in Figure 2, Figure 3 and Figure 4 show slopes from LM, even for species where GLM or GAM were implemented. Symbols indicate these cases, and full model details are provided in File S2.2.

Regarding water-related variables (Figure 4), 16 species show significant advances, and 11 species show delays in one or more phenophases with the loss of water, i.e., decrease in precipitation or increase in evapotranspiration. For the reproductive phenophases (FBF, F and FS), the average advance was −3.19 days/100 mm decreased, and the average delay was 3.24 days/100 mm decreased in relation to precipitation variables (annual P, spring P and autumn P of the previous year). With respect to actual evapotranspiration (AET), the average advance was −3.58 days/100 mm increased, with no delays. For DVG, the average advance was −2.43 days/100 mm decreased, and there were no delays in relation to precipitation variables, while with respect to AET only *S. mellifera* showed advance (−5.01 days/100 mm increased), and *Q. agrifolia* and *Q. wislizeni* showed delays (2.24 days/100 mm increased on average).

Many species showed change with an increase in annual P (Figure 4a), where two species in FBF (*Dudleya cymosa* (Lem.) Britton & Rose and *H. arbutifolia*) and one species in F (*Q. durata*) were advanced per 100 mm increase, while one species in FBF (*Q. vaccinifolia*), three species in F and four in FS were delayed per 100 mm increase. Regarding an increase in spring P (Figure 4b), the advances were found in the F of two species (*P. emarginata* and *Q. chrysolepis*) and the FS of one (*A. patula*), while the delays were found in the F of four species, the FS of one (*N. pulchra*) and the DVG of two species (*P. nothofulvus* and *Q. agrifolia*) when the variable increased. An increase in autumn P of the previous year (Figure 4c) was related to advances in the F of two species (*D. cymosa* and *S. mellifera*), the advance in FS of three species and the delay of the F in one species (*A. patula*). With the increase in AET, two species showed advances in F (*H. arbutifolia* and *Q. lobata*) and one in DVG (*S. mellifera*), while two showed delays in DVG (*Q. agrifolia* and *Q. wislizeni*) (Figure 4d).

Only two species showed changes in more than one phenophase: *P. emarginata* delaying both F and DVG with the increase in the annual P with a coherent sequencing, while *A. patula* showed contrary results with the increase in autumn P of the previous year, advancing FS but delaying F.

### 3.2. Phenological Trends by Jepson Ecoregion

**Figure 5:**
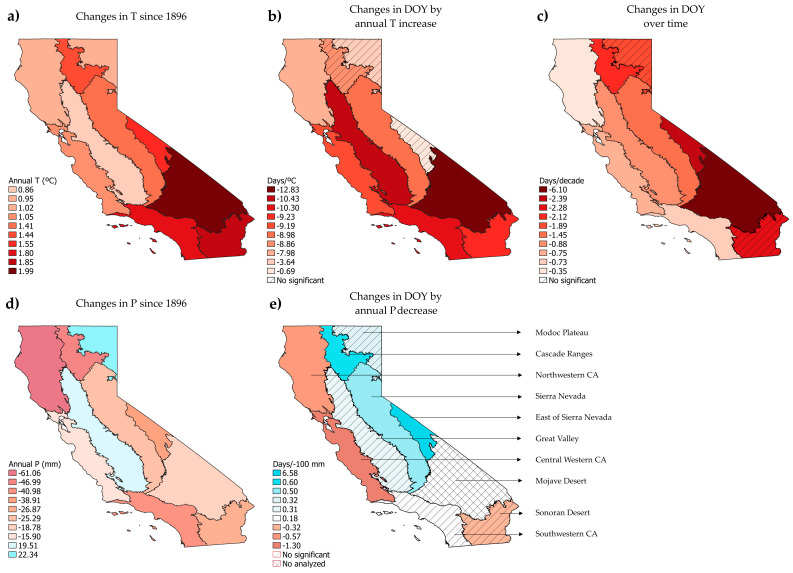
Left of the figure: Changes in the climatic variables of annual T (**a**) and P (**d**) from 1986 until 2023 by Jepson ecoregion. All changes in T were significant, while changes in P were not. Center and right columns of the figure: relation of the flowering DOY against annual T (**b**), time (**c**) and annual P (**e**), based on the preserved specimens of the target species present in each Jepson ecoregion. Red and brown colors indicate an increase in annual T (**a**) or a decrease in annual P since 1896 (**d**), while blue colors indicate an increase in annual P (**d**). Advances in the DOY over time (**c**) and annual T (**b**) are indicated in red colors. Changes in the DOY with the decrease in annual P (**e**) are represented in red colors when advancing and in blue colors when delaying.

The 10 Jepson ecoregions show significant changes in the DOY of flowering in front of long-term variables (Figure 5). All the trends in F over time and over the increase in annual T were of advance in all ecoregions. The average F advance over time was −1.9 days/decade by Jepson ecoregion, and −8.21 days/°C per increased mean annual T. Regarding annual P, the trends were heterogeneous with many non-significant changes. The average advance with decreasing annual P was −0.73 days/−100 mm, while the average delay was 1.41 days/−100 mm. To examine the outcomes of all the models conducted in detail for each Jepson ecoregion, as well as the results and graphs of the model validation, refer to File S3.

In general, the time models displayed a low proportion of explained variance suggesting that the time variable (years) alone was not a strong enough predictor. This was different in the models with climatic variables, where the R^2^ increased significantly (0.11–0.75). The results in the trends of changes in the DOY by annual T and over time were closely related to the increase in annual T in each ecoregion. The trends in annual T showed a greater increase in the southern ecoregions compared to the northern ones (Figure 5). Both changes in DOY over annual T and time matched with the changes found in T since 1896. This reflects a more pronounced advance in the DOY, in relation to annual T and to years, as we move latitudinally downward across the ecoregions. The highest trends of warming in annual T since 1896 to the present were found in the Mojave Desert (1.99 °C), Sonoran Desert (1.85 °C), southwestern CA (1.8 °C), the east of Sierra Nevada (1.55 °C), Cascade Ranges (1.44 °C) and Sierra Nevada (1.41 °C) ecoregions. In parallel, the highest phenological advance by increased °C was found in the Mojave Desert (−12.83 days/°C), followed by the Great Valley (−10.43 days/°C), southwestern CA (−10.3 days/°C) and Sonoran Desert (−9.19 days/°C), while Sierra Nevada performed a lower change relative to the other of ecoregions (−8.98 days/decade). Regarding phenology advances by decade, the highest were found in the Mojave Desert (−6.1 days/decade), the east of Sierra Nevada (−2.39 days/decade), Sonoran Desert (−2.28 days/decade) and Cascade Ranges (−2.12 days/decade).

Regarding the trends of change in the DOY by annual P since 1896, no clear trends were related to the changes observed in P by ecoregion (Figure 5). The highest decreases in P from 1896 to the present were found in northwestern CA (−61.06 mm), Cascade Ranges (−46.99 mm), southwestern CA (−40.98 mm) and the east of Sierra Nevada (−38.91 mm), while some increases were found in the Great Valley and Modoc Plateau ecoregions.

However, no trend for annual P was significant. Matching with the decrease in P were the advances found in northwestern CA, central western CA and the Sonoran Desert (not significant). However, the decrease in P in the Cascade Ranges, the east of Sierra Nevada and Sierra Nevada ecoregions, caused the opposite effect on the DOY, a delay in these three ecoregions with the decrease in annual P. The Great Valley, Modoc Plateau and southwestern CA showed a low and non-significant trend of change, and no model performed with this variable for the Mojave Desert ecoregion.

The time models for Great Valley, Cascade Ranges and Sonoran Desert were LM type, for East of Sierra Nevada, Modoc Plateau and Mojave Desert were GLM type, and for central western CA, northwestern CA, Sierra Nevada and southwestern CA were GAM type. The selected climatic models for Great Valley, central western CA, Modoc Plateau and Sonoran Desert were LM type, while for Cascade Ranges, the east of Sierra Nevada, Mojave Desert, northwestern CA, Sierra Nevada and southwestern CA were GAMs. Refer to File S3.1 to examine in detail the outcomes of the time models and File S3.2 for the climatic models.

## 4. Discussion

The results of this study shed light on the phenological trends for the analyzed plants in response to climate change, revealing that the primary response is an advancement of the phenophases, mainly in flowering (F) followed by fruiting (FS), growth (DVG) and preflowering (FBF). The findings emphasize the critical role of climate in driving phenological change across different species, phenophases and ecoregions. Temperature-related variables emerged as more influential drivers than water-related variables. While temperature trends consistently led to phenological advances, the responses to precipitation and evapotranspiration were more variable. However, the results demonstrate that all studied species are undergoing significant phenological shifts due to climate change over the past century. At the species level, flowering was the most affected phenophase, and the variable contributing most consistently to phenological changes across all species was the increase in mean annual temperature. We also highlight the delaying effect of increased spring and annual precipitation on several species, contrasting with the advancing effect observed with increased autumn precipitation of the previous year. However, long-term climate data indicate a general decline in precipitation across the state [69,70]. Taking this into account, the interpretations of the results concerning P must be considered inversely. Specifically, if the observed relationship between a phenophase and an increase in P is positive, it implies a trend toward advancement, as P has generally decreased. Consequently, the changes observed in annual and spring precipitation have contributed to a general phenological advance under drier conditions [71,72], and the opposite effect was observed with autumn precipitation. The influence of AET can be expected to mirror the effects of decreasing precipitation because evapotranspiration is limited by available soil moisture; a decrease in AET indicates loss of available water in plants. This water deficit could lead to varying phenological responses depending on the species. However, AET appeared to have a relatively minor influence on the studied species. These findings underscore the well-established dominant role of temperature in regulating plant phenology [27,73,74,75] and the comparatively smaller influence of precipitation [39,76]. Notably, in the models incorporating climatic variables, the greatest contribution was from monthly temperature, further highlighting the strength of intra-annual temperature variations in shaping phenological responses [77].

### 4.1. Phenological Response to Climate Change

Temporal analyses revealed a pronounced rate of change, with an average phenological advancement of approximately −1.8 days per decade on reproductive phenophases. This suggests that reproductive phenology is now occurring over 20 days earlier than in 1896. Additionally, we found an average advancement in reproductive phenology of −7.8 days per degree of increase in mean annual T, and an average advance in DVG of −9.6 days per degree of increase in mean annual T. Given that the state’s mean annual T has risen by approximately 1.4 °C over the past 127 years (File S1, Tables S5 and S6), it is expected that most phenological phases now occur more than 10 days earlier than at the beginning of the 20th century. This aligns with findings from numerous studies developed in other Mediterranean regions [27,78], Europe [22,63,79] and North America [80,81]. Spring T plays a particularly critical role in the development of reproductive structures and growth in plants within Mediterranean-type climates. They are among the most significant drivers triggering the flowering period and, subsequently, the fruiting period [82,83]. Our findings confirm the strong influence of spring T in advancing both DVG and later reproductive phases. However, it is noteworthy that no changes were observed in FBF. This early reproductive phenophase was instead associated with the increase in mean annual T, indicating that the temperatures influencing FBF are those preceding the spring season. It is likely that spring T has limited influence on FBF because many species initiate this phenophase between February and April (File S1, Figure S3), before spring T has fully taken effect. Since spring T in California has risen by approximately 1.1 °C, and the average phenological advancement in response to this variable is −7.2 days per degree, it is estimated that phenological events are occurring more than a week earlier now than before the 1900s.

The observed sequencing in our results for most species support the idea that warming temperatures are the primary drivers of phenological shifts. While these patterns suggest potential synchrony, this was a finding that emerged during the analysis rather than an initial objective of the study. After observing these results, we believe that further investigation into this phenomenon would be valuable. Future studies could explore whether these patterns reflect causal dependencies or are driven by shared climatic drivers. However, many studies have previously indicated how rising temperatures affect earlier phenophases, which, in turn, influence subsequent ones [74,84,85]. Moreover, the changes found in FS are crucial for plant fitness, as the timing of fruit development and ripening directly impacts the efficiency of seed dispersal, emphasizing the role of FS in ensuring successful reproduction and species perpetuation [86]. Most plant species demonstrated a consistent directional shift in their phenophases in response to the different climatic variables, with only two exceptions. In the case of *Q. wislizeni*, F advanced by approximately one day per decade, while FBF and FS were delayed by nearly four days per decade. This makes *Q. wislizeni* the only *Quercus* species in the study to exhibit delays. Phenological delays are often attributed to the failure to meet chilling requirements [87], where sufficient cold temperatures are needed to induce dormancy. However, the contrasting shifts observed between phenophases in *Q. wislizeni* seem contradictory and difficult to reconcile. This inconsistency could suggest that different phenophases may be influenced by distinct climatic drivers, physiological mechanisms or local site conditions in this species. Similar situations have been reported in other studies showing conflicting results for different phenophases [48,88]. While it is true that FBF was delayed in our results for *Q. wislizeni*, CaraDonna et al. [48] point out that “changes in phenological firsts can misrepresent overall phenological change”, suggesting that, although part of the reproductive phenology is delayed, the overall trend may still indicate advancement. Nevertheless, despite these reports, no clear explanation exists for these anomalous phenomena, particularly the lack of sequencing among advances in reproductive phenophases observed in *Q. wislizeni*. Additionally, the delay in FBF for this species is unexpected, as chilling-hour accumulation has been reported to have a limited influence on other oak phenophases, such as leafing or budburst [89,90]. Therefore, this observed effect could be more closely associated with sampling bias in the data used for this case. On the other hand, if this effect is not due to sampling bias, delays observed in FBF could result in a shortening of the reproductive phenological period due to constraints imposed by FBF on F. Specifically, early phenological phases can constrain later phenophases, something reported in Ettinger et al. [85], supporting the idea that shifts in one phenophase can have cascading effects on later phases. The interphase duration between FBF and F may limit the development time available for flowering, potentially leading to a shorter flowering period. Similarly, the delay in FS in *Q. wislizeni* over time is difficult to explain using the climatic variables analyzed in this study, as annual temperature and precipitation had the opposite effect. *Q. wislizeni* is a species with high ecological variability [91], which may also contribute to these contrasting findings.

The other case of species without sequencing in phenophases was the annual herbaceous species *Amsinckia menziesii*. This species exhibited a delay in FBF but an advancement in FS with increasing mean annual temperature. *Amsinckia menziesii* appears to be undergoing a similar process to *Q. wislizeni*. In this case, the chilling effect may play a role in regulating the onset of earlier phenophases, possibly due to its herbaceous growth form, which is known to respond flexibly to temperature cues [92,93]. In contrast, the FS in this species is advancing, with sequencing in the advance of F and DVG over spring T and time, aligning with findings by Alecrim et al. [94]. Another herbaceous species that exhibited phenological delays was *N. pulchra*. The FS in this species appears to be influenced by the increase in mean annual T and the decrease in spring P, which tended to advance the phenophase. However, spring T exerts varying effects, accelerating F and FS. It seems that the combined influence of these variables, along with the delay of DVG, contributes to the observed delay of FS over time. *N. pulchra* was the only Poaceae species analyzed in this study, and its delayed phenology aligns with previous findings for this family, where similar phenological delays have been reported in response to time [95]. This phenological shift could pose an additional threat to the species, potentially increasing the risk of being replaced by other grasses with greater plasticity that are invasive [96]. These threats also affect herbaceous species such as the annual *Amsinckia menziesii* and *P. nothofulvus*, as well as the perennial *D. cymosa*, since they are undergoing changes in many phenophases, including DVG, which is crucial for their development and success.

The shrub *A. tridentata* was the only species to delay all three reproductive phenophases with sequencing and in response to the same variable. Some studies suggest that Asteraceae species, such as *A. tridentata*, are not reliable indicators of climate change [95,97], which may explain the lack of phenological changes observed beyond those related to mean annual T. Conversely, the same studies highlight that Rosaceae species are good indicators of climate change, consistent with our results for *H. arbutifolia* and *P. emarginata*, which exhibited a highly advancement with sequencing in multiple phenophases, strongly reflecting the effects of climate change. Most of the remaining shrub and bush species generally advanced their phenology, with some exceptions related to precipitation trends. *A. nevadensis*, *A. patula*, *C. cuneatus*, *Q. durata*, *Q. vaccinifolia* and *S. mellifera* showed phenological advances in response to spring and annual T. Contrary to our findings, Parker [98] did not detect phenological changes in flowering over time in various species of *Arctostaphylos* and *Ceanothus*. Here, we observed an advance of −1.2 days per decade in the F of *A. nevadensis* and *C. cuneatus* over time, along with an average advance of −6.9 days across additional phenophases in *A. patula*, associated with each degree increase in spring and annual T. This discrepancy with Parker [98] could be attributed to differences in the *Arctostaphylos* species analyzed, as well as variations in the sample size of herbarium specimens studied. Parker [98] noted that results might change with larger sample sizes or different species from these genera. The contrasting results for F in *A. patula*, showing advancing patterns with increasing temperatures but delays with autumn P of the previous year, are likely related to the species’ ecological niche. *A. patula* occupies higher-elevation areas prone to snowpack [5], where increased autumn precipitation could lead to greater snow accumulation and, consequently, delayed flowering. Conversely, rising temperatures, particularly in spring, result in earlier snowmelt and, therefore, a potential advancement in flowering for *A. patula*. The shrub-vine *L. interrupta* showed similar advances to those reported by Cayan et al. [99], where species of *Lonicera* exhibited an average flowering advance of −3.8 days per decade, being similar with our study, where we found an advance of −1.2 days per decade and −5 days per degree increase in annual T for the flowering of *L. interrupta*. The advances found in the shrub *J. californica* with the autumn P of the previous year stand out because it was the only gymnosperm influenced by a water-related variable, this is a species with an anisohydric strategy [100], potentially conferring more direct responses to changes in P. The rest of the gymnosperms advanced only in response to annual T and exclusively in the F phenophase, except for *A. concolor* (see DVG and FS). These results further emphasize the dominant role of temperatures as phenological drivers, while precipitation appears to have a lesser influence on long-term changes. On average, F in gymnosperms advanced by −11.5 days per degree increased in annual T, a higher ratio compared to the −5.4 days in angiosperms. It is worth highlighting the contrasting results for *P. contorta*, which, unlike the general advance observed with annual T, exhibited a delay of 7.3 days per decade in F. It is likely that these differences are due to variables not accounted for in this study (e.g., soil moisture or photoperiod [101]), or due to the fact that sometimes, this species has a wetland-riparian or coastal strand ecology. Furthermore, this wide ecological range and its infraspecific diversity could contribute to distorting of the flowering patterns when analyzed collectively. In addition, it should also be mentioned that it was one of those with the smallest sample size, this being a potential reason to bias these contrary results [102]; therefore, the conclusions for this species should be taken with caution.

The advance of F in the evergreen *Arbutus menziesii* was influenced by spring T, while FBF advanced in response to annual T. This again suggests that the advancement of FBF is likely driven by temperatures outside the spring. *Acer* species advanced their phenology over time, with rising temperature variables and decreasing annual P. Notably, a significant advance was observed in FS for both species and in DVG for *Acer macrophyllum* Pursh. An earlier fruiting period could be beneficial for these species, as a delay in this phenophase might postpone or extend germination, increasing the risk for seeds or seedlings to face harsher conditions, such as hot, dry summers [103]. However, earlier growth in both species, as a plant response to the earlier onset of optimal temperatures, could pose a risk to their survival. Some populations of *A. macrophyllum* have exhibited vulnerability to rising temperatures, responding with declines in tree health and vitality, and increased mortality rates [104].

With the exception of specific cases already mentioned for *Q. wislizeni*, all *Quercus* species advanced their phenology, particularly in response to increasing temperatures and spring and annual P. Similarly to *Acer* species, the species *Q. lobata*, *Q. douglasii* and *Q. kelloggii* are deciduous trees that shed their foliage in autumn and regrow it in spring. Regarding these species and the evergreen *Q. agrifolia*, Armstrong-Herniman and Greenwood [105] identified mean accumulated precipitation, winter temperature and precipitation as the main drivers of vegetative growth and reproductive phenophases. Their findings, along with other oak studies [89,106], partially align with ours, as these four species in our study showed a mean phenological advance of −9 days per degree increase in annual T and −8.4 days per degree increase in spring T. Both *Q. agrifolia* and *Q. lobata* exhibited phenological changes in response to spring T and P but not to annual or autumn P of the previous year. Notably, Gerst et al. [107] suggested that the deep root systems of these species might shield them from the effects of precipitation variability, although they did find an acceleration in the onset of phenophases driven by temperature increases. On the other hand, Mazer et al. [37] reported an advance in flowering but a delay in budburst for *Q. lobata* during periods of heavy rainfall in December and March, as well as delays in vegetative bud break in response to increased March T. Although we did not measure budburst directly, this phenophase precedes and synchronizes the onset of DVG [54]. Therefore, these findings differ from ours, where temperature increases consistently caused advances in both DVG and F, while increased spring P delayed F. Overall, five *Quercus* species showed phenological changes influenced by precipitation or evapotranspiration (water-related variables), consistent with other studies that highlighted the role of precipitation in oak budburst, flowering and fruiting phenophases [37,105,107]. *Q. vaccinifolia* advanced both FBF and F in response to decreasing P, likely translated as less snowpack and earlier melt out due to spring conditions influencing earlier flowerings [108], while *Q. chrysolepis* exhibited contrasting results between annual and spring P. Unlike temperature, which showed a clear pattern of advance across oaks, precipitation effects varied by species, variable, and phenophase. Consequently, the influence of precipitation in this study remains uncertain and warrants more detailed investigation in future research.

### 4.2. Phenology Shifts in Jepson Ecoregions and Impacts over Vegetation Groups

Phenological changes were not only observed at the species level for flowering but patterns also emerged at the Jepson ecoregion level. All findings in DOY flowering related to temperatures and time showed advances, with no delays recorded. This aligns with the fact that the majority of species demonstrated advances, with only two species showing delays under these variables, effectively masking any delaying effects. The clear pattern of advance found in the ecoregions mostly aligned with those ecoregions showing the highest increase in T since 1896, i.e., southernmost ecoregions showed advances in flowering more intensively. However, the plants in the southern ecoregions are better adapted to harder climatic conditions: some species are well adapted to desert conditions, with extreme warm and cold T and almost no P during the year, while other species more related to coastal conditions are adapted to the hot and dry Mediterranean conditions of California’s southwestern coast. Considering the observed temperature trends over the last century and the future projections of continued increases in these areas [109], we expect that phenological timing will continue to shift toward earlier events. Matching with our results, previous studies have reported a phenological advance in the Sonoran Desert and other semi-arid areas [110,111]. This could result in a decoupling from other key biological and ecological interactions, such as migratory hummingbirds [112], native pollinating bees or seed and fruit predation [111,113,114], particularly in deserts where rainfall is highly infrequent and occurs during a specific season. However, despite the reduction in annual P observed in these ecoregions since 1896, our results did not reveal a relationship between annual P and changes in flowering. This may be due to the sporadic and localized nature of rainfall in these areas, which could be obscured when analyzed as a broad variable like annual P. Therefore, while flowering in these ecoregions appears to be primarily driven by T, it would be valuable to include additional ecohydrological variables in future studies.

The northernmost ecoregions and those far from the coast and with higher topographic elevation (i.e., cooler conditions) also experienced advances in flowering, although not as pronounced as those in the southern ecoregions. Ecoregions such as the east of Sierra Nevada, Sierra Nevada and Cascade Ranges have the risk of reproductive structure damage due to earlier flowering [108,115], since these regions are prone to heavy snowfalls and spring frosts. In contrast to southern ecoregions, precipitation decrease was associated with later flowering times, as reported in Rafferty et al. [116]. It is well known that snowmelt in high-altitude areas triggers the onset of flowering for many mountain species [108,117]. Therefore, if precipitation decreases, the amount of available snow in elevated regions also diminishes, and earlier snowmelt leads to earlier spring conditions. This trade-off between the effect of temperatures and the loss of precipitation could cause a counterbalance effect that softens the phenological advances.

The coastal ecoregions showed a north to south increase in the flowering advance matching with the T increase as well. Advances were observed in northwestern and central western California ecoregions due to a decrease in P. These ecoregions are strongly influenced by precipitation coming from the Pacific Ocean [118,119], and the plants living here could present a higher precipitation dependency. In addition, this advancement could reflect the changes found in abundant species of these regions, such as *A. macrophyllum*, *L. interrupta*, *Q. agrifolia*, *Q. durata*, *Q. kelloggii*, *Q. vaccinifolia*, *Q. wislizeni* and *S. sempervirens* (File S3.3).

Phenological changes across entire ecoregions can have negative consequences for both plant species and the organisms that depend on them. For instance, in the Great Valley, butterflies have advanced their phenology due to increased temperatures and decreased winter P [120]. If these advances are not synchronized with the phenology of the plants in this ecoregion, the resulting decoupling between these groups could lead to population collapses and extinctions [121]. Populations of butterflies have already suffered a decline in northern California [122], adding more risks to pollination effectiveness.

Given the observed changes in species and ecoregions, it is reasonable to expect that these shifts will have broader consequences for the vegetation they compose. Many of the affected trees belong to the “California Forests and Woodlands” and “Californian-Vancouverian Montane and Foothill Forest” macrogroups, the former dominated primarily by *Quercus* species and the latter by gymnosperms and *Acer*, among a wide variety of broadleaf tree species. Among the observed changes, the most concerning are those affecting deciduous species at higher altitudes, as they may be particularly vulnerable to frost damage [123].

In contrast, for the “Vancouverian Rainforest” macrogroup, *S. sempervirens* was the only species analyzed. This tree has very high humidity and precipitation requirements. It showed an advancement in F only in response to increasing annual T, with no significant changes linked to P or evapotranspiration. Therefore, it is expected that this species will continue advancing its F phenophase, given that the region it inhabits has experienced rising temperatures, with projections indicating further increases [10]. On the other hand, if P decreases, it may also contribute to earlier flowering but could jeopardize the species’ proper development and survival [124].

Most of the shrubs analyzed belong to the “Cool Interior Chaparral”, “California Chaparral” and “California Coastal Scrub” macrogroups. These groups are expected to experience phenological advances in their species. The primary concern for species in the “Cool Interior Chaparral” macrogroup is the increased risk of frost and cold conditions caused by earlier phenology [125], which could expose the plant’s most vulnerable parts, such as flower buds, new leaf shoots, or flowers. In contrast, the other two chaparral groups, typical of warmer areas, are less at risk from late spring frosts. However, they may face challenges from mismatches with pollinators and other key ecosystem components [126,127]. Nonetheless, we consider the shrub species most in need of attention to be *A. tridentata*, which is part of the “Western North America Tall Sage Shrubland and Steppe” macrogroup. This species was the only one to show a clear delay in all its phenophases. As previously noted, delays may indicate that the species is not effectively tracking climatic changes in its environment. While this delay could help the species avoid issues such as late frosts, it may face other risks, including reduced reproductive success due to mismatches with pollinators, and diminished germination success caused by harsher conditions during the warmer months [103]. Late spring frosts appear to be decreasing due to global warming. For example, in another Mediterranean area (Barcelona, Spain), annual frost events have dropped drastically [27]. This suggests a possible trade-off between the earlier phenology triggered by climate change and a simultaneous reduction in frost risk due to the declining frequency of these events.

The phenological shifts observed in herbaceous species within the “California Annual and Perennial Grasslands” macrogroup could negatively impact the survival and persistence of native species in this group. Of particular concern is *Amsinckia menziesii*, as its early flowering provides a crucial resource for many pollinators in California [128]. However, its flowering period appears to be constrained, and this mismatch could jeopardize both the survival of these pollinators and the reproductive success of the plant itself [116].

Earlier flowering can have negative consequences in the reproductive success of plants [31,129,130] reducing the long-term survival of the species (but see [131,132]). Moreover, the complex interplay between climatic factors and phenological responses underscores the importance of integrated, multidisciplinary approaches in conservation efforts. This study emphasizes the role of herbarium specimens in monitoring climate change while also revealing the general trends affecting many plant species. These findings indicate a paradigm shift in the timing of phenological activity in California.

## 5. Conclusions

Climate change has led to significant phenological advances in most of the 29 plant species analyzed, especially during flowering. Temperature variables emerged as the primary drivers of phenological change, while the effects of water-related variables were more variable and species specific. The phenological shifts observed in southern Jepson ecoregions highlight the pronounced impact of warming trends, with individuals in these regions showing greater advances in timing compared to those in northern ecoregions.

Observed phenological advances may result in mismatches between plants and their ecological partners and increase the species’ vulnerability to frost damage by exposing sensitive structures. The findings reveal the ongoing and multiple impacts of climate change on California’s vegetation, showing an urgent need for conservation strategies.

This study underscores the importance of using herbarium specimens as a powerful tool to track climate change impacts across diverse plant species and their vegetation. Additionally, it is important to complement these historical records with field monitoring efforts to better predict future changes and guide conservation strategies.

## Data Availability

The data that support the findings of this study are contained within the article and the Supplementary Materials.

## Supplementary Materials

The following supporting information can be downloaded as a document file at: https://www.mdpi.com/article/10.3390/plants14060843/s1.
