# Supplementary material for: Phenological Shifts Since 1830 in 29 Native Plant Species of California and Their Responses to Historical Climate Change
Source: Plants (Basel). 2025 Mar 7;14(6):843. doi: 10.3390/plants14060843 (PMC11945038; doi:10.3390/plants14060843)
Supplement: Supplementary file 1 [file plants-14-00843-s001.zip › File S1 - Supplementary_Figures.pdf]

Supplementary Material for

Phenological shifts since 1830 in 29 native plant species of California and their responses to historical climate change

Andros Solakis-Tena [1]*, Noelia Hidalgo-Triana [1], Ryan Boynton [2] and James H. Thorne [2]

[1] Department of Botany and Plant Physiology (Botany Area), Faculty of Science, University of Málaga, Málaga 29010, Spain; andros@uma.es (A.S.T); nhidalgo@uma.es (N.H.T.)
[2] Department of Environmental Science and Policy, University of California, Davis 95616, USA; rmboynton@ucdavis.edu (R.B.); jhthorne@ucdavis.edu (J.H.T.)
* Correspondence: andros@uma.es

File **S1 - Figures**

Climatic variables used in the models

We correlated all climate variables (annual and monthly) for two datasets:

1. Climate across all points in California.
2. Climate by Jepson ecoregion.

To create the correlation matrix, we used preserved specimen data points rather than the entire California climate dataset (which contains 5 million points) because the unused data were not relevant to our analysis. Annual and monthly variables related to evap and rchrunscaler were excluded due to the high proportion of missing (NA) values. Records prior to 1896 were removed due to the lack of corresponding climatic data.

The results from the first run of correlations for (1) climate across all points in California are as follows:

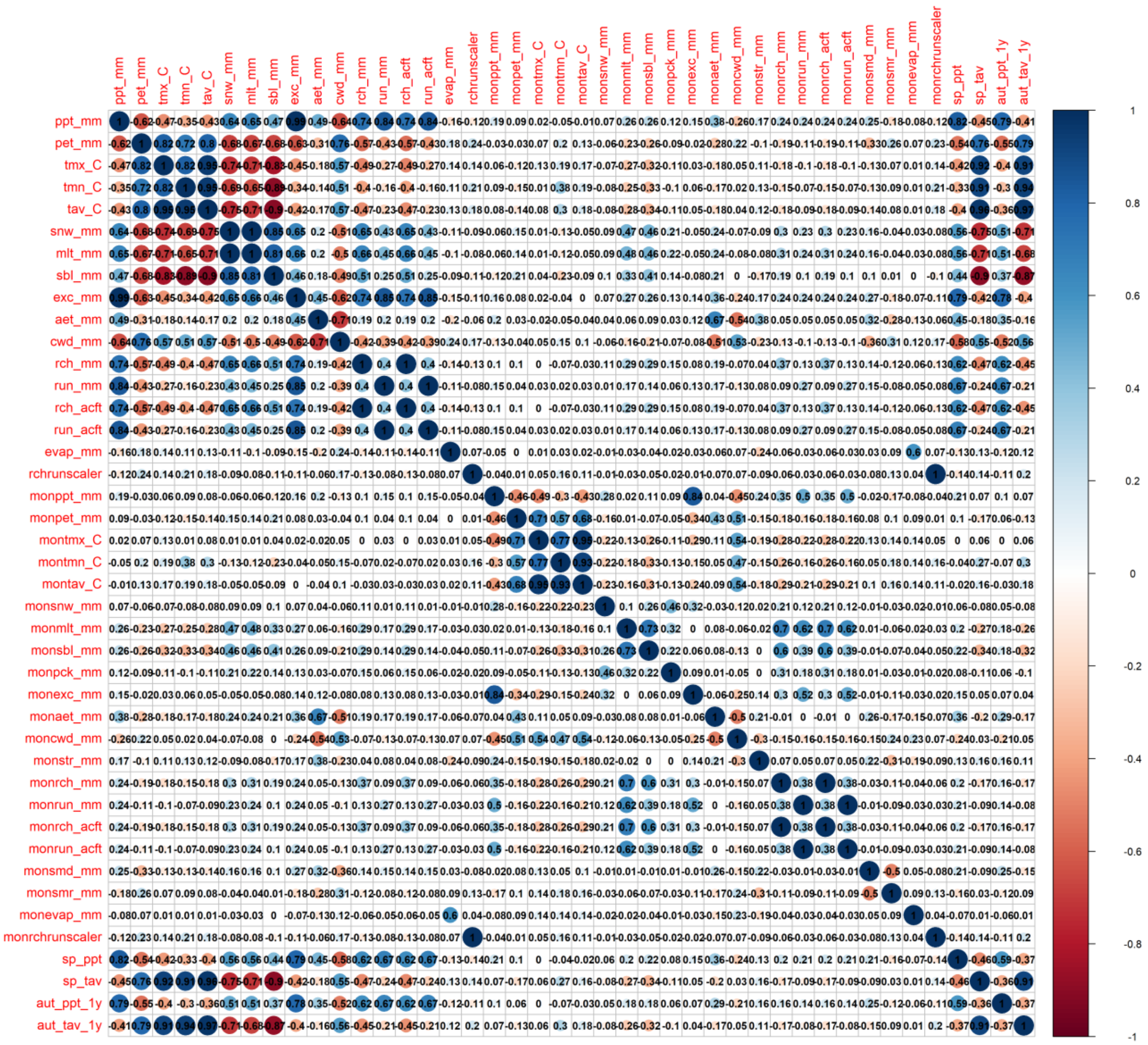

Figure S1 Initial correlation analysis for climatic data across all points in California. Correlation matrix of climatic variables based on data derived from 270m resolution points across the entire state of California. The climatic data were extracted from the Basin Characterization Model (Flint et al., 2021).

The results from the first run of correlations for (2) Jepson ecoregion climate are as follows:

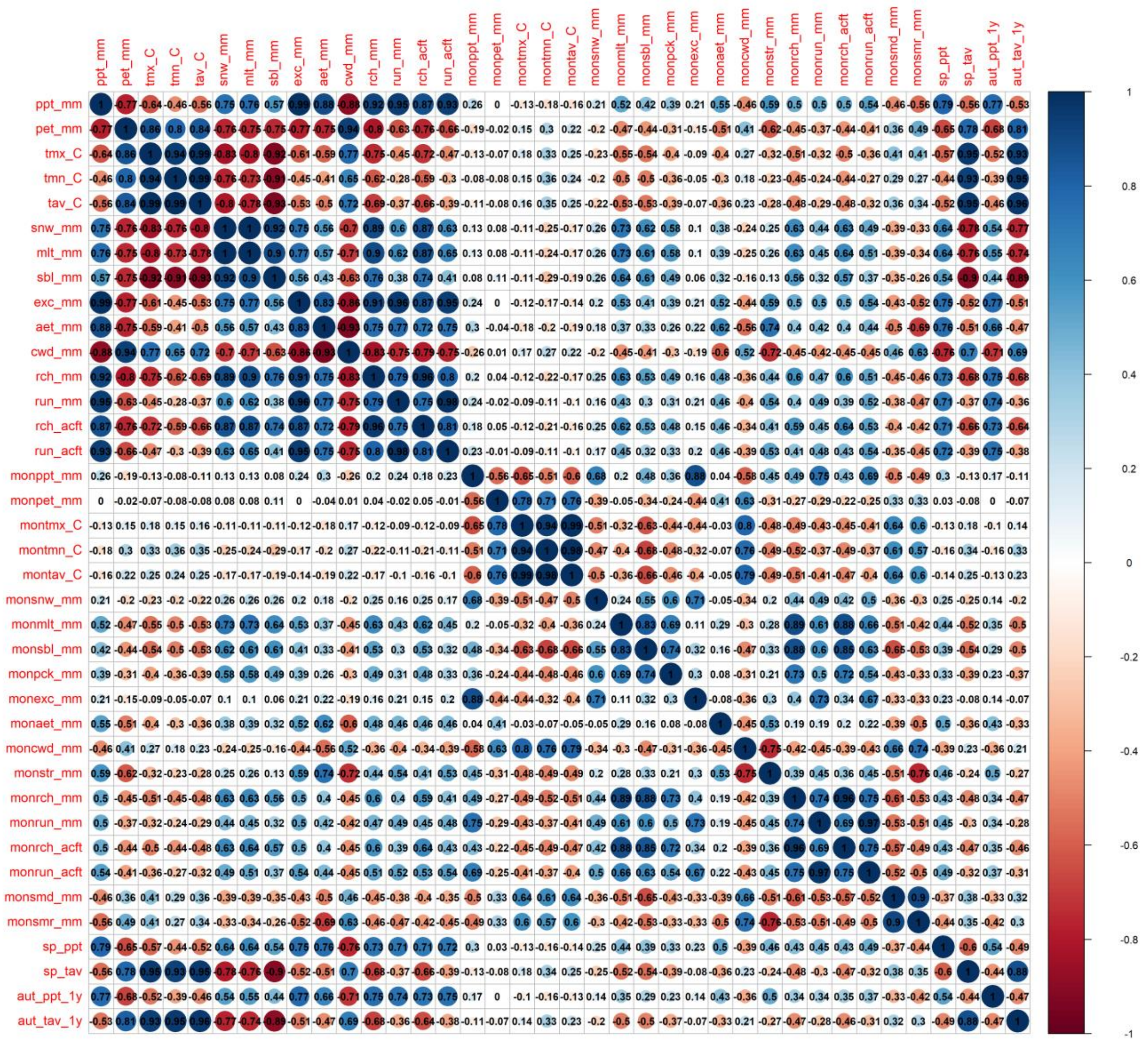

Figure S2. Initial correlation analysis for climatic data in Jepson regions. Correlation matrix of climatic variables based on data calculated for the Jepson regions in California. The climatic data were extracted from the Basin Characterization Model (Flint et al., 2021).

Phenological calendars

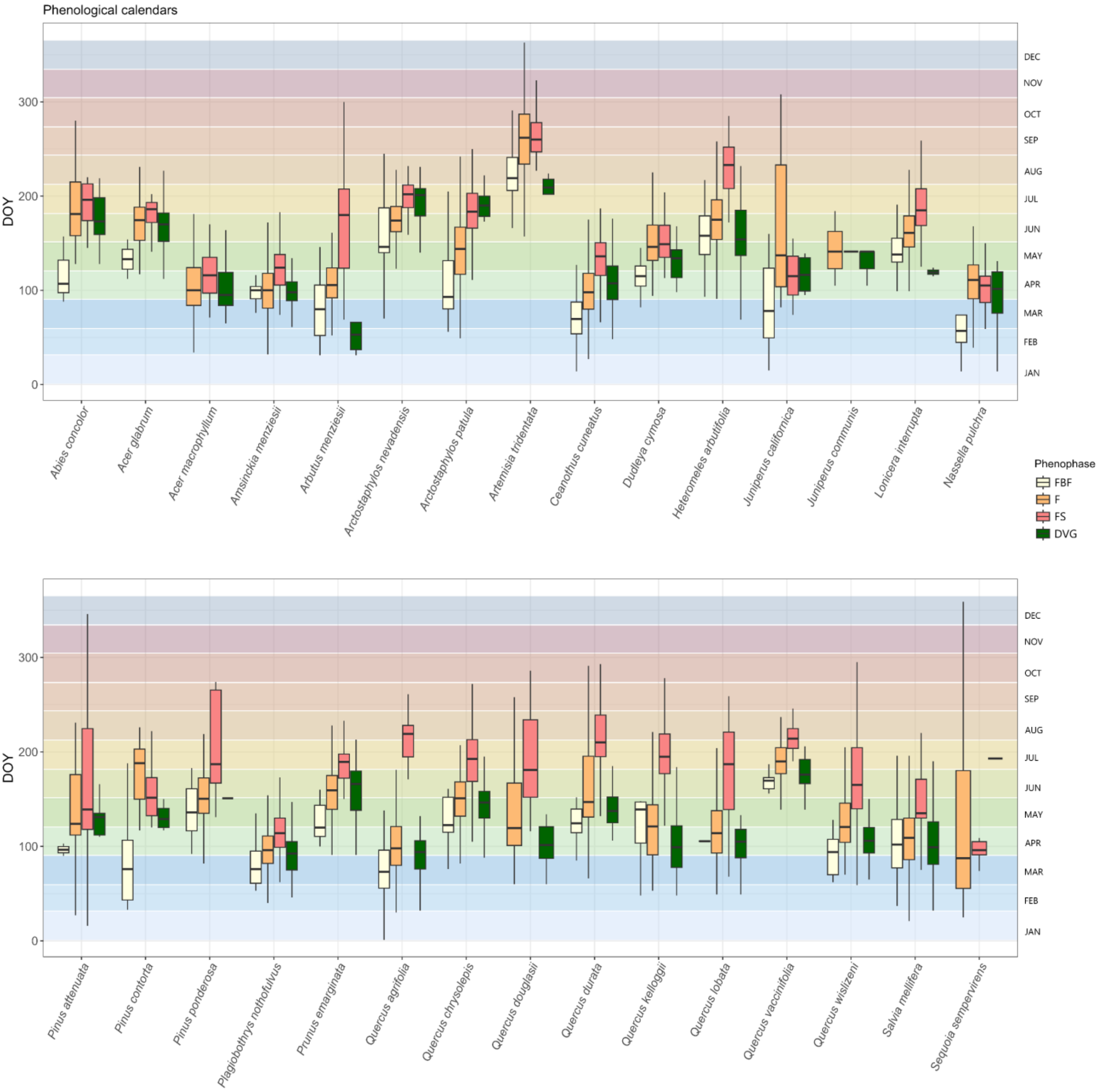

Figure S3. Phenological calendars for each studied species based on observed preserved specimens.

Climatic context: Trends in California

Climatic data were analyzed to identify the main trends across California. The following plots are based on monthly, spring, and annual temperatures and precipitation in different contexts. The general findings indicate that precipitation tends to decrease overall, although increases were observed in some cases, while temperatures consistently increase across all scenarios. These variables were regressed against the Day of Year (DOY) for each phenophase by species and Jepson ecoregion to track phenological patterns (advancement, delay, or no significant changes).

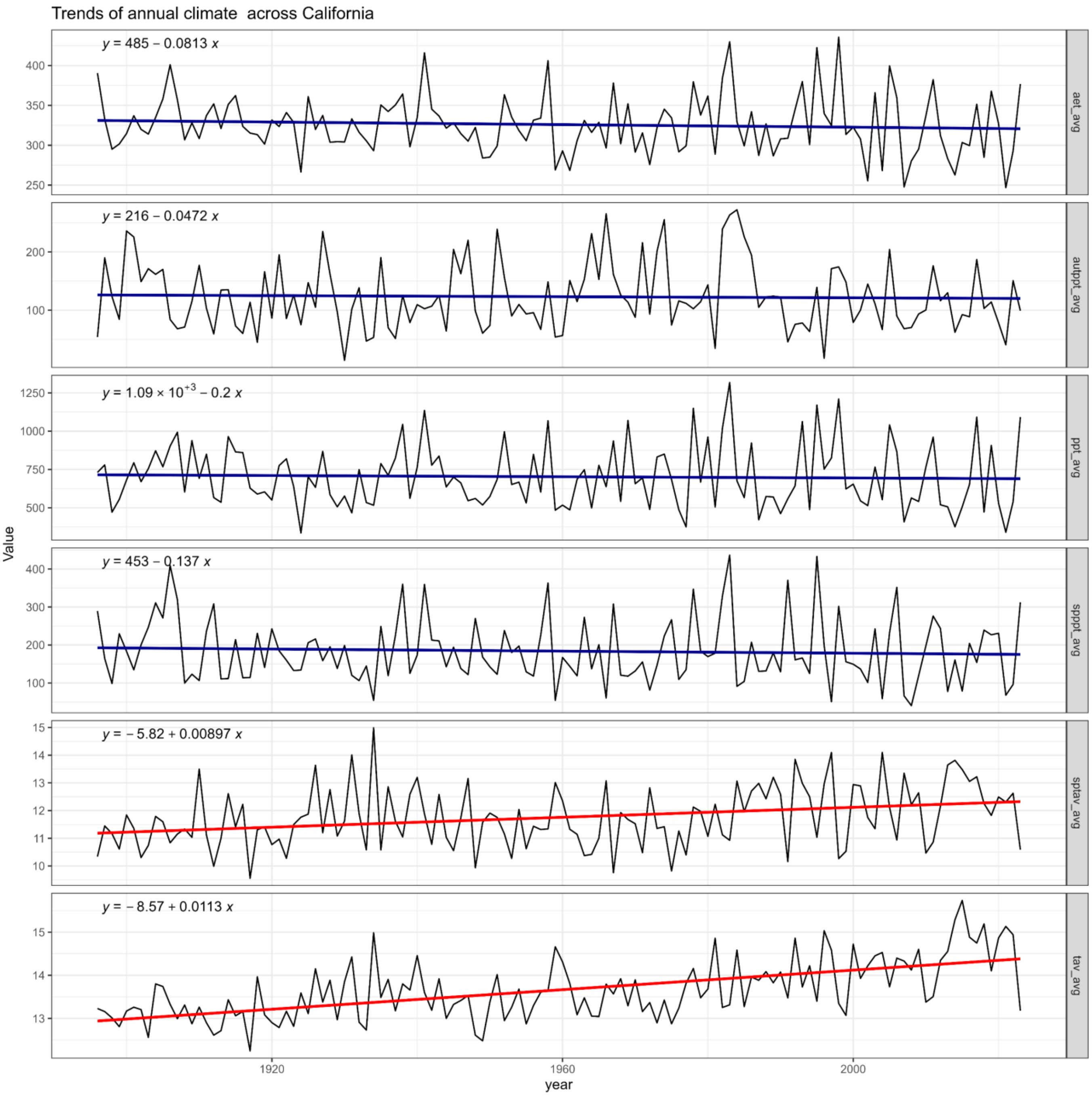

Figure S4. Climatic trends across the state of California from the late 19th century to the present. Variables are presented from top to bottom: annual AET, autumn P, total annual P, spring P, spring T, and mean annual T. Temperatures show a consistent increasing trend, while precipitation tends to decrease.
