# Supplementary material for: Phenological Shifts Since 1830 in 29 Native Plant Species of California and Their Responses to Historical Climate Change
Source: Plants (Basel). 2025 Mar 7;14(6):843. doi: 10.3390/plants14060843 (PMC11945038; doi:10.3390/plants14060843)
Supplement: Supplementary file 1 [file plants-14-00843-s001.zip › File S1 - Supplementary_Tables.pdf]

Supplementary Material for

Phenological shifts since 1830 in 29 native plant species of California and their responses to historical climate change

Andros Solakis-Tena [1]*, Noelia Hidalgo-Triana [1], Ryan Boynton [2] and James H. Thorne [2]

[1] Department of Botany and Plant Physiology (Botany Area), Faculty of Science, University of Málaga, Málaga 29010, Spain; andros@uma.es (A.S.T); nhidalgo@uma.es (N.H.T.)
[2] Department of Environmental Science and Policy, University of California, Davis 95616, USA; rmboynton@ucdavis.edu (R.B.); jhthorne@ucdavis.edu (J.H.T.)
* Correspondence: andros@uma.es

File **S1 - Tables**

Table S1. Target species of the study, family and its biological type based on Hickman (1993), and vegetation macrogroup class based on the USNVC.

Species	Growth form	Family	Vegetation class (macrogroup)
Abies concolor	Tree	Pinaceae	MG009: Macrogroup California Forest and Woodland/MG023: Macrogroup Californian-Vancouverian Montane and Foothill Forest
Acer glabrum	Tree/shrub	Sapindaceae	MG023: Macrogroup Californian-Vancouverian Montane and Foothill Forest/MG114: Macrogroup Vancouverian Cliff, Scree, and Other Rock Vegetation; endemic and limited to California
Acer macrophyllum	Tree	Sapindaceae	MG023: Macrogroup Californian-Vancouverian Montane and Foothill Forest
Amsinckia menziesii	Annual herb	Boraginaceae	MG045: Macrogroup California Annual and Perennial Grassland
Arbutus menziesii	Tree	Ericaceae	MG023: Macrogroup Californian-Vancouverian Montane and Foothill Forest
Arctostaphylos nevadensis	Shrub	Ericaceae	MG052 Cool Interior Chaparral Macrogroup
Arctostaphylos patula	Shrub	Ericaceae	MG052 Cool Interior Chaparral Macrogroup
Artemisia tridentata	Shrub	Asteraceae	MG096: Macrogroup Western North America Tall Sage Shrubland and Steppe
Ceanothus cuneatus	Shrub	Rhamnaceae	MG043: Macrogroup California Chaparral
Dudleya cymosa	Perennial herb	Crassulaceae	MG110: Macrogroup California Cliff, Scree, and Other Rock Vegetation
Heteromeles arbutifolia	Shrub	Rosaceae	MG043: Macrogroup California Chaparral
Juniperus californica	Shrub	Cupressaceae	MG009: Macrogroup California Forest and Woodland
Juniperus communis	Shrub	Cupressaceae	MG009: Macrogroup California Forest and Woodland/MG023: Macrogroup Californian-Vancouverian Montane and Foothill Forest
Lonicera interrupta	Vine/shrub	Caprifoliaceae	MG009: Macrogroup California Forest and Woodland.
Nasella pulchra	Perennial grasslike herb	Poaceae	MG045: Macrogroup California Annual and Perennial Grassland
Pinus attenuata	Tree	Pinaceae	MG009: Macrogroup California Forest and Woodland
Pinus contorta	Tree	Rosaceae	MG020: Macrogroup Rocky Mountain Subalpine and High Montane Conifer Forest/MG024: Macrogroup Vancouverian Rainforest.
Pinus ponderosa	Tree	Pinaceae	MG009: Macrogroup California Forest and Woodland/MG023: Macrogroup Californian-Vancouverian Montane and Foothill Forest

Plagiobothrys nothofulvus	Annual herb	Boraginaceae	MG045: Macrogroup California Annual and Perennial Grassland
Prunus emarginata	Tree/shrub	Rosaceae	MG052 Cool Interior Chaparral Macrogroup
Quercus agrifolia	Tree	Fagaceae	MG009: Macrogroup California Forest and Woodland
Quercus chrysolepis	Tree	Fagaceae	MG009: Macrogroup California Forest and Woodland
Quercus douglasii	Tree	Fagaceae	MG009: Macrogroup California Forest and Woodland
Quercus durata	Shrub	Fagaceae	MG043: Macrogroup California Chaparral
Quercus kelloggii	Tree	Fagaceae	MG009: Macrogroup California Forest and Woodland/MG023: Macrogroup Californian-Vancouverian Montane and Foothill Forest
Quercus lobata	Tree	Fagaceae	MG009: Macrogroup California Forest and Woodland/MG023: Macrogroup Californian-Vancouverian Montane and Foothill Forest
Quercus vaccinnifolia	Shrub	Fagaceae	MG052 Cool Interior Chaparral Macrogroup
Quercus wislizeni	Tree/shrub	Fagaceae	MG009: Macrogroup California Forest and Woodland/MG023: Macrogroup Californian-Vancouverian Montane and Foothill Forest
Salvia mellifera	Shrub	Lamiaceae	MG044: Macrogroup California Coastal Scrub
Sequoia sempervirens	Tree	Cupressaceae	MG024: Macrogroup Vancouverian Rainforest

Climatic variables used in the models

Here are presented the climatic variables used in the models by doing a preselection of the interesting variables, removing the correlated ones and selecting the most important.

Table S2. List of the 42 initial variables and its summary (max., min. and avg.).

Abreviation	Name	Units	Average	Minimum	Maximum
ppt_mm	total annual P	mm	702.6	45.4	7850.5
pet_mm	annual potential evapotranspiration	mm	1245.8	336.6	1598.8
tmx_C	annual avg of the max T	°C	20.6	2.6	31.3
tmn_C	annual avg of the min T	°C	7.0	-10.6	18.1
tav_C	mean annual T	°C	13.8	-4.0	24.4
snw_mm	annual snowfall	mm	151.5	0.0	2594.7
mlt_mm	annual snowmelt	mm	125.0	0.0	2483.2
sbl_mm	annual sublimation	mm	26.4	0.0	163.7
exc_mm	annual excess water (ppt-pet)	mm	398.7	0.0	6607.4
aet_mm	annual actual evapotranspiration	mm	323.0	0.0	947.1
cwd_mm	annual climatic water deficit (pet-aet)	mm	912.8	0.0	1560.2
rch_mm	annual recharge	mm	157.3	0.0	2453.6
run_mm	annual runoff	mm	190.2	0.0	4586.6
rch_acft	annual recharge (acft)	acre-feet	9.3	0.0	145.0
run_acft	annual runoff (acft)	acre-feet	11.2	0.0	271.1
evap_mm	annual evapotranspiration from soil	mm	5.6	0.0	250.0
rchrunscaler	scaler to enhance annual run/rch in streamflow	mm	890.2	5.7	1726.5
monppt_mm	total monthly P	mm	31.6	0.0	777.5
monpet_mm	monthly potential evapotranspiration	mm	129.2	17.3	212.5
montmx_C	monthly avg of the max T	°C	22.5	3.4	38.2
montmn_C	monthly avg of the min T	°C	8.2	-9.1	22.1
montav_C	mean monthly T	°C	15.3	-2.3	28.3
monsnw_mm	monthly snowfall	mm	0.6	0.0	500.2
monmlt_mm	monthly snowmelt	mm	7.4	0.0	899.8
monsbl_mm	monthly sublimation	mm	0.9	0.0	27.2
monpck_mm	monthly snowpack	mm	1.9	0.0	1360.7
monexc_mm	monthly snowpack	mm	6.8	0.0	731.2

monaet_mm	monthly actual evapotranspiration	mm	44.4	0.0	198.5
moncwd_mm	monthly climatic water deficit (pet-aet)	mm	83.8	0.0	205.1
monstr_mm	monthly soil moisture storage	mm	441.7	0.0	2613.0
monrch_mm	monthly recharge	mm	8.2	0.0	525.4
monrun_mm	monthly runoff	mm	8.2	0.0	800.0
monrch_acft	annual recharge (acft)	acre-feet	0.5	0.0	31.0
monrun_acft	annual runoff (acft)	acre-feet	0.5	0.0	47.3
monsmd_mm	monthly soil water deficit to produce recharge	mm	311.5	0.0	2074.8
monsmr_mm	monthly soil water deficit to produce runoff	mm	-3299.8	0.0	2400.0
monevap_mm	monthyl evapotranspiration from soil	mm	0.3	0.0	33.5
monrchrunscaler	scaler to enhance monthly run/rch in streamflow	mm	818.9	4.1	1588.6
sp_ppt	spring P (avg of MAM)	mm	179.5	0.0	1408.7
sp_tav	spring T (avg of MAM)	°C	11.9	-6.9	24.8
aut_ppt_1y	autumn P (avg of SON of the previous year)	mm	122.0	0.0	2288.1
aut_tav_1y	autumn T (avg of SON of the previous year)	°C	15.2	-3.8	25.9

Number of specimens by phenophase for modeling

Table S3. Target species of the study. Species highlighted in red indicate those that did not meet the minimum number of preserved specimens required for each phenophase (>10) to be included in the modeling.

Phenophase	Species	N	Phenophase	Species	N
FBF	*Abies concolor*	3	FS	*Abies concolor*	13
	Acer glabrum	2		*Acer glabrum*	24
	Acer macrophyllum	0		*Acer macrophyllum*	49
	Amsinckia menziesii	33		*Amsinckia menziesii*	111
	Arbutus menziesii	16		*Arbutus menziesii*	18
	Arctostaphylos nevadensis	0		*Arctostaphylos nevadensis*	40
	Arctostaphylos patula	18		*Arctostaphylos patula*	100
	Artemisia tridentata	77		*Artemisia tridentata*	27
	Ceanothus cuneatus	56		*Ceanothus cuneatus*	128
	Dudleya cymosa	12		*Dudleya cymosa*	61
	Heteromeles arbutifolia	53		*Heteromeles arbutifolia*	29
	Juniperus californica	11		*Juniperus californica*	60
	Juniperus communis	0		*Juniperus communis*	1
	Lonicera interrupta	30		*Lonicera interrupta*	50
	Nassella pulchra	4		*Nassella pulchra*	152
	Pinus attenuata	2		*Pinus attenuata*	28
	Pinus contorta	6		*Pinus contorta*	8
	Pinus ponderosa	7		*Pinus ponderosa*	10
	Plagiobothrys nothofulvus	25		*Plagiobothrys nothofulvus*	206
	Prunus emarginata	15		*Prunus emarginata*	66
	Quercus agrifolia	30		*Quercus agrifolia*	28
	Quercus chrysolepis	12		*Quercus chrysolepis*	140
	Quercus douglasii	0		*Quercus douglasii*	37
	Quercus durata	10		*Quercus durata*	29
	Quercus kelloggii	7		*Quercus kelloggii*	89
	Quercus lobata	2		*Quercus lobata*	29
	Quercus vaccinifolia	14		*Quercus vaccinifolia*	38
	Quercus wislizeni	19		*Quercus wislizeni*	112
	Salvia mellifera	22		*Salvia mellifera*	57
	Sequoia sempervirens	0		*Sequoia sempervirens*	9
F	*Abies concolor*	45	DVG	*Abies concolor*	14
	Acer glabrum	50		*Acer glabrum*	29

	Acer macrophyllum	265		*Acer macrophyllum*	61
	Amsinckia menziesii	662		*Amsinckia menziesii*	49
	Arbutus menziesii	130		*Arbutus menziesii*	5
	Arctostaphylos nevadensis	222		*Arctostaphylos nevadensis*	29
	Arctostaphylos patula	385		*Arctostaphylos patula*	15
	Artemisia tridentata	635		*Artemisia tridentata*	5
	Ceanothus cuneatus	753		*Ceanothus cuneatus*	126
	Dudleya cymosa	336		*Dudleya cymosa*	11
	Heteromeles arbutifolia	492		*Heteromeles arbutifolia*	15
	Juniperus californica	22		*Juniperus californica*	8
	Juniperus communis	3		*Juniperus communis*	3
	Lonicera interrupta	303		*Lonicera interrupta*	3
	Nassella pulchra	89		*Nassella pulchra*	14
	Pinus attenuata	27		*Pinus attenuata*	9
	Pinus contorta	25		*Pinus contorta*	4
	Pinus ponderosa	32		*Pinus ponderosa*	1
	Plagiobothrys nothofulvus	655		*Plagiobothrys nothofulvus*	88
	Prunus emarginata	406		*Prunus emarginata*	90
	Quercus agrifolia	361		*Quercus agrifolia*	103
	Quercus chrysolepis	400		*Quercus chrysolepis*	104
	Quercus douglasii	110		*Quercus douglasii*	34
	Quercus durata	179		*Quercus durata*	64
	Quercus kelloggii	192		*Quercus kelloggii*	58
	Quercus lobata	122		*Quercus lobata*	29
	Quercus vaccinifolia	163		*Quercus vaccinifolia*	43
	Quercus wislizeni	270		*Quercus wislizeni*	86
	Salvia mellifera	1206		*Salvia mellifera*	85
	Sequoia sempervirens	38		*Sequoia sempervirens*	1

Table S4. Jepson ecoregions with the number of preserved specimens in flowering and the number of species represented.

Region	Number of species	Number of registers in F
Cascade Ranges	25	364
Central Western CA	23	2121
East of Sierra Nevada	14	373
Great Valley	18	485
Modoc Plateau	12	96
Mojave Desert	18	409
NorthWestern CA	30	1901
Sierra Nevada	26	2252
Sonoran Desert	8	98
Southwestern CA	26	5609

Climatic context: Trends in California

Table S5. Linear models of climatic trends for key variables from 1896 to 2023. Annual variables are listed from top to bottom: annual T, spring T, total P, spring P, autumn P, and AET.

AVERAGED POINTS BY YEAR RESULTS								
**Variable**	**Intercept**	**Slope**	**p_value**	**SE_Intercept**	**SE_Slope**	**R^2**	**BP_p_value**	**KS_p_value**
tav_avg	-8.57	0.011	0.000	2.594	0.001	0.368	0.037	0.963
sptav_avg	-5.82	0.009	0.000	4.903	0.003	0.093	0.443	0.852
ppt_avg	1094.39	-0.200	0.684	960.549	0.490	0.001	0.003	0.109
spppt_avg	453.06	-0.137	0.502	399.364	0.204	0.004	0.242	0.133
autppt_avg	215.77	-0.047	0.733	271.055	0.138	0.001	0.902	0.137
aet_avg	485.18	-0.081	0.358	172.575	0.088	0.007	0.003	0.248
ALL POINTS RESULTS								
**Variable**	**Intercept**	**Slope**	**p_value**	**SE_Intercept**	**SE_Slope**	**R^2**	**BP_p_value**	**KS_p_value**

tav_C	-8.57	0.011	0	0.17	8.51E-05	0.01517	1.33E-35	0
sp_tav	-5.82	0.009	0	0.18	9.46E-05	0.007718	4.94E-107	0
ppt_mm	1094.39	-0.200	7.16E-49	26.68	0.013613	0.000187	8.58E-35	0
sp_ppt	453.06	-0.137	1.87E-246	8.03	0.004096	0.000973	3.30E-77	0
aut_ppt_1y	215.77	-0.047	1.25E-38	7.12	0.003633	0.000146	8.84E-25	0
aet_mm	485.18	-0.081	6.16E-92	7.83	0.003996	0.000358	5.02E-157	0

Table S6. Total change in each climatic variable over the past 127 years (1896–2023). Annual variables are listed from top to bottom: annual T, spring T, total P, spring P, autumn P, and AET.

Variable	*127 years (1896-2023)	Units
tav_avg	1.44	ºC
sptav_avg	1.14	ºC
ppt_avg	-25.40	mm
spppt_avg	-17.44	mm
autppt_avg	-6.00	mm
aet_avg	-10.32	mm
