# Supplementary material for: Phenological Shifts Since 1830 in 29 Native Plant Species of California and Their Responses to Historical Climate Change
Source: Plants (Basel). 2025 Mar 7;14(6):843. doi: 10.3390/plants14060843 (PMC11945038; doi:10.3390/plants14060843)
Supplement: Supplementary file 1 [file plants-14-00843-s001.zip › File S1 - Vegetation and ecoregions.pdf]

Supplementary Material for

Phenological shifts since 1830 in 29 native plant species of California and their responses to historical climate change

Andros Solakis-Tena [1*], Noelia Hidalgo-Triana [1], Ryan Boynton [2] and James H. Thorne [2]

[1] Department of Botany and Plant Physiology (Botany Area), Faculty of Science, University of Málaga, Málaga 29010, Spain; andros@uma.es (A.S.T); nhidalgo@uma.es (N.H.T.)
[2] Department of Environmental Science and Policy, University of California, Davis 95616, USA; rmboynton@ucdavis.edu (R.B.); jhthorne@ucdavis.edu (J.H.T.)
* Correspondence: andros@uma.es

File **S1 – Vegetation and ecoregions**

Description of the main vegetation present in the ecoregions

Northwestern CA is the wettest ecoregion of the state and encompasses coastal (prairies, marshes and closed-cone-pine/cypress or grand-fir forests) and chaparral communities with the dominate forest types being redwoods (*Sequoia sempervirens* (D.Don) Endl.), mixed-evergreen and mixed-hardwood forests. The Cascade Ranges are comprised of chaparral communities and blue-oak (*Quercus douglasii* Hook. & Arn.) and foothill-pine woodlands in the lower elevation areas, and ponderosa-pine (*Pinus ponderosa* Douglas ex C.Lawson), lodgepole-pine (*Pinus contorta* Douglas ex Loudon) and montane fir/pine forests in the high elevation areas. The next ecoregion to the east is the Modoc Plateau. It is a high plateau (1300-1800 m) whose vegetation is mainly dominated by juniper woodlands and sagebrush (*Artemisia tridentata* Nutt.) steppe, but also montane fir/pine forests and ponderosa-pine and Jeffrey-pine (*Pinus jeffreyi* Balf.) forests. In the Sierra Nevada mountains ecoregion, the foothills (<800 m) are dominated by grasslands, chaparral and blue-oak/foothill-pine woodlands, while the high Sierra (below 800 up to 4400 m) contains lower montane ponderosa-pine, white fir (*Abies concolor* (Gordon & Glend.) Lindl. ex Hildebr.), giant-sequoia (*Sequoiadendron giganteum* (Lindl.) J.Buchholz), upper montane red-fir (*Abies magnifica* A.Murray bis), Jeffrey-pine, lodgepole-pine, subalpine mountain-hemlock (*Tsuga mertensiana* (Bong.) Carrière) and whitebark-pine (*Pinus albicaulis* Engelm.) forests. In the East of Sierra Nevada ecoregion (1100-4330 m) the vegetation is dominated by sagebrush steppe, pinyon/juniper woodlands, riparian cottonwood-dominated communities, Jeffrey-pine forests, subalpine-fir/pine forests and alpine communities. The natural vegetation of the Great Valley is dominated by grasslands (Californian prairies), marshes, riparian woodlands and valley-oak (*Quercus lobata* Née) woodlands. The Central Western CA ecoregion contains salt marshes, coastal prairies, dry oak/pine woodlands, chaparral, and some wet redwood and mixed-hardwood forests. The Southwestern CA ecoregion contains similar vegetation as the Central Western, but due to its hotter and drier conditions it is mostly characterized by chaparral communities, southern oak forests, or dry montane forests with white or Jeffrey firs, sugar or lodgepole pines. The two ecoregions in the southeastern portion of the state are desert types. The Mojave Desert is mainly dominated by the Mojave creosote-bush (*Larrea tridentata* (DC.) Coville) and saltbush scrubs, with pinyon-juniper woodlands in the high desert mountains. It has greater temperature ranges and more diverse elevations than the Sonoran Desert ecoregion to the southeast, which is dominated by the Sonoran creosote-bush scrub.
