# Supplementary figures and images for: Phenological Shifts Since 1830 in 29 Native Plant Species of California and Their Responses to Historical Climate Change (part 2 of 2)
Source: Plants (Basel). 2025 Mar 7;14(6):843. doi: 10.3390/plants14060843 (PMC11945038; doi:10.3390/plants14060843)

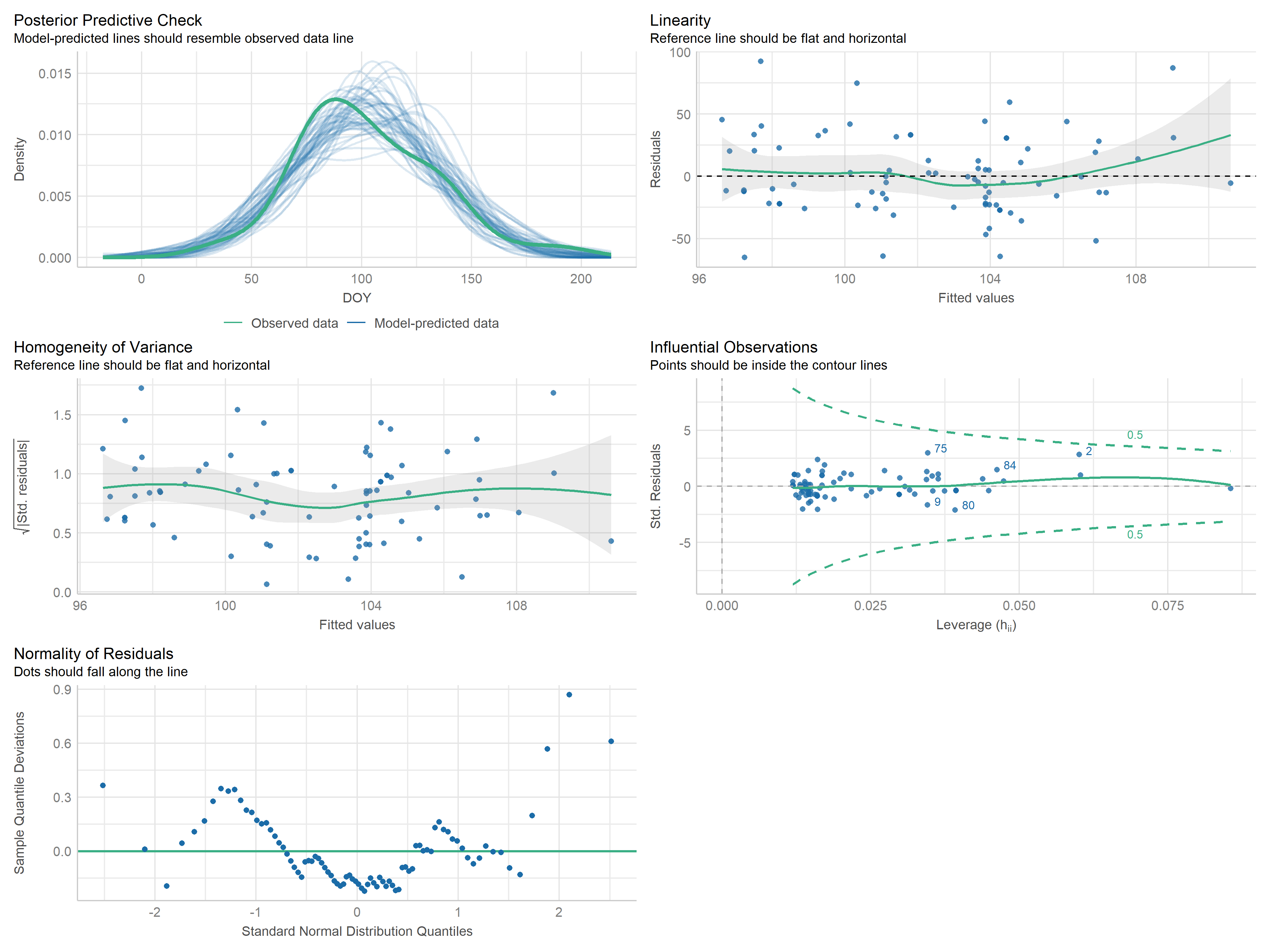

Supplement: Supplementary file 1 [file plants-14-00843-s001.zip › File S2-Species/S2.1-DOYvsYears/1_LM/Plots/Residuals_DVG_Salvia mellifera.png]

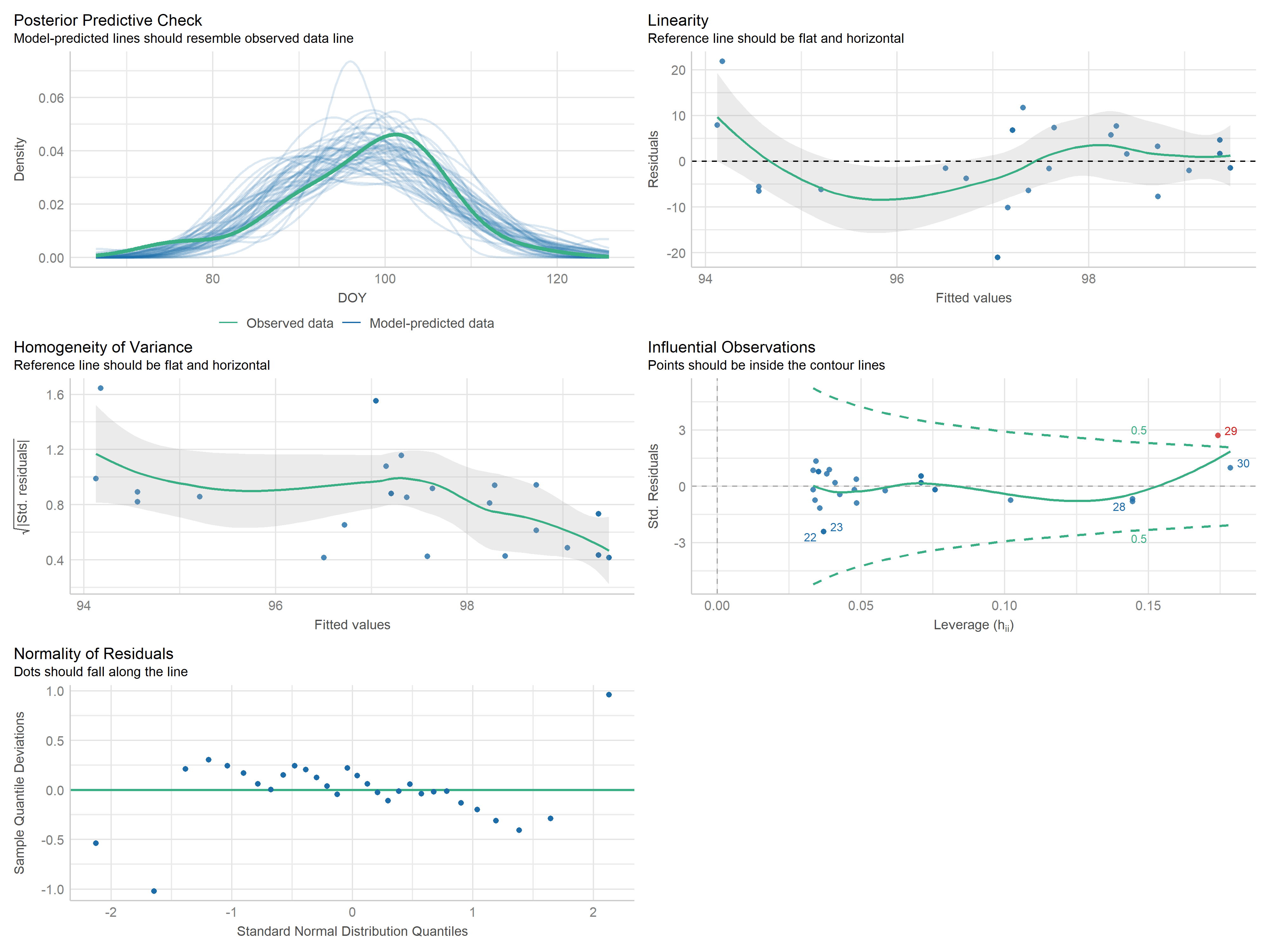

Supplement: Supplementary file 1 [file plants-14-00843-s001.zip › File S2-Species/S2.1-DOYvsYears/1_LM/Plots/Residuals_FBF_Amsinckia menziesii.png]

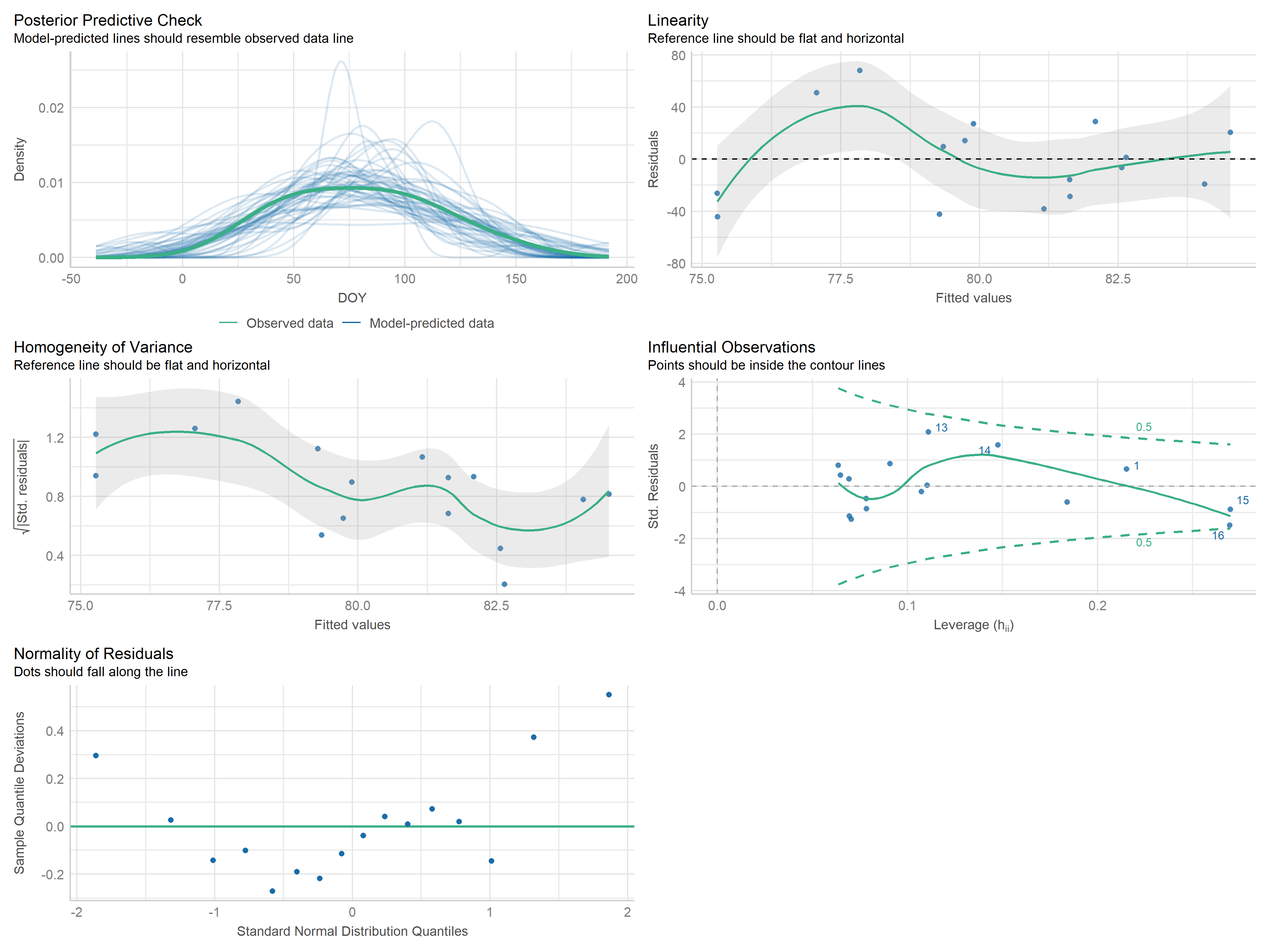

Supplement: Supplementary file 1 [file plants-14-00843-s001.zip › File S2-Species/S2.1-DOYvsYears/1_LM/Plots/Residuals_FBF_Arbutus menziesii.png]

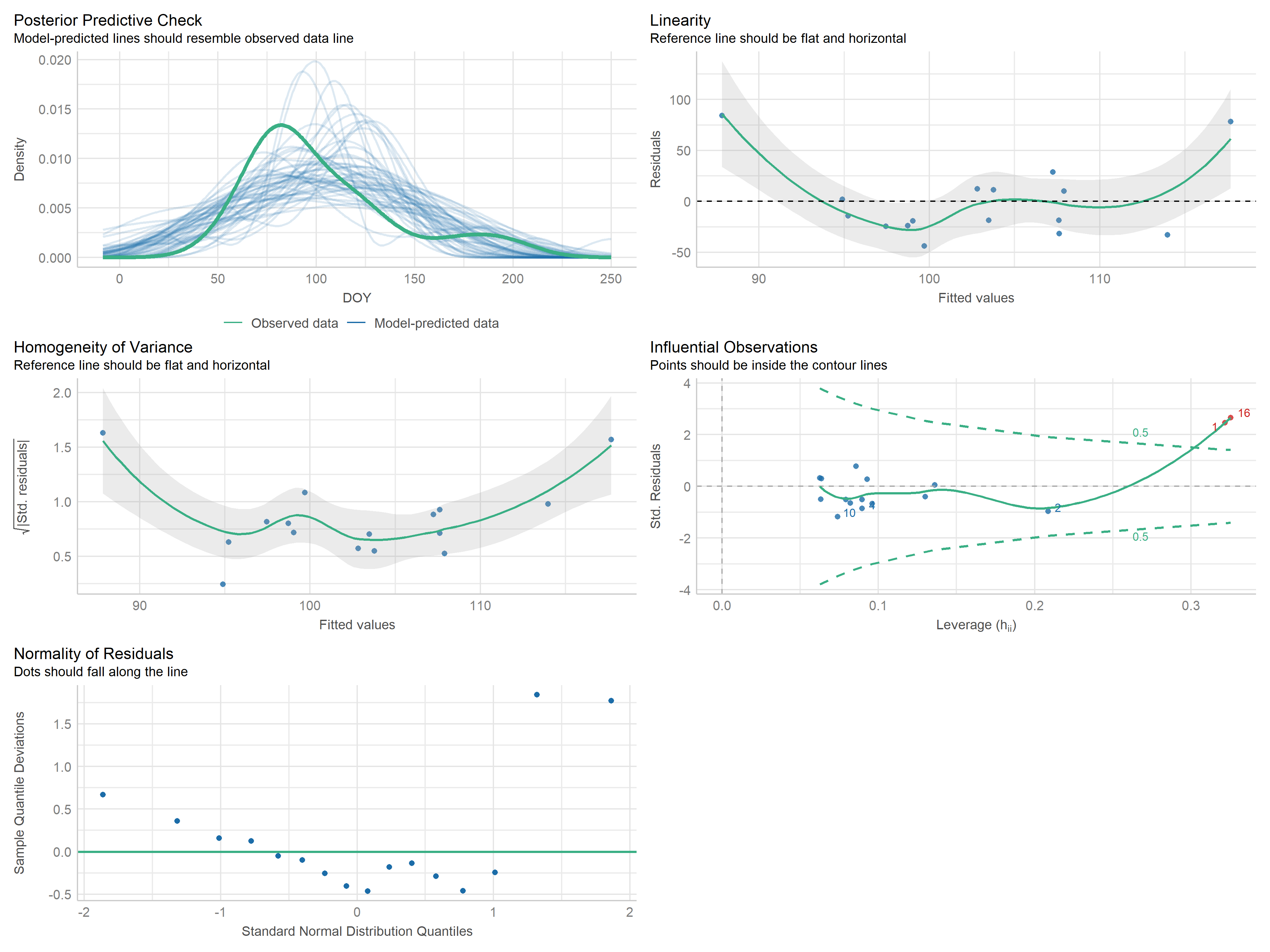

Supplement: Supplementary file 1 [file plants-14-00843-s001.zip › File S2-Species/S2.1-DOYvsYears/1_LM/Plots/Residuals_FBF_Arctostaphylos patula.png]

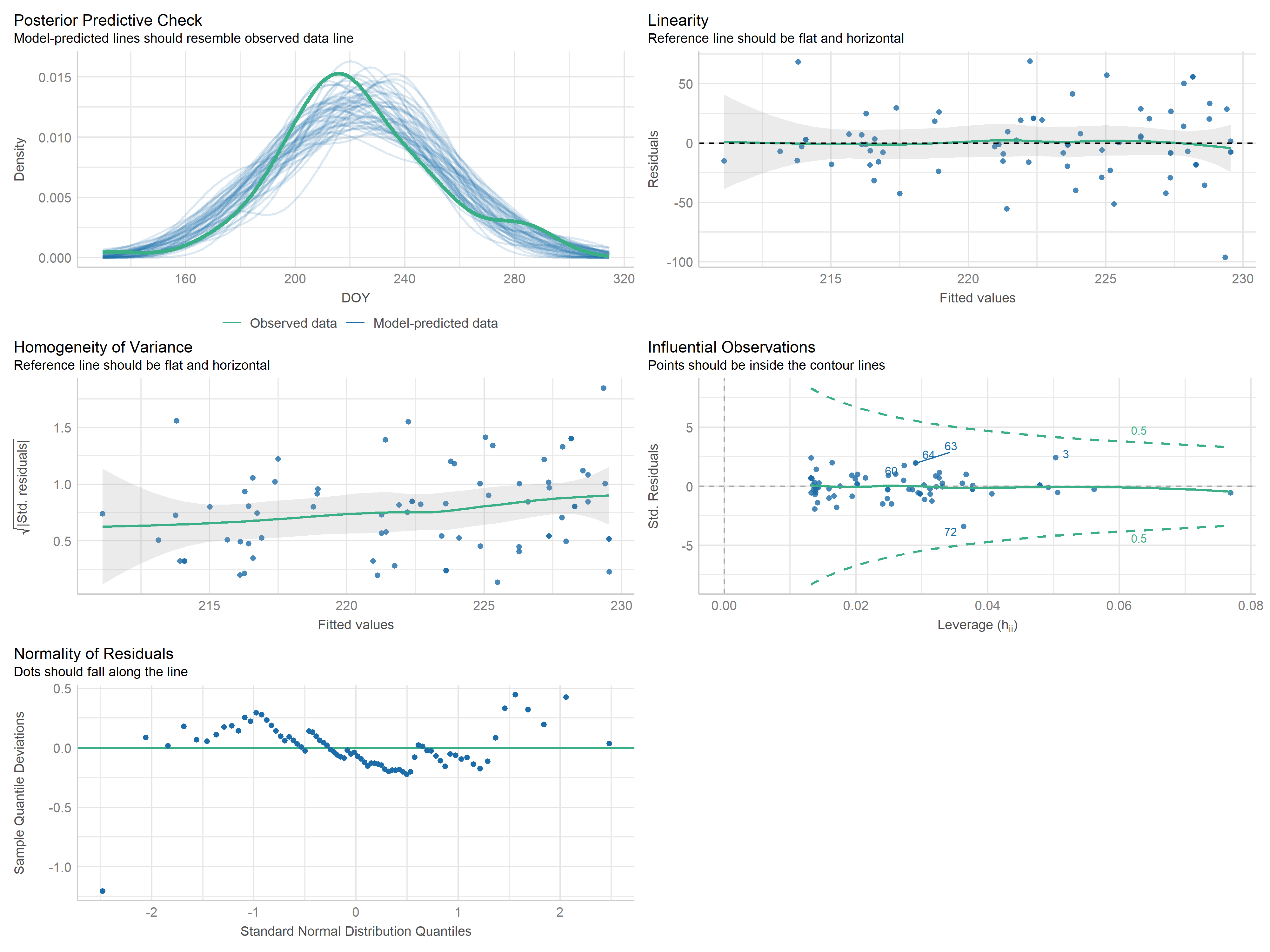

Supplement: Supplementary file 1 [file plants-14-00843-s001.zip › File S2-Species/S2.1-DOYvsYears/1_LM/Plots/Residuals_FBF_Artemisia tridentata.png]
