# Supplementary figures and images for: Phenological Shifts Since 1830 in 29 Native Plant Species of California and Their Responses to Historical Climate Change (part 1 of 2)
Source: Plants (Basel). 2025 Mar 7;14(6):843. doi: 10.3390/plants14060843 (PMC11945038; doi:10.3390/plants14060843)

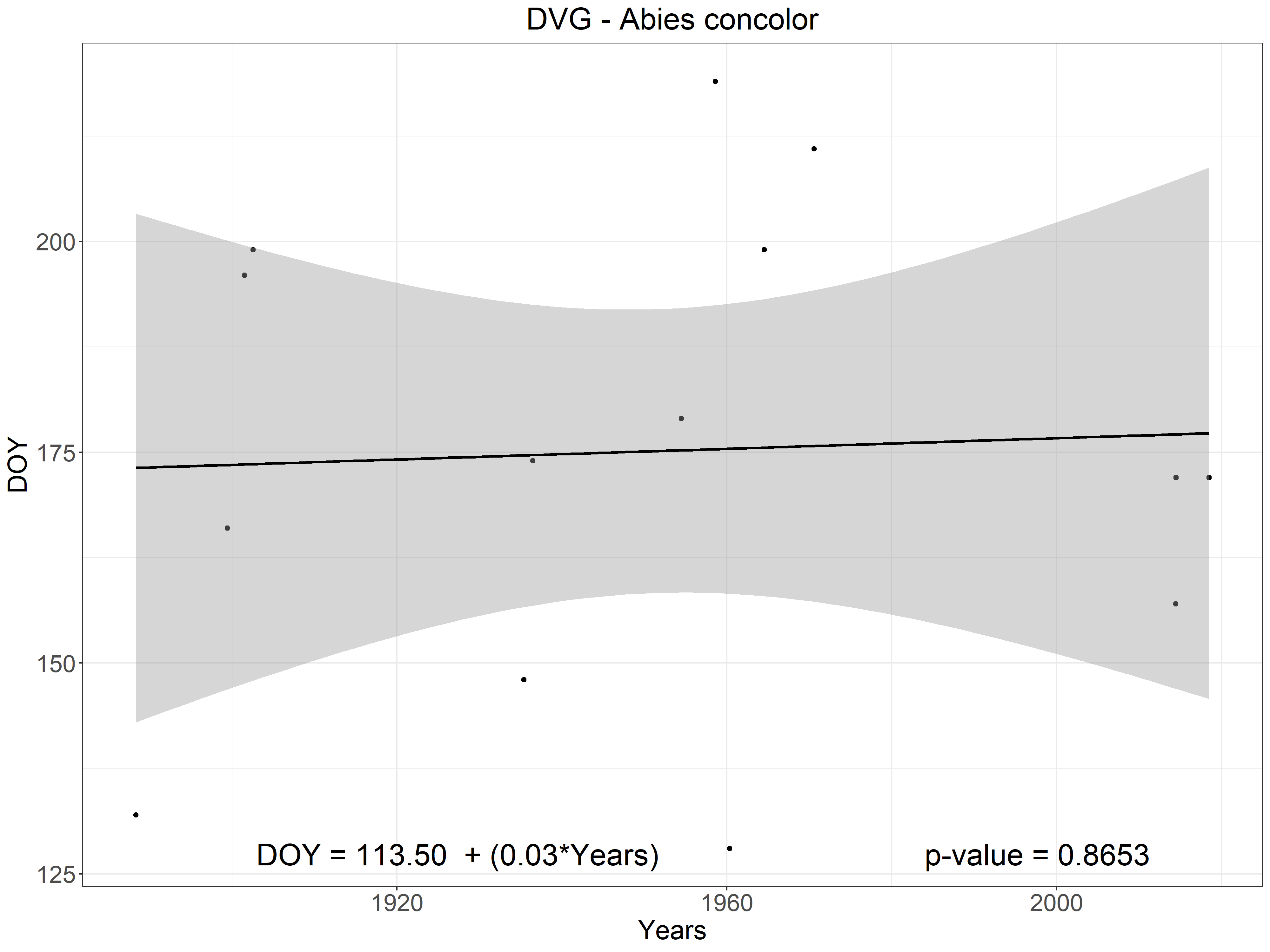

Supplement: Supplementary file 1 [file plants-14-00843-s001.zip › File S2-Species/S2.1-DOYvsYears/1_LM/Plots/DVG_Abies concolor_plot.png]

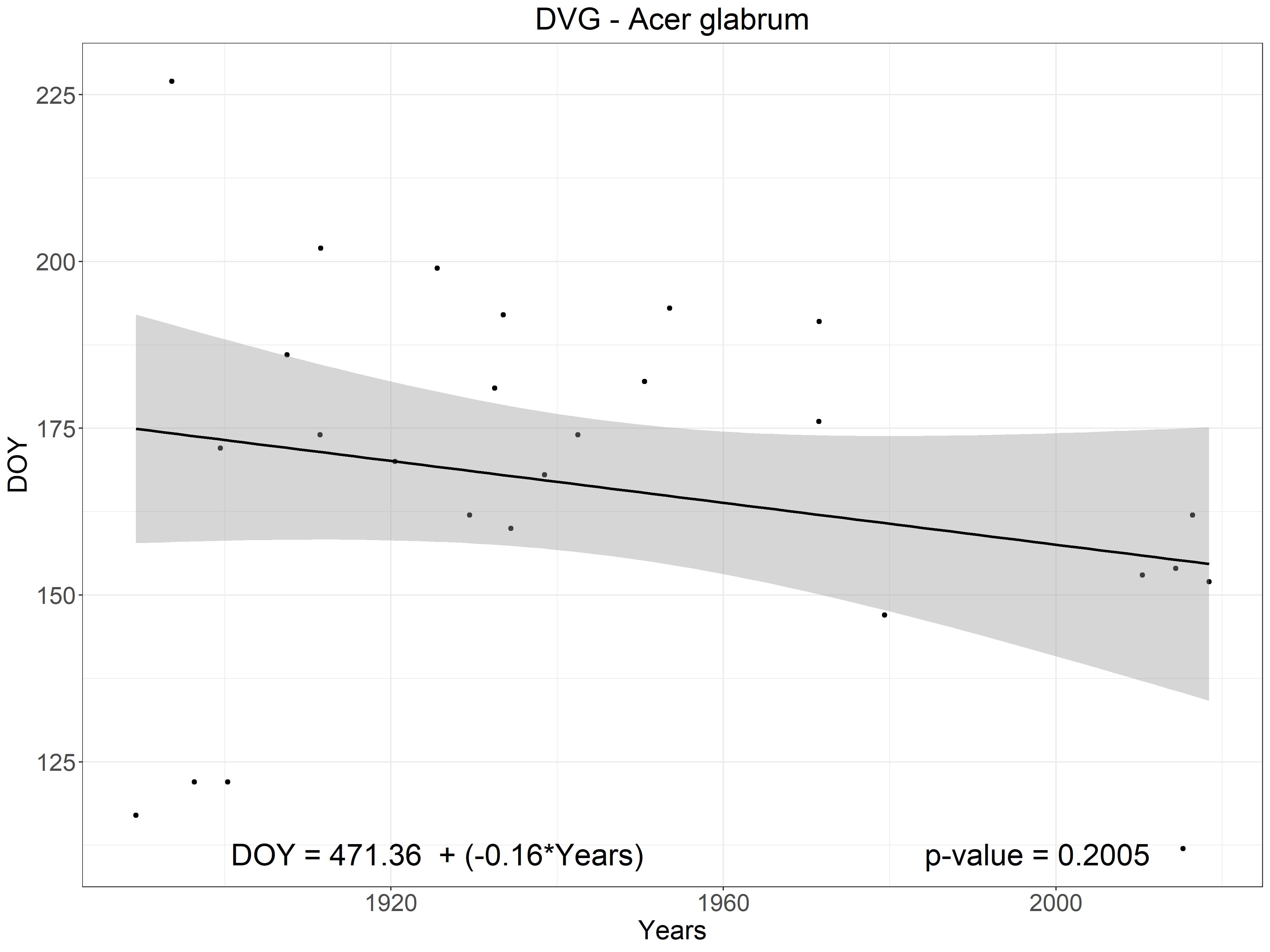

Supplement: Supplementary file 1 [file plants-14-00843-s001.zip › File S2-Species/S2.1-DOYvsYears/1_LM/Plots/DVG_Acer glabrum_plot.png]

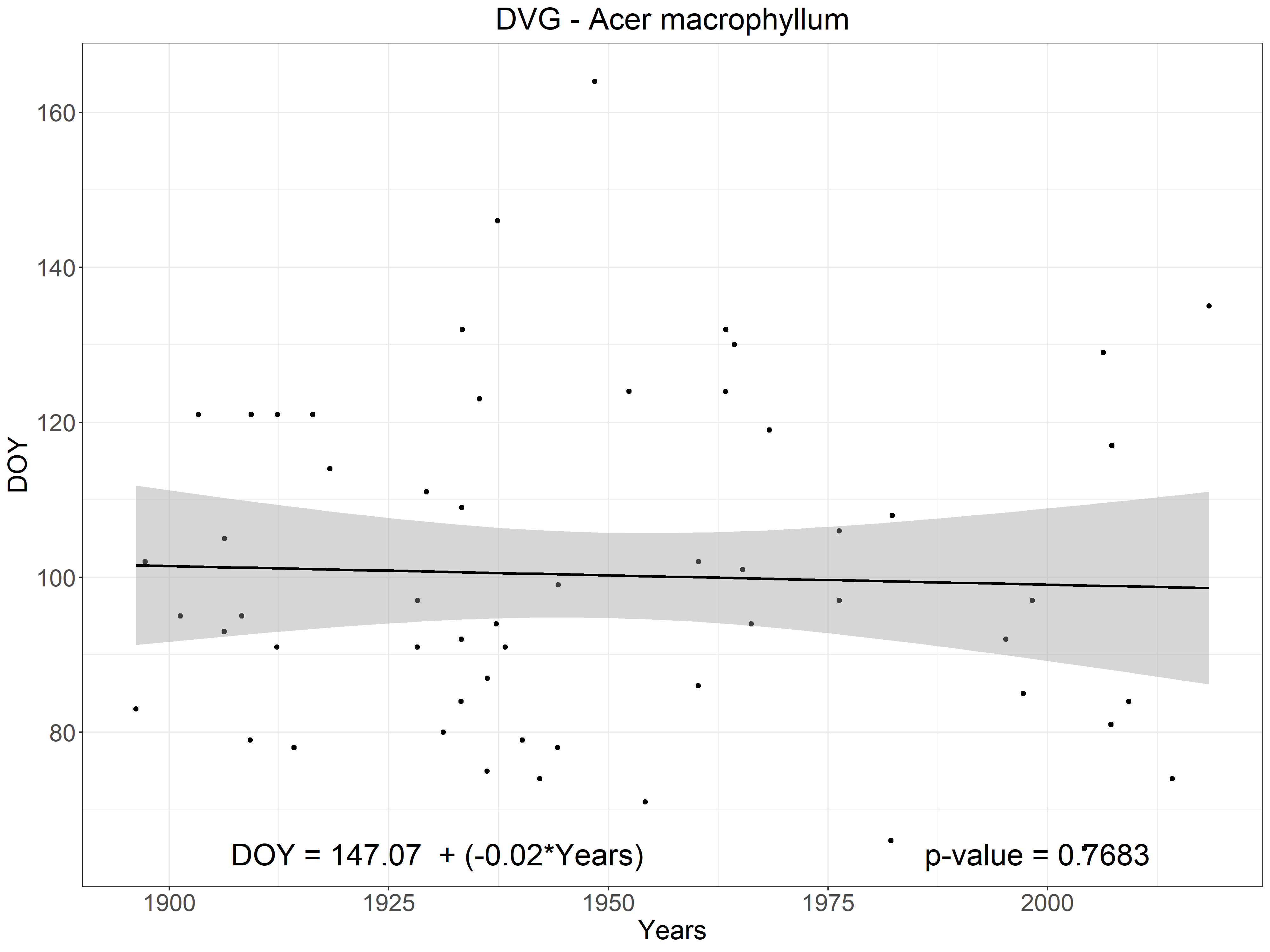

Supplement: Supplementary file 1 [file plants-14-00843-s001.zip › File S2-Species/S2.1-DOYvsYears/1_LM/Plots/DVG_Acer macrophyllum_plot.png]

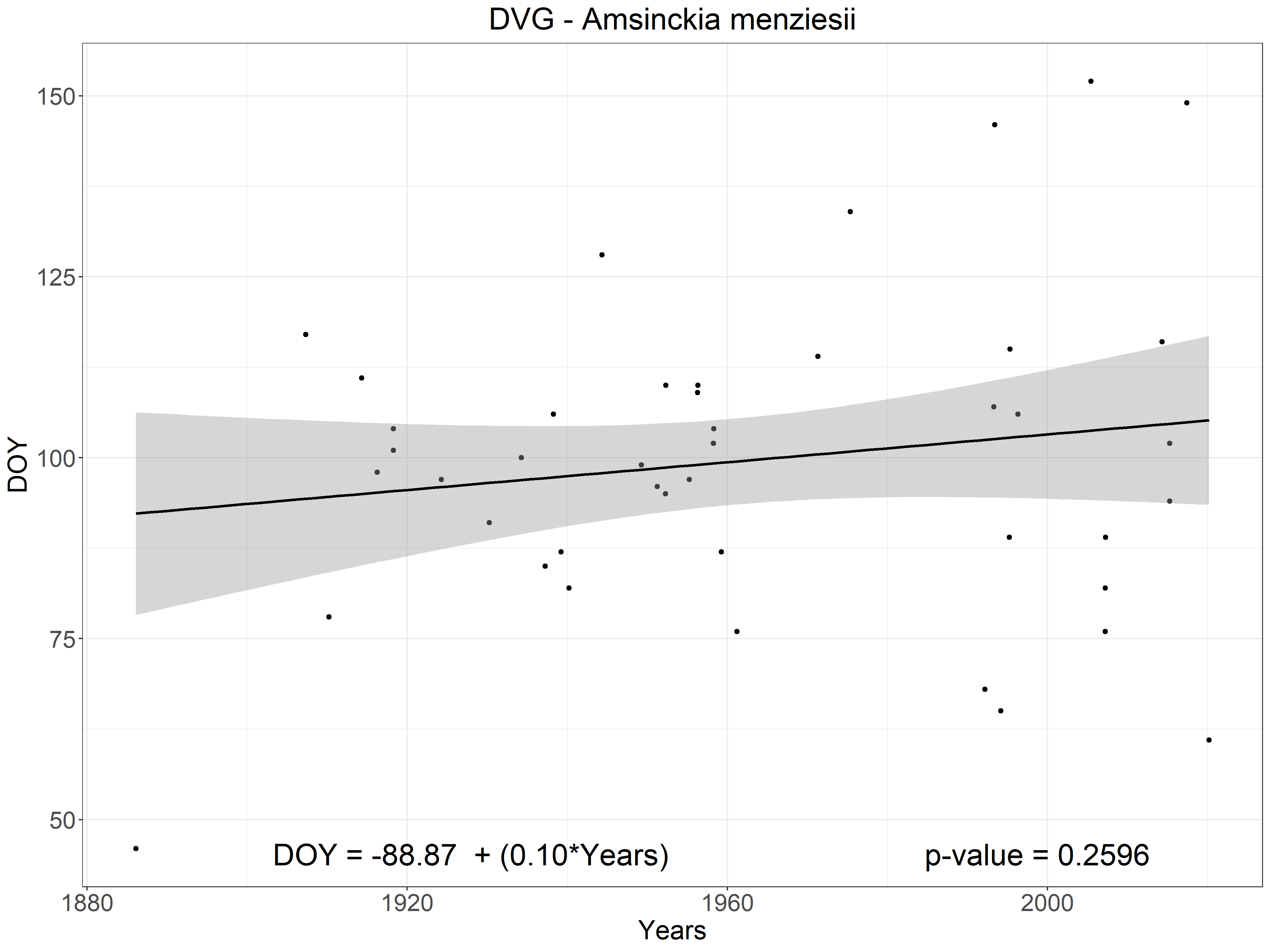

Supplement: Supplementary file 1 [file plants-14-00843-s001.zip › File S2-Species/S2.1-DOYvsYears/1_LM/Plots/DVG_Amsinckia menziesii_plot.png]

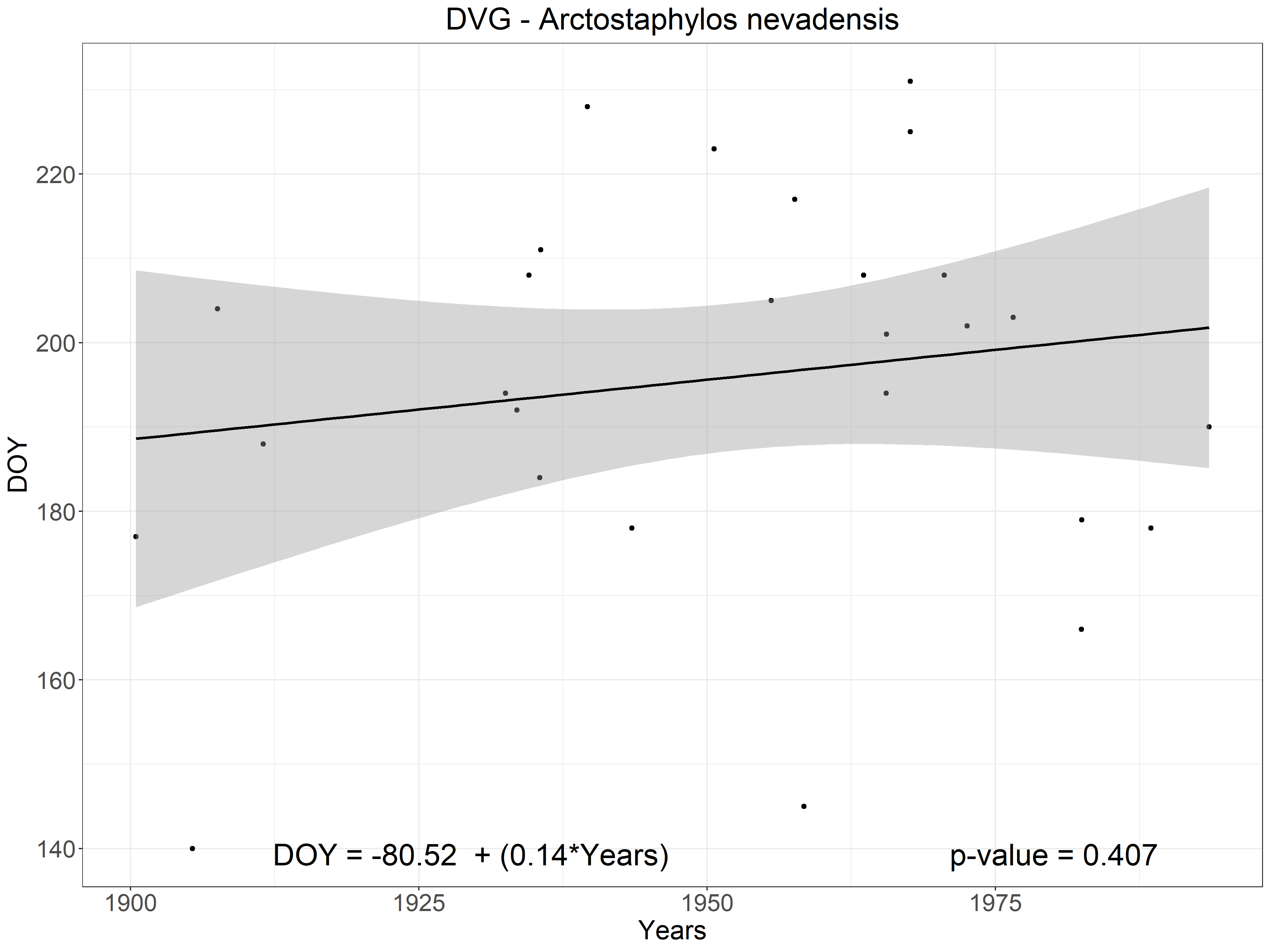

Supplement: Supplementary file 1 [file plants-14-00843-s001.zip › File S2-Species/S2.1-DOYvsYears/1_LM/Plots/DVG_Arctostaphylos nevadensis_plot.png]

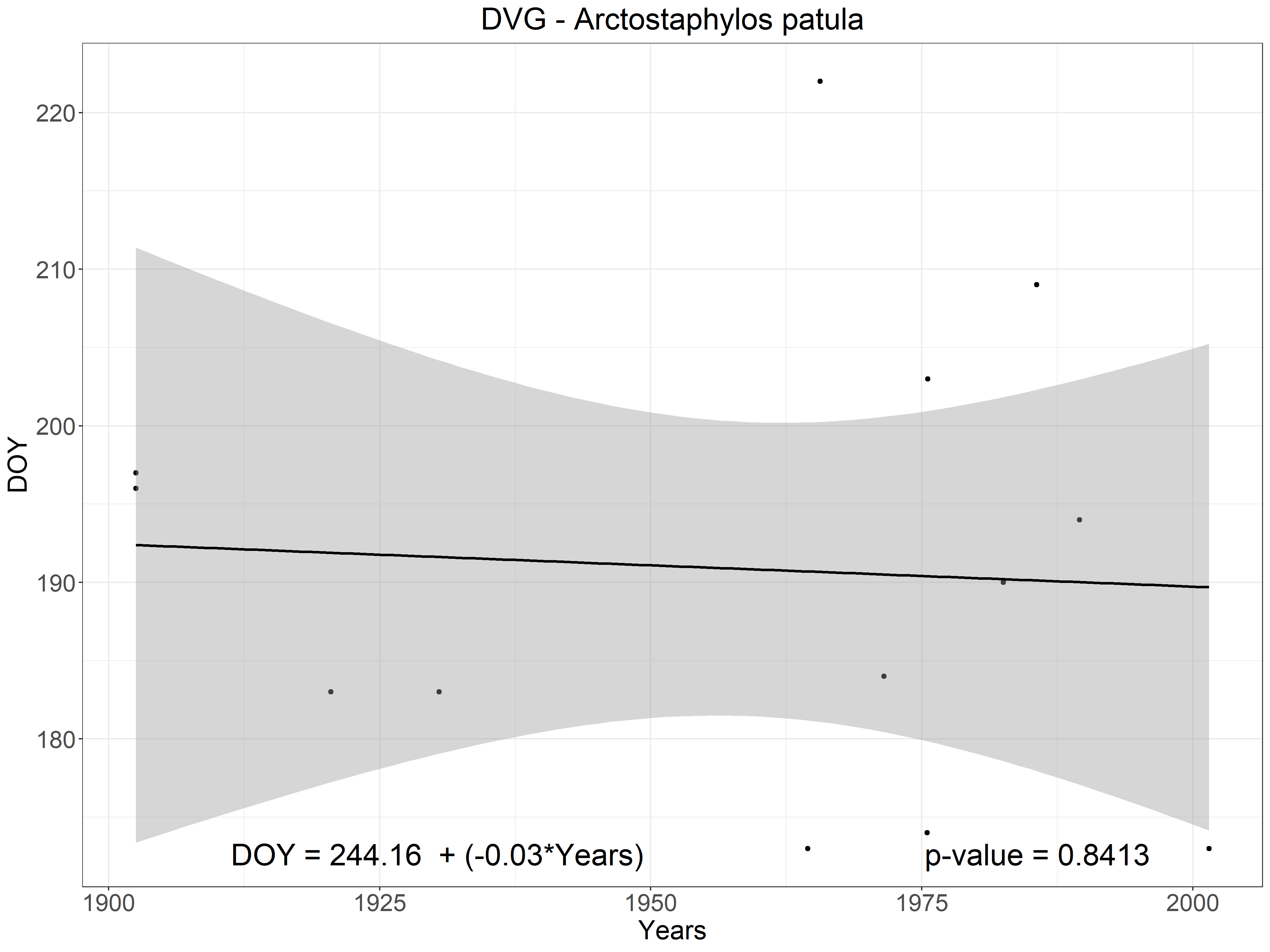

Supplement: Supplementary file 1 [file plants-14-00843-s001.zip › File S2-Species/S2.1-DOYvsYears/1_LM/Plots/DVG_Arctostaphylos patula_plot.png]

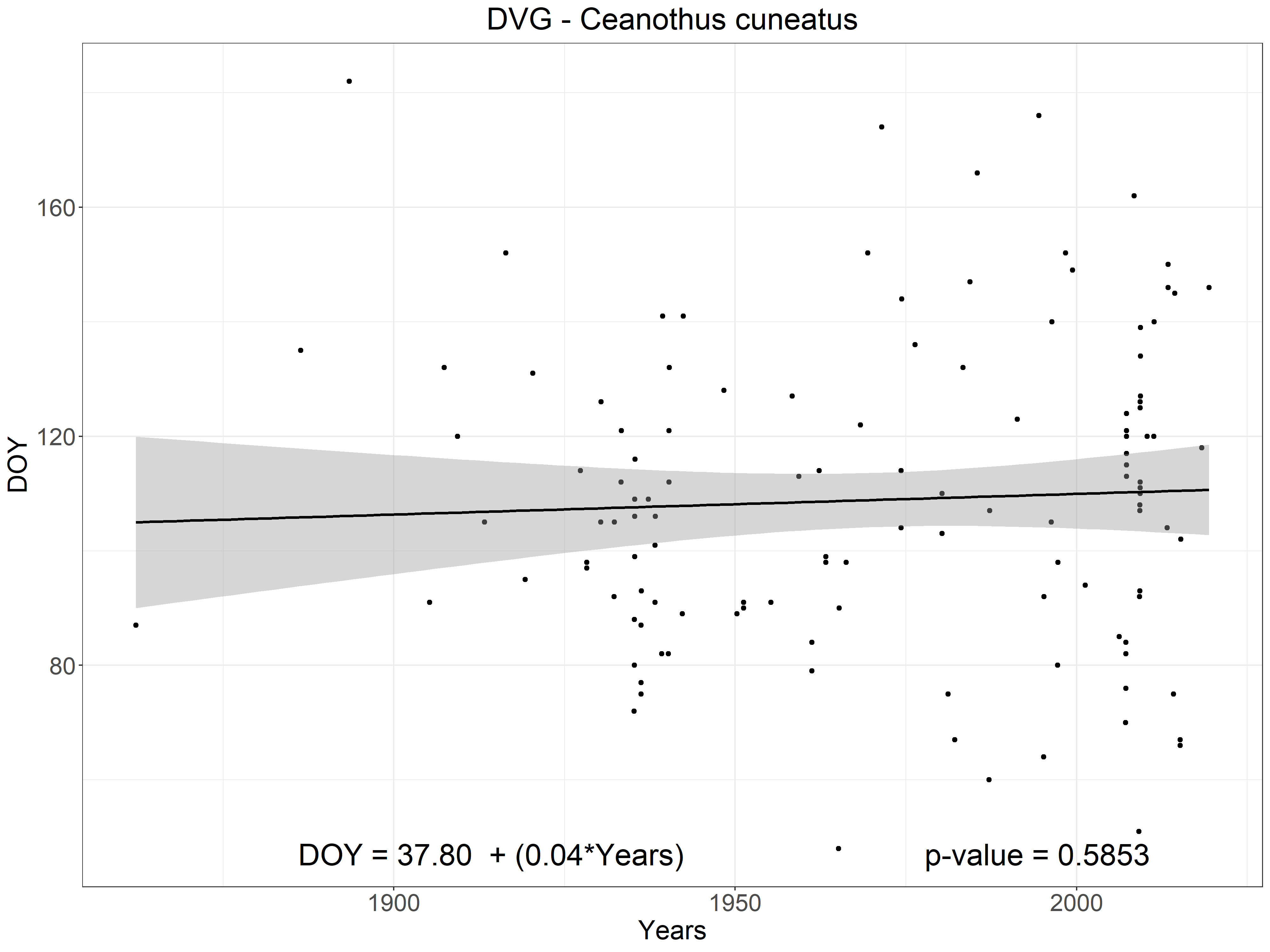

Supplement: Supplementary file 1 [file plants-14-00843-s001.zip › File S2-Species/S2.1-DOYvsYears/1_LM/Plots/DVG_Ceanothus cuneatus_plot.png]

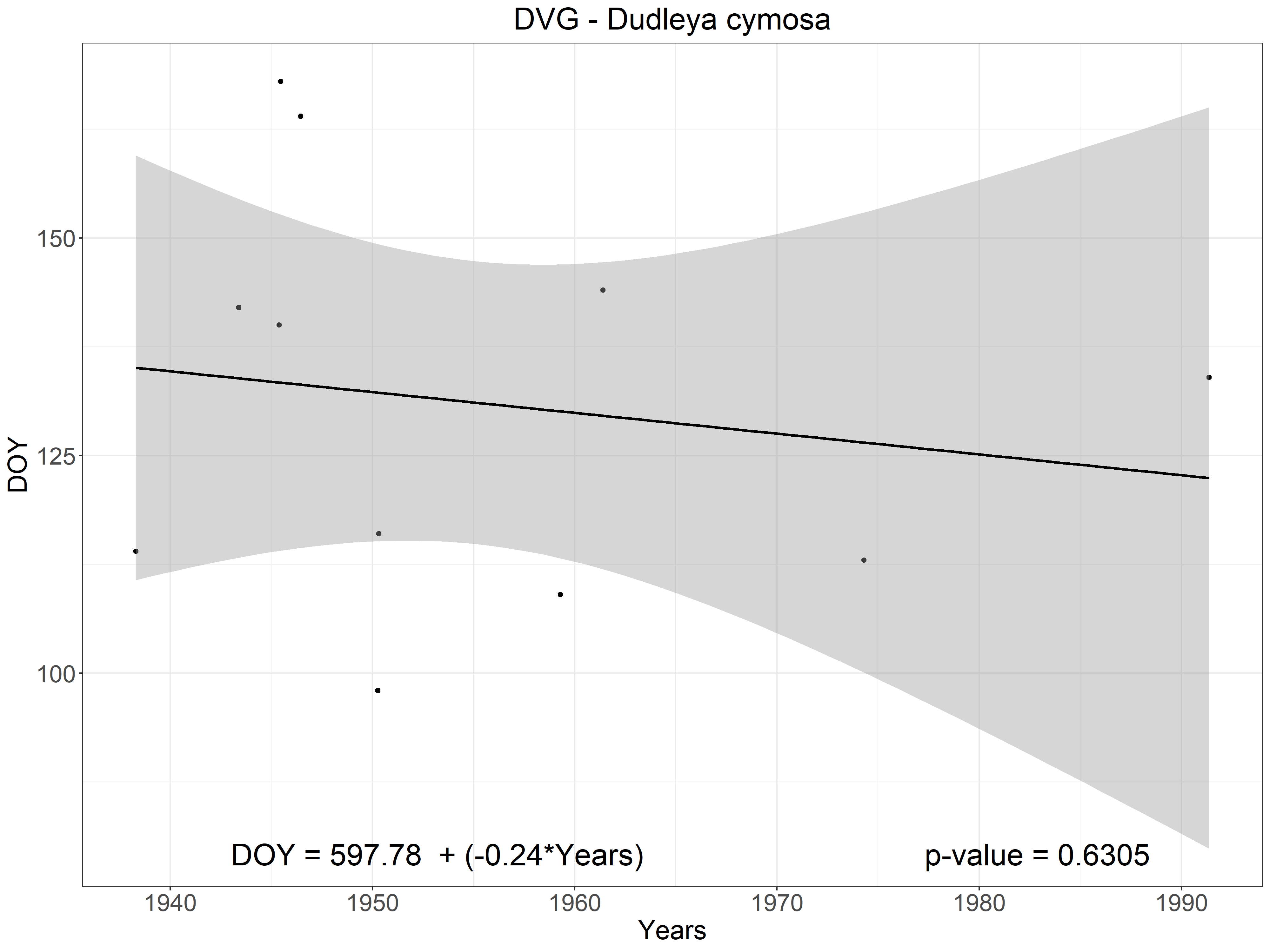

Supplement: Supplementary file 1 [file plants-14-00843-s001.zip › File S2-Species/S2.1-DOYvsYears/1_LM/Plots/DVG_Dudleya cymosa_plot.png]

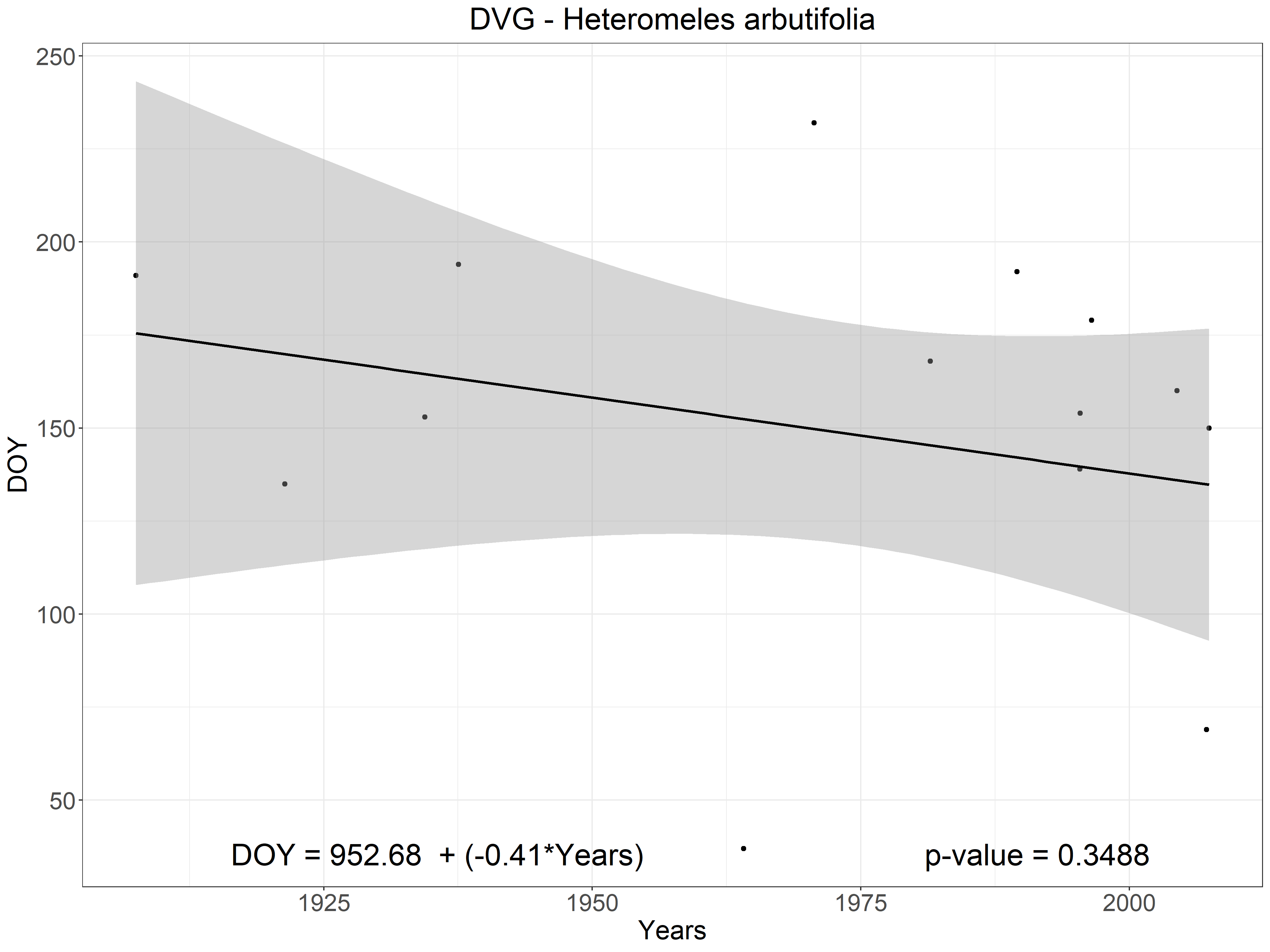

Supplement: Supplementary file 1 [file plants-14-00843-s001.zip › File S2-Species/S2.1-DOYvsYears/1_LM/Plots/DVG_Heteromeles arbutifolia_plot.png]

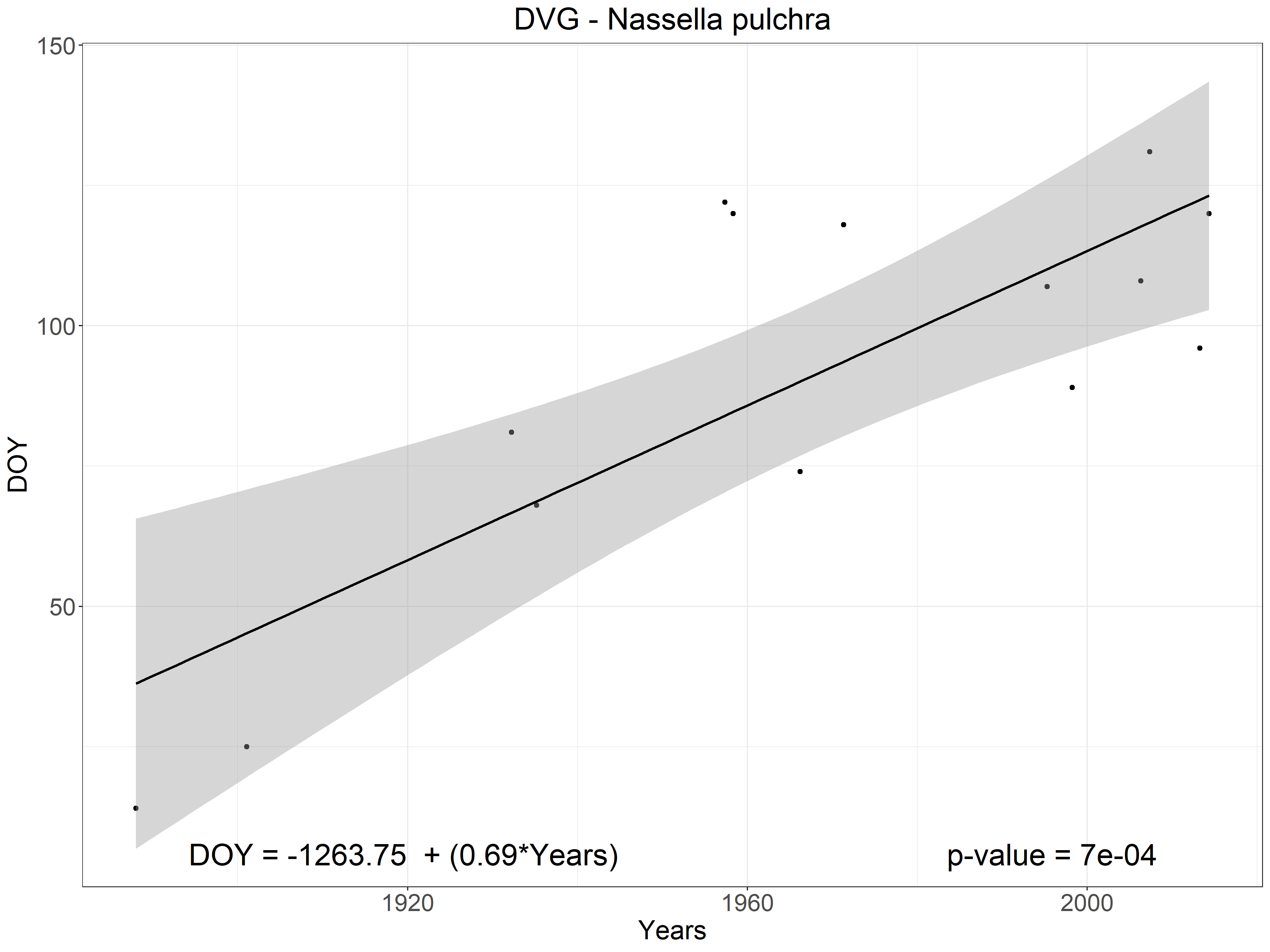

Supplement: Supplementary file 1 [file plants-14-00843-s001.zip › File S2-Species/S2.1-DOYvsYears/1_LM/Plots/DVG_Nassella pulchra_plot.png]

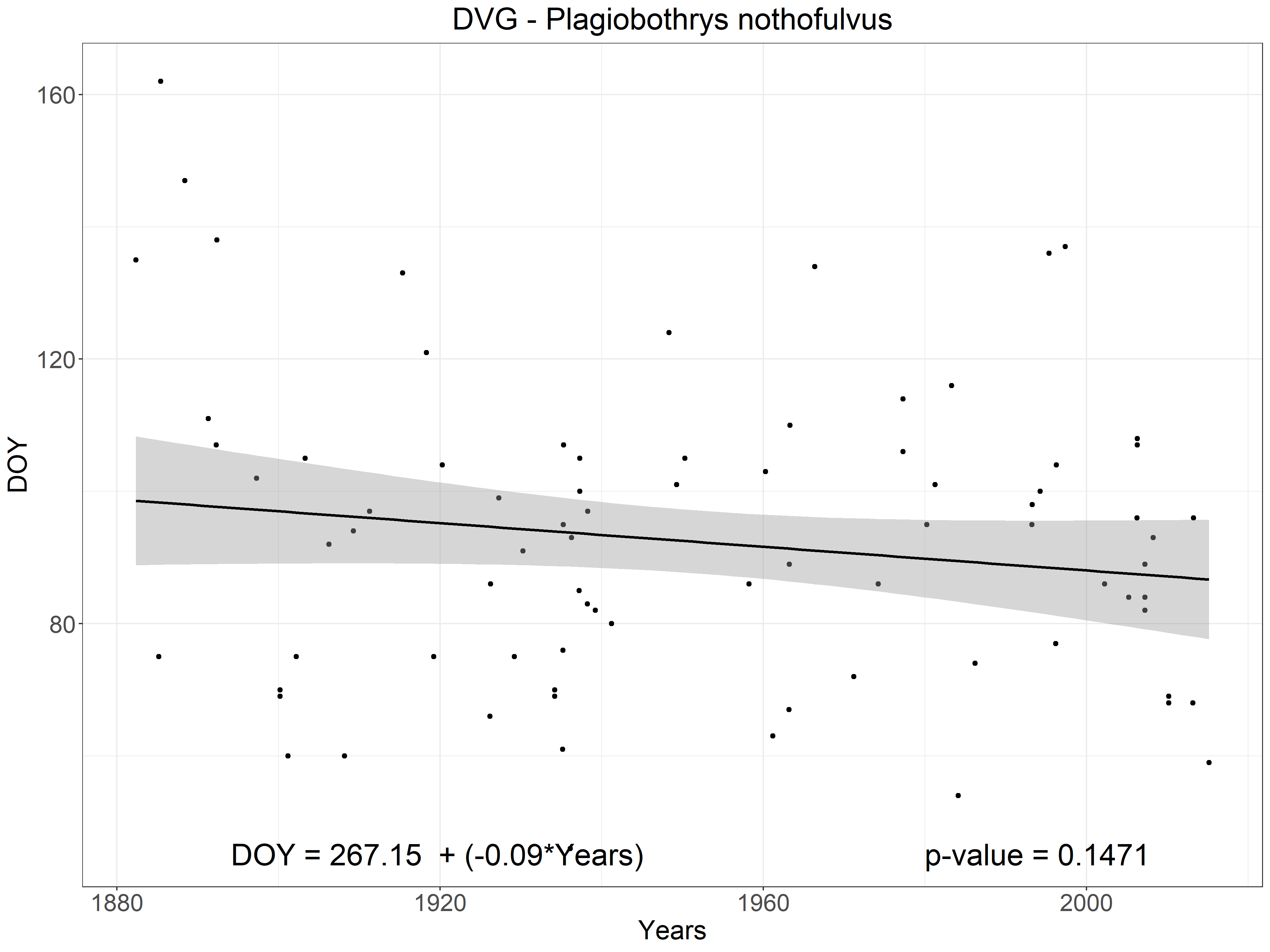

Supplement: Supplementary file 1 [file plants-14-00843-s001.zip › File S2-Species/S2.1-DOYvsYears/1_LM/Plots/DVG_Plagiobothrys nothofulvus_plot.png]

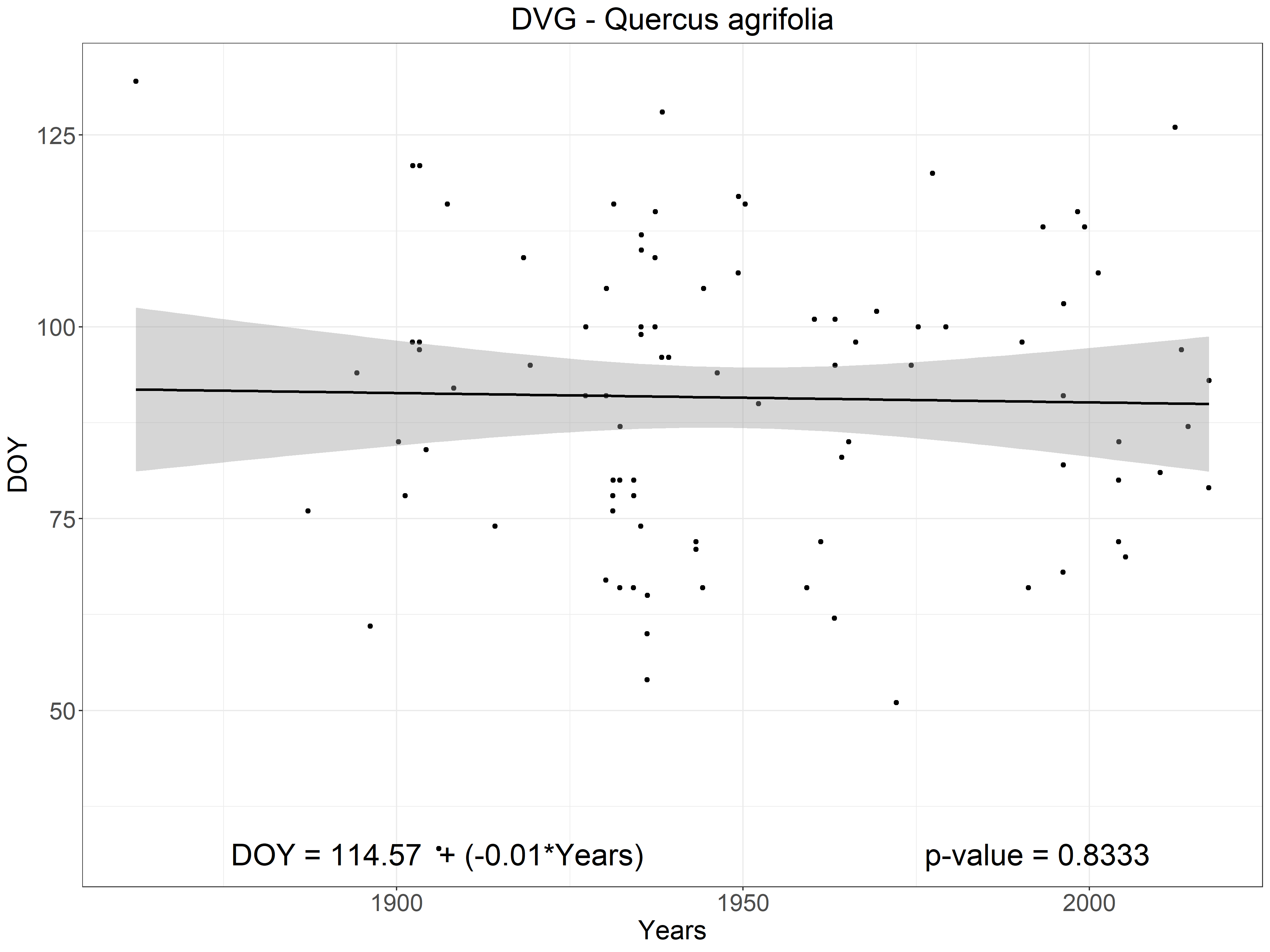

Supplement: Supplementary file 1 [file plants-14-00843-s001.zip › File S2-Species/S2.1-DOYvsYears/1_LM/Plots/DVG_Quercus agrifolia_plot.png]

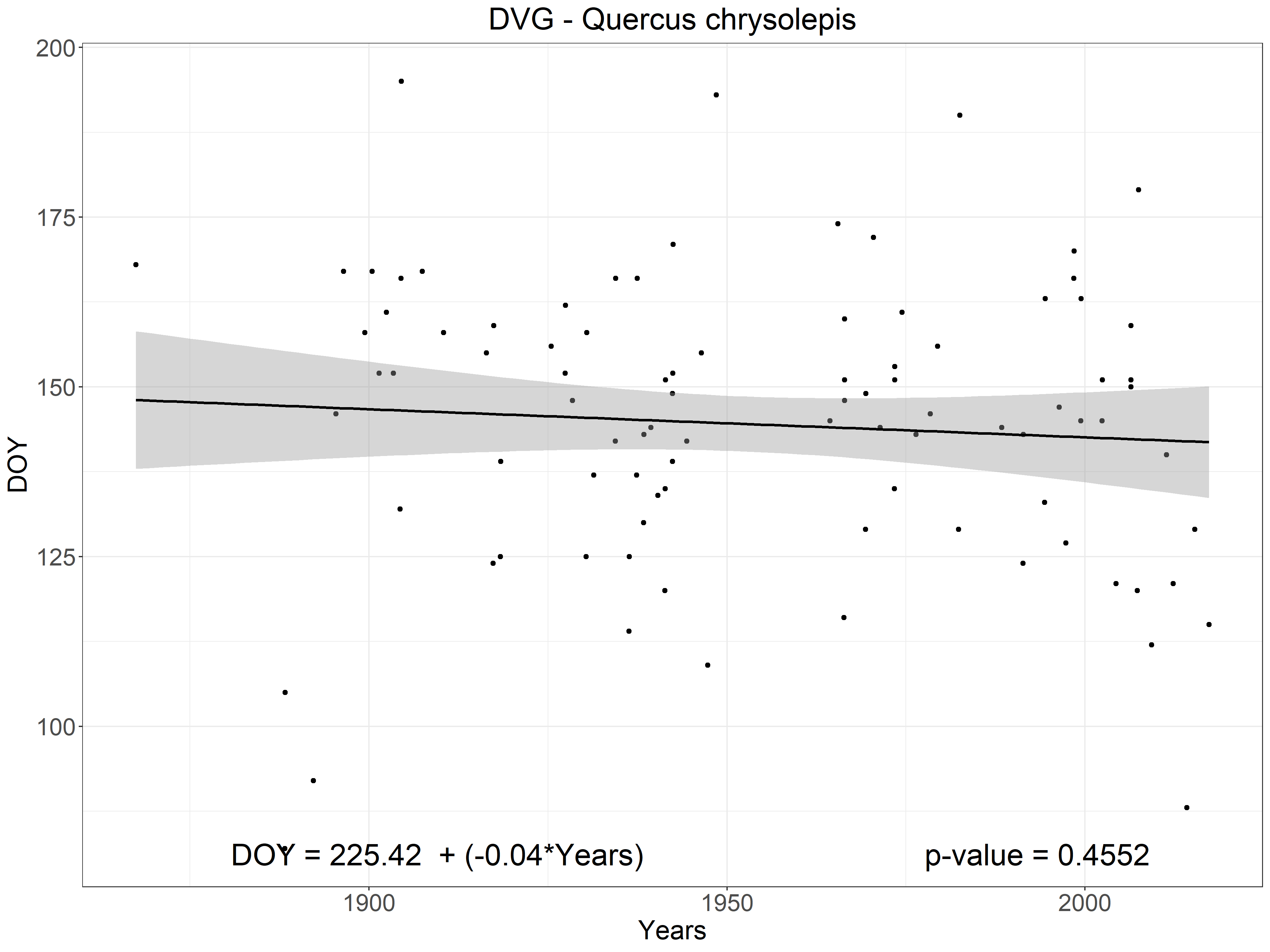

Supplement: Supplementary file 1 [file plants-14-00843-s001.zip › File S2-Species/S2.1-DOYvsYears/1_LM/Plots/DVG_Quercus chrysolepis_plot.png]

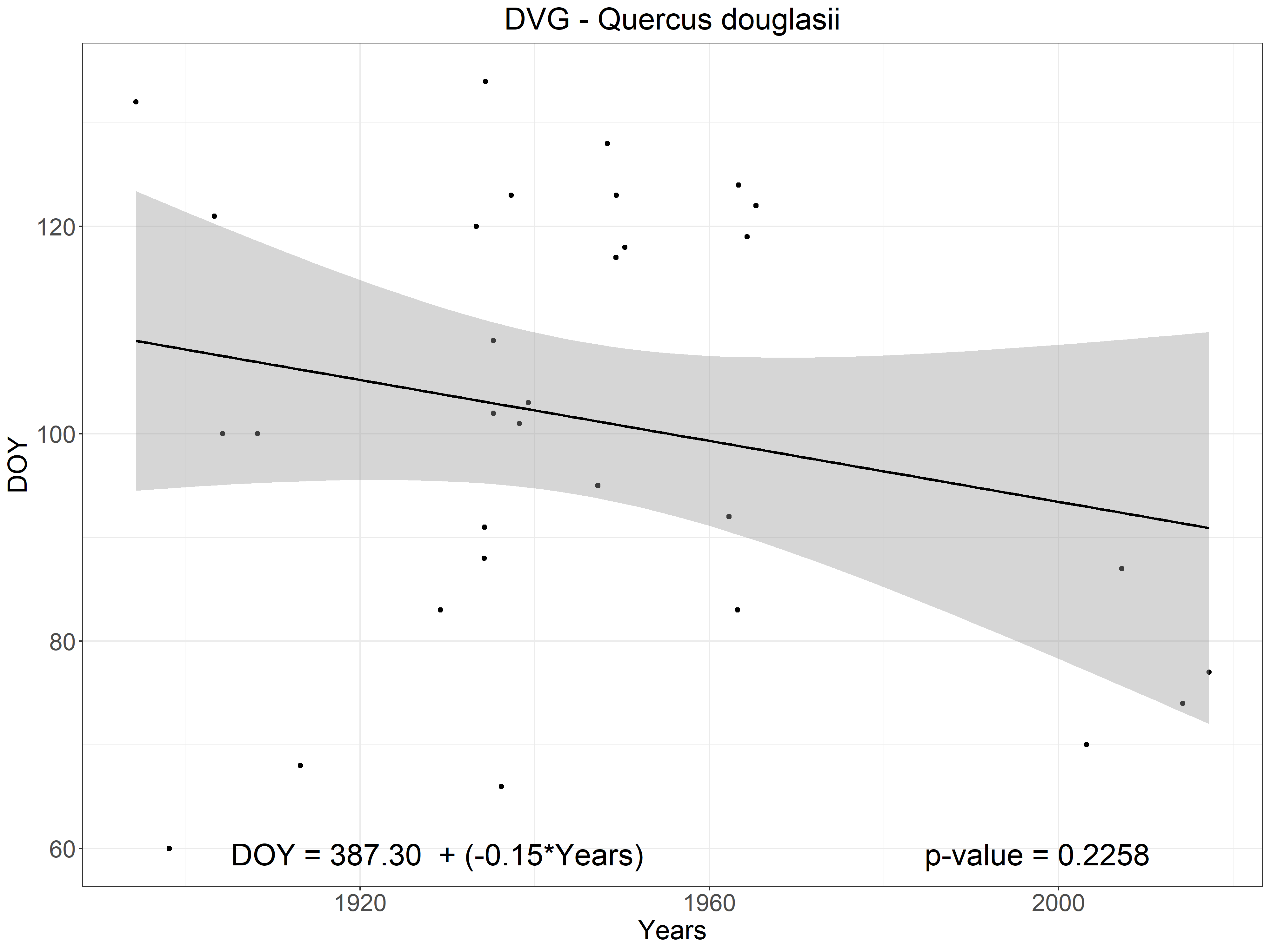

Supplement: Supplementary file 1 [file plants-14-00843-s001.zip › File S2-Species/S2.1-DOYvsYears/1_LM/Plots/DVG_Quercus douglasii_plot.png]

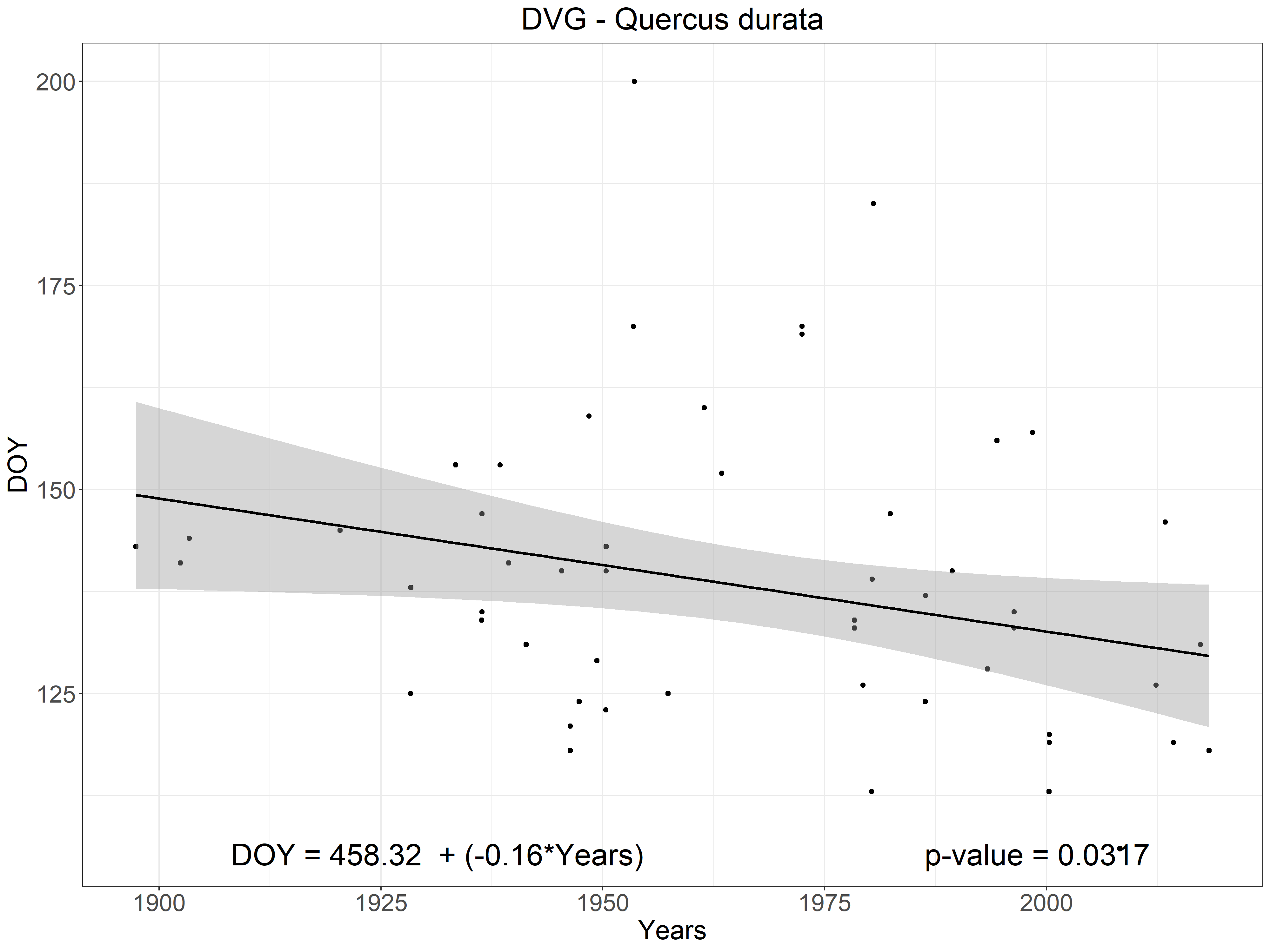

Supplement: Supplementary file 1 [file plants-14-00843-s001.zip › File S2-Species/S2.1-DOYvsYears/1_LM/Plots/DVG_Quercus durata_plot.png]

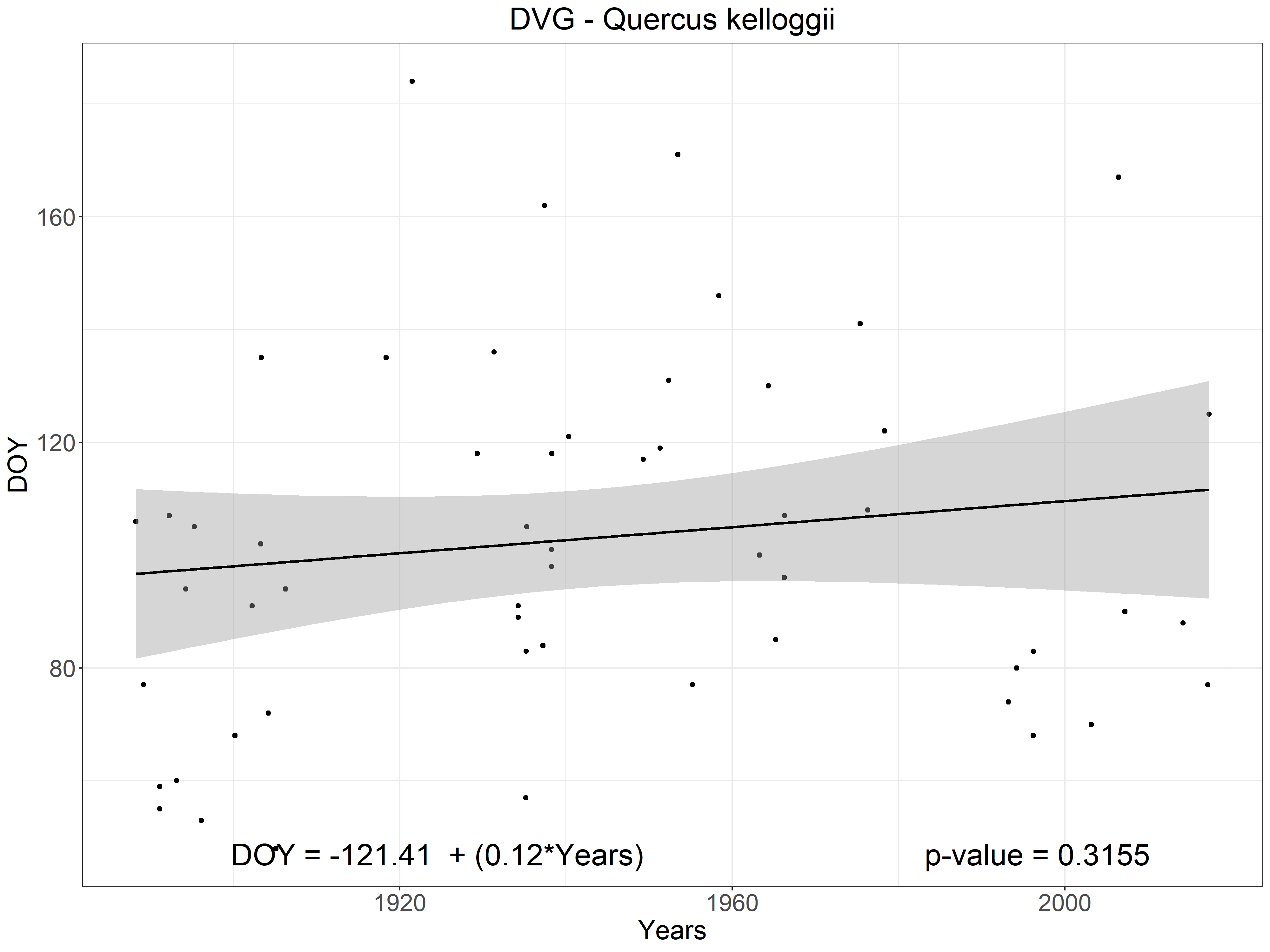

Supplement: Supplementary file 1 [file plants-14-00843-s001.zip › File S2-Species/S2.1-DOYvsYears/1_LM/Plots/DVG_Quercus kelloggii_plot.png]

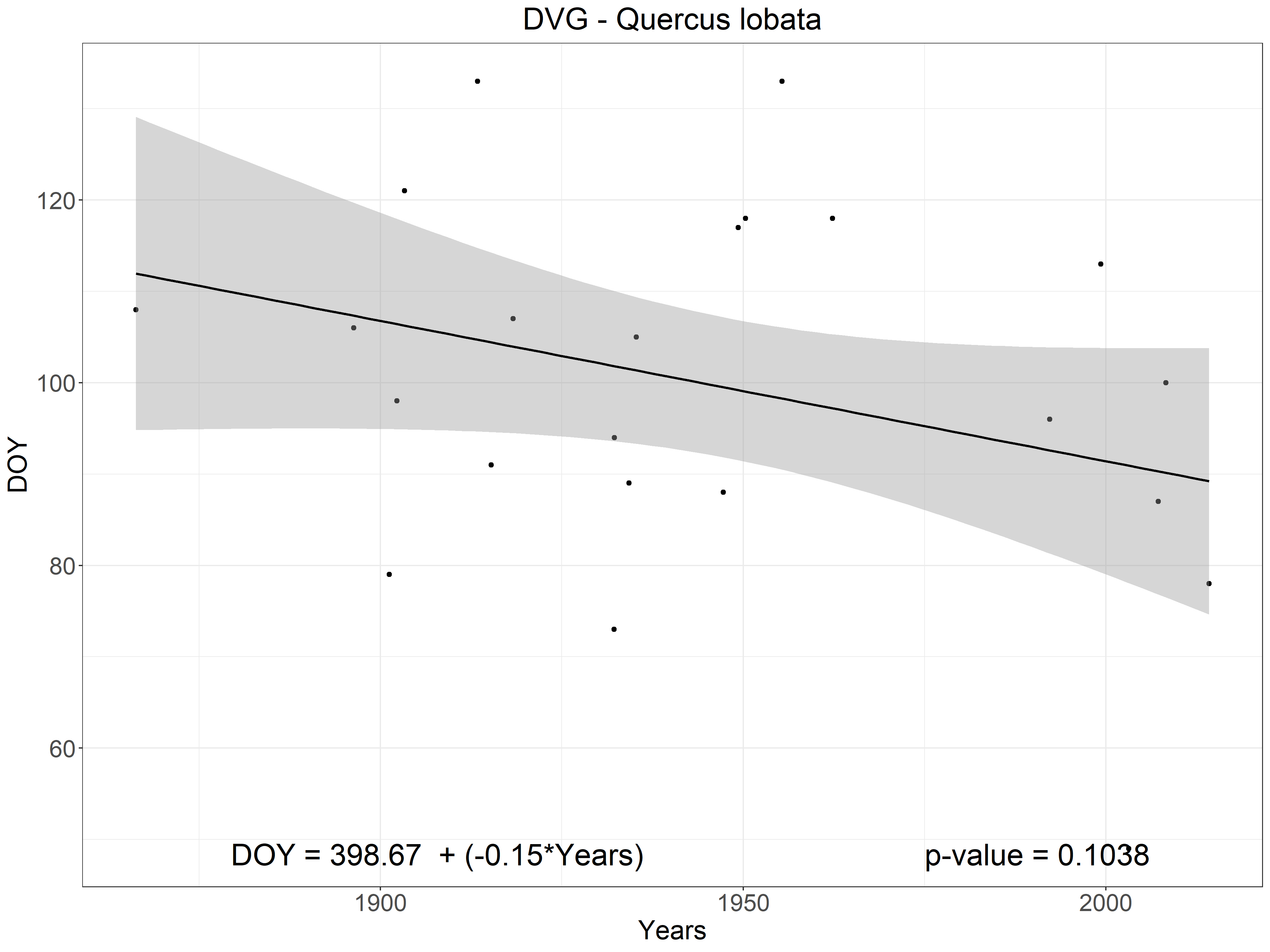

Supplement: Supplementary file 1 [file plants-14-00843-s001.zip › File S2-Species/S2.1-DOYvsYears/1_LM/Plots/DVG_Quercus lobata_plot.png]

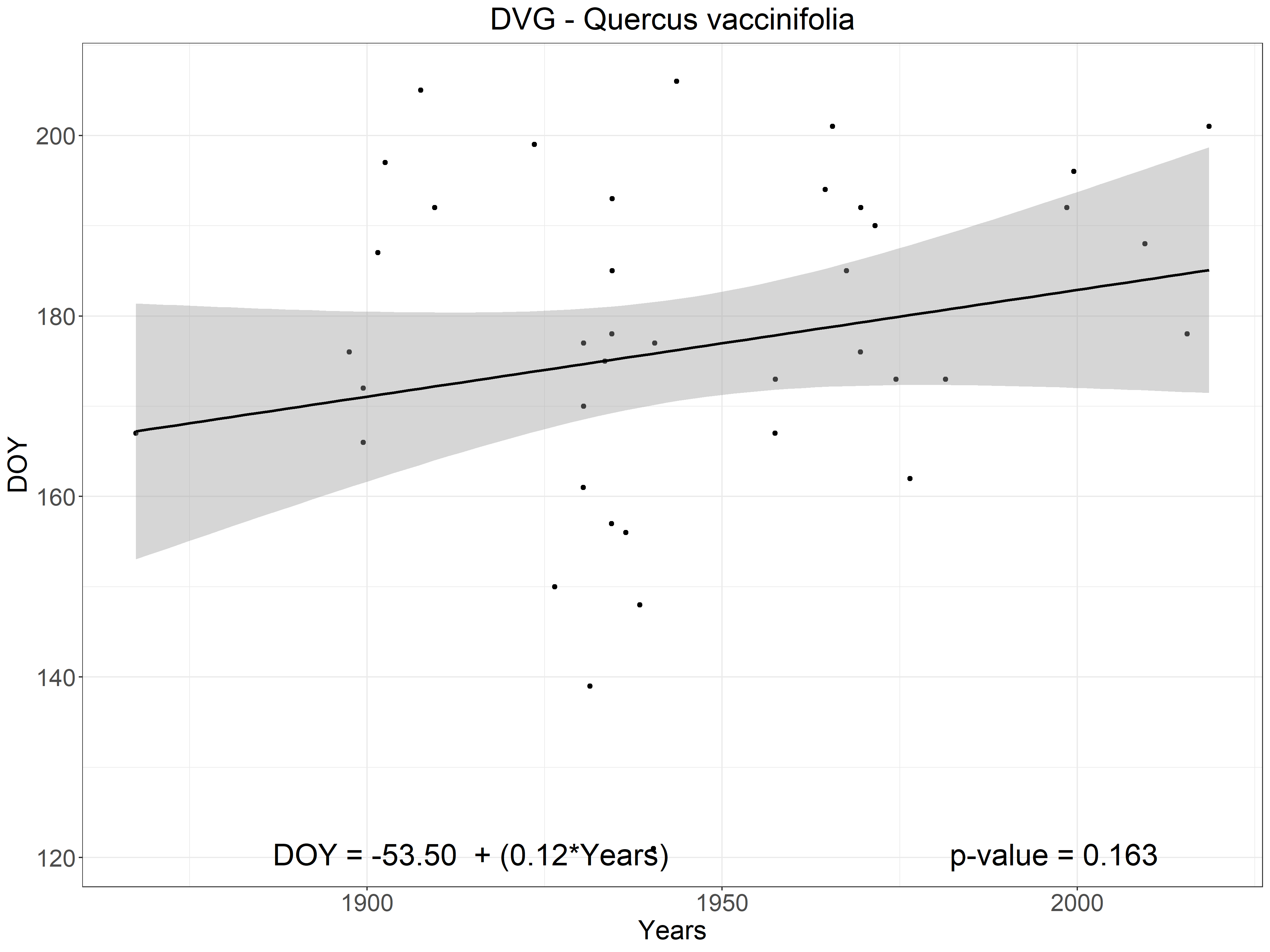

Supplement: Supplementary file 1 [file plants-14-00843-s001.zip › File S2-Species/S2.1-DOYvsYears/1_LM/Plots/DVG_Quercus vaccinifolia_plot.png]

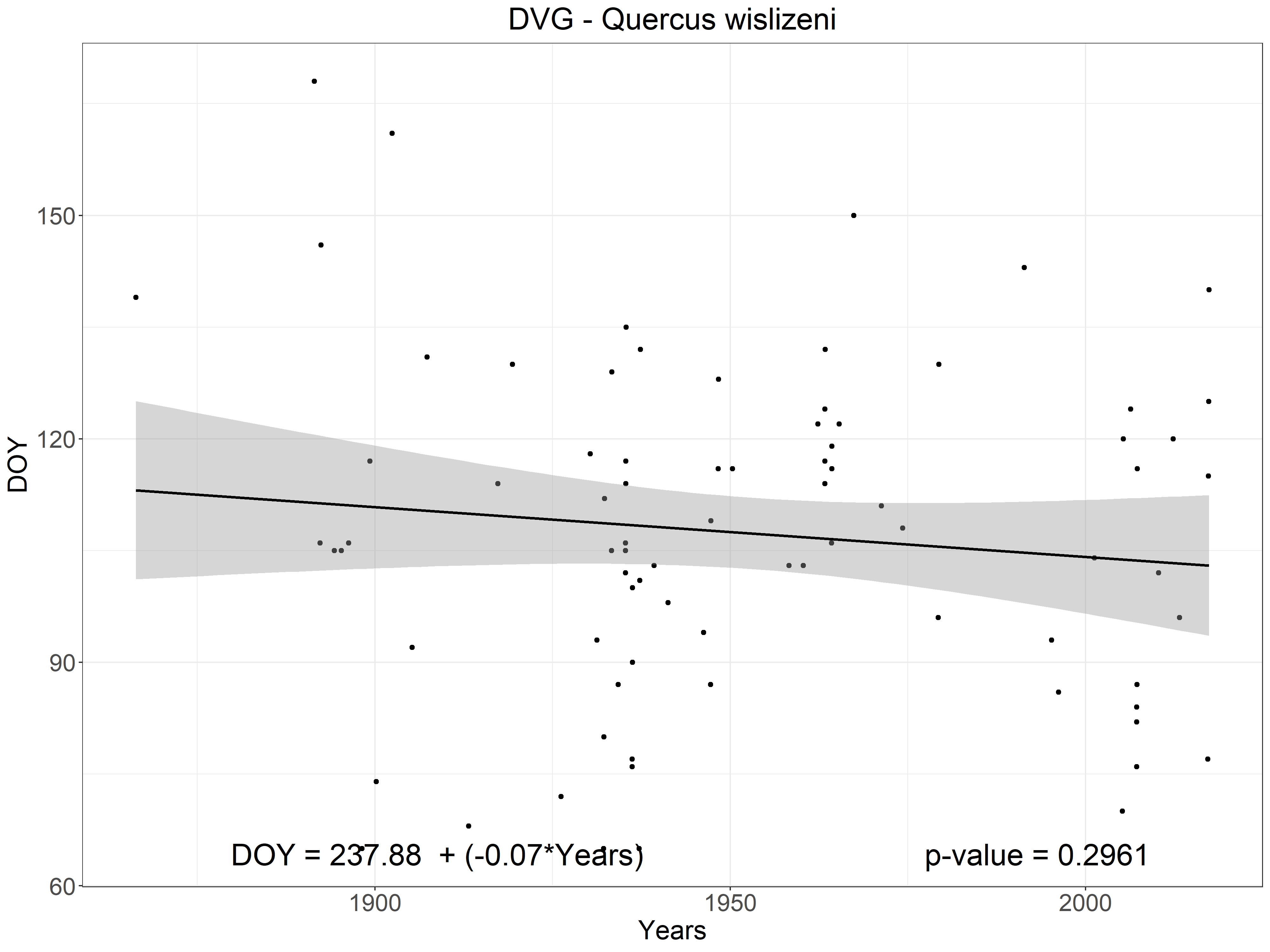

Supplement: Supplementary file 1 [file plants-14-00843-s001.zip › File S2-Species/S2.1-DOYvsYears/1_LM/Plots/DVG_Quercus wislizeni_plot.png]

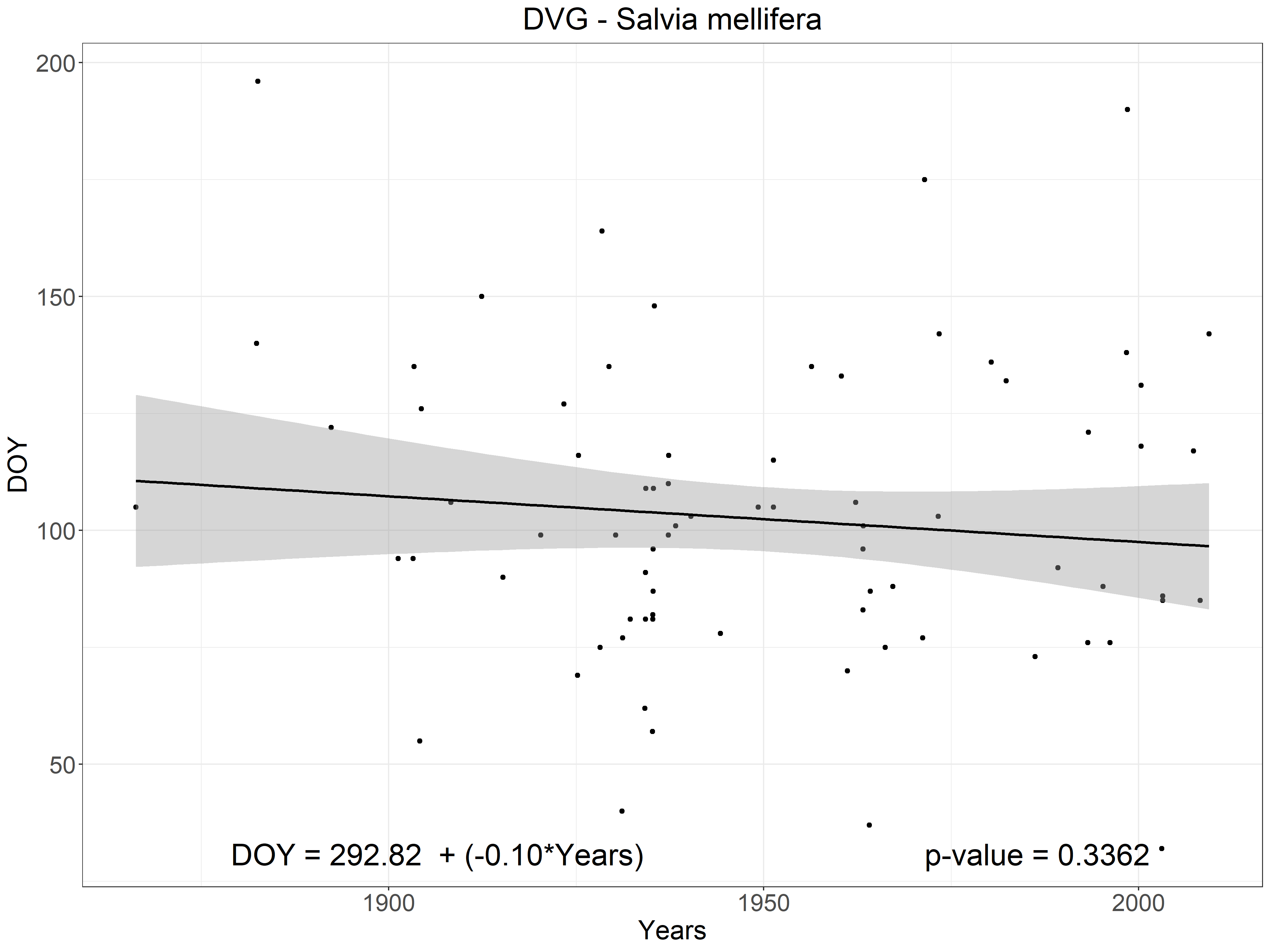

Supplement: Supplementary file 1 [file plants-14-00843-s001.zip › File S2-Species/S2.1-DOYvsYears/1_LM/Plots/DVG_Salvia mellifera_plot.png]

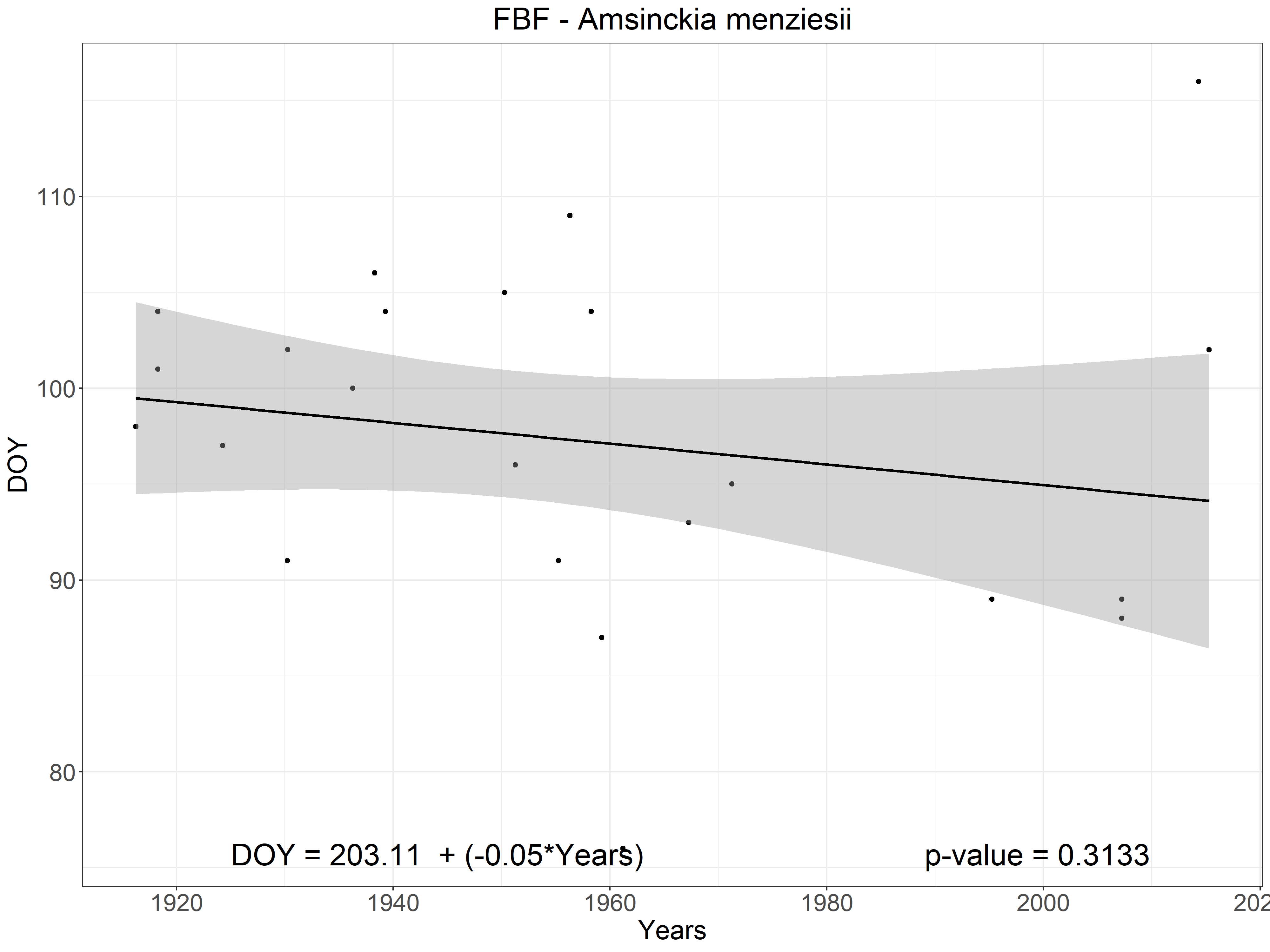

Supplement: Supplementary file 1 [file plants-14-00843-s001.zip › File S2-Species/S2.1-DOYvsYears/1_LM/Plots/FBF_Amsinckia menziesii_plot.png]

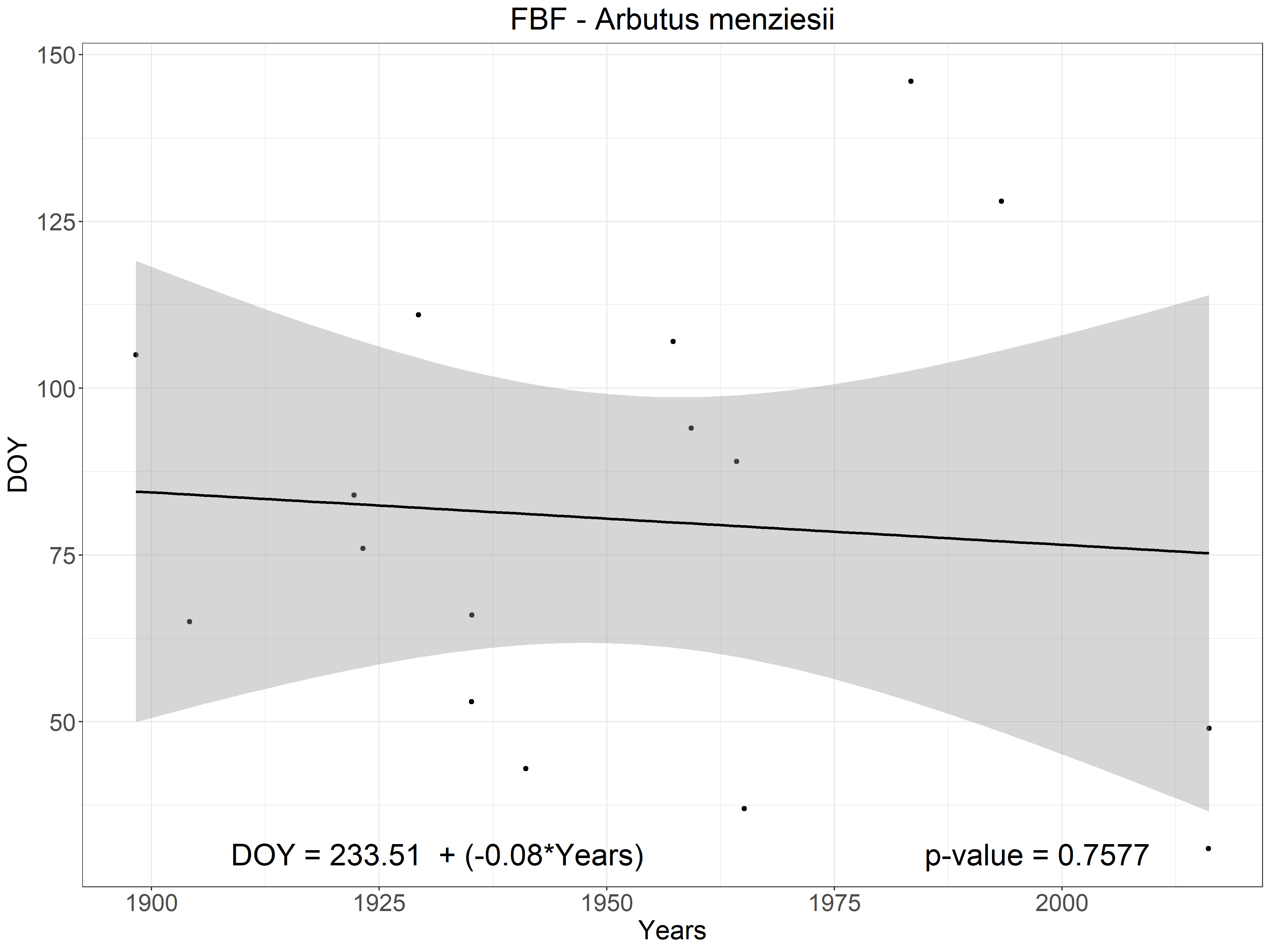

Supplement: Supplementary file 1 [file plants-14-00843-s001.zip › File S2-Species/S2.1-DOYvsYears/1_LM/Plots/FBF_Arbutus menziesii_plot.png]

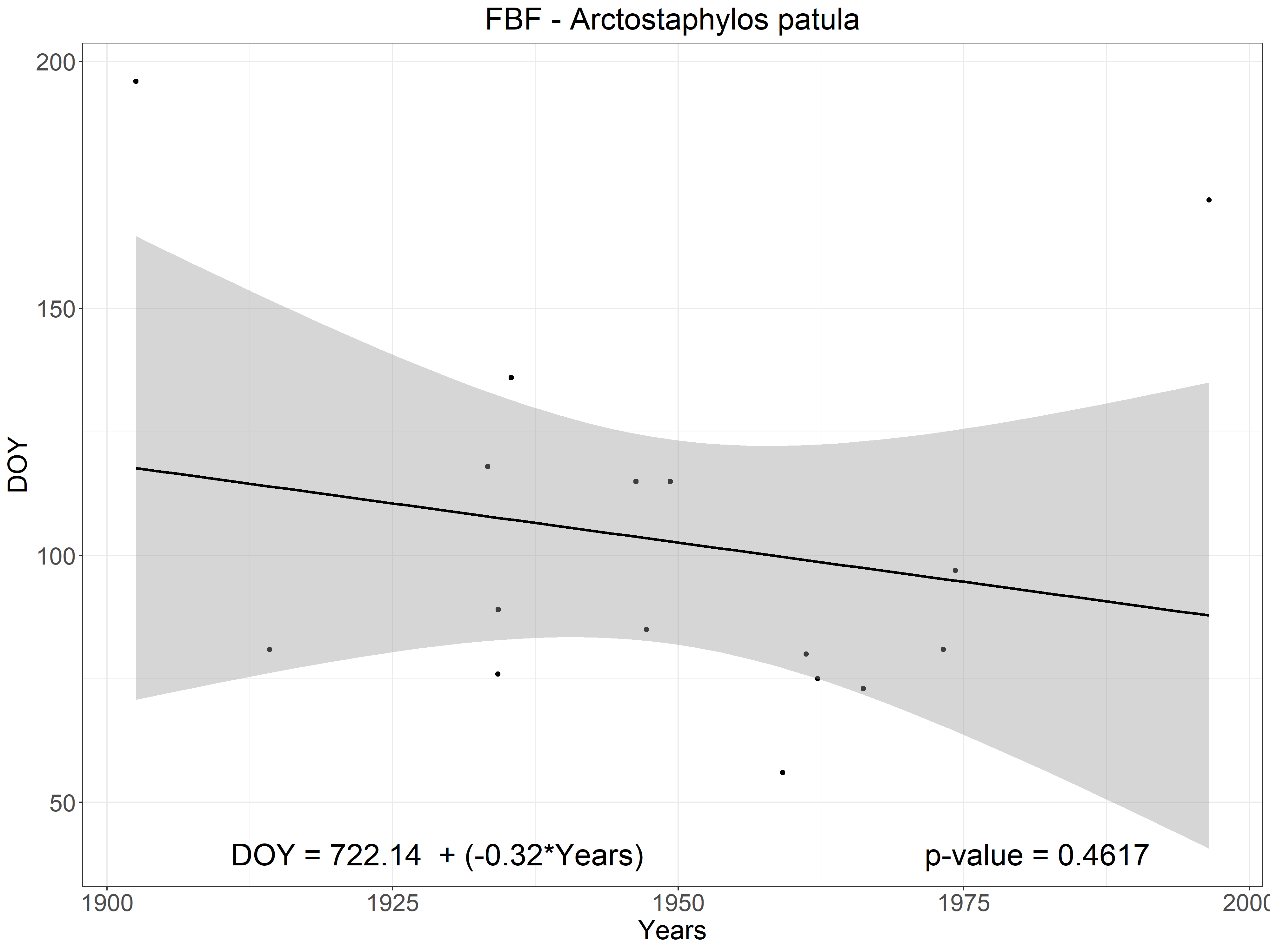

Supplement: Supplementary file 1 [file plants-14-00843-s001.zip › File S2-Species/S2.1-DOYvsYears/1_LM/Plots/FBF_Arctostaphylos patula_plot.png]

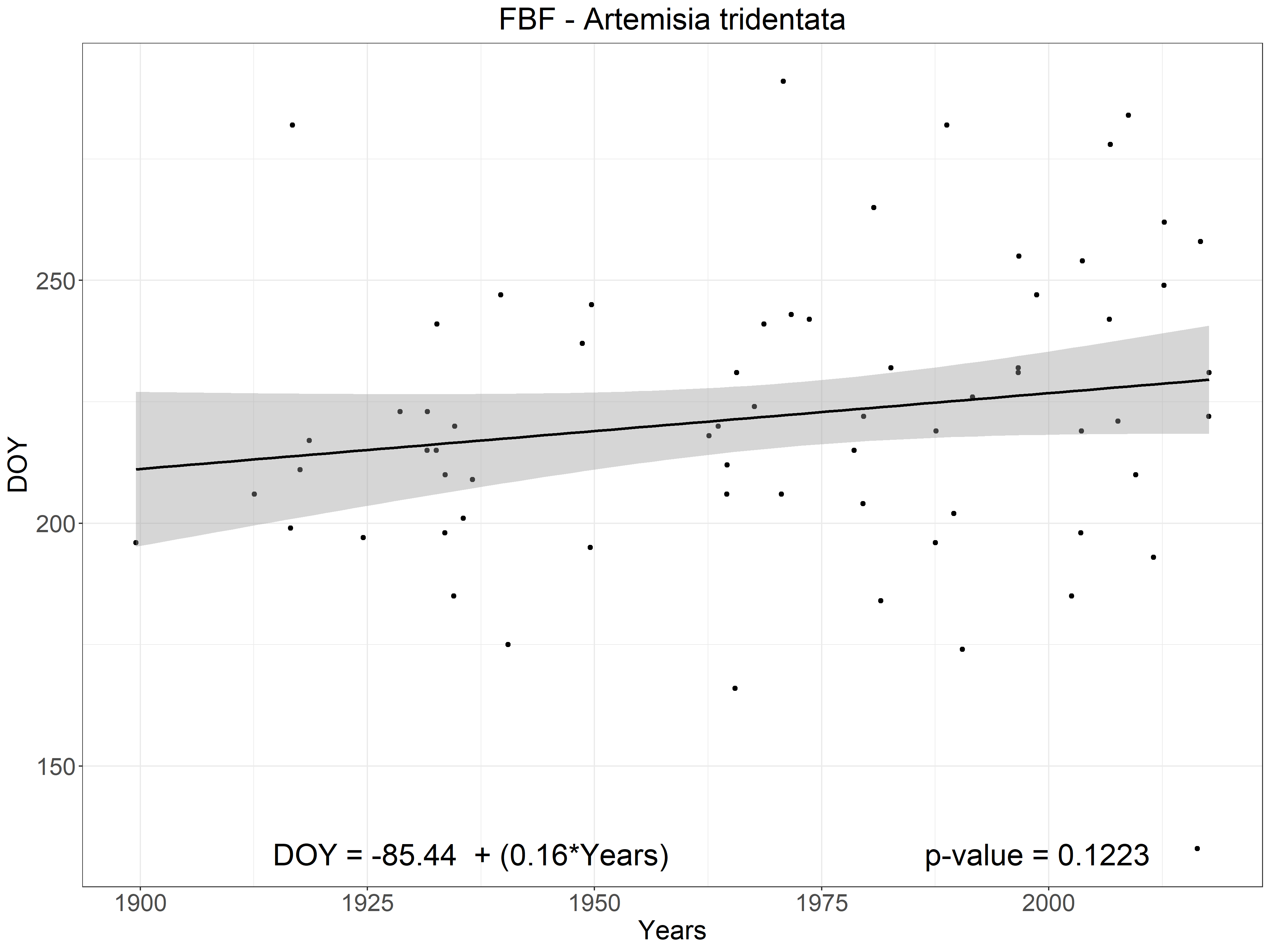

Supplement: Supplementary file 1 [file plants-14-00843-s001.zip › File S2-Species/S2.1-DOYvsYears/1_LM/Plots/FBF_Artemisia tridentata_plot.png]

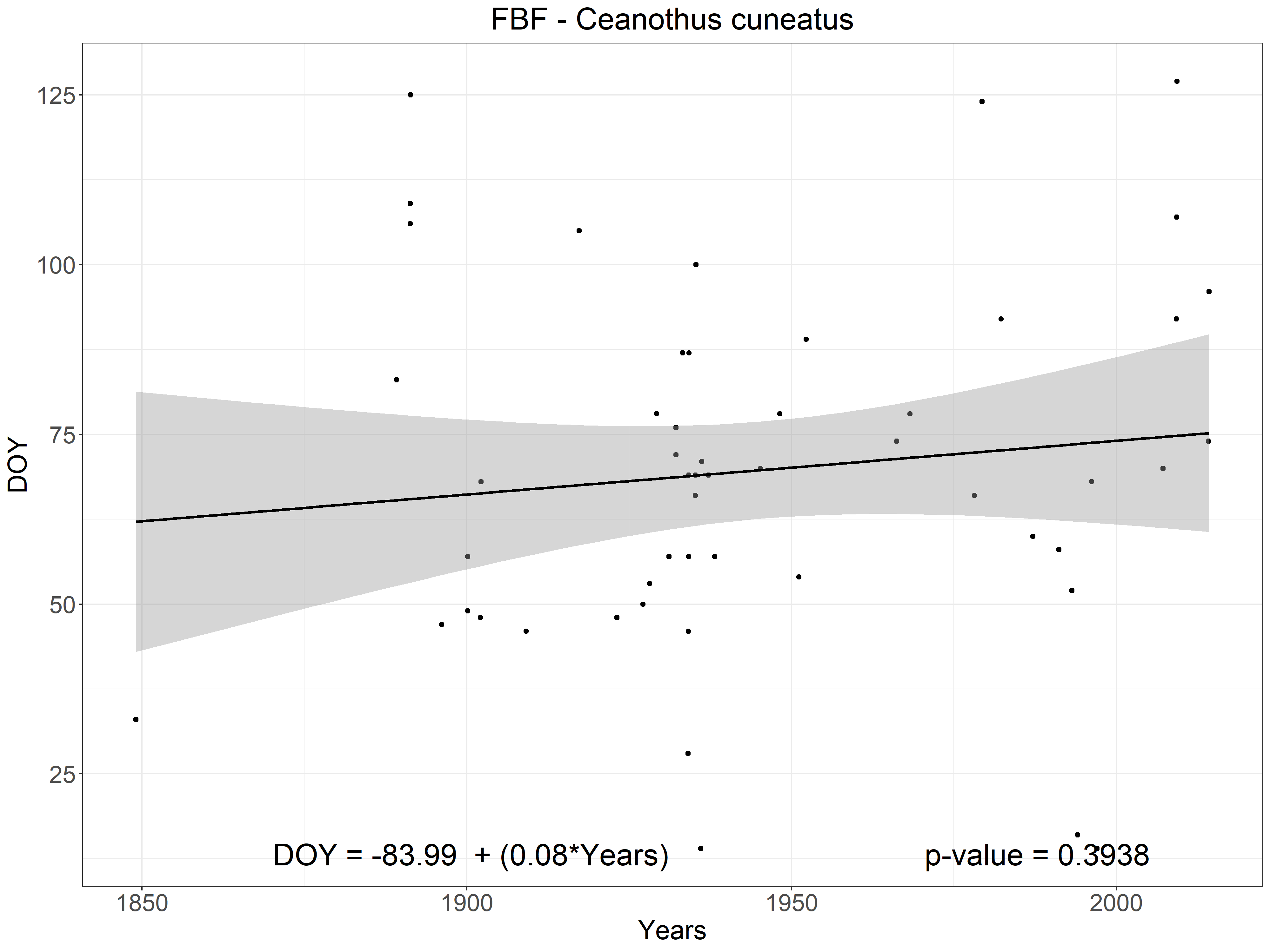

Supplement: Supplementary file 1 [file plants-14-00843-s001.zip › File S2-Species/S2.1-DOYvsYears/1_LM/Plots/FBF_Ceanothus cuneatus_plot.png]

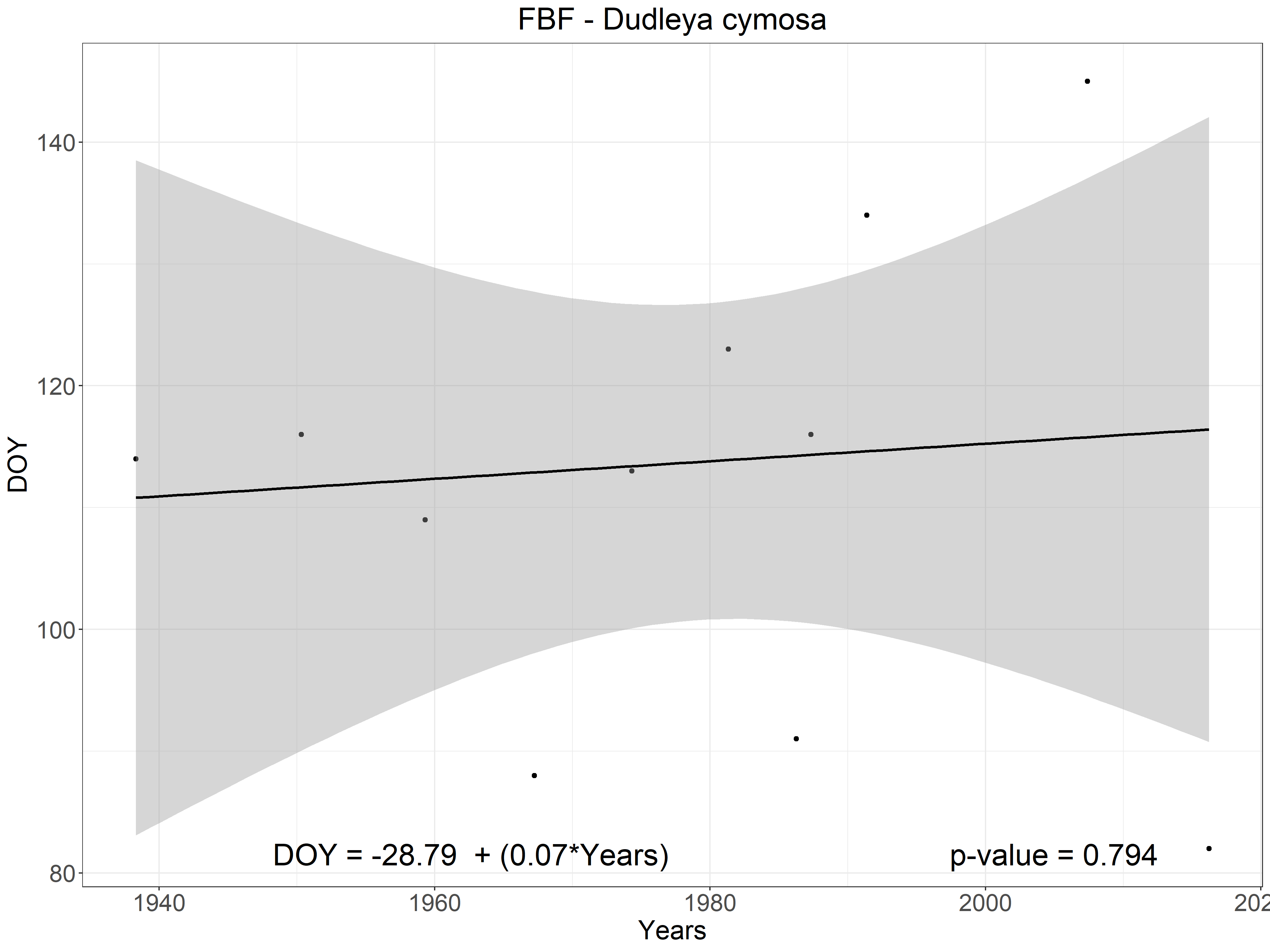

Supplement: Supplementary file 1 [file plants-14-00843-s001.zip › File S2-Species/S2.1-DOYvsYears/1_LM/Plots/FBF_Dudleya cymosa_plot.png]

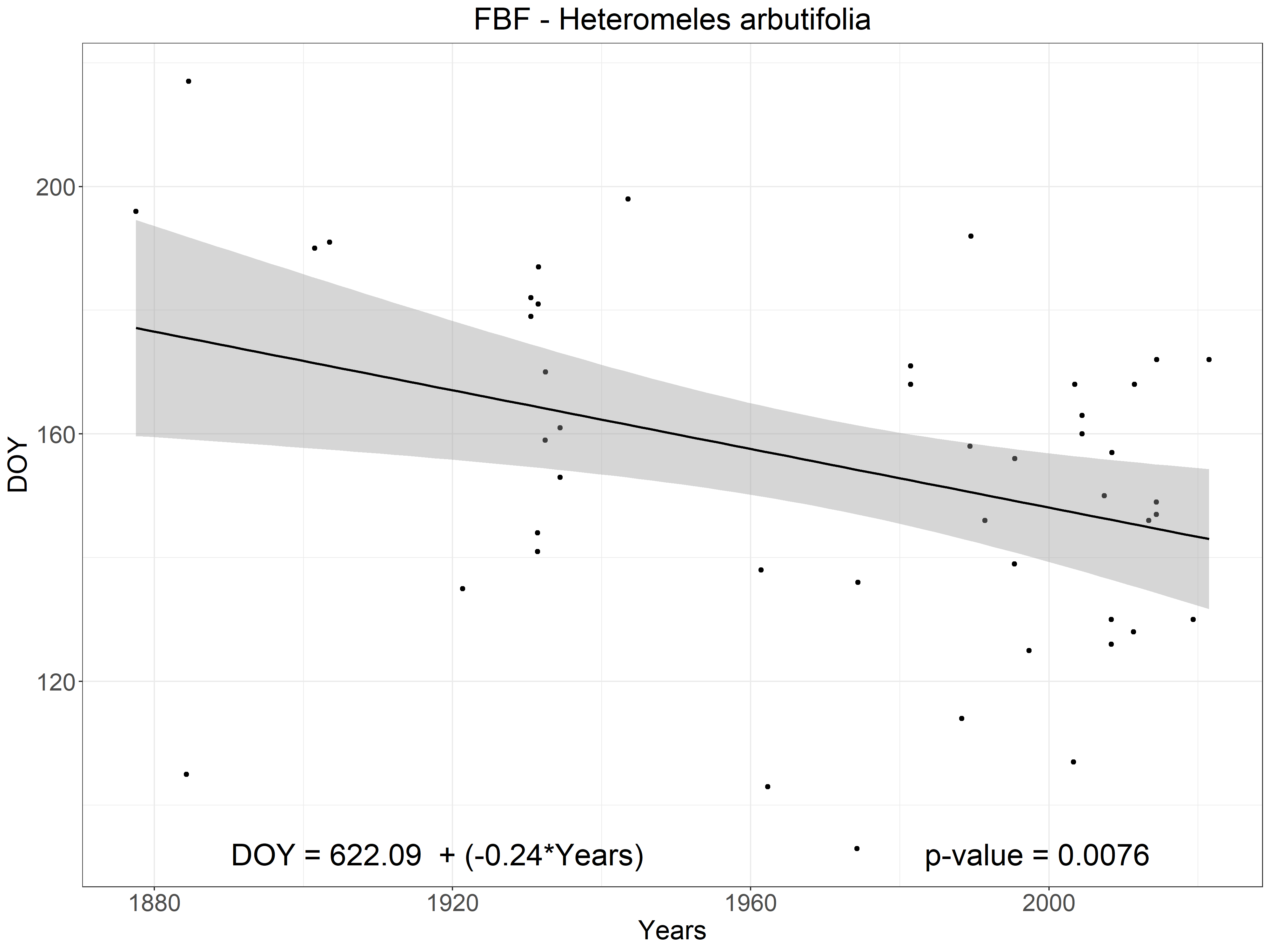

Supplement: Supplementary file 1 [file plants-14-00843-s001.zip › File S2-Species/S2.1-DOYvsYears/1_LM/Plots/FBF_Heteromeles arbutifolia_plot.png]

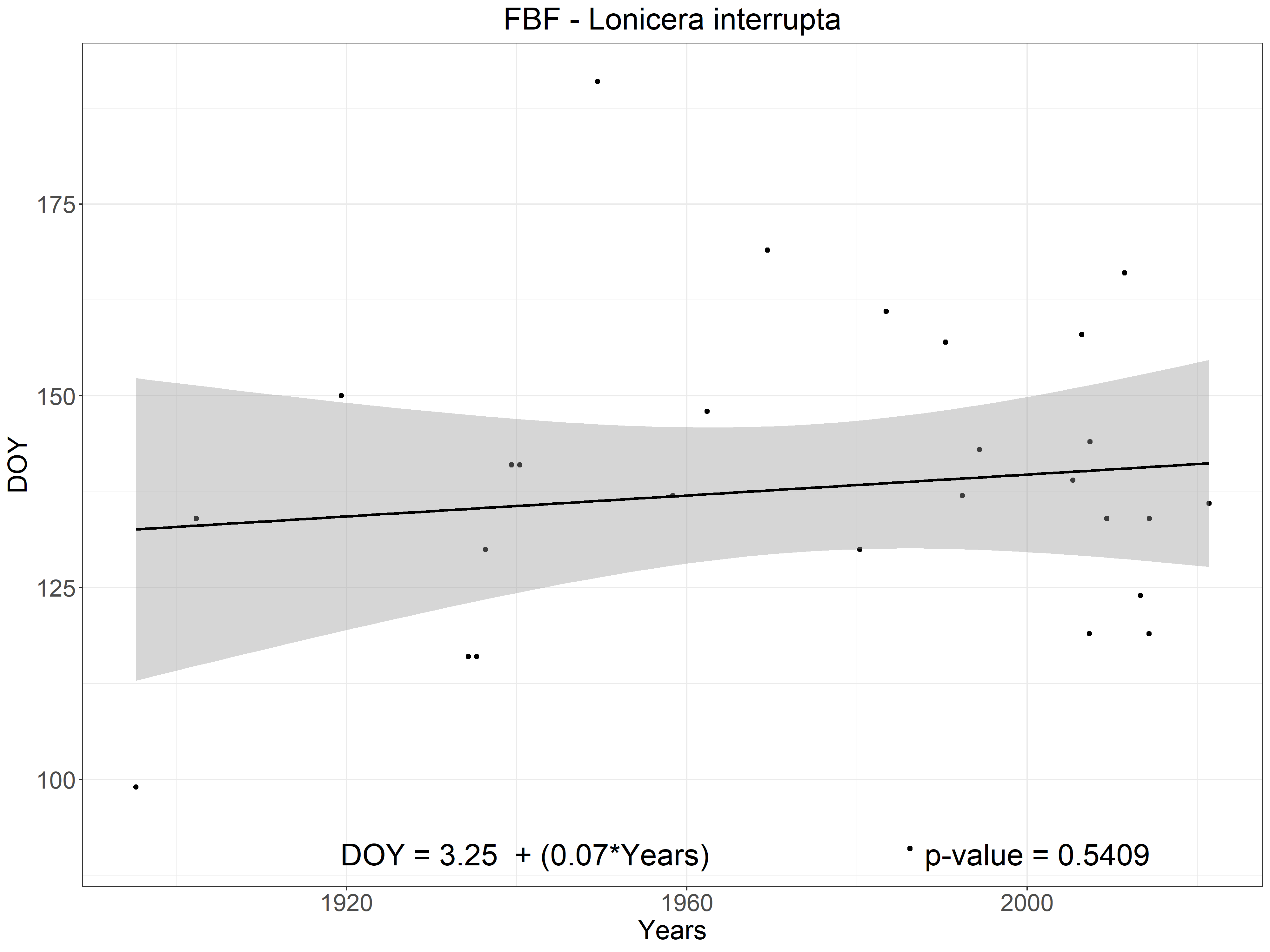

Supplement: Supplementary file 1 [file plants-14-00843-s001.zip › File S2-Species/S2.1-DOYvsYears/1_LM/Plots/FBF_Lonicera interrupta_plot.png]

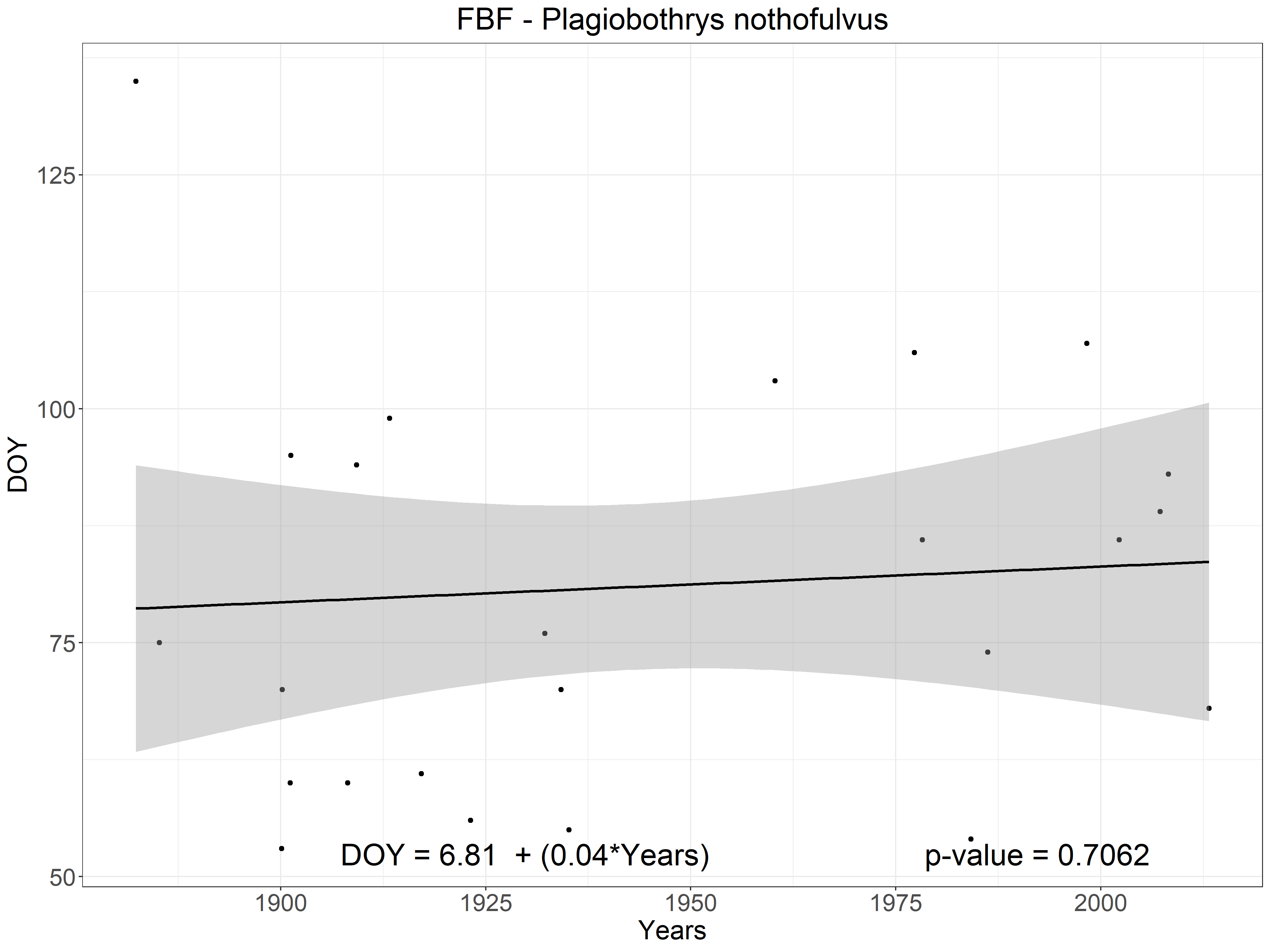

Supplement: Supplementary file 1 [file plants-14-00843-s001.zip › File S2-Species/S2.1-DOYvsYears/1_LM/Plots/FBF_Plagiobothrys nothofulvus_plot.png]

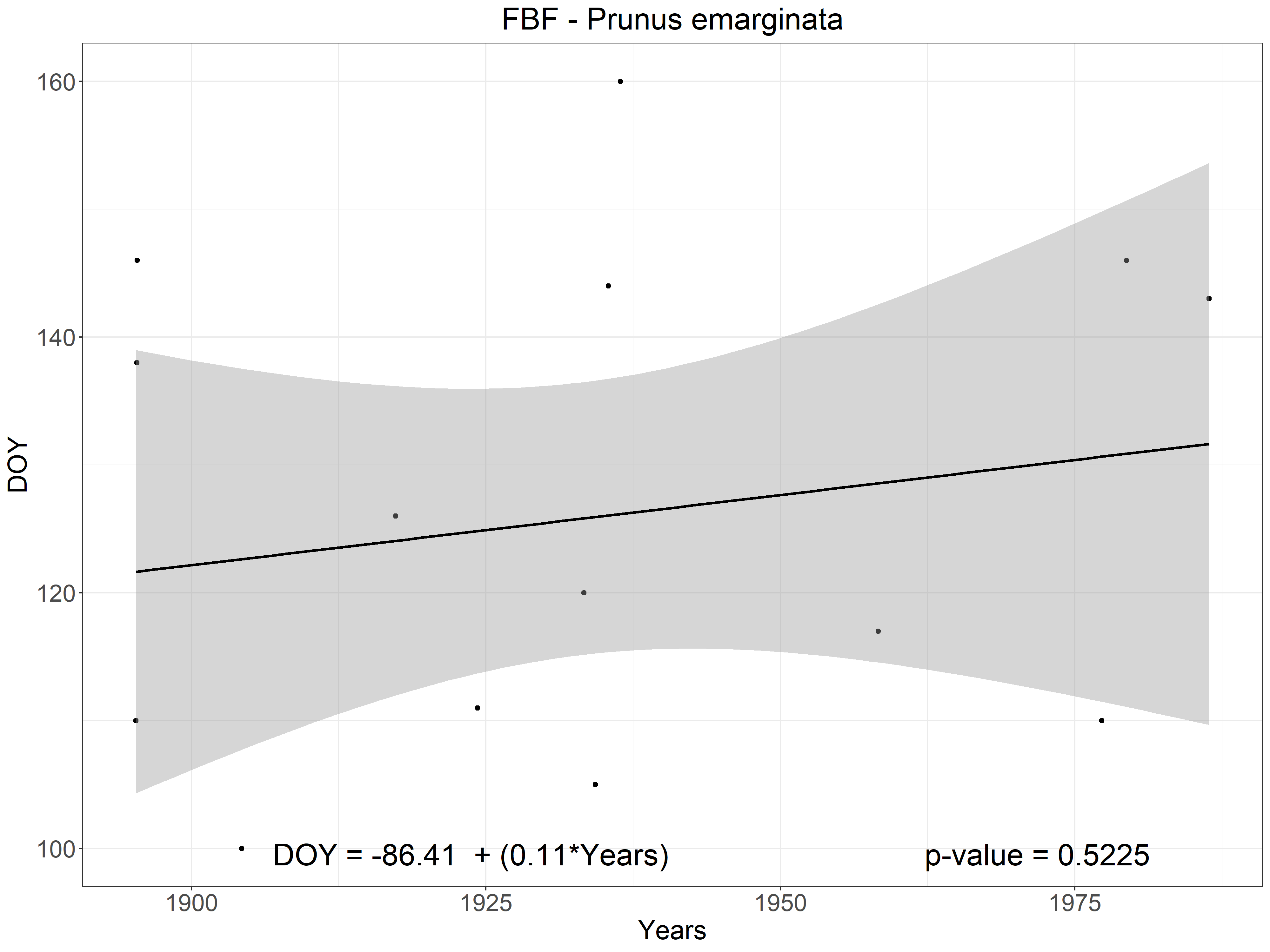

Supplement: Supplementary file 1 [file plants-14-00843-s001.zip › File S2-Species/S2.1-DOYvsYears/1_LM/Plots/FBF_Prunus emarginata_plot.png]

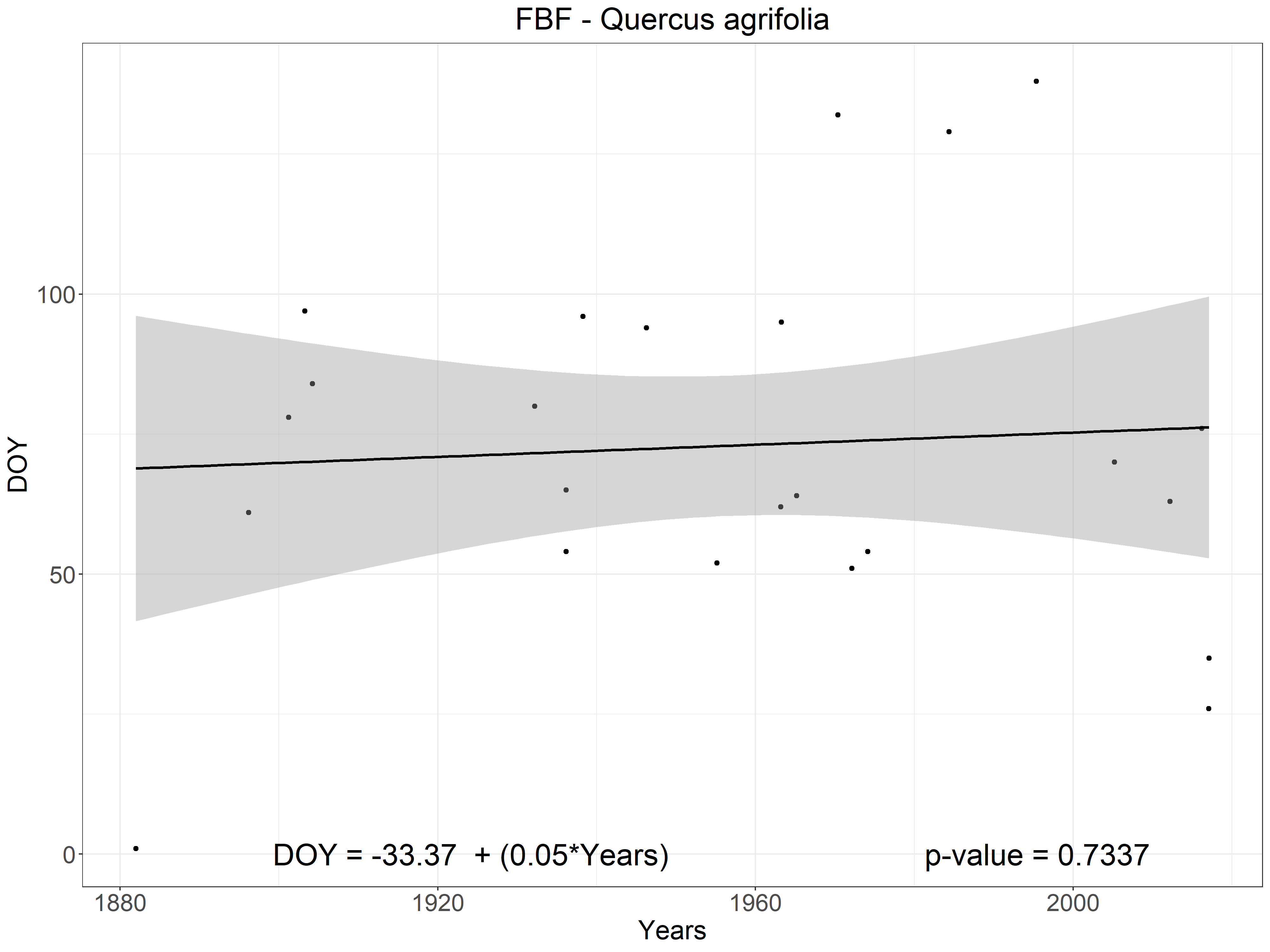

Supplement: Supplementary file 1 [file plants-14-00843-s001.zip › File S2-Species/S2.1-DOYvsYears/1_LM/Plots/FBF_Quercus agrifolia_plot.png]

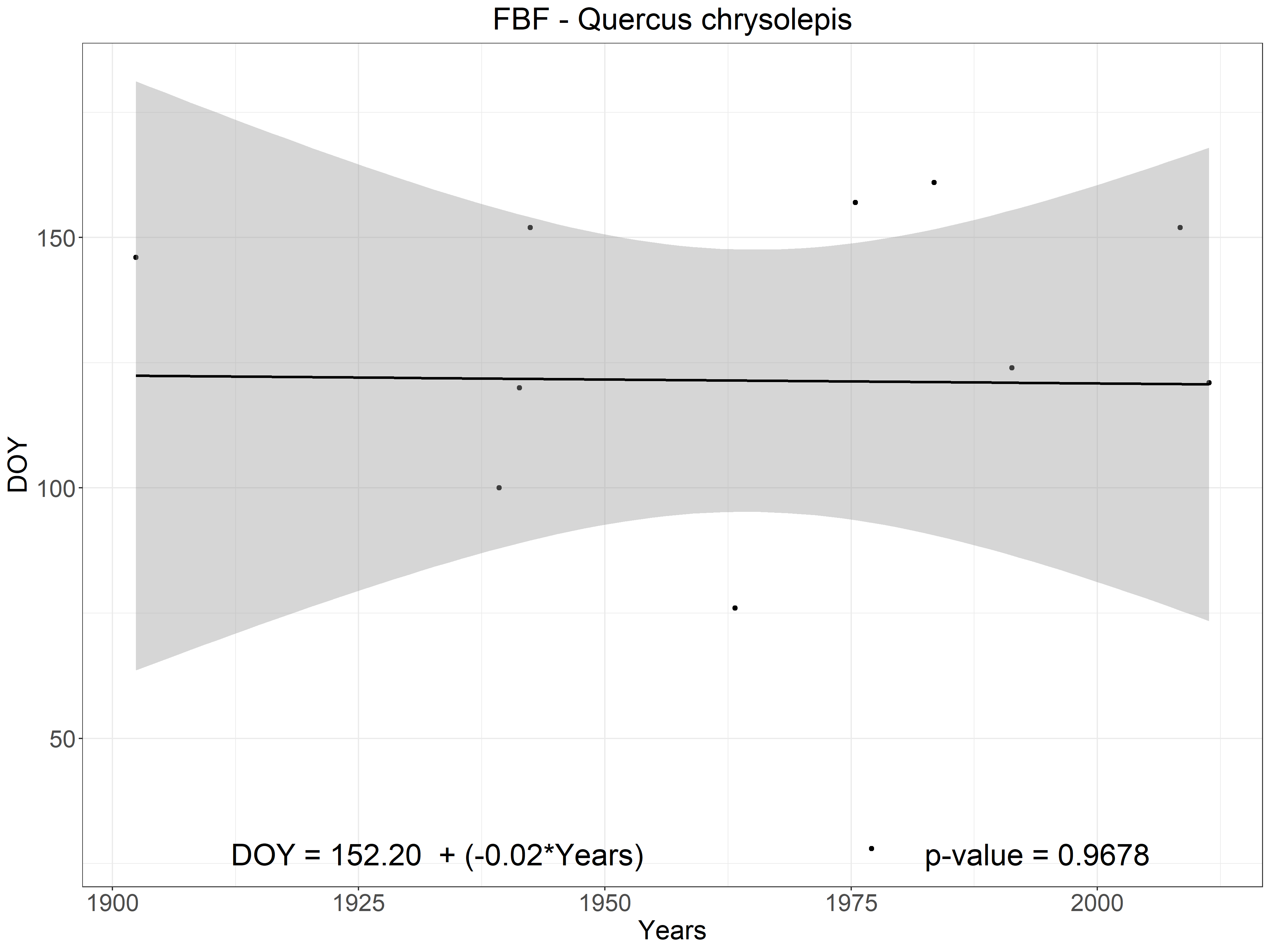

Supplement: Supplementary file 1 [file plants-14-00843-s001.zip › File S2-Species/S2.1-DOYvsYears/1_LM/Plots/FBF_Quercus chrysolepis_plot.png]

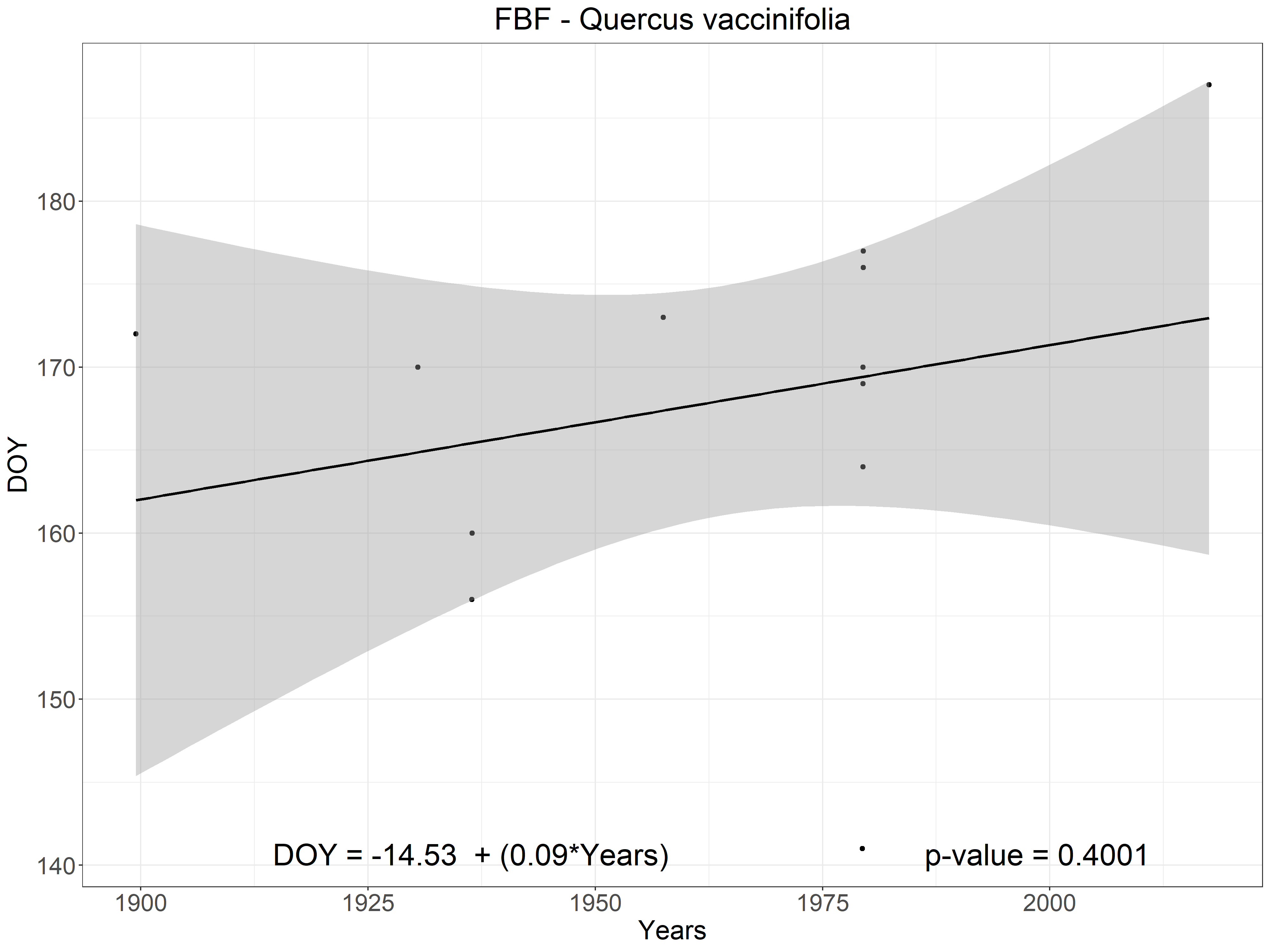

Supplement: Supplementary file 1 [file plants-14-00843-s001.zip › File S2-Species/S2.1-DOYvsYears/1_LM/Plots/FBF_Quercus vaccinifolia_plot.png]

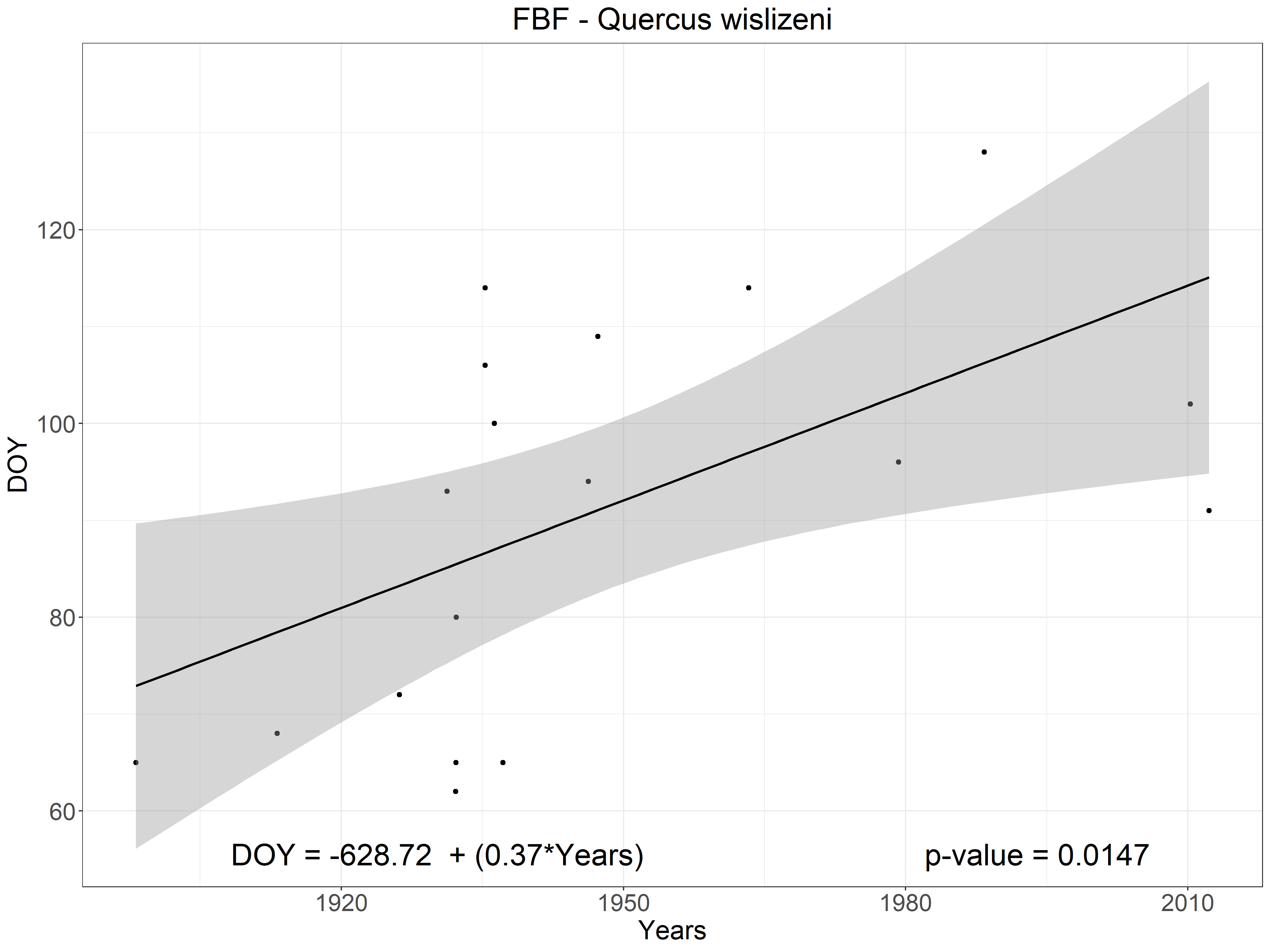

Supplement: Supplementary file 1 [file plants-14-00843-s001.zip › File S2-Species/S2.1-DOYvsYears/1_LM/Plots/FBF_Quercus wislizeni_plot.png]

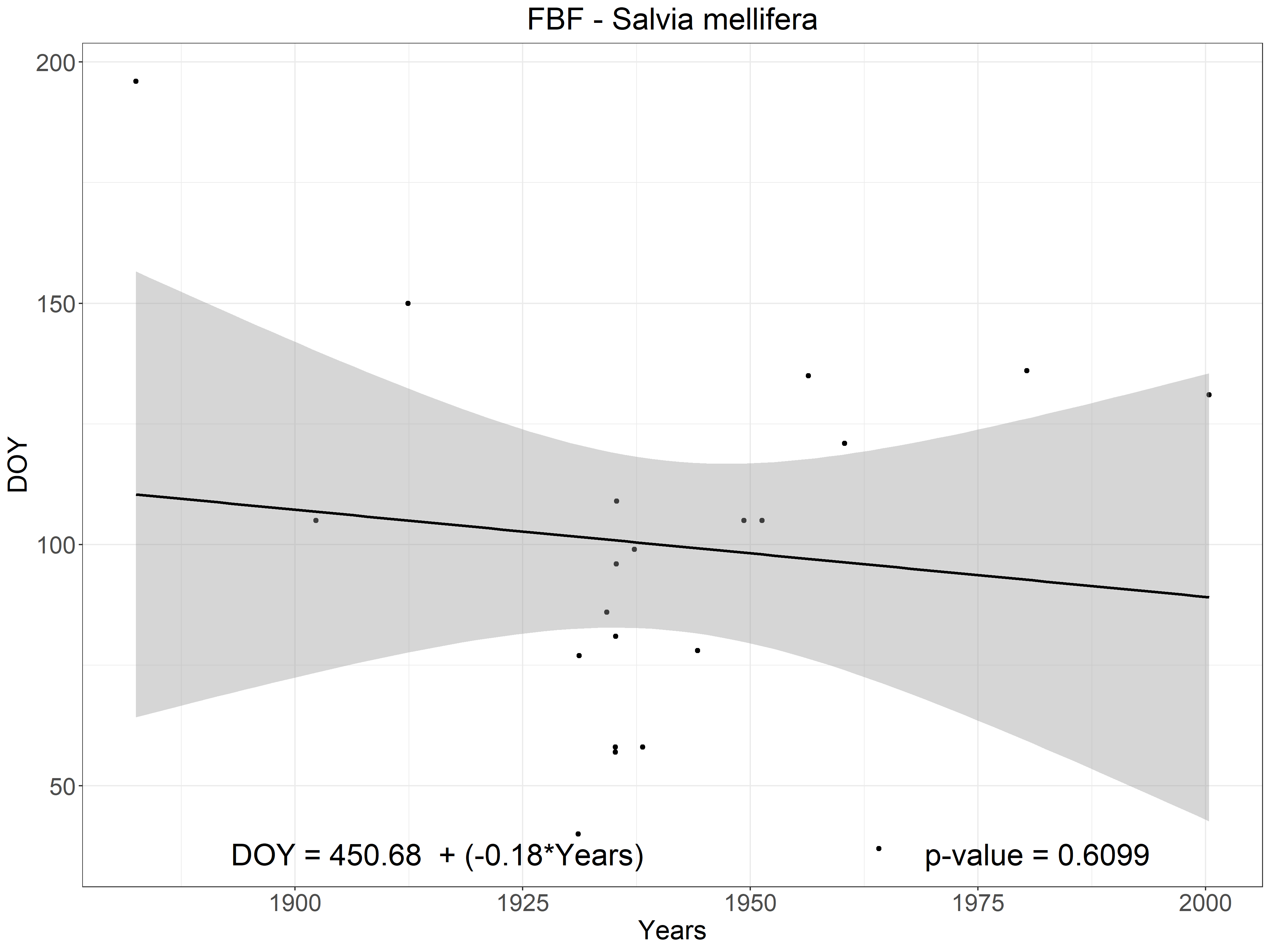

Supplement: Supplementary file 1 [file plants-14-00843-s001.zip › File S2-Species/S2.1-DOYvsYears/1_LM/Plots/FBF_Salvia mellifera_plot.png]

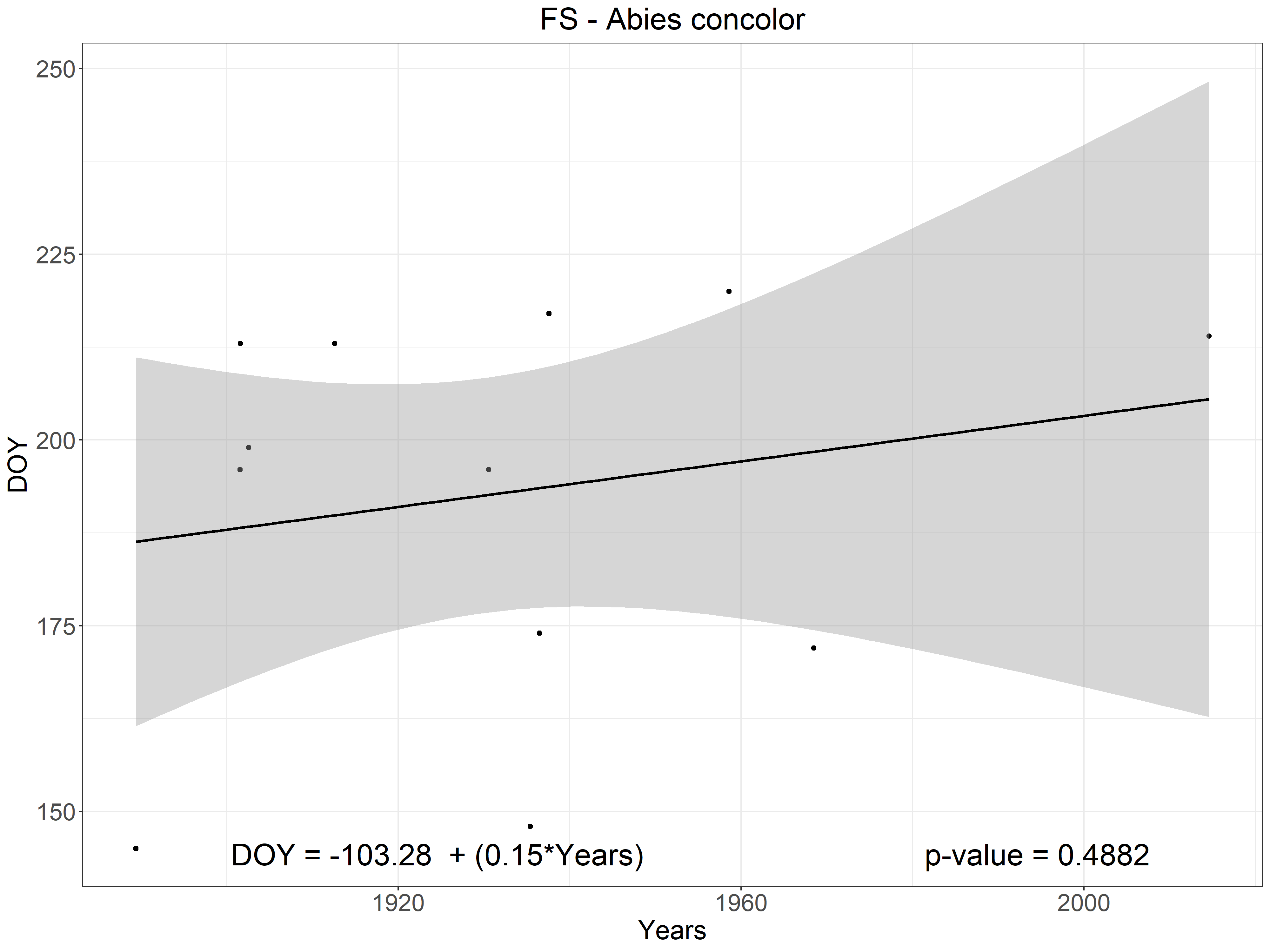

Supplement: Supplementary file 1 [file plants-14-00843-s001.zip › File S2-Species/S2.1-DOYvsYears/1_LM/Plots/FS_Abies concolor_plot.png]

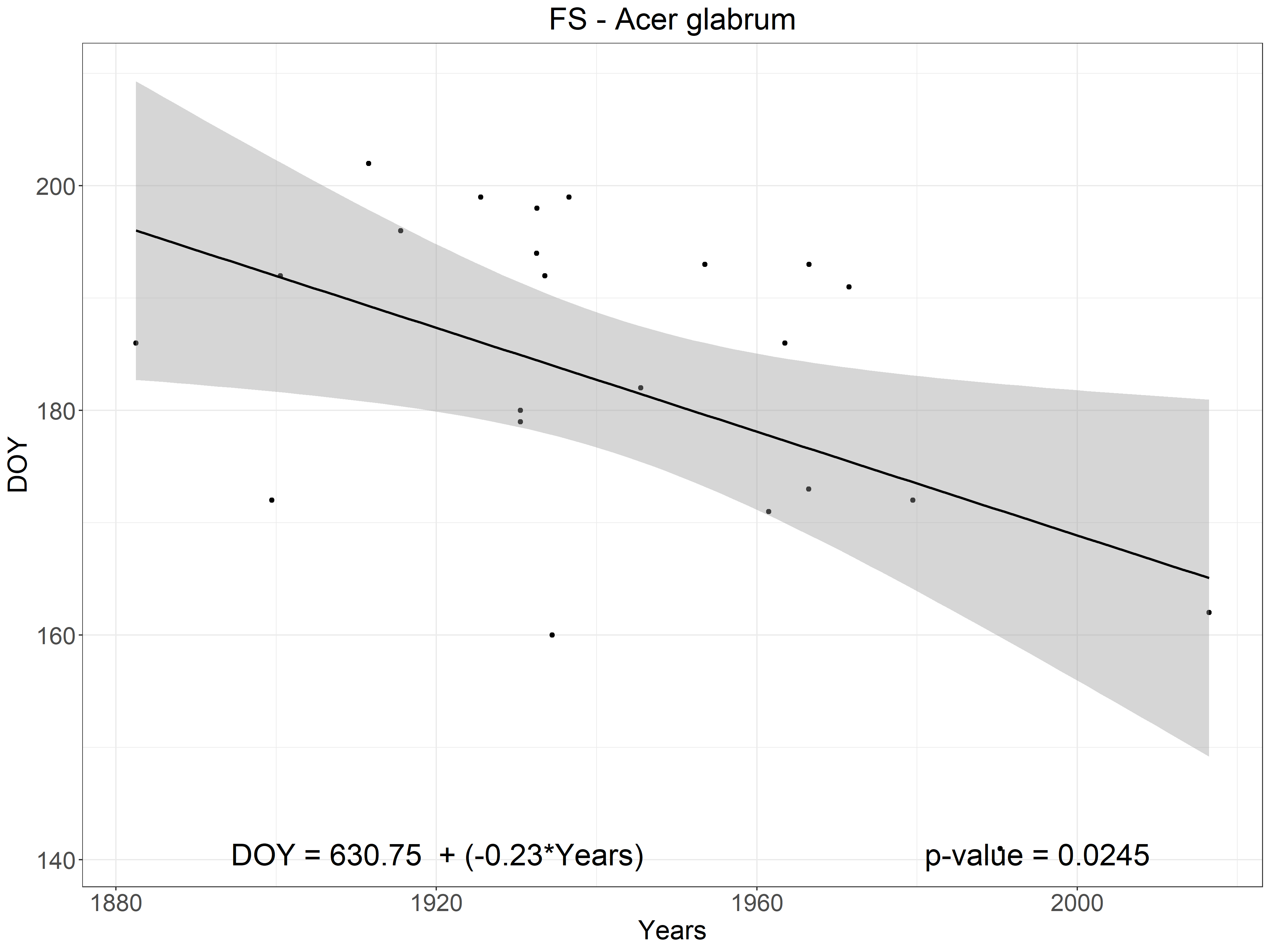

Supplement: Supplementary file 1 [file plants-14-00843-s001.zip › File S2-Species/S2.1-DOYvsYears/1_LM/Plots/FS_Acer glabrum_plot.png]

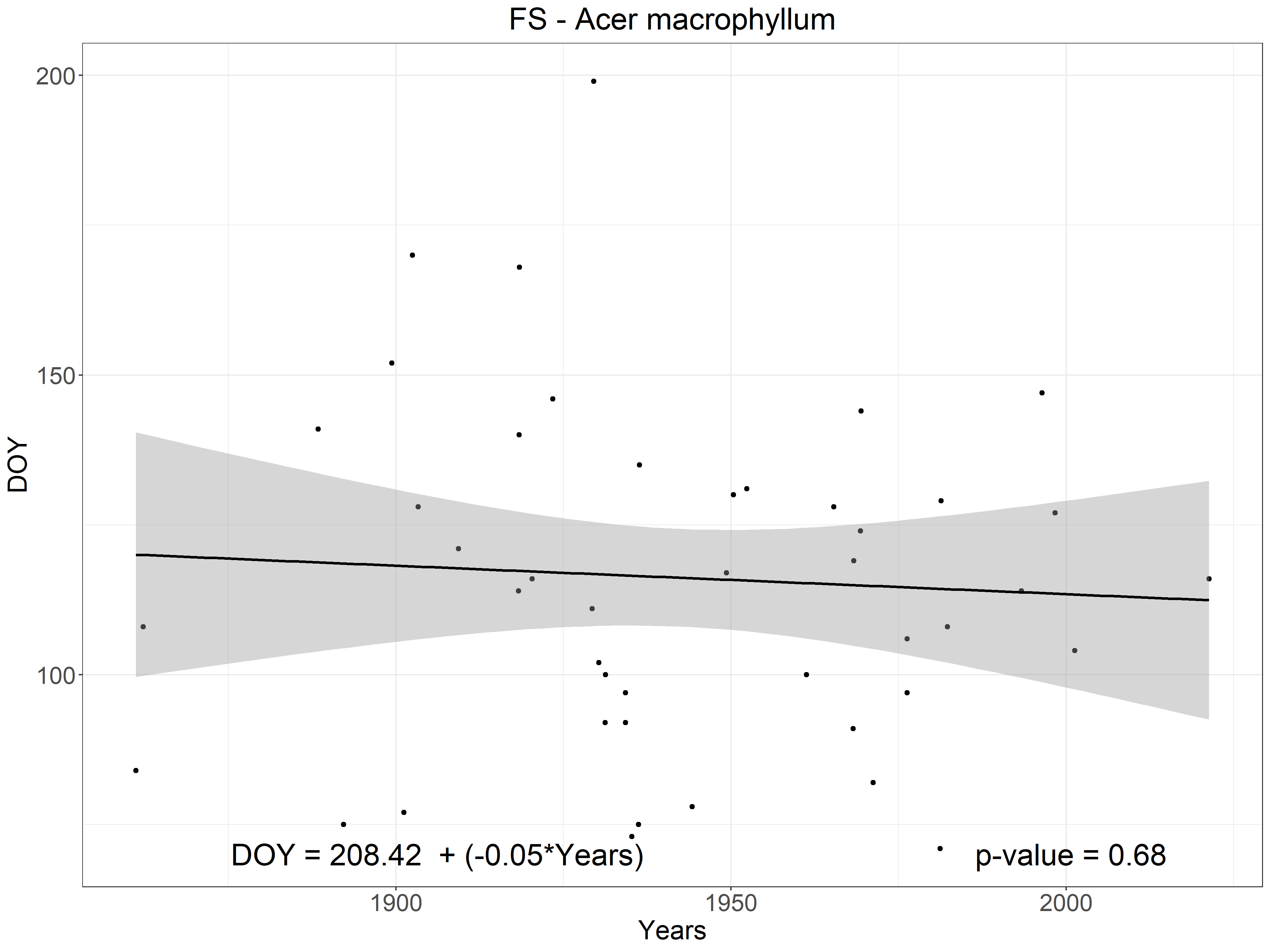

Supplement: Supplementary file 1 [file plants-14-00843-s001.zip › File S2-Species/S2.1-DOYvsYears/1_LM/Plots/FS_Acer macrophyllum_plot.png]

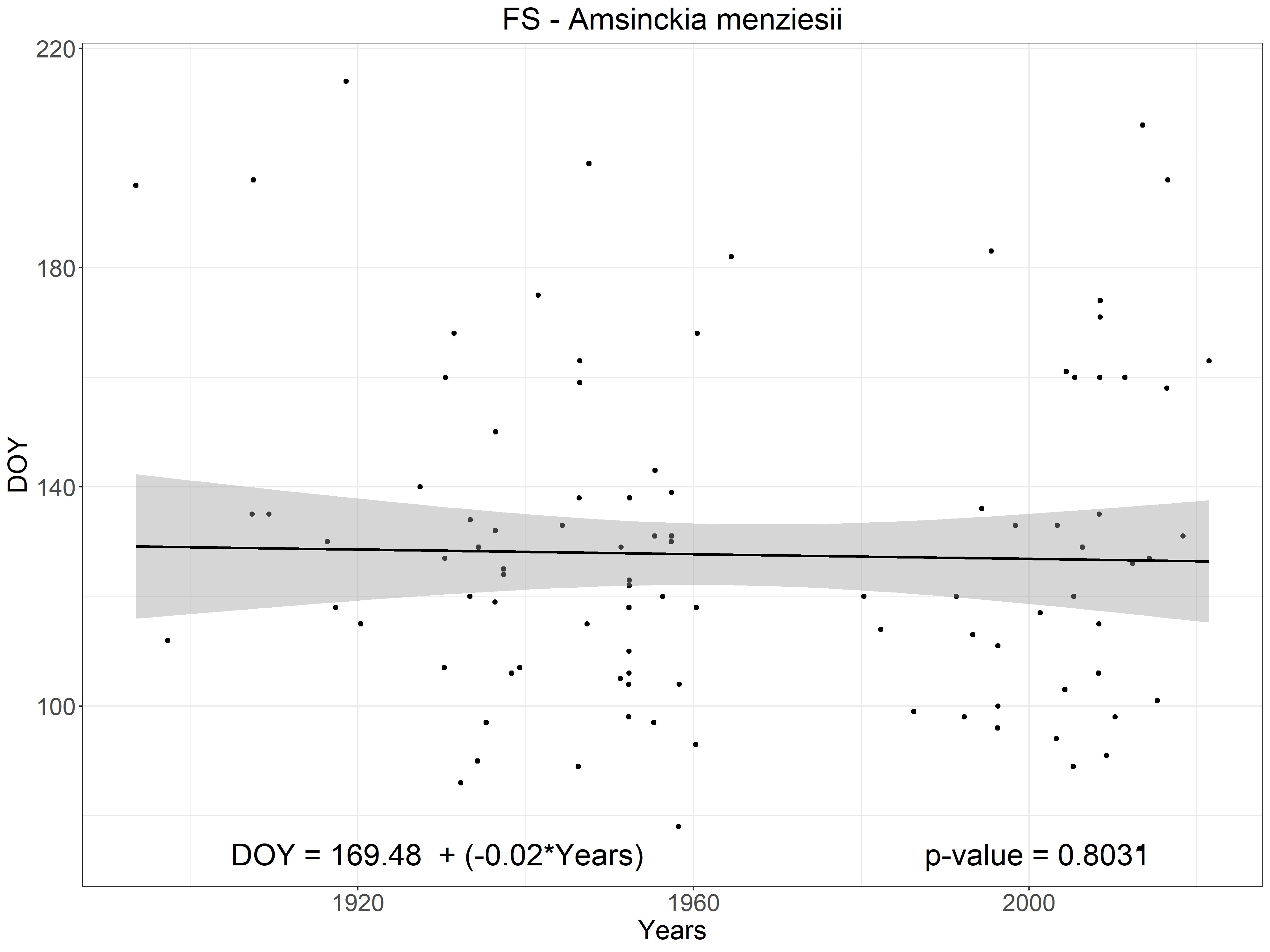

Supplement: Supplementary file 1 [file plants-14-00843-s001.zip › File S2-Species/S2.1-DOYvsYears/1_LM/Plots/FS_Amsinckia menziesii_plot.png]

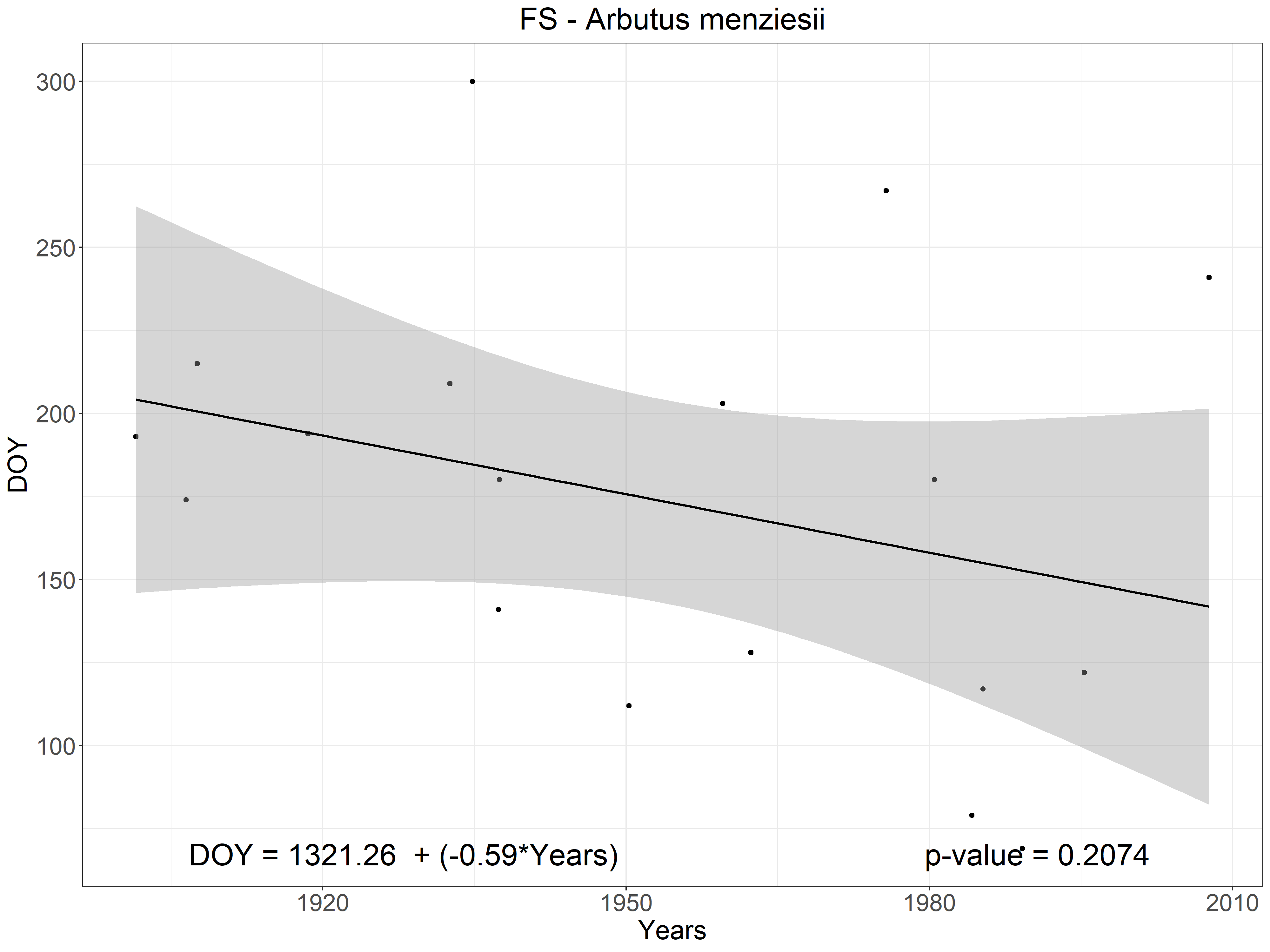

Supplement: Supplementary file 1 [file plants-14-00843-s001.zip › File S2-Species/S2.1-DOYvsYears/1_LM/Plots/FS_Arbutus menziesii_plot.png]

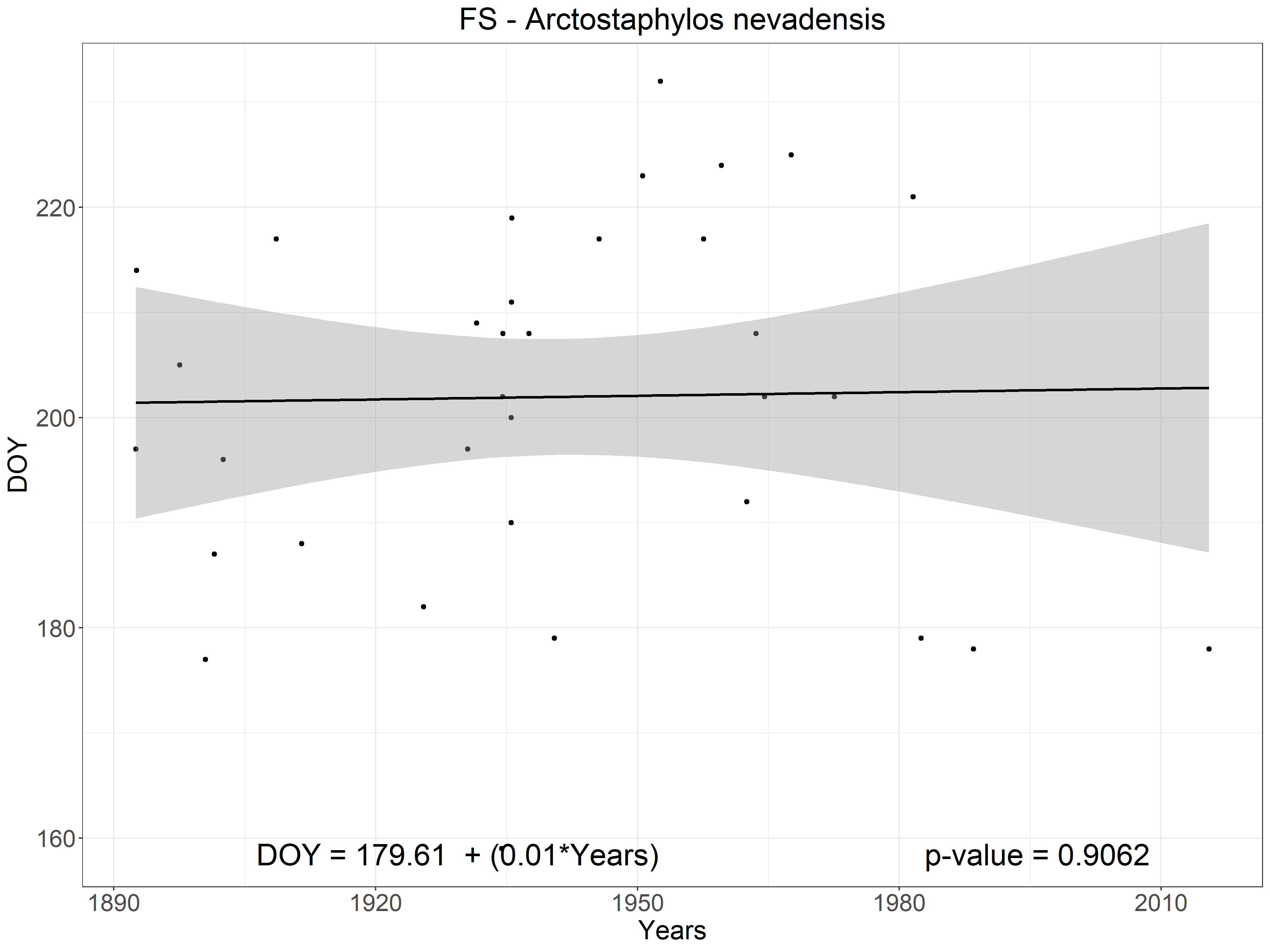

Supplement: Supplementary file 1 [file plants-14-00843-s001.zip › File S2-Species/S2.1-DOYvsYears/1_LM/Plots/FS_Arctostaphylos nevadensis_plot.png]

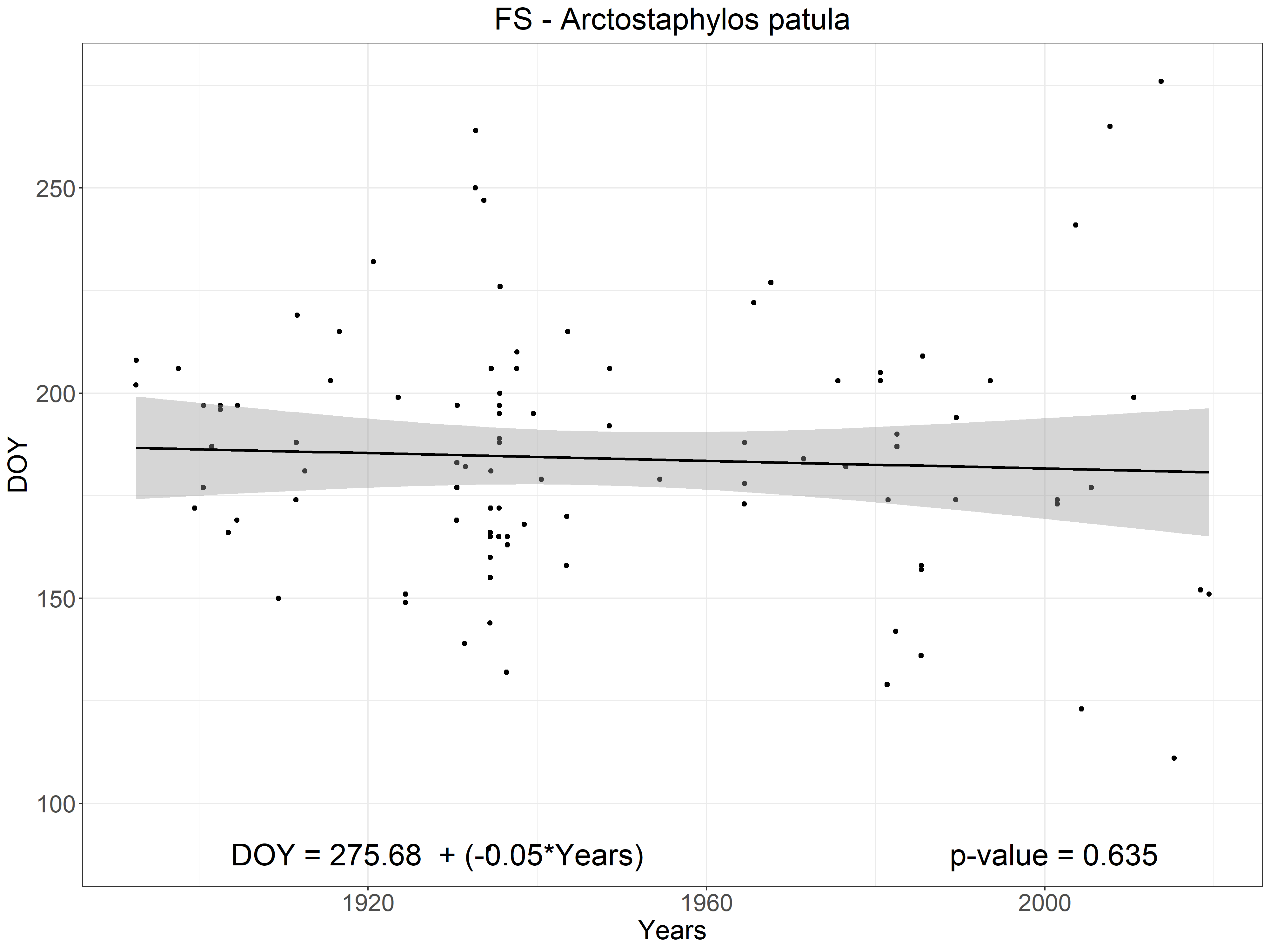

Supplement: Supplementary file 1 [file plants-14-00843-s001.zip › File S2-Species/S2.1-DOYvsYears/1_LM/Plots/FS_Arctostaphylos patula_plot.png]

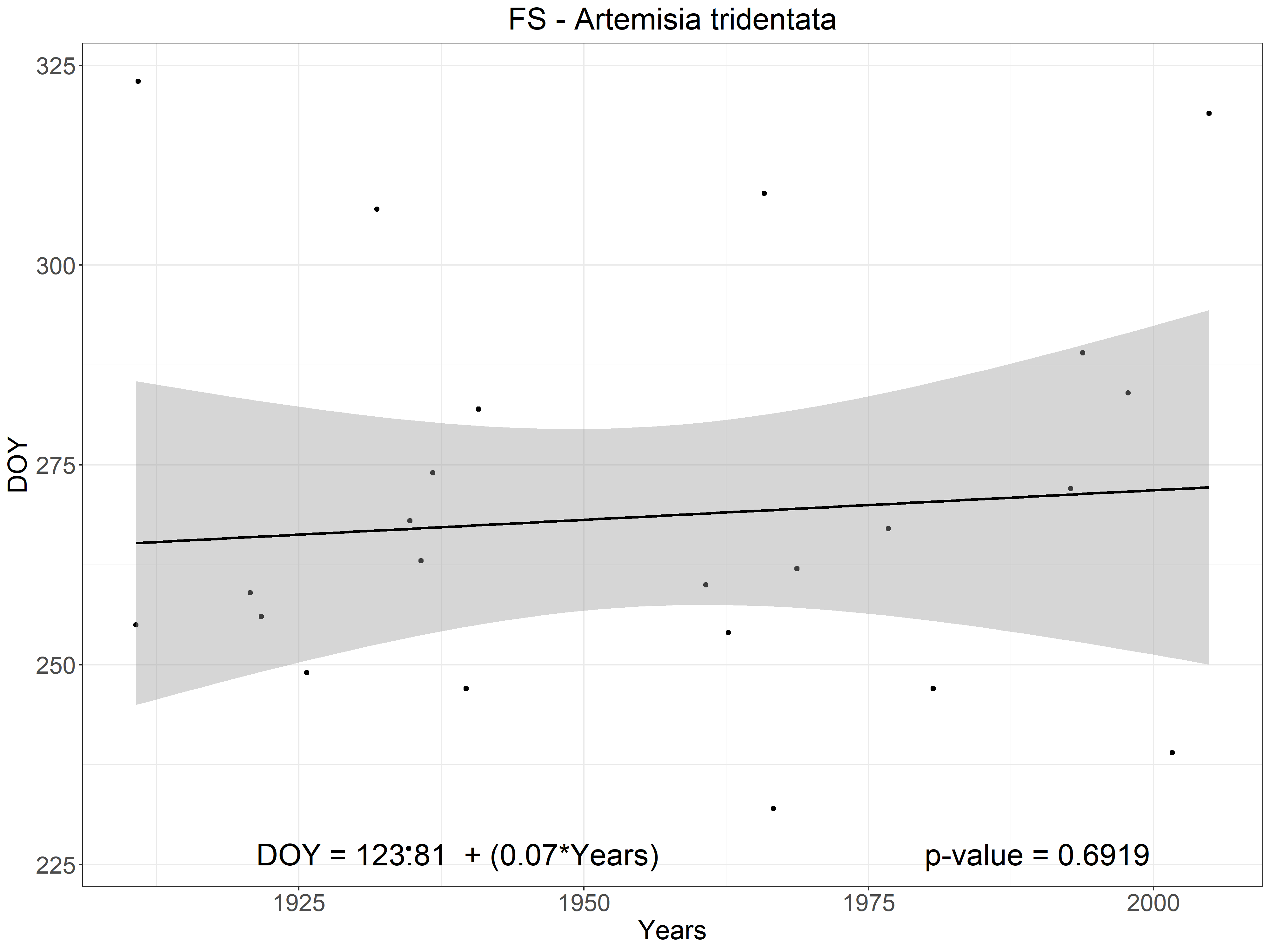

Supplement: Supplementary file 1 [file plants-14-00843-s001.zip › File S2-Species/S2.1-DOYvsYears/1_LM/Plots/FS_Artemisia tridentata_plot.png]

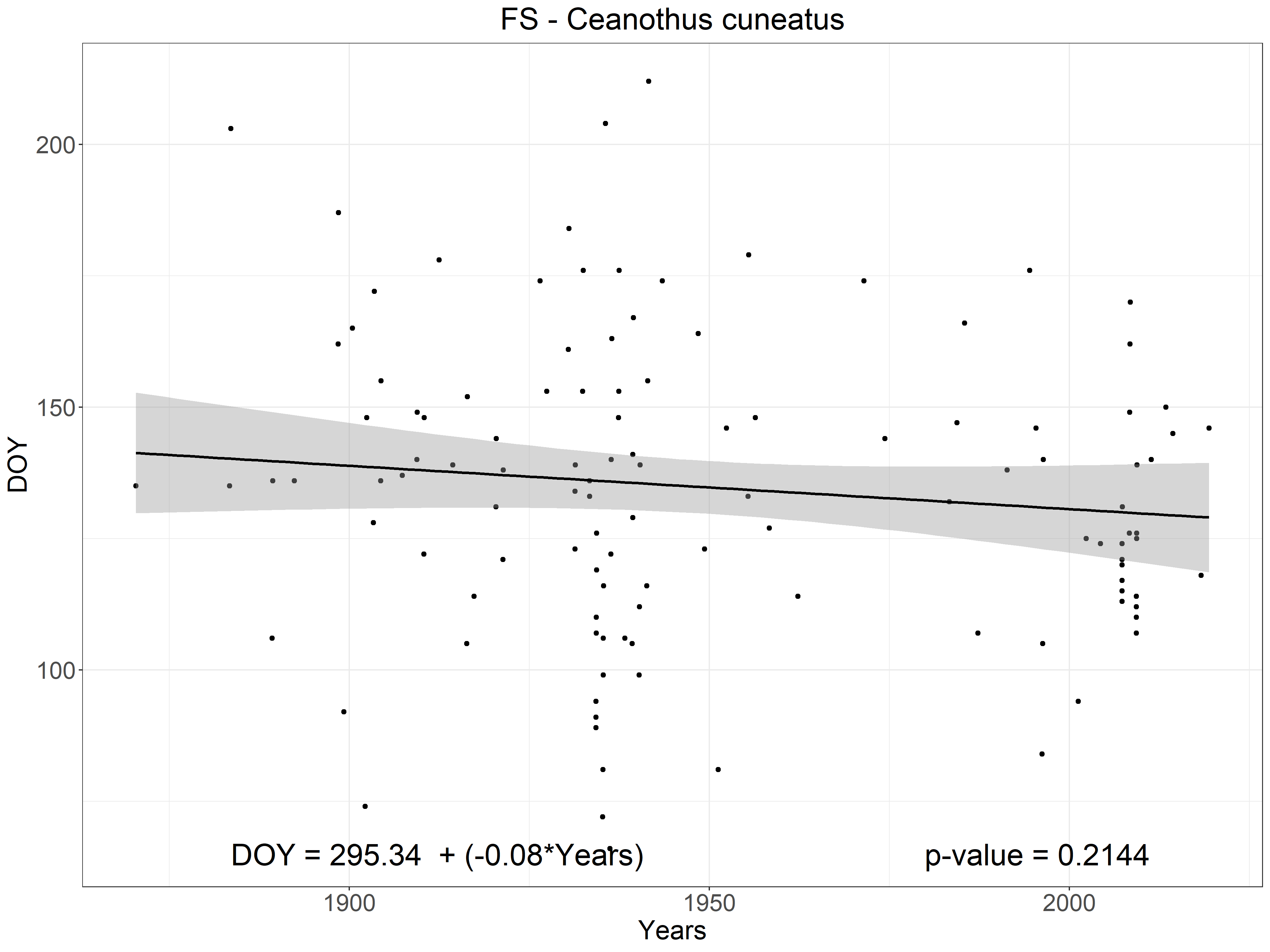

Supplement: Supplementary file 1 [file plants-14-00843-s001.zip › File S2-Species/S2.1-DOYvsYears/1_LM/Plots/FS_Ceanothus cuneatus_plot.png]

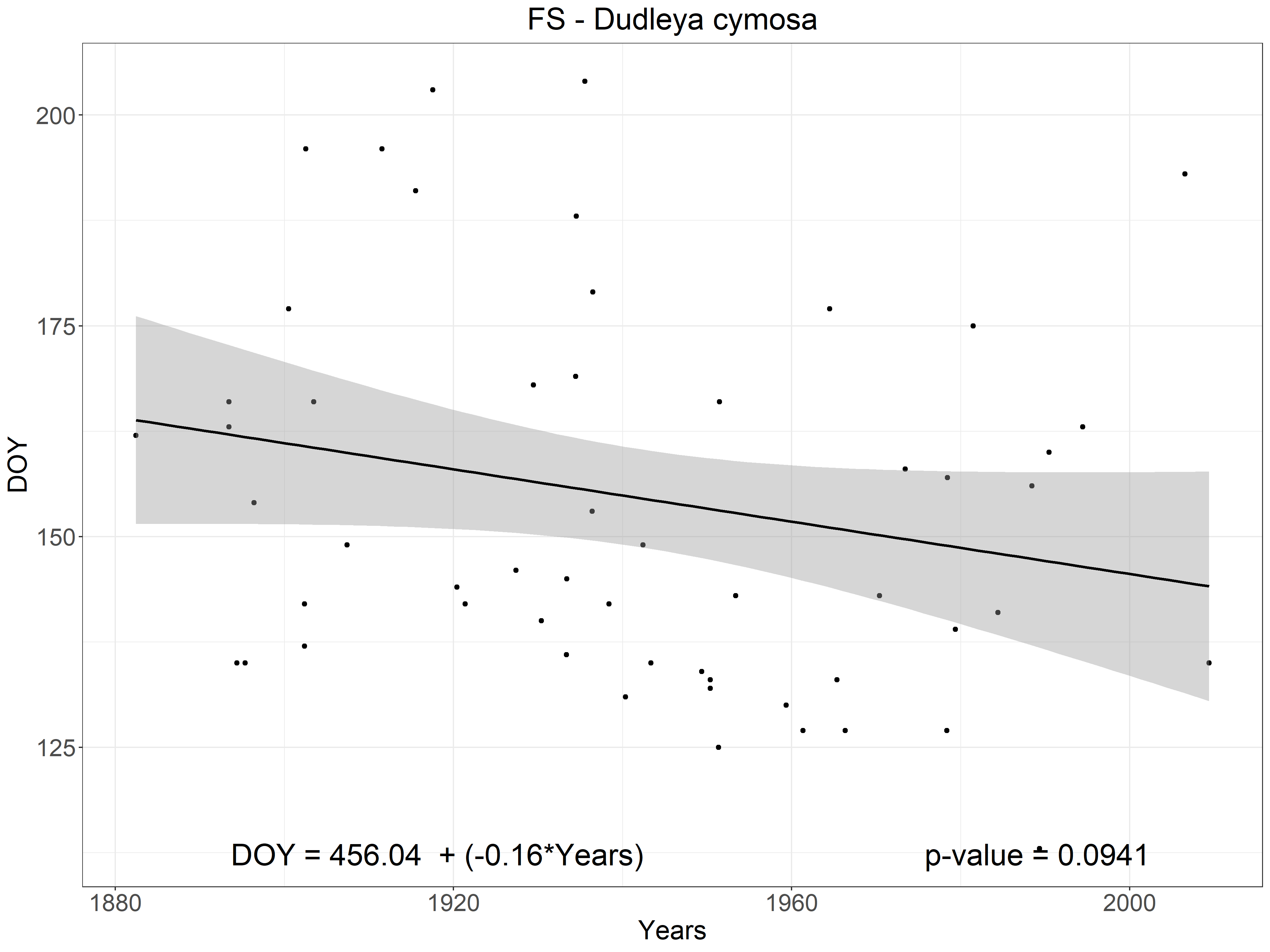

Supplement: Supplementary file 1 [file plants-14-00843-s001.zip › File S2-Species/S2.1-DOYvsYears/1_LM/Plots/FS_Dudleya cymosa_plot.png]

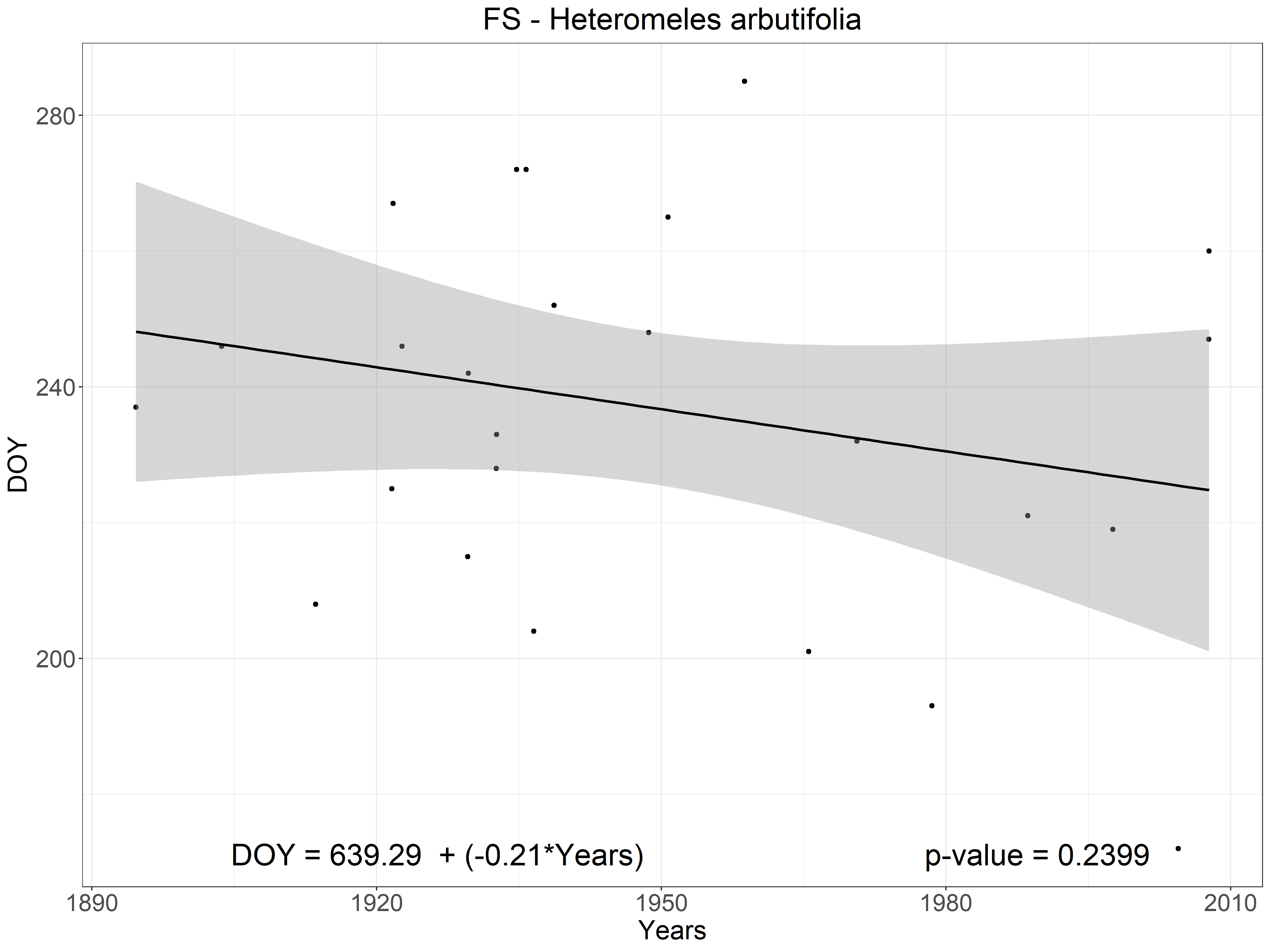

Supplement: Supplementary file 1 [file plants-14-00843-s001.zip › File S2-Species/S2.1-DOYvsYears/1_LM/Plots/FS_Heteromeles arbutifolia_plot.png]

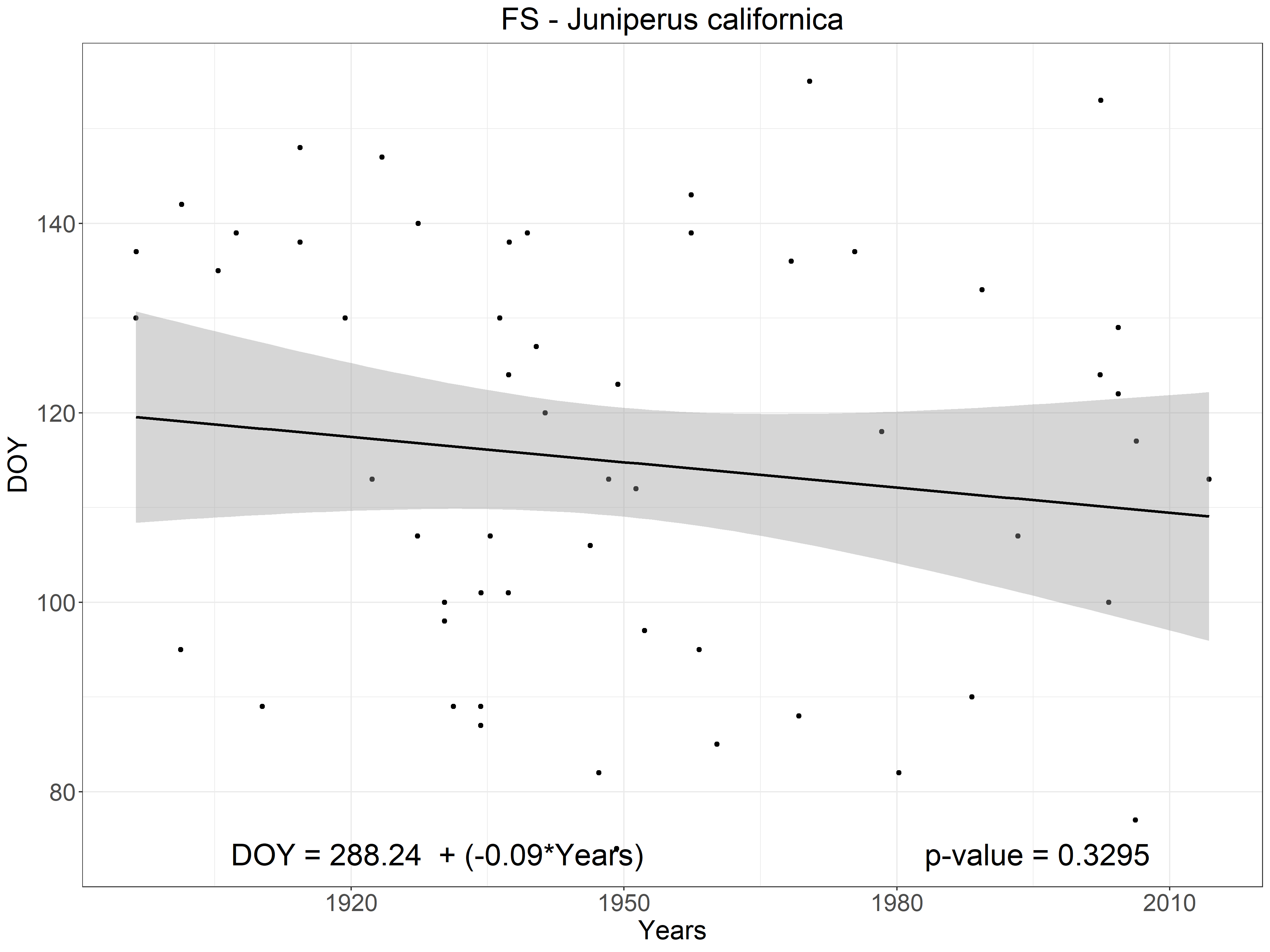

Supplement: Supplementary file 1 [file plants-14-00843-s001.zip › File S2-Species/S2.1-DOYvsYears/1_LM/Plots/FS_Juniperus californica_plot.png]

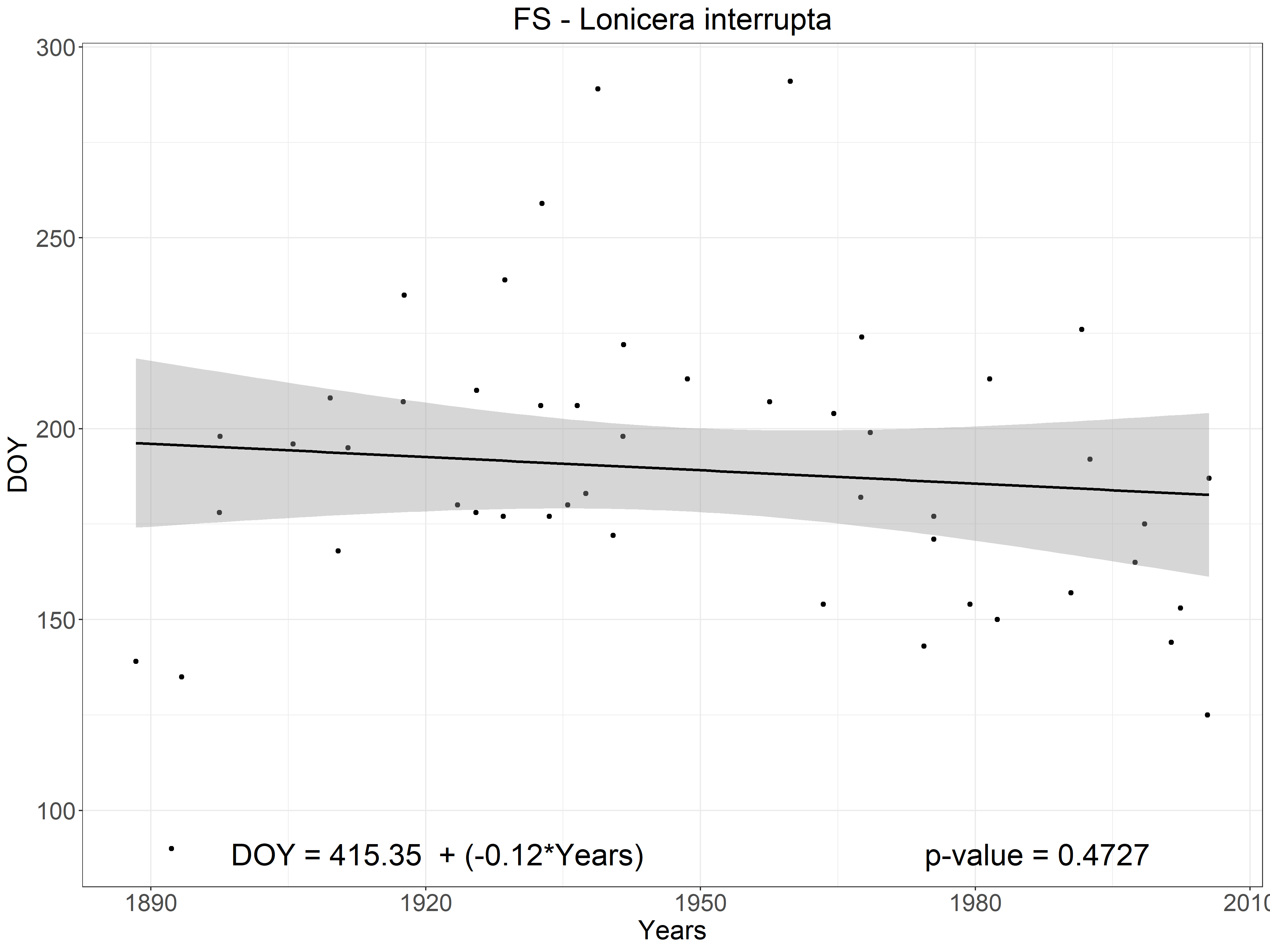

Supplement: Supplementary file 1 [file plants-14-00843-s001.zip › File S2-Species/S2.1-DOYvsYears/1_LM/Plots/FS_Lonicera interrupta_plot.png]

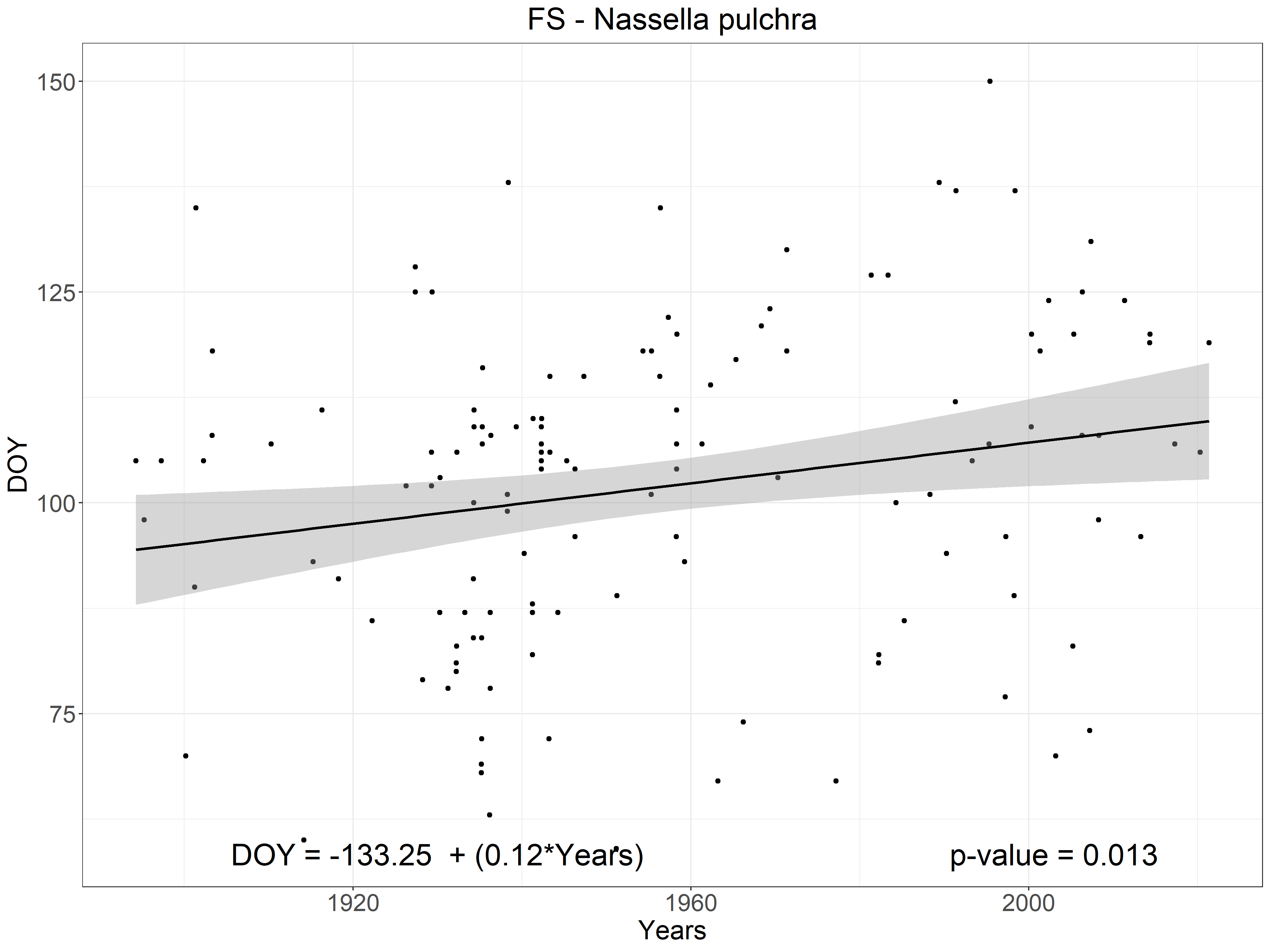

Supplement: Supplementary file 1 [file plants-14-00843-s001.zip › File S2-Species/S2.1-DOYvsYears/1_LM/Plots/FS_Nassella pulchra_plot.png]

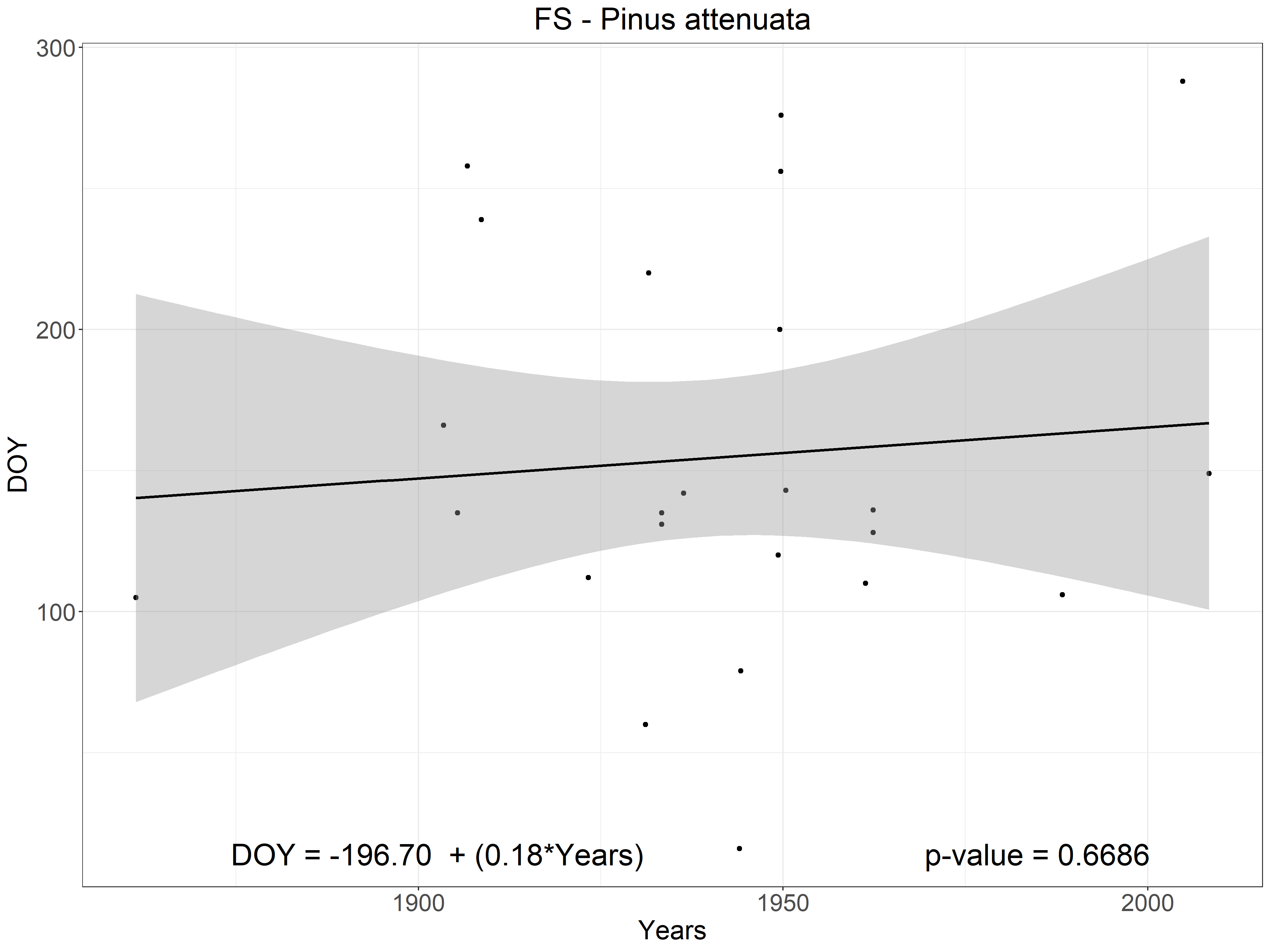

Supplement: Supplementary file 1 [file plants-14-00843-s001.zip › File S2-Species/S2.1-DOYvsYears/1_LM/Plots/FS_Pinus attenuata_plot.png]

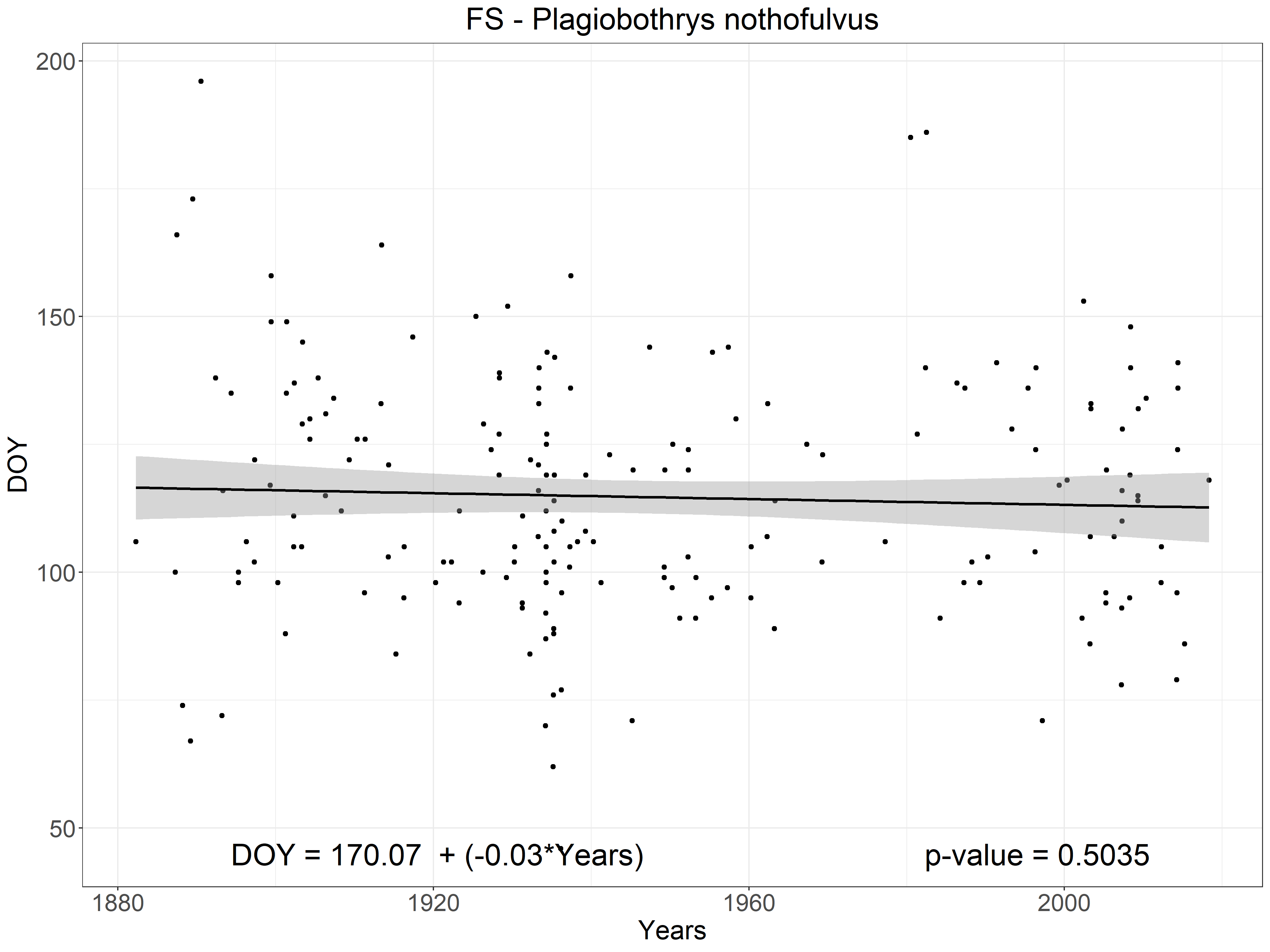

Supplement: Supplementary file 1 [file plants-14-00843-s001.zip › File S2-Species/S2.1-DOYvsYears/1_LM/Plots/FS_Plagiobothrys nothofulvus_plot.png]

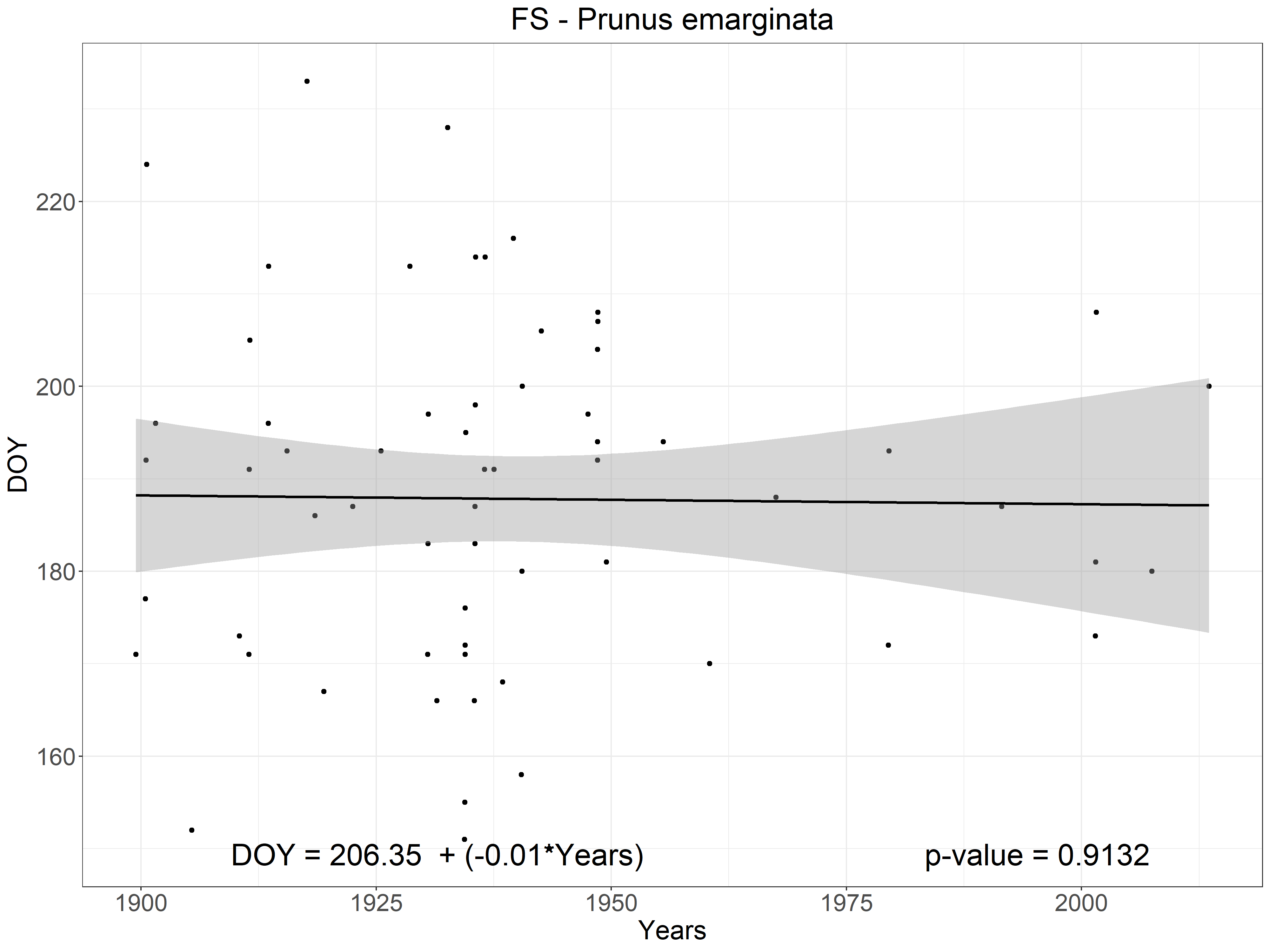

Supplement: Supplementary file 1 [file plants-14-00843-s001.zip › File S2-Species/S2.1-DOYvsYears/1_LM/Plots/FS_Prunus emarginata_plot.png]

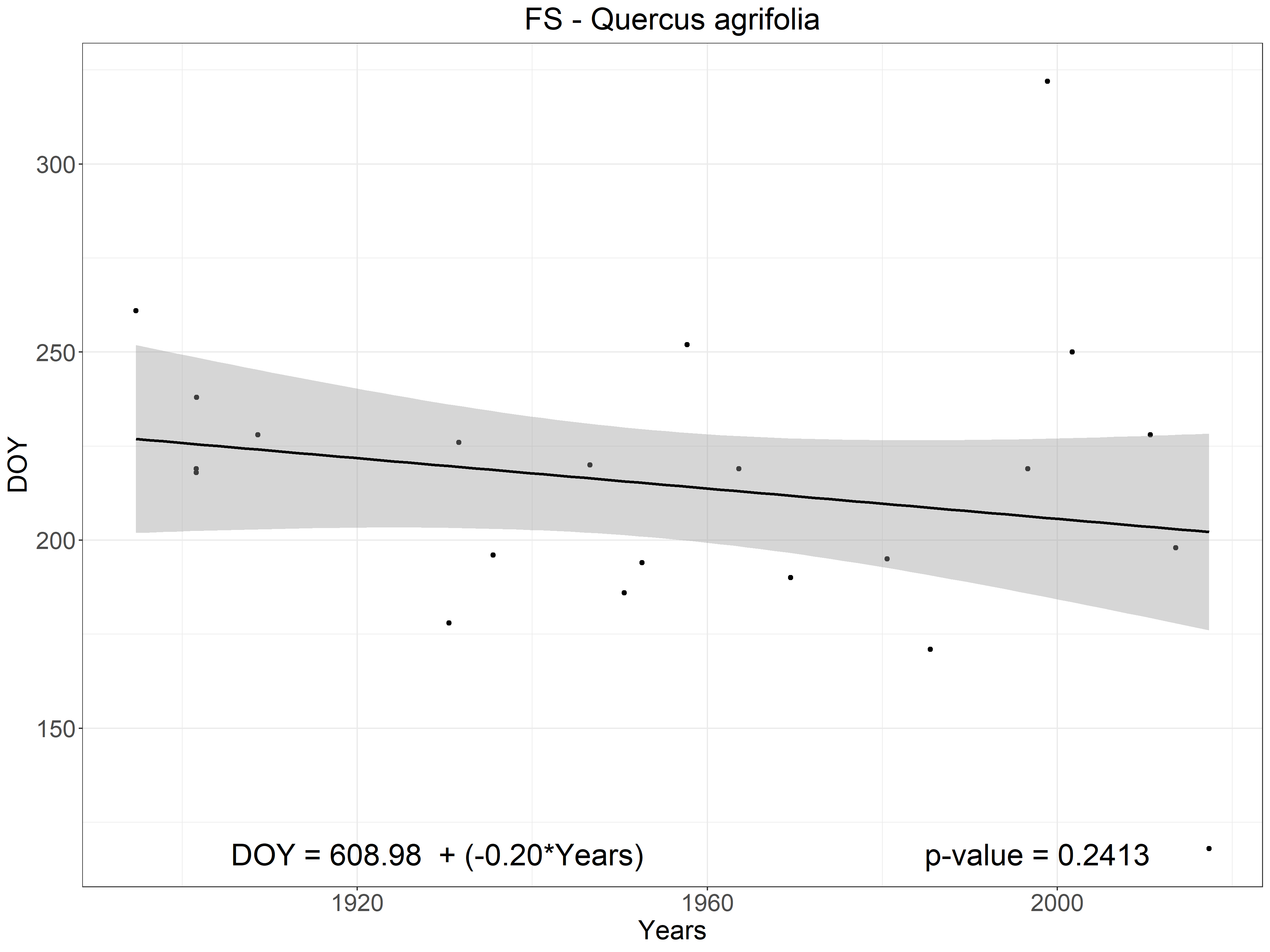

Supplement: Supplementary file 1 [file plants-14-00843-s001.zip › File S2-Species/S2.1-DOYvsYears/1_LM/Plots/FS_Quercus agrifolia_plot.png]

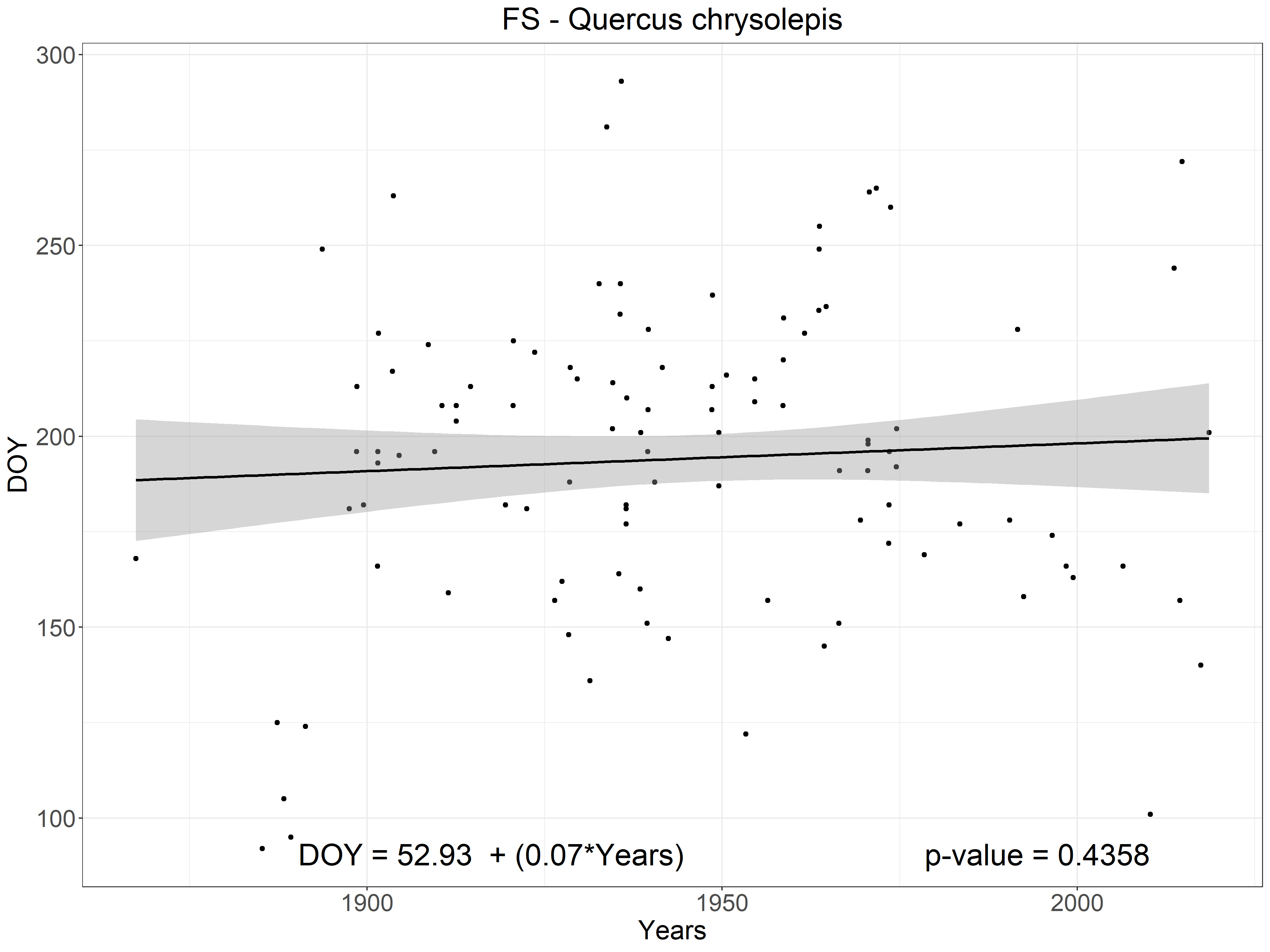

Supplement: Supplementary file 1 [file plants-14-00843-s001.zip › File S2-Species/S2.1-DOYvsYears/1_LM/Plots/FS_Quercus chrysolepis_plot.png]

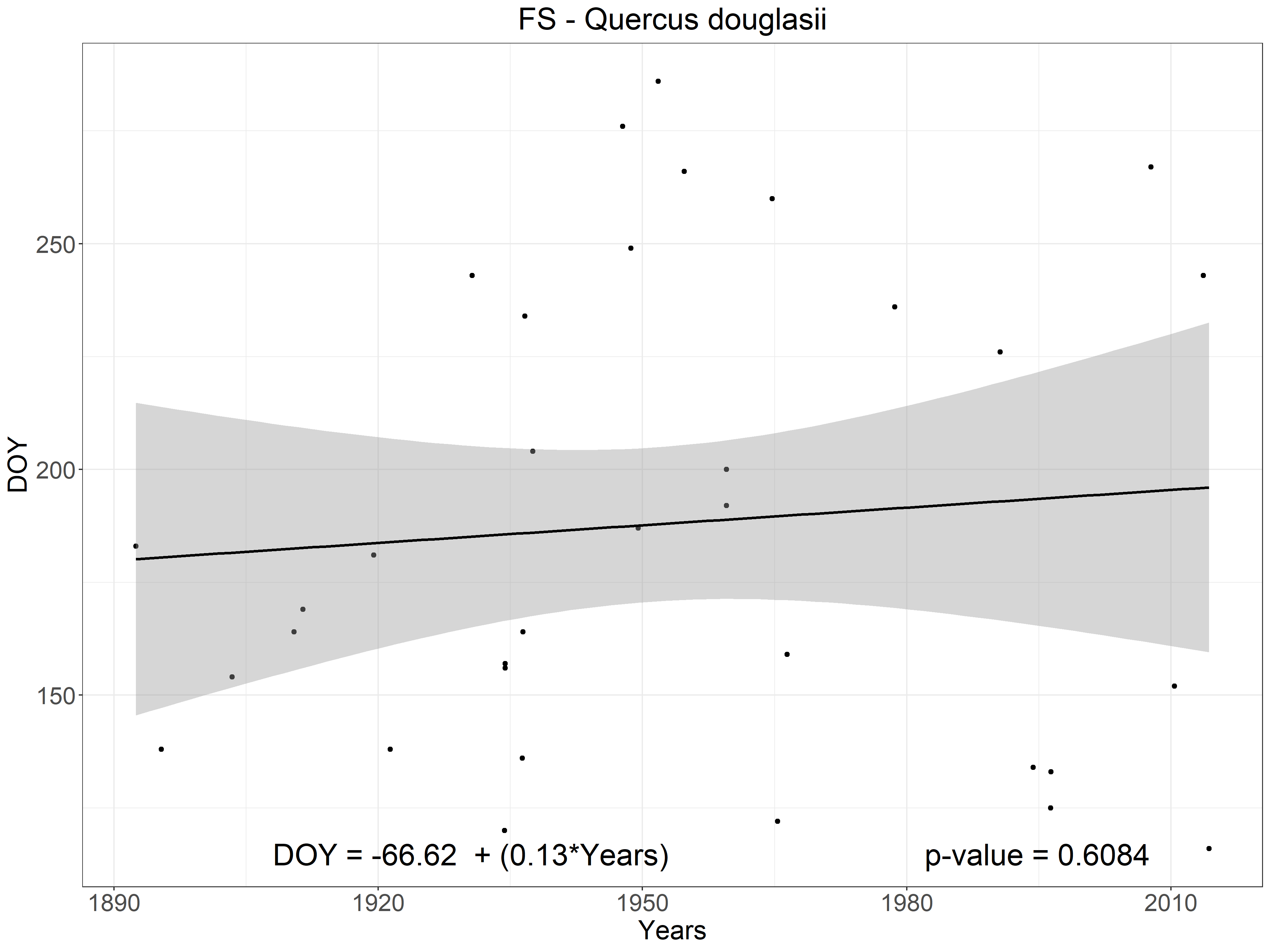

Supplement: Supplementary file 1 [file plants-14-00843-s001.zip › File S2-Species/S2.1-DOYvsYears/1_LM/Plots/FS_Quercus douglasii_plot.png]

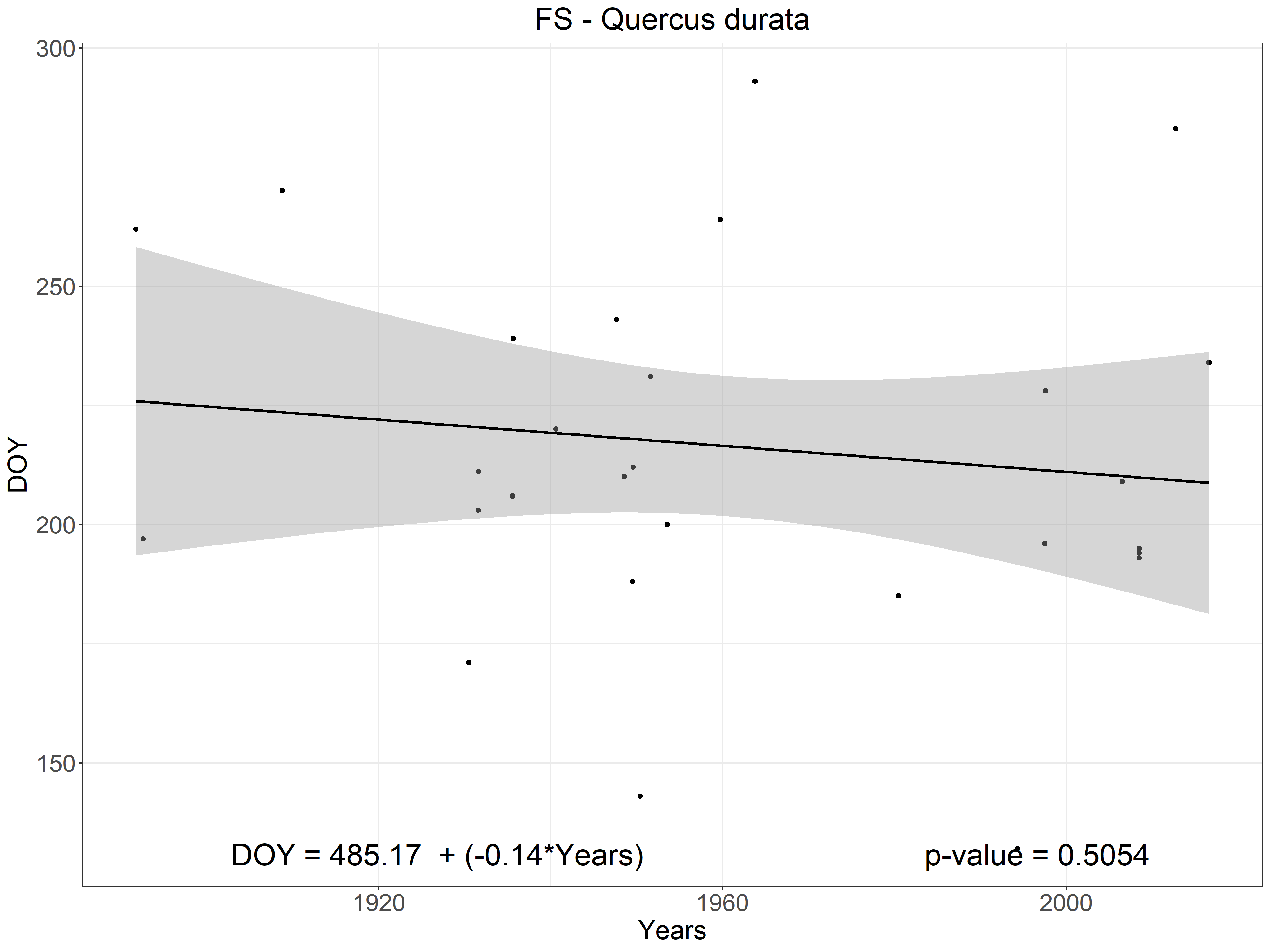

Supplement: Supplementary file 1 [file plants-14-00843-s001.zip › File S2-Species/S2.1-DOYvsYears/1_LM/Plots/FS_Quercus durata_plot.png]

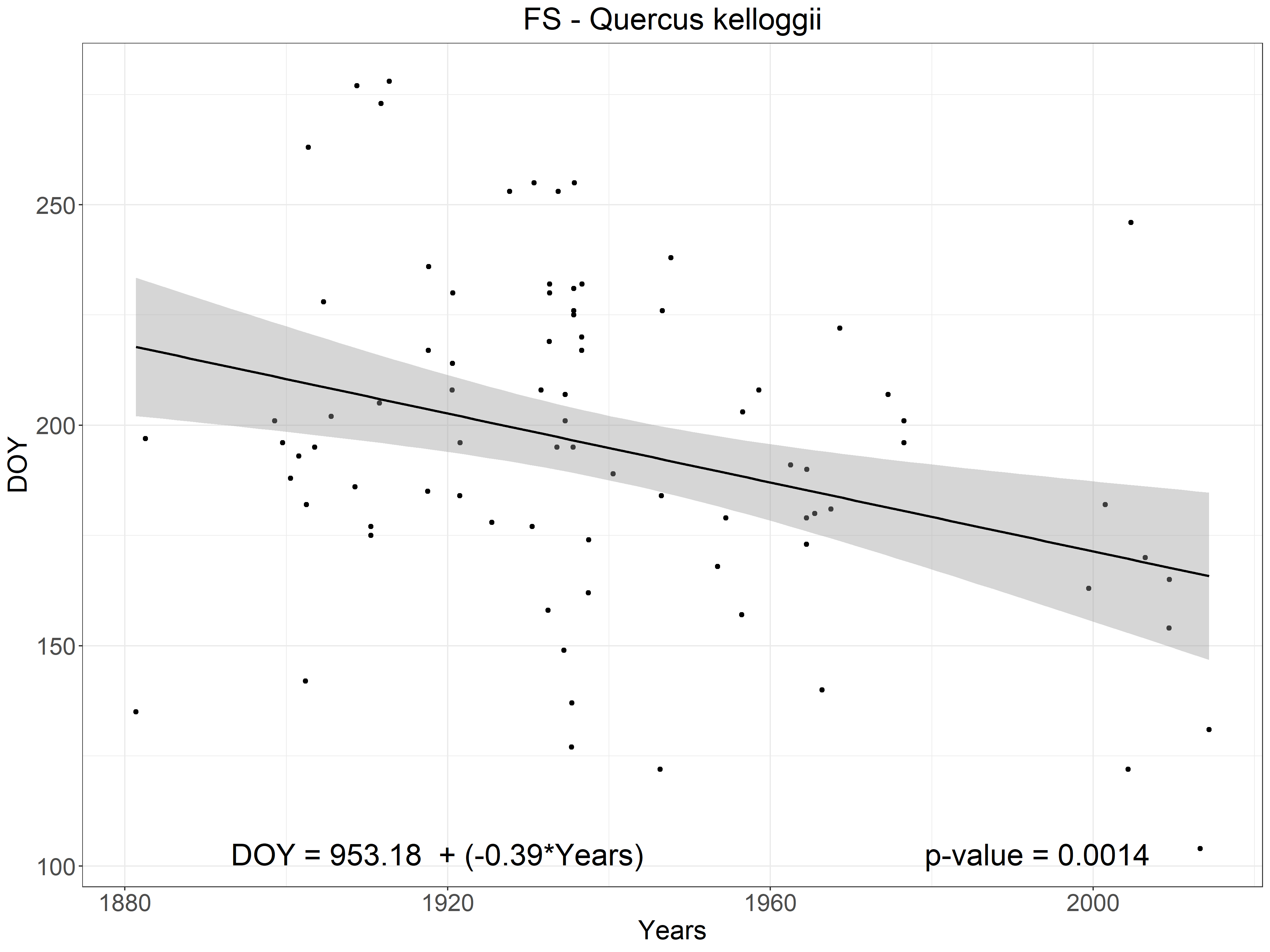

Supplement: Supplementary file 1 [file plants-14-00843-s001.zip › File S2-Species/S2.1-DOYvsYears/1_LM/Plots/FS_Quercus kelloggii_plot.png]

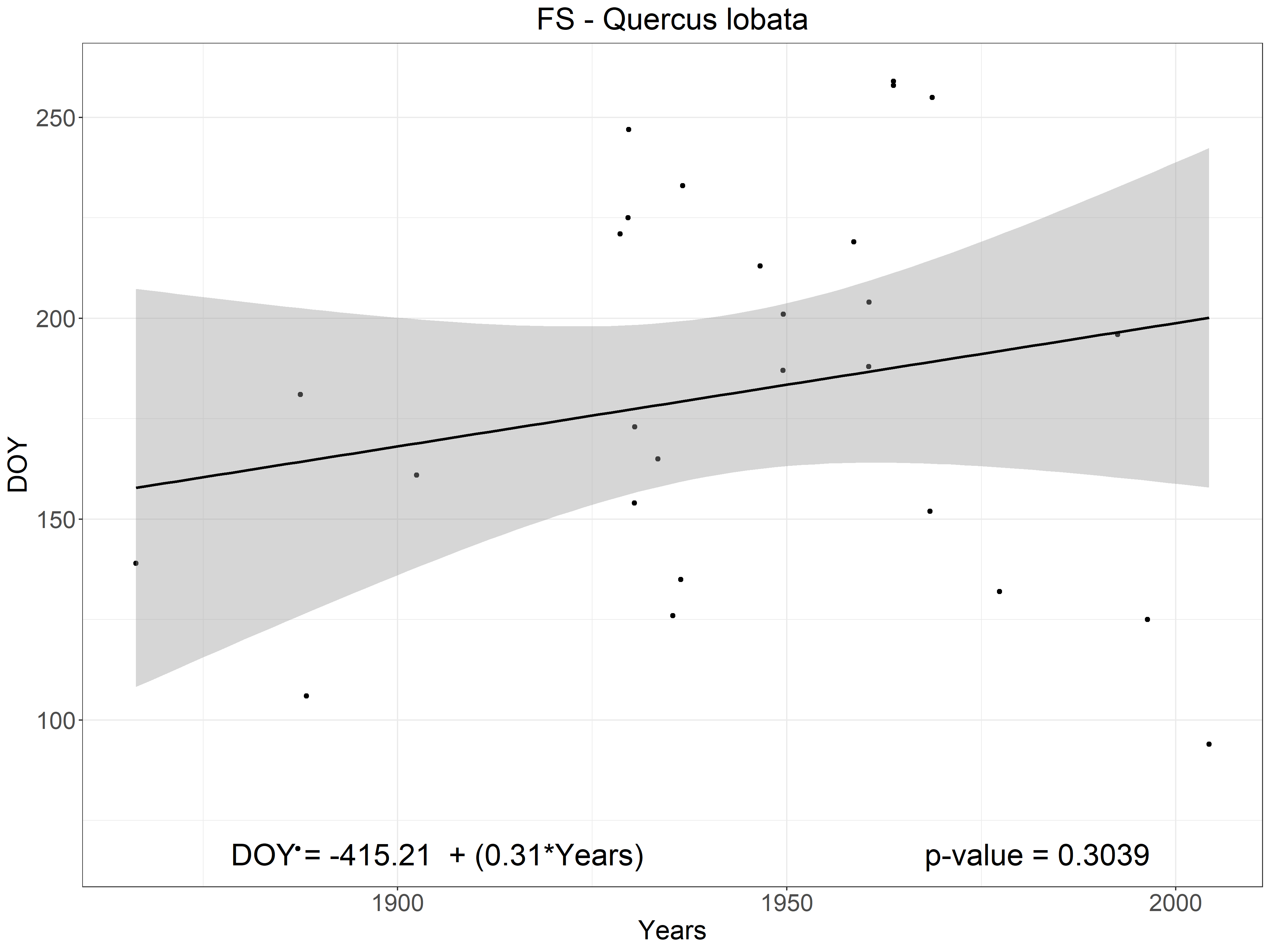

Supplement: Supplementary file 1 [file plants-14-00843-s001.zip › File S2-Species/S2.1-DOYvsYears/1_LM/Plots/FS_Quercus lobata_plot.png]

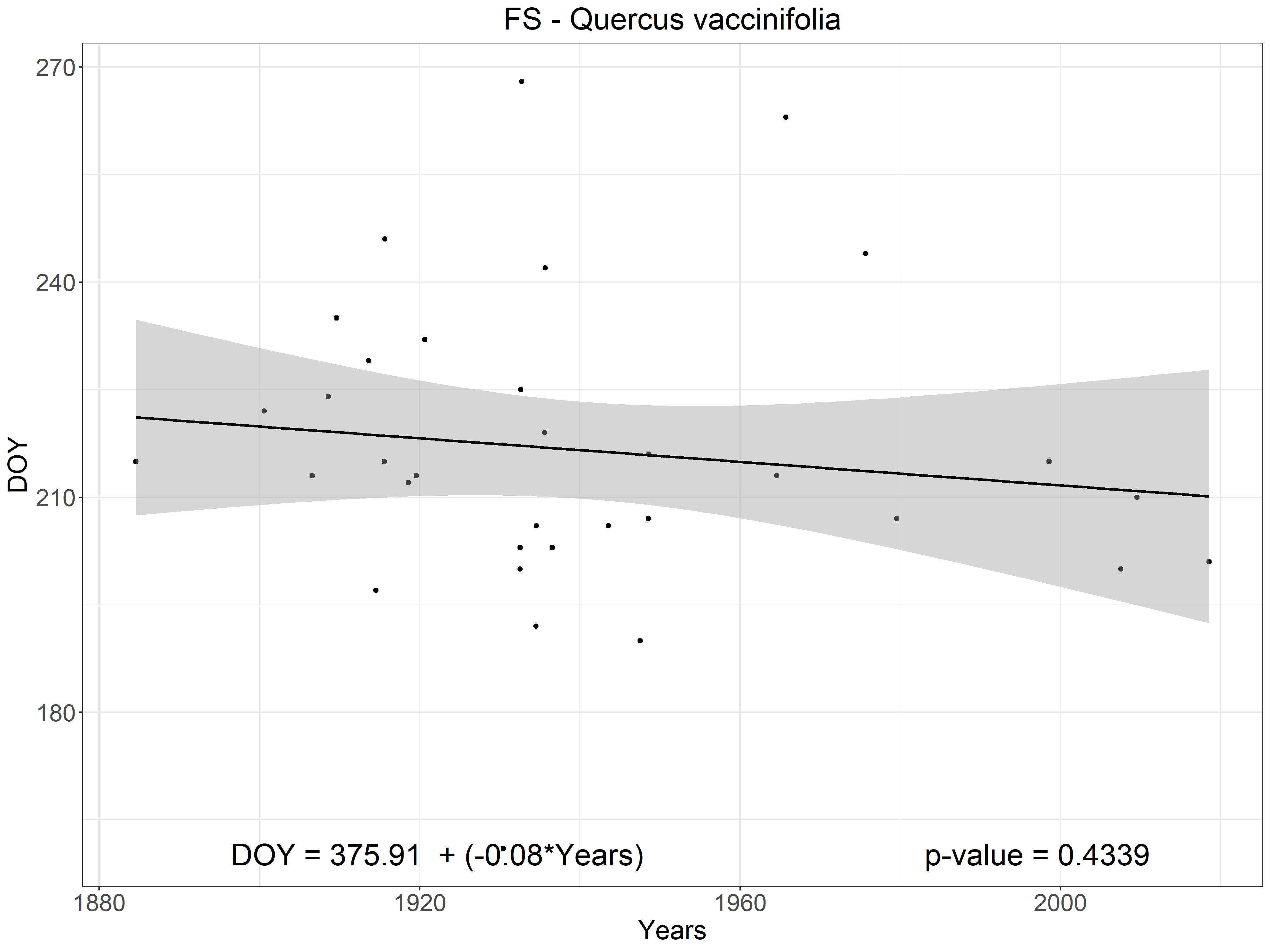

Supplement: Supplementary file 1 [file plants-14-00843-s001.zip › File S2-Species/S2.1-DOYvsYears/1_LM/Plots/FS_Quercus vaccinifolia_plot.png]

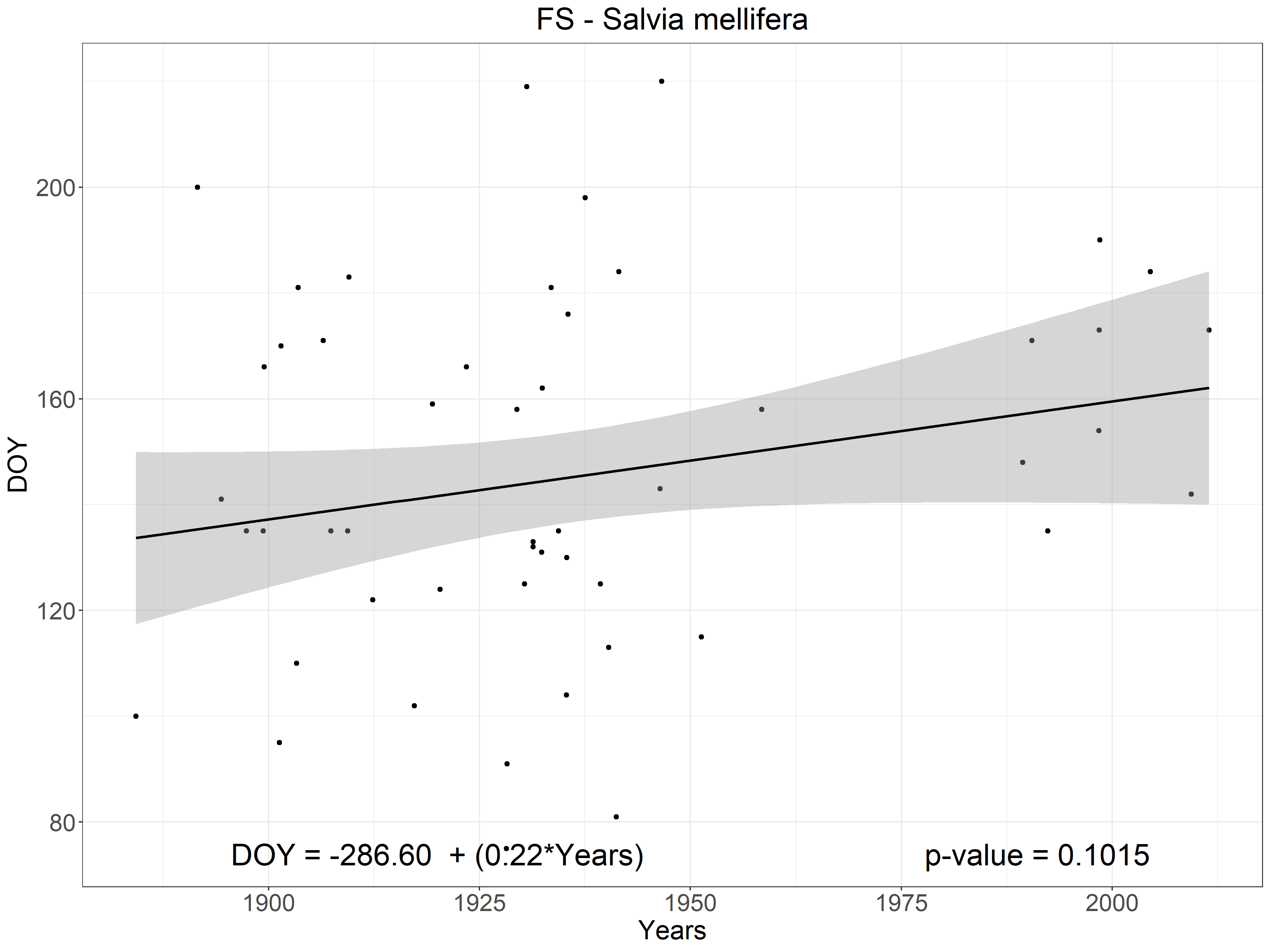

Supplement: Supplementary file 1 [file plants-14-00843-s001.zip › File S2-Species/S2.1-DOYvsYears/1_LM/Plots/FS_Salvia mellifera_plot.png]

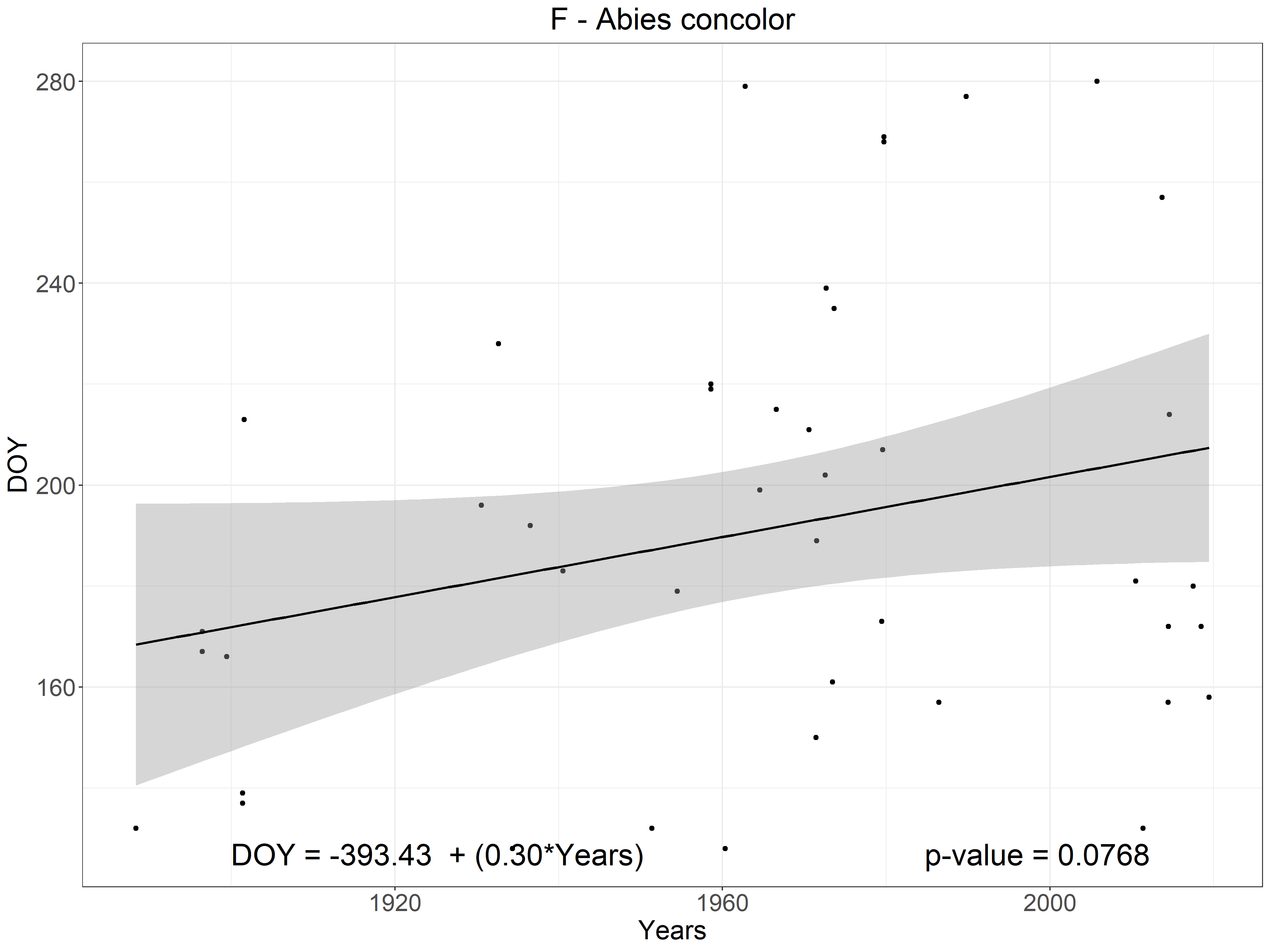

Supplement: Supplementary file 1 [file plants-14-00843-s001.zip › File S2-Species/S2.1-DOYvsYears/1_LM/Plots/F_Abies concolor_plot.png]

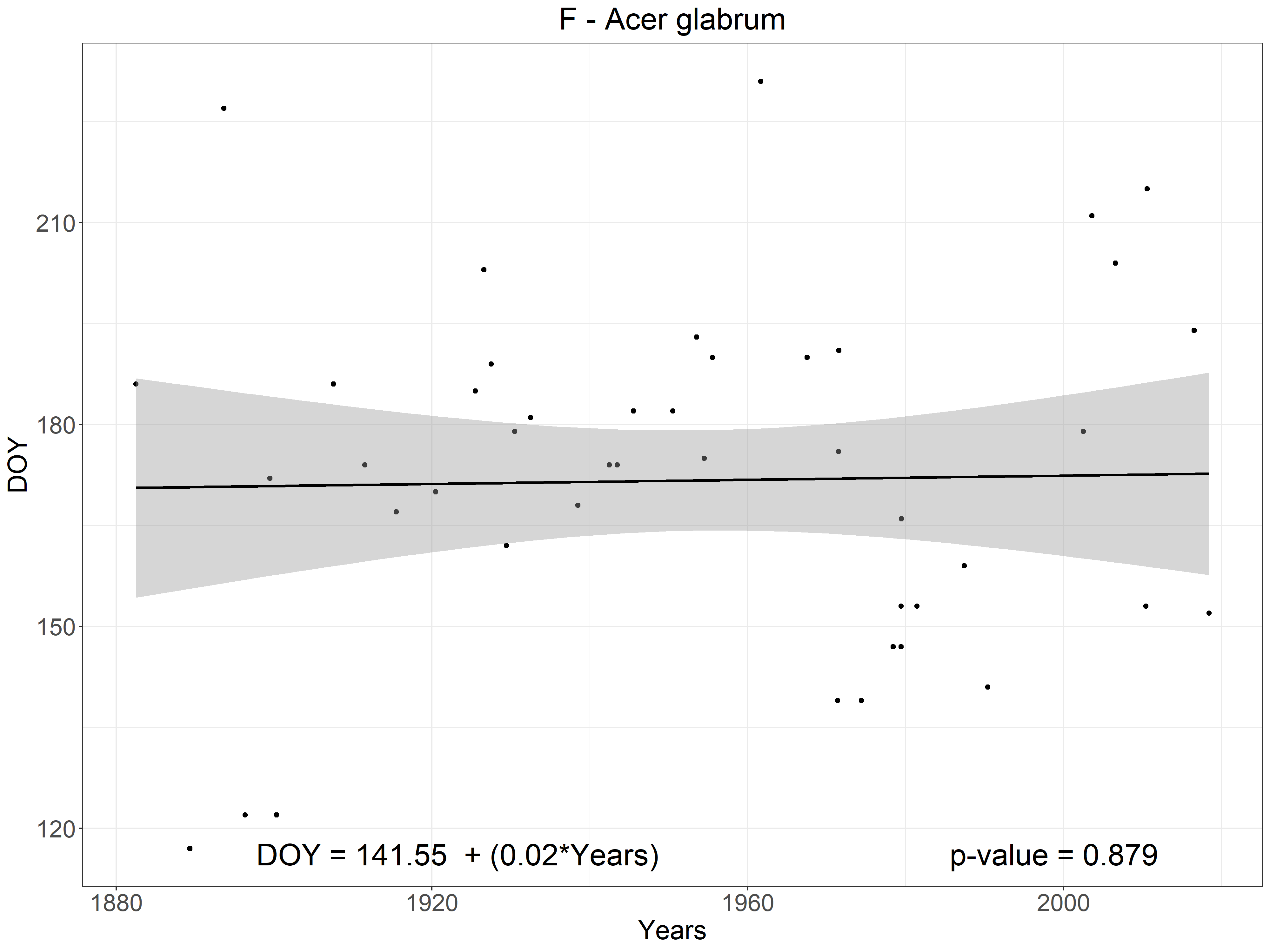

Supplement: Supplementary file 1 [file plants-14-00843-s001.zip › File S2-Species/S2.1-DOYvsYears/1_LM/Plots/F_Acer glabrum_plot.png]

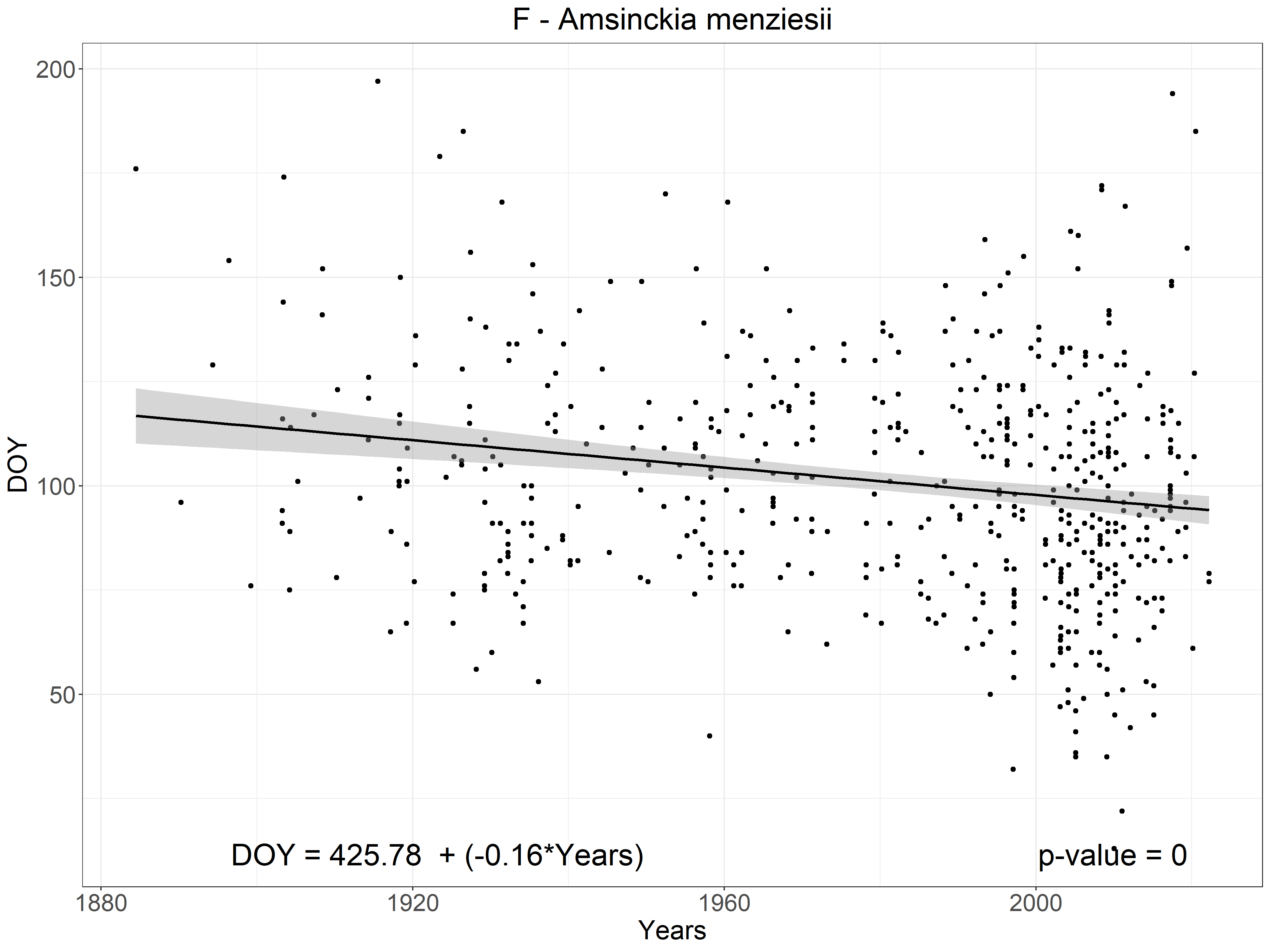

Supplement: Supplementary file 1 [file plants-14-00843-s001.zip › File S2-Species/S2.1-DOYvsYears/1_LM/Plots/F_Amsinckia menziesii_plot.png]

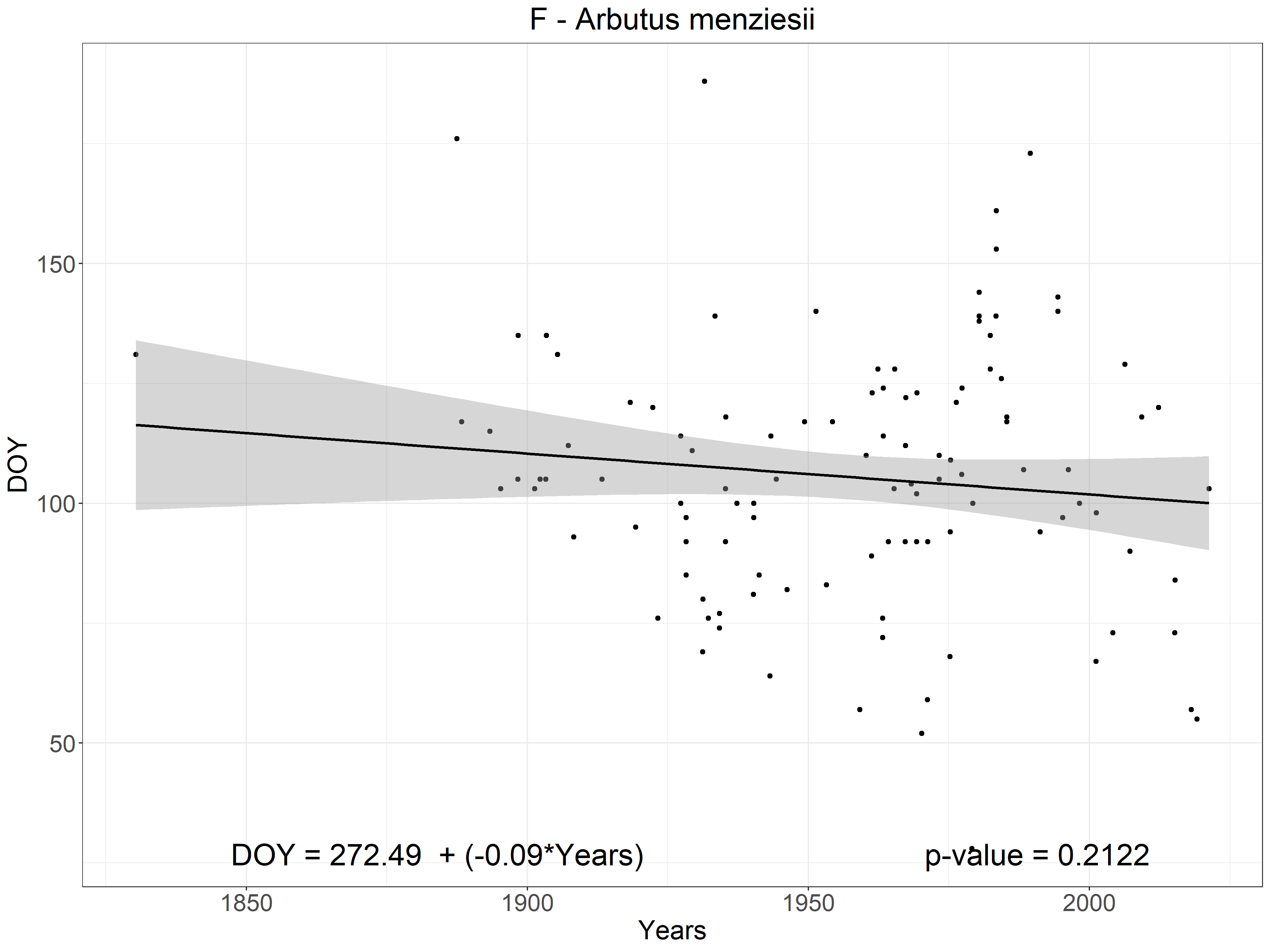

Supplement: Supplementary file 1 [file plants-14-00843-s001.zip › File S2-Species/S2.1-DOYvsYears/1_LM/Plots/F_Arbutus menziesii_plot.png]

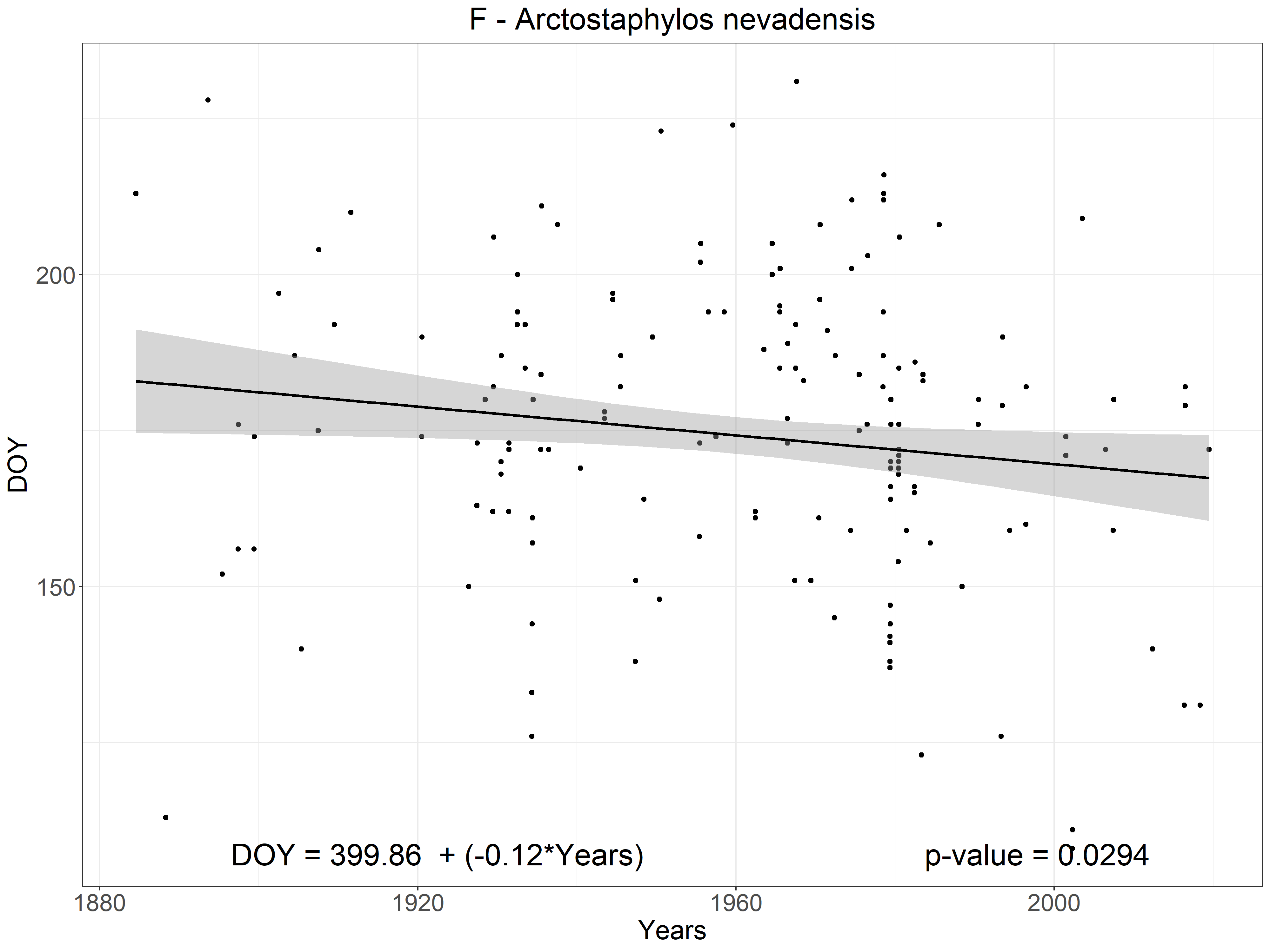

Supplement: Supplementary file 1 [file plants-14-00843-s001.zip › File S2-Species/S2.1-DOYvsYears/1_LM/Plots/F_Arctostaphylos nevadensis_plot.png]

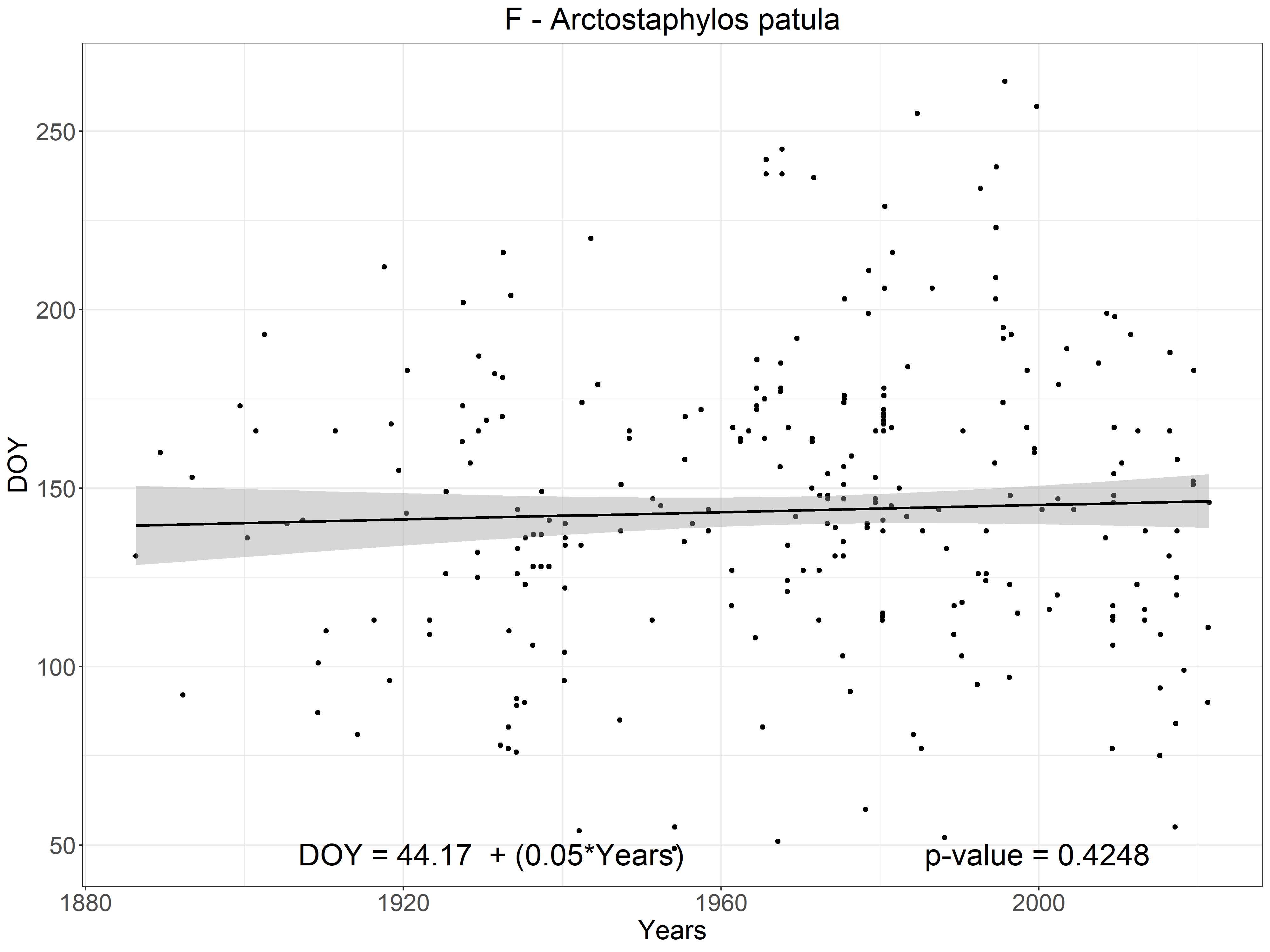

Supplement: Supplementary file 1 [file plants-14-00843-s001.zip › File S2-Species/S2.1-DOYvsYears/1_LM/Plots/F_Arctostaphylos patula_plot.png]

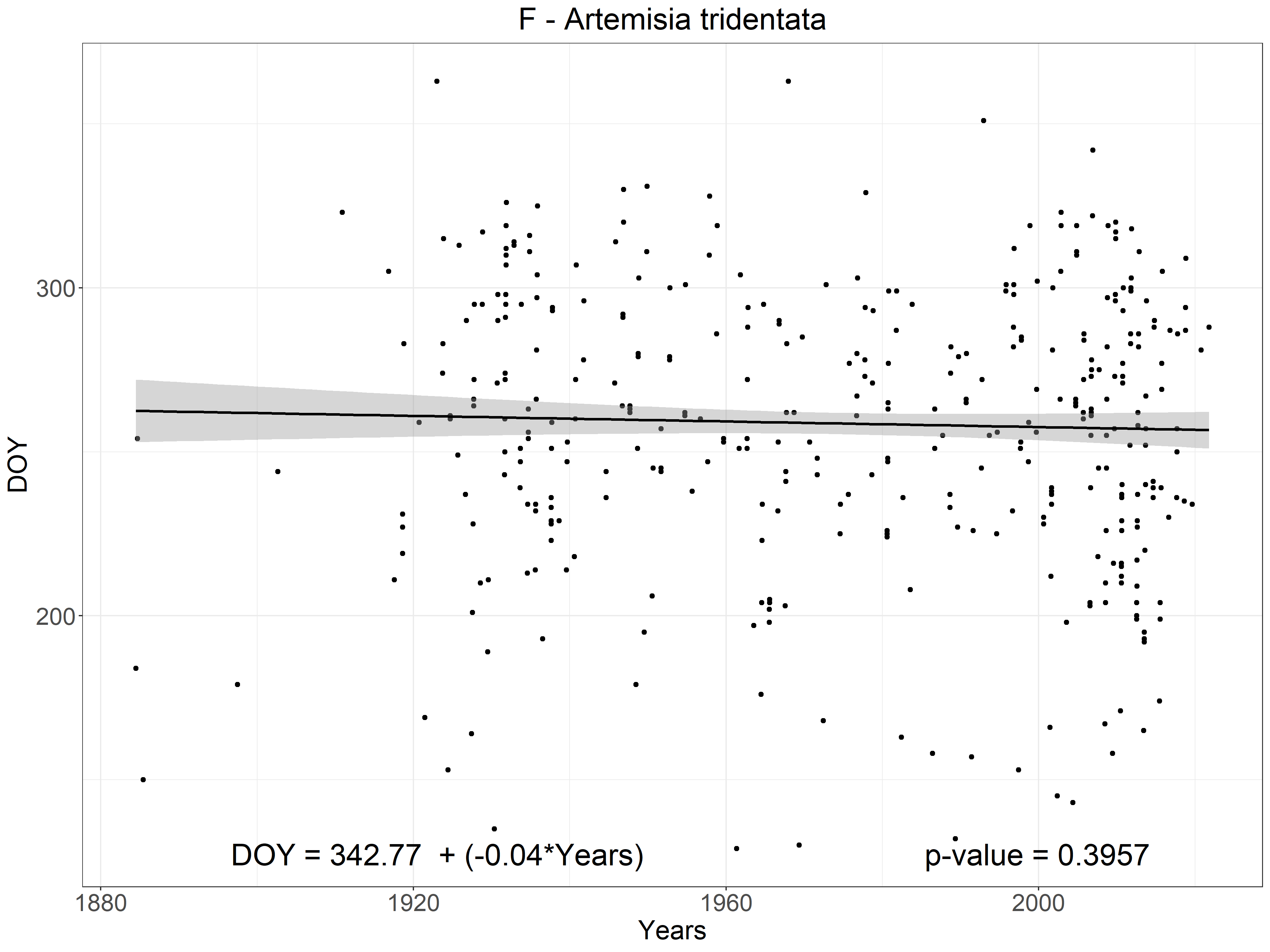

Supplement: Supplementary file 1 [file plants-14-00843-s001.zip › File S2-Species/S2.1-DOYvsYears/1_LM/Plots/F_Artemisia tridentata_plot.png]

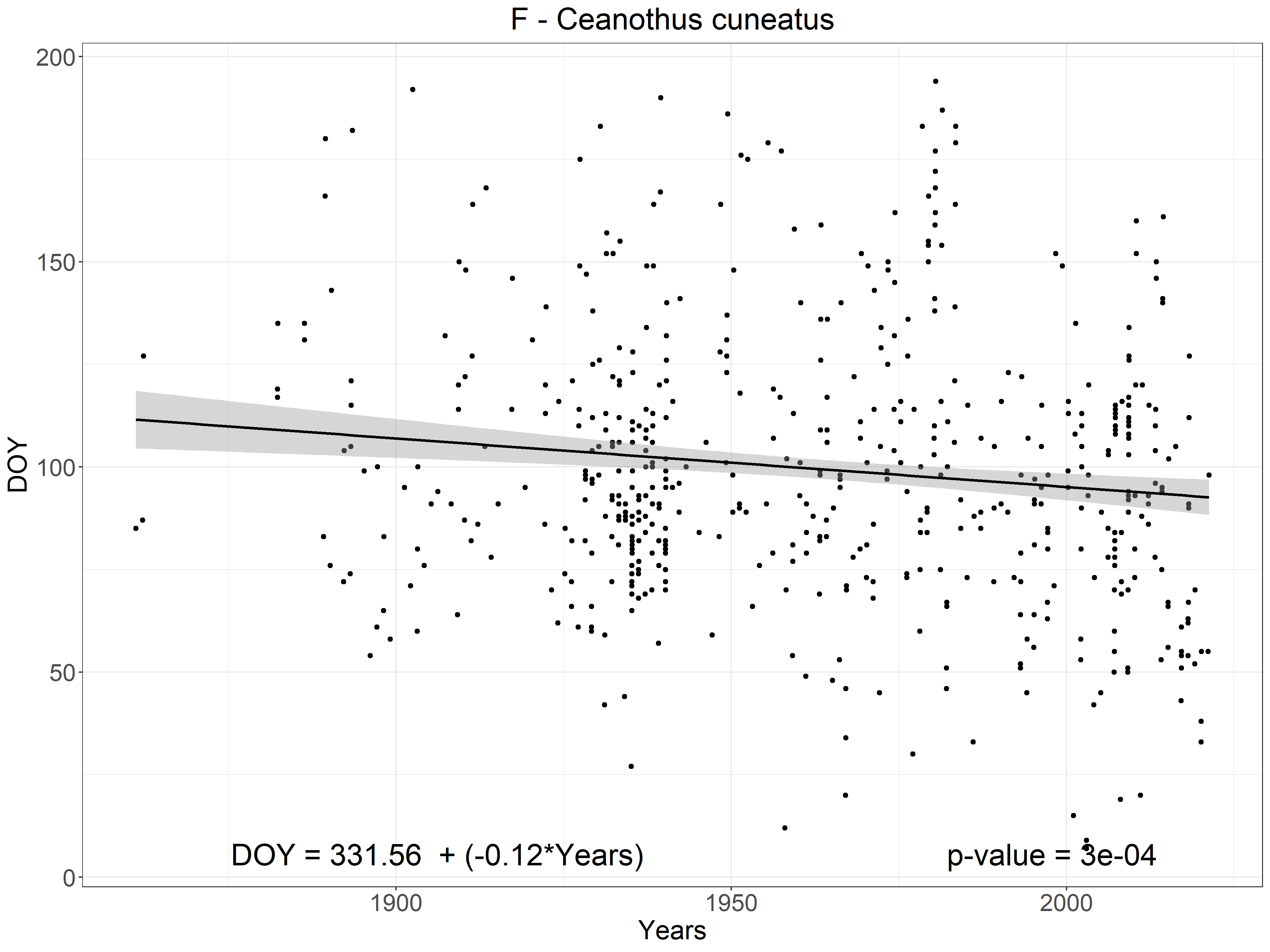

Supplement: Supplementary file 1 [file plants-14-00843-s001.zip › File S2-Species/S2.1-DOYvsYears/1_LM/Plots/F_Ceanothus cuneatus_plot.png]

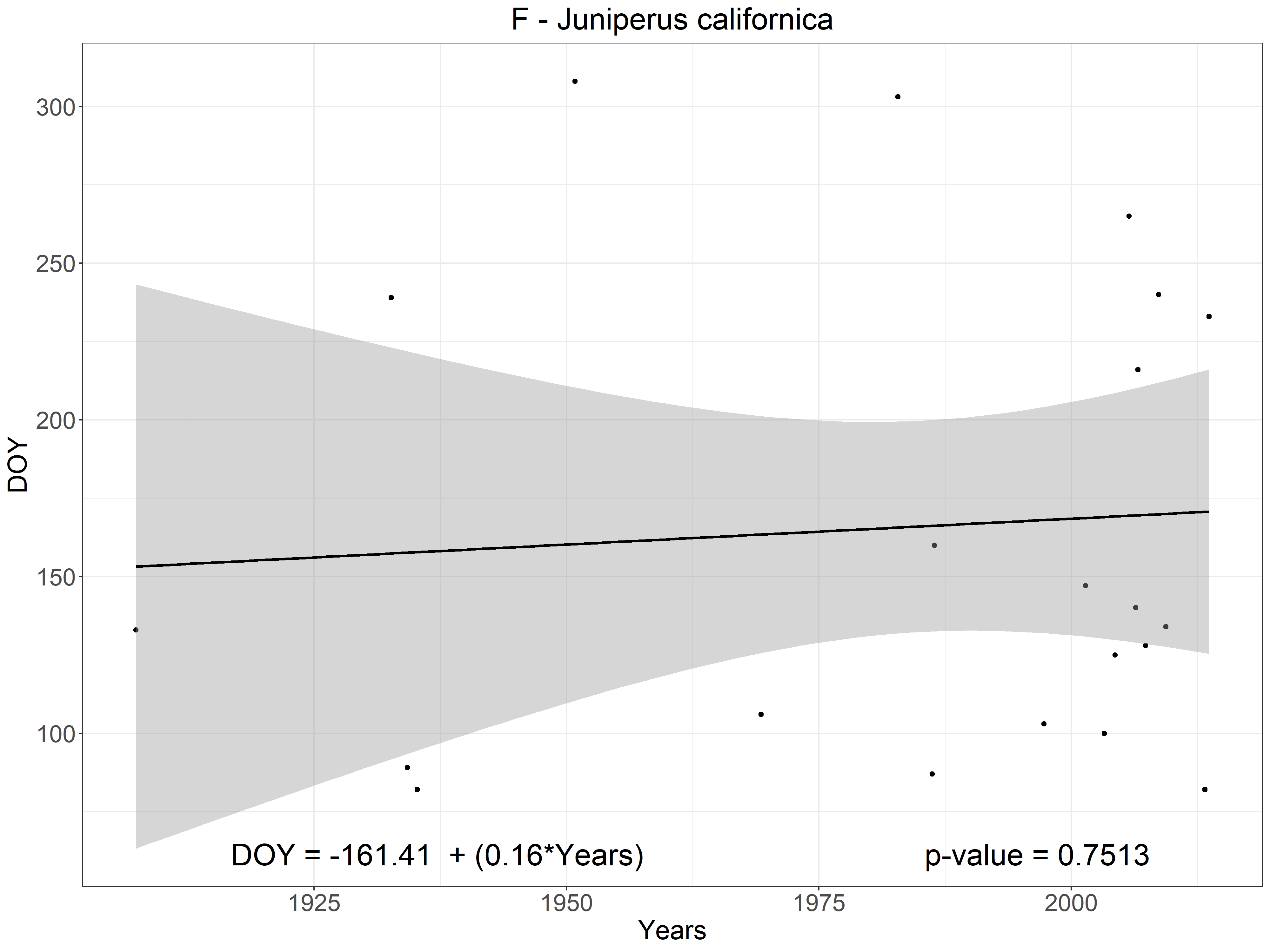

Supplement: Supplementary file 1 [file plants-14-00843-s001.zip › File S2-Species/S2.1-DOYvsYears/1_LM/Plots/F_Juniperus californica_plot.png]

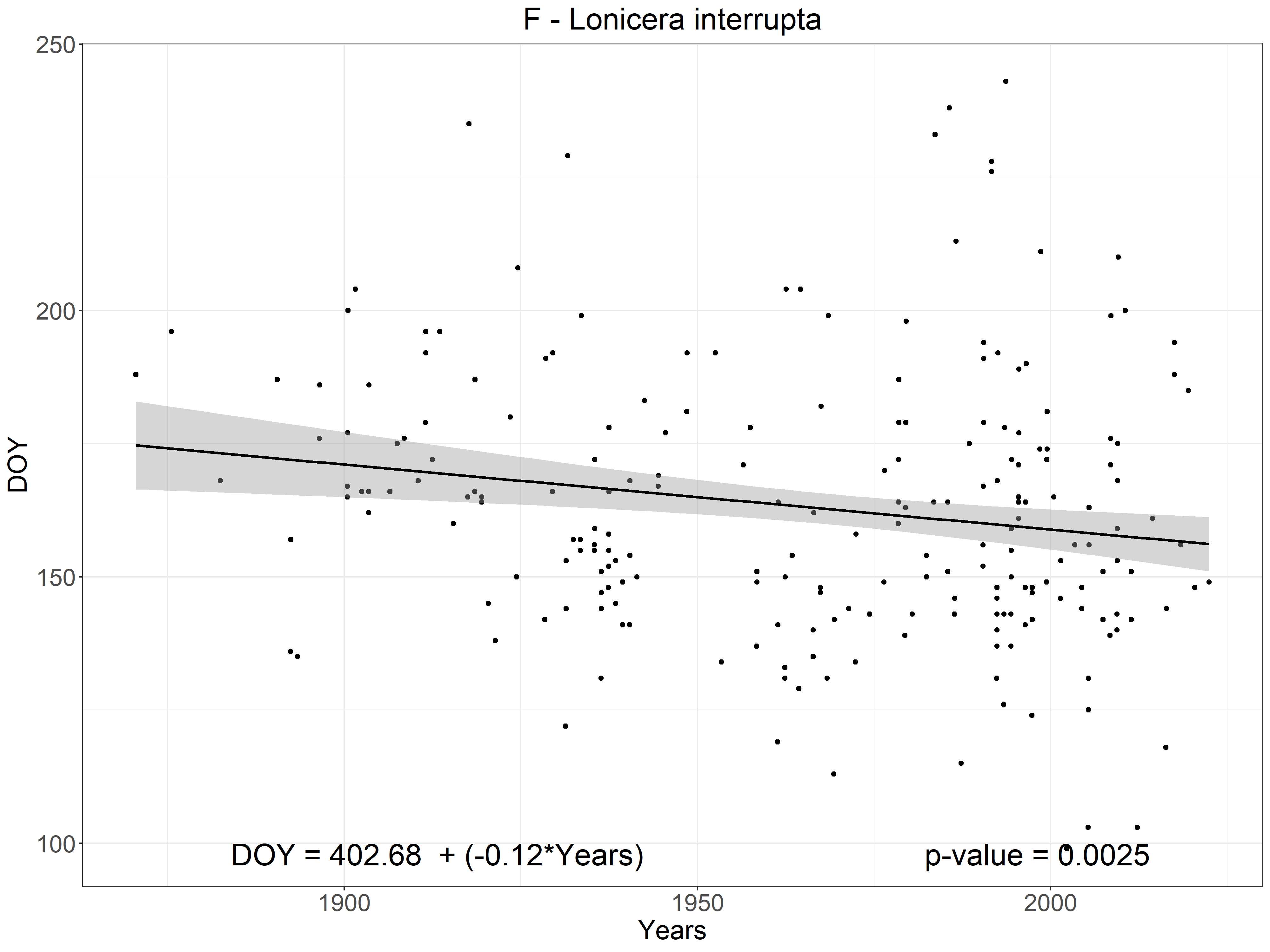

Supplement: Supplementary file 1 [file plants-14-00843-s001.zip › File S2-Species/S2.1-DOYvsYears/1_LM/Plots/F_Lonicera interrupta_plot.png]

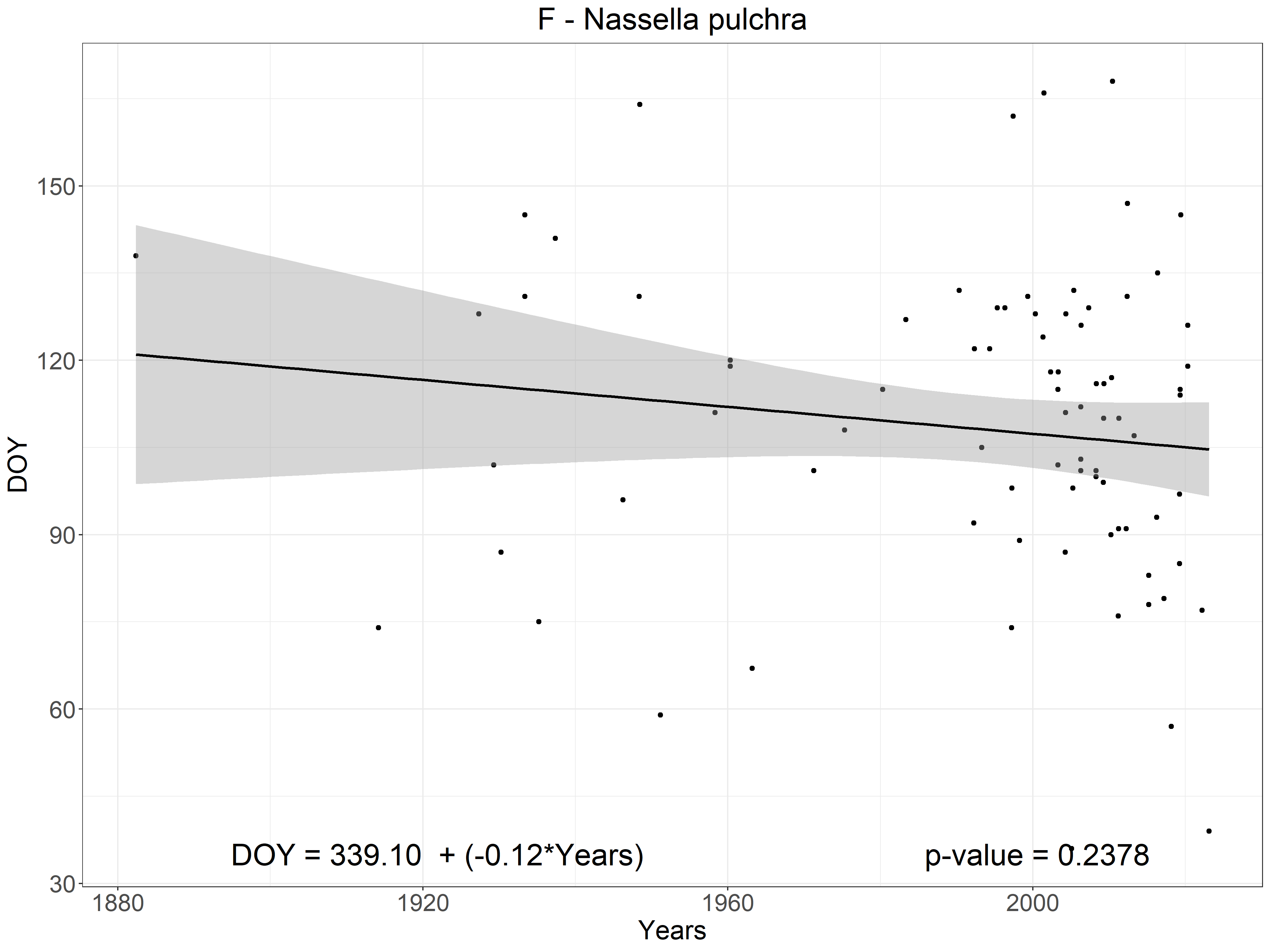

Supplement: Supplementary file 1 [file plants-14-00843-s001.zip › File S2-Species/S2.1-DOYvsYears/1_LM/Plots/F_Nassella pulchra_plot.png]

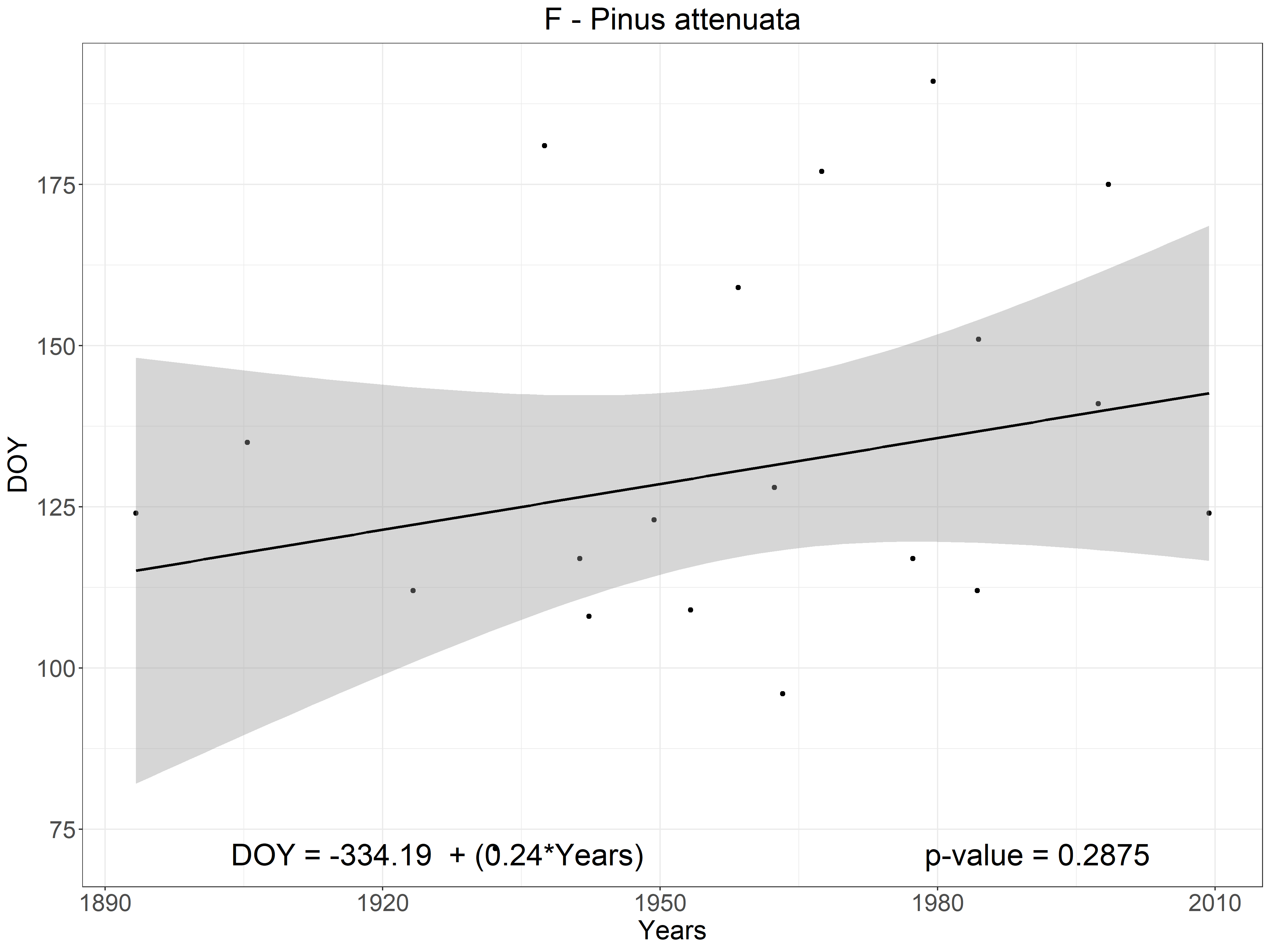

Supplement: Supplementary file 1 [file plants-14-00843-s001.zip › File S2-Species/S2.1-DOYvsYears/1_LM/Plots/F_Pinus attenuata_plot.png]

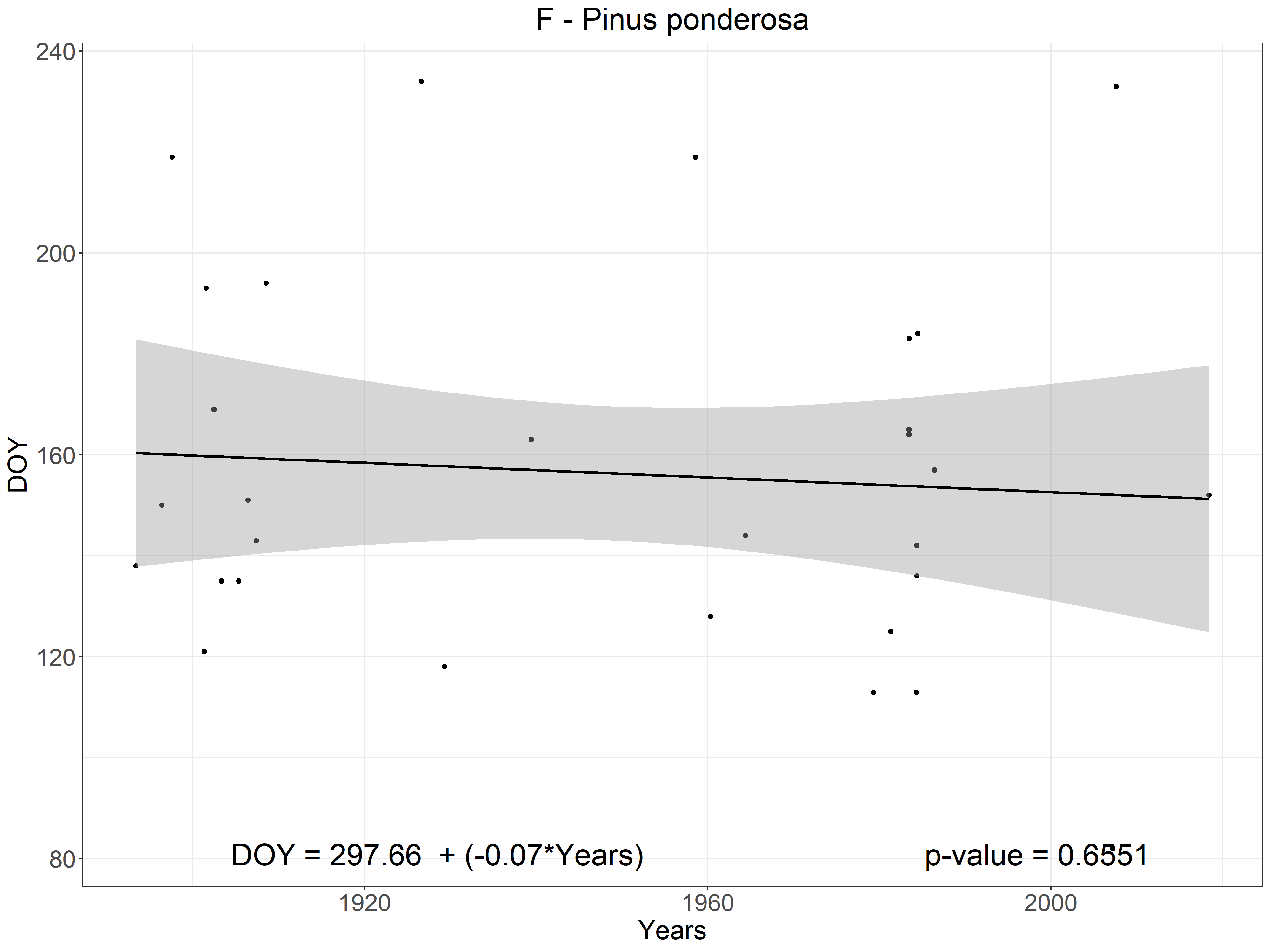

Supplement: Supplementary file 1 [file plants-14-00843-s001.zip › File S2-Species/S2.1-DOYvsYears/1_LM/Plots/F_Pinus ponderosa_plot.png]

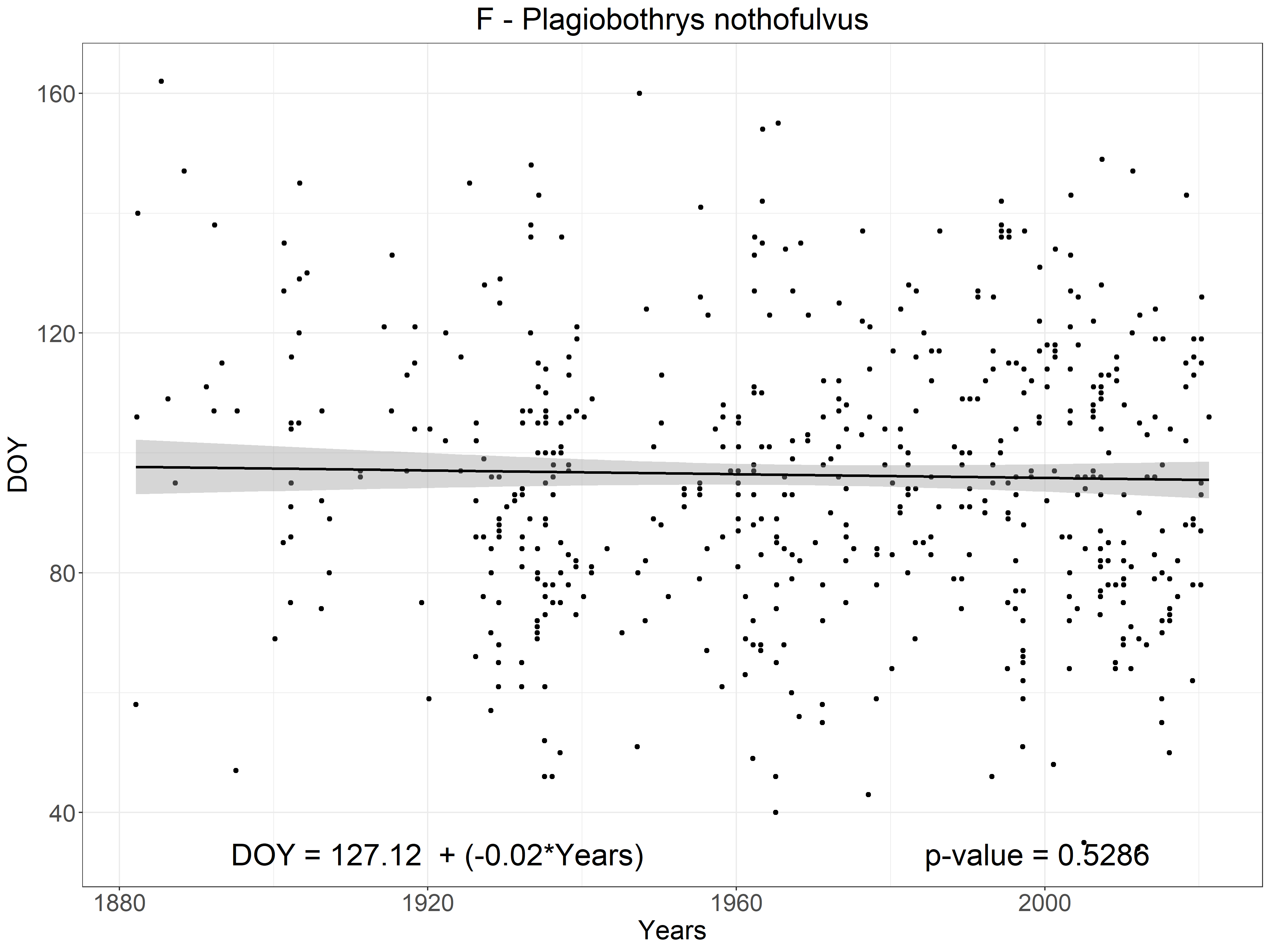

Supplement: Supplementary file 1 [file plants-14-00843-s001.zip › File S2-Species/S2.1-DOYvsYears/1_LM/Plots/F_Plagiobothrys nothofulvus_plot.png]

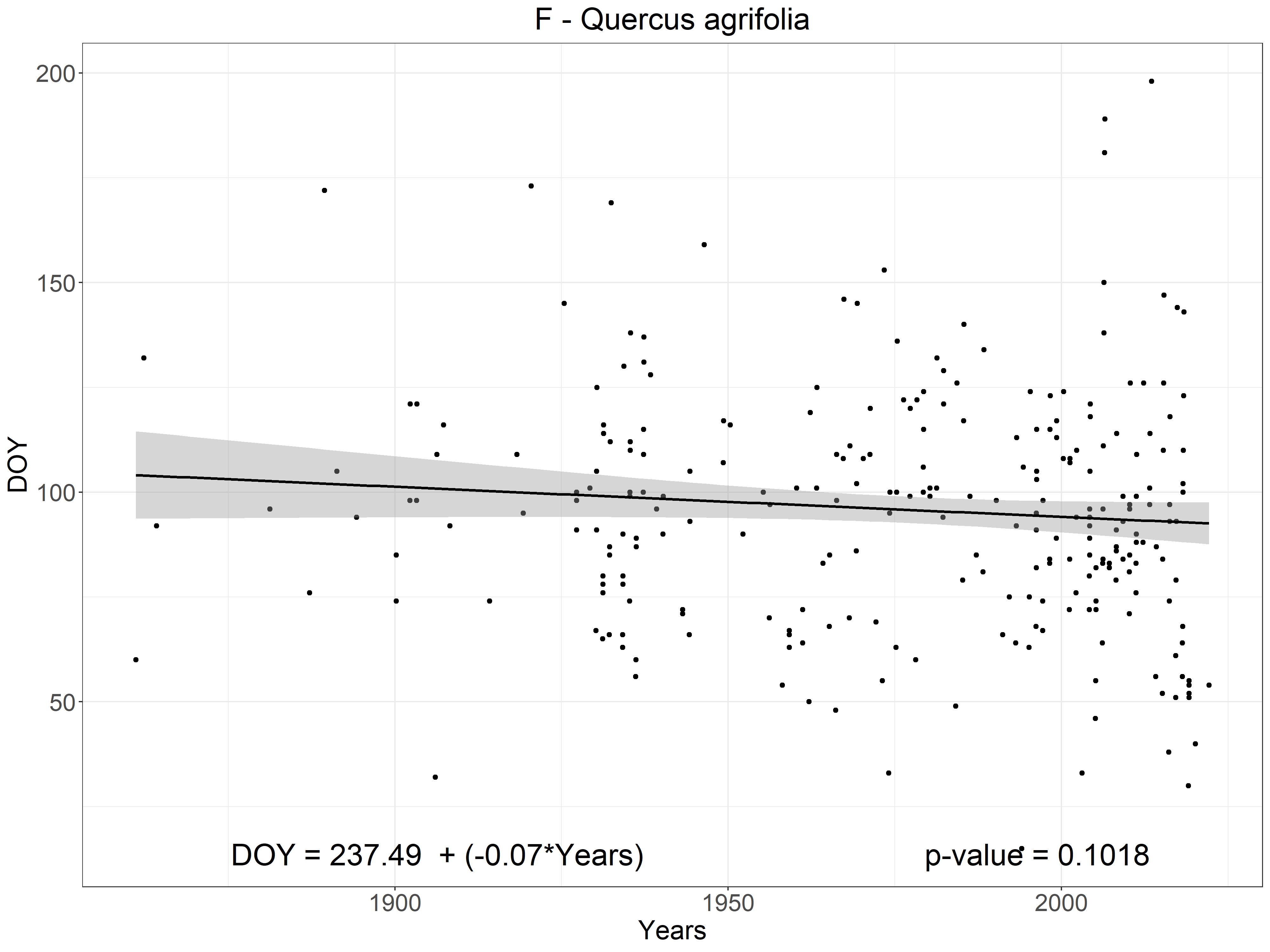

Supplement: Supplementary file 1 [file plants-14-00843-s001.zip › File S2-Species/S2.1-DOYvsYears/1_LM/Plots/F_Quercus agrifolia_plot.png]

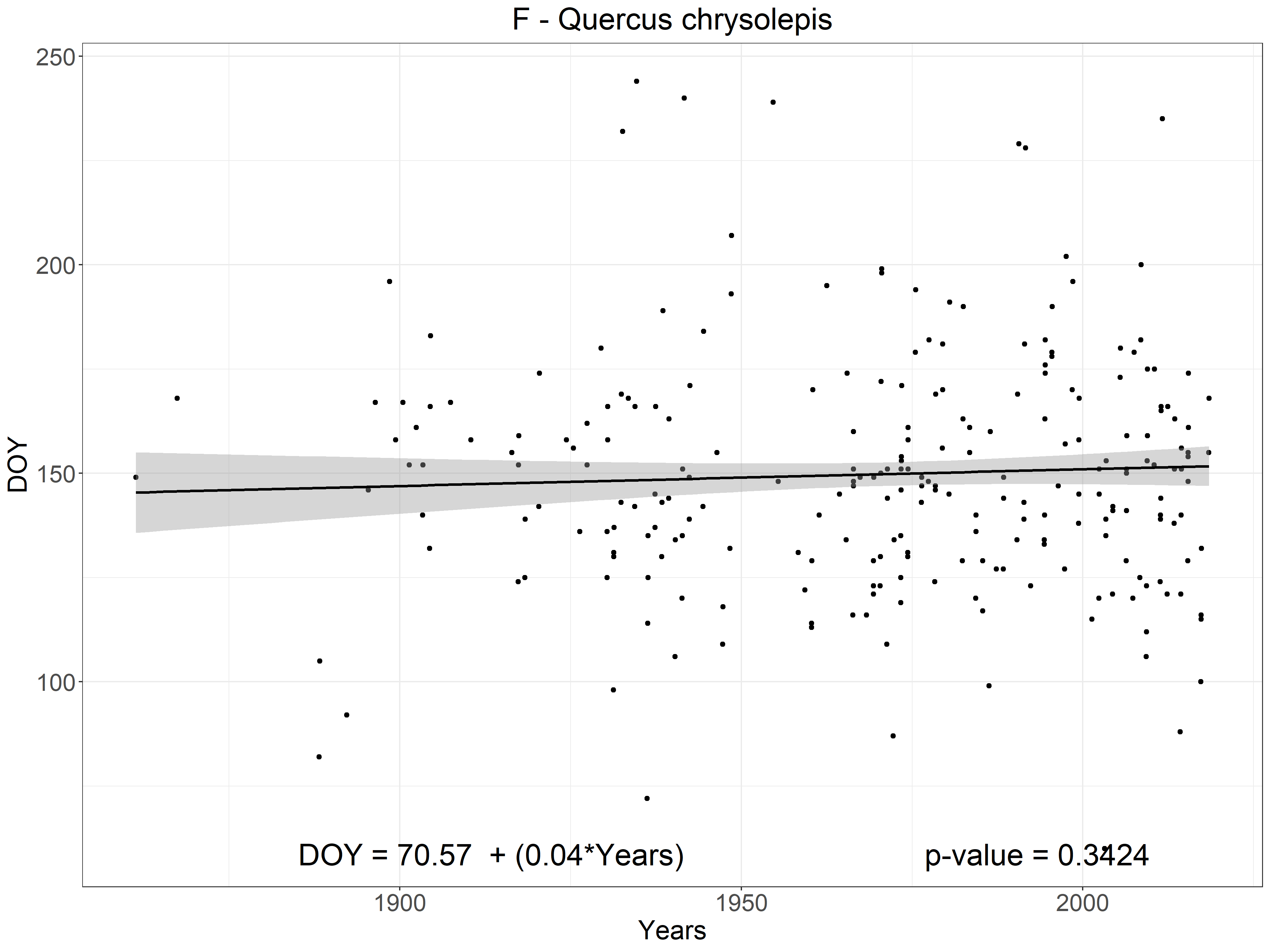

Supplement: Supplementary file 1 [file plants-14-00843-s001.zip › File S2-Species/S2.1-DOYvsYears/1_LM/Plots/F_Quercus chrysolepis_plot.png]

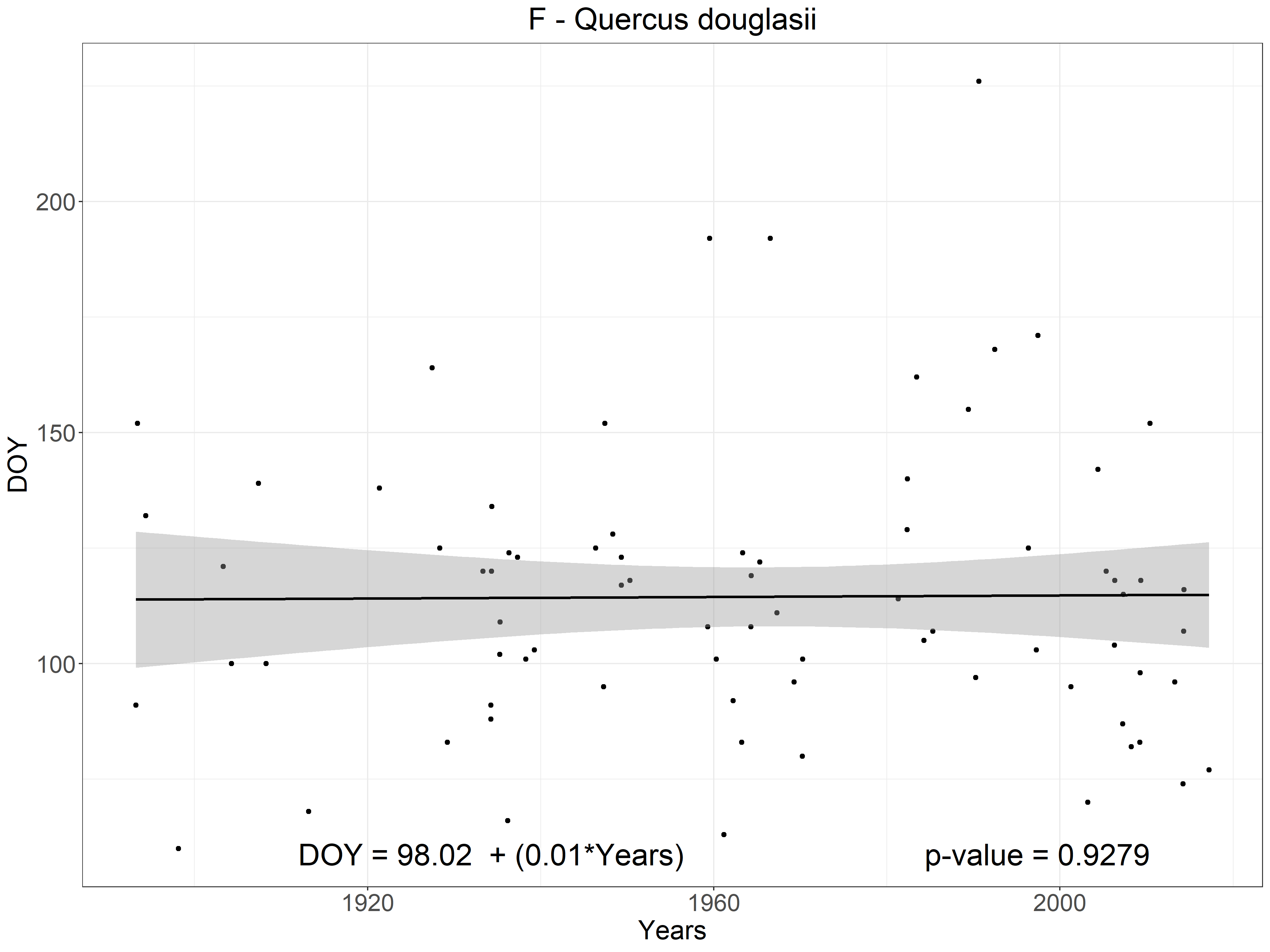

Supplement: Supplementary file 1 [file plants-14-00843-s001.zip › File S2-Species/S2.1-DOYvsYears/1_LM/Plots/F_Quercus douglasii_plot.png]

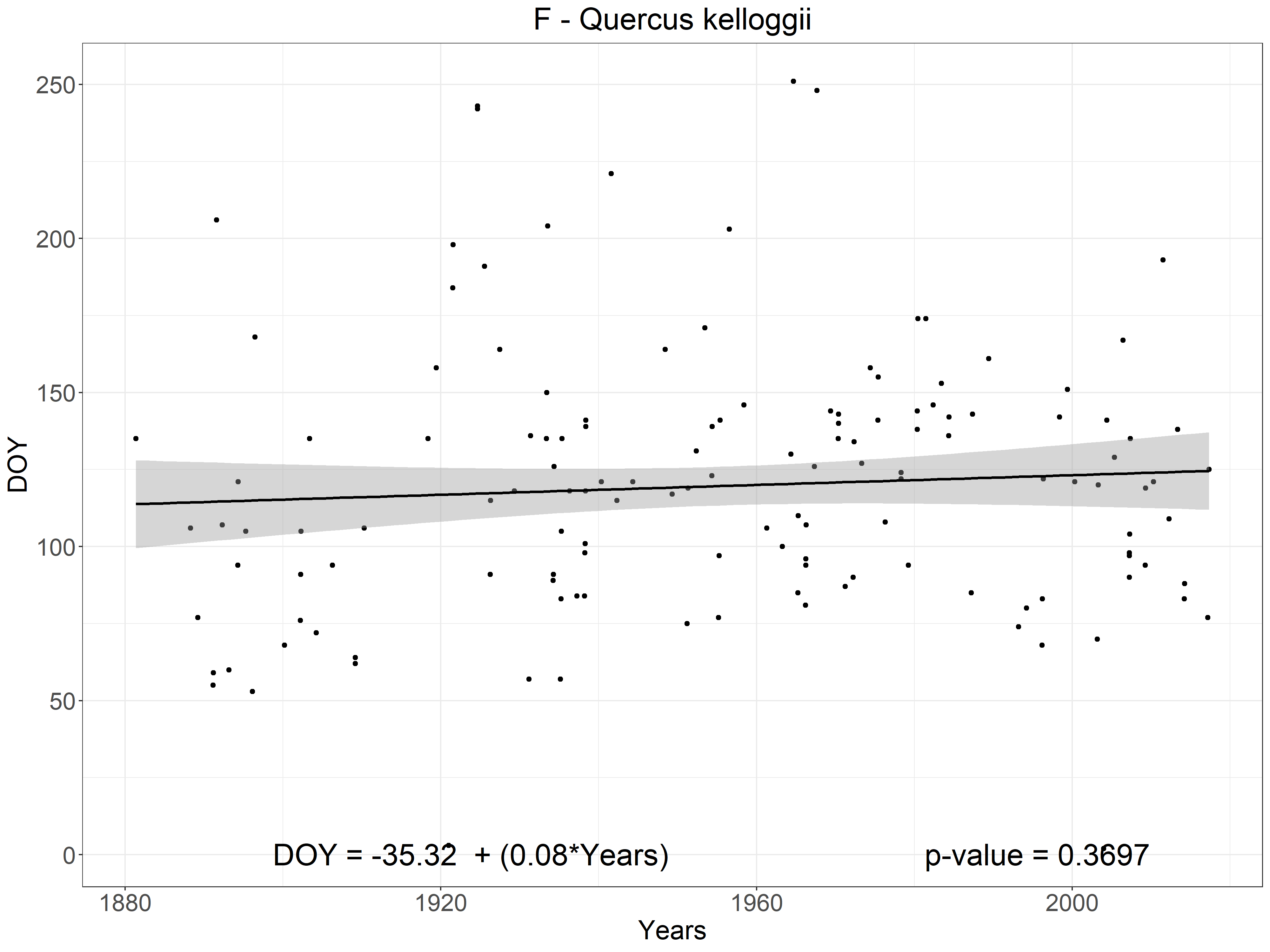

Supplement: Supplementary file 1 [file plants-14-00843-s001.zip › File S2-Species/S2.1-DOYvsYears/1_LM/Plots/F_Quercus kelloggii_plot.png]

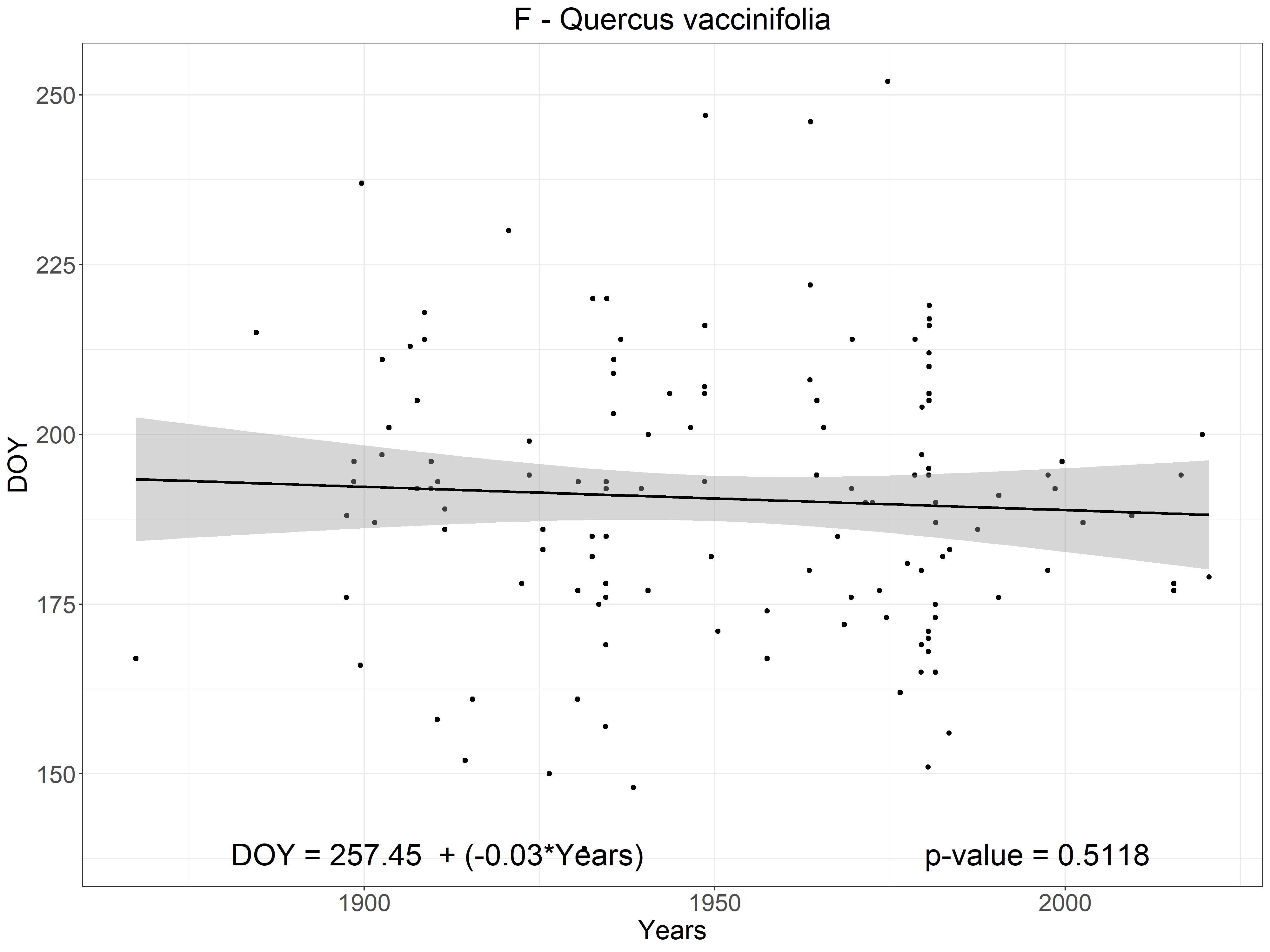

Supplement: Supplementary file 1 [file plants-14-00843-s001.zip › File S2-Species/S2.1-DOYvsYears/1_LM/Plots/F_Quercus vaccinifolia_plot.png]

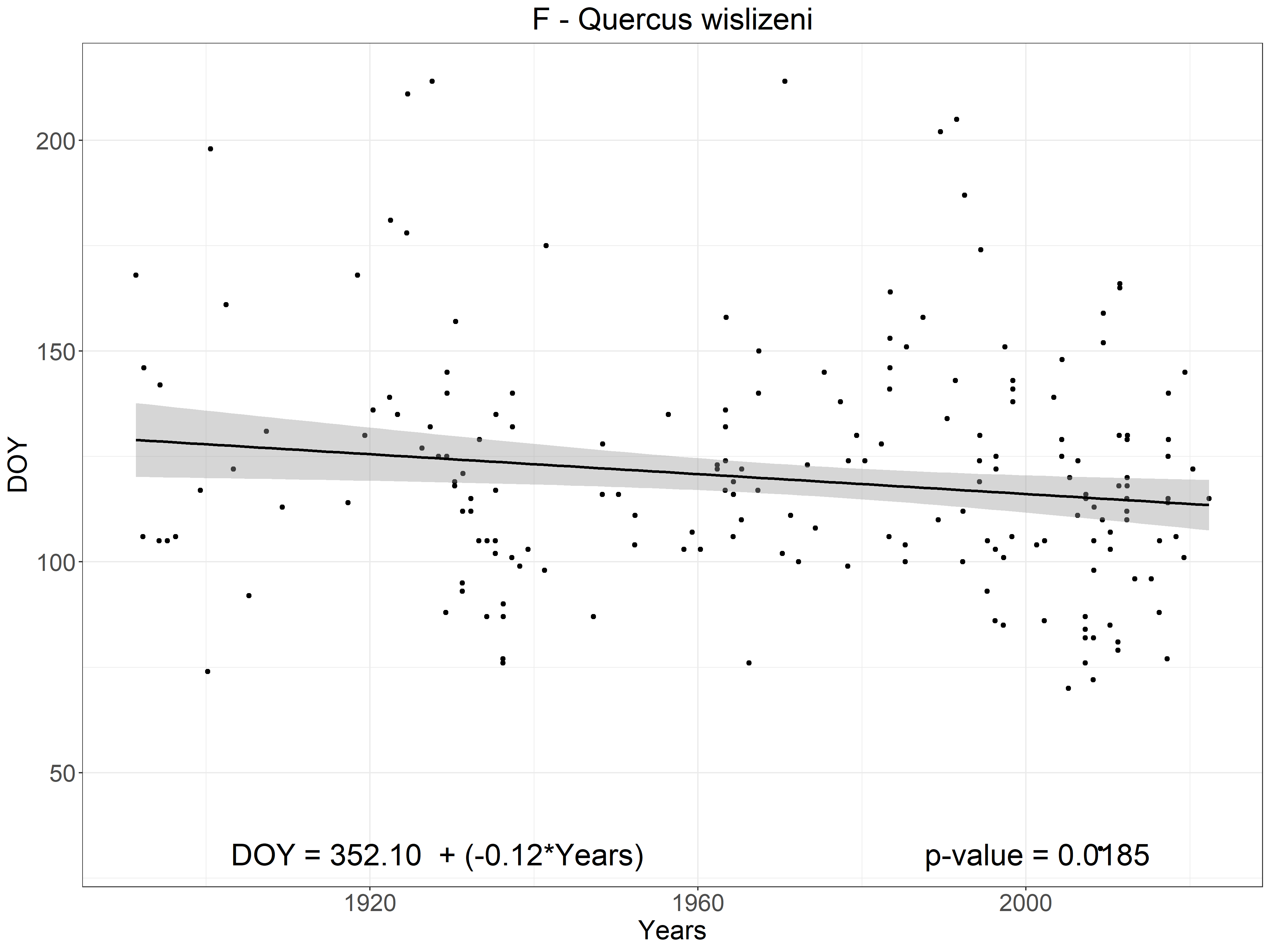

Supplement: Supplementary file 1 [file plants-14-00843-s001.zip › File S2-Species/S2.1-DOYvsYears/1_LM/Plots/F_Quercus wislizeni_plot.png]

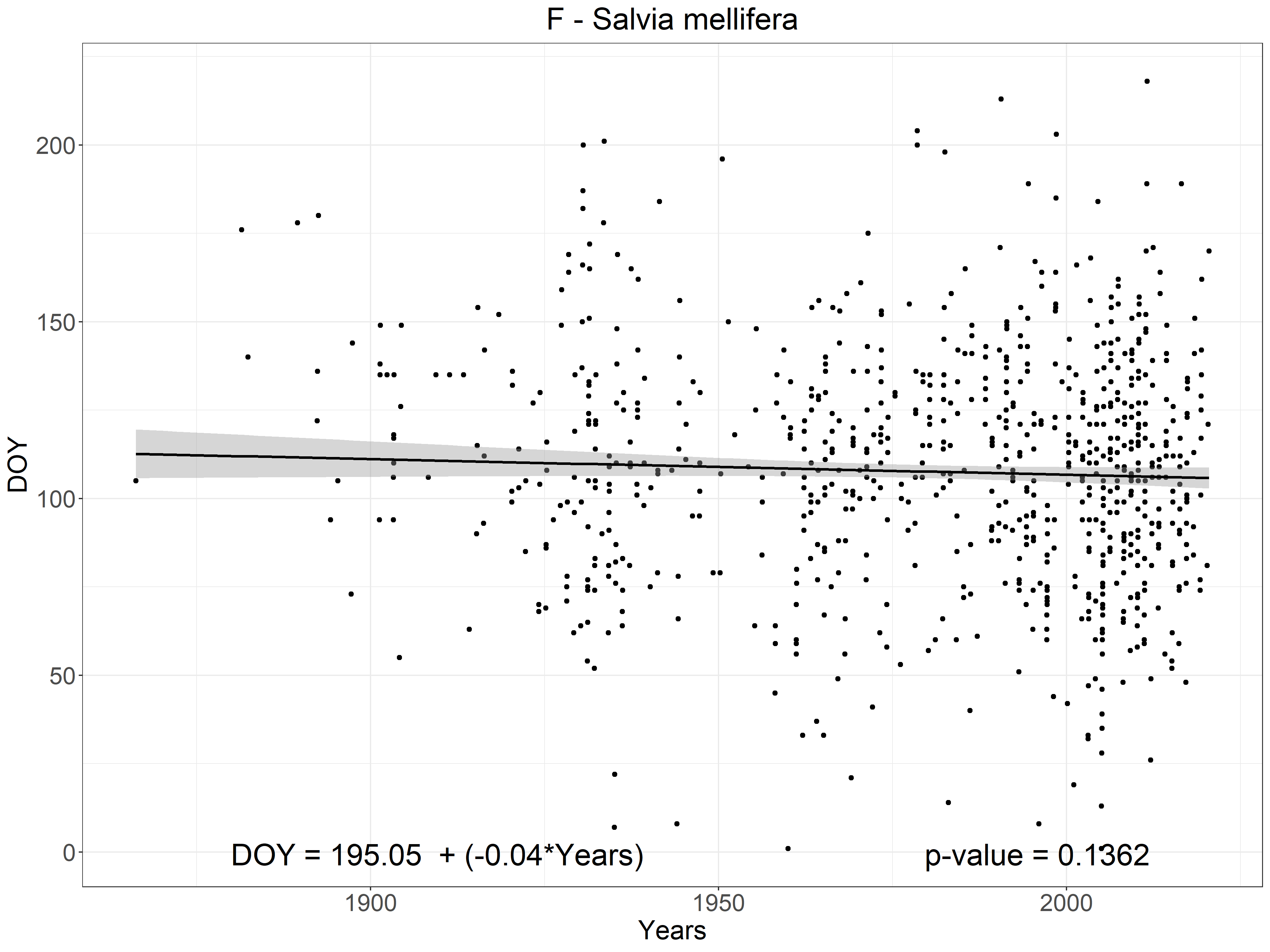

Supplement: Supplementary file 1 [file plants-14-00843-s001.zip › File S2-Species/S2.1-DOYvsYears/1_LM/Plots/F_Salvia mellifera_plot.png]

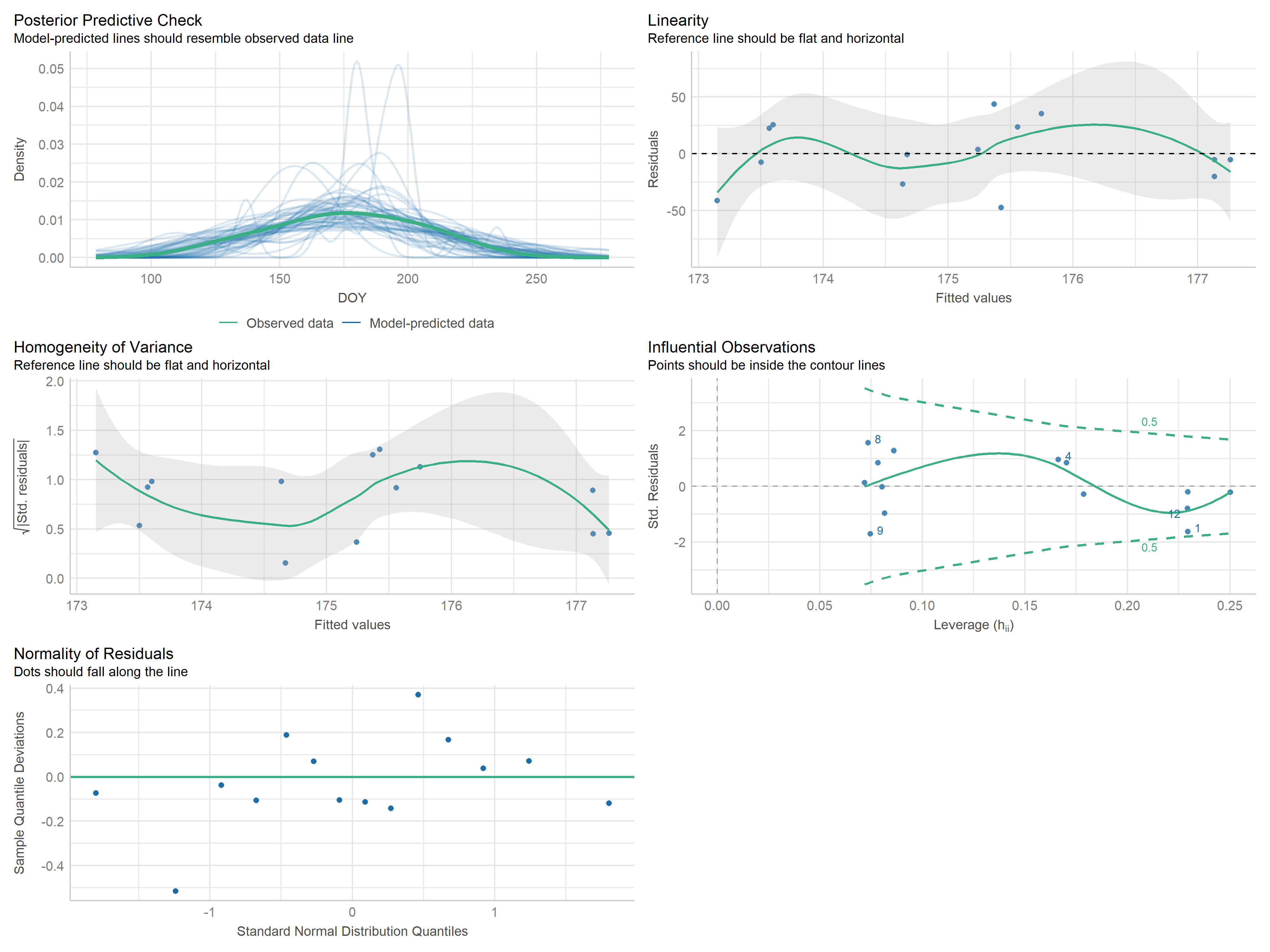

Supplement: Supplementary file 1 [file plants-14-00843-s001.zip › File S2-Species/S2.1-DOYvsYears/1_LM/Plots/Residuals_DVG_Abies concolor.png]

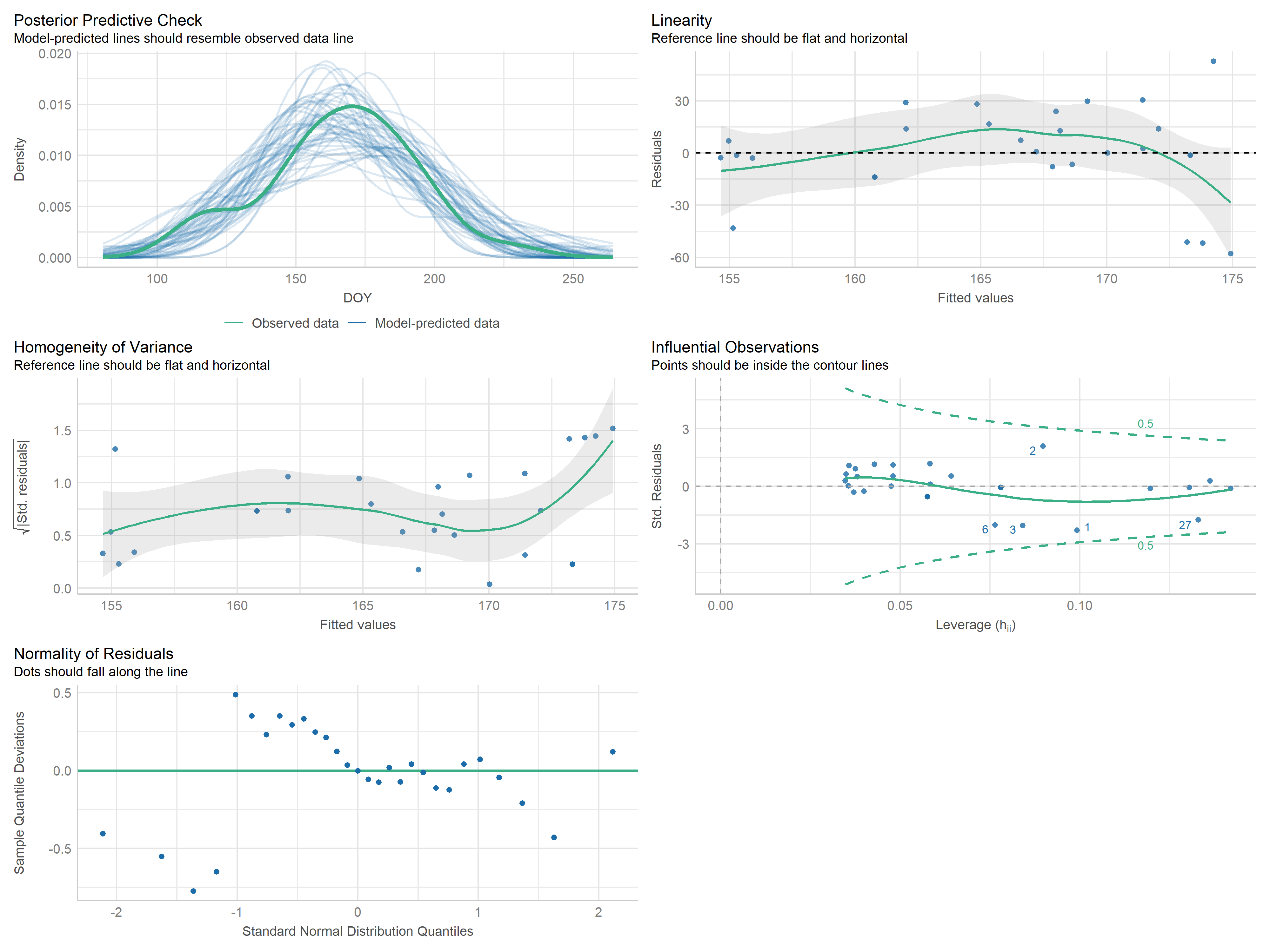

Supplement: Supplementary file 1 [file plants-14-00843-s001.zip › File S2-Species/S2.1-DOYvsYears/1_LM/Plots/Residuals_DVG_Acer glabrum.png]

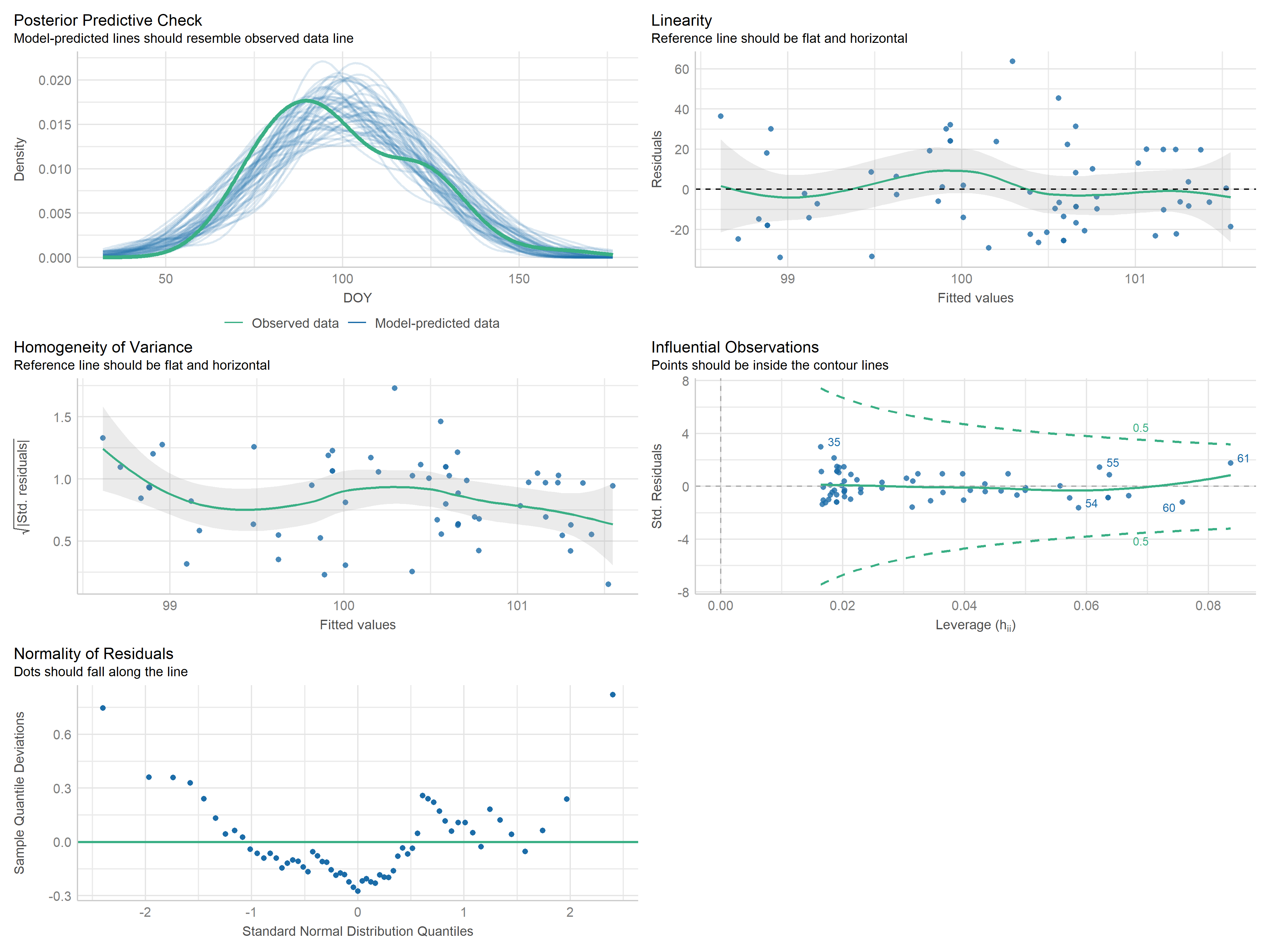

Supplement: Supplementary file 1 [file plants-14-00843-s001.zip › File S2-Species/S2.1-DOYvsYears/1_LM/Plots/Residuals_DVG_Acer macrophyllum.png]

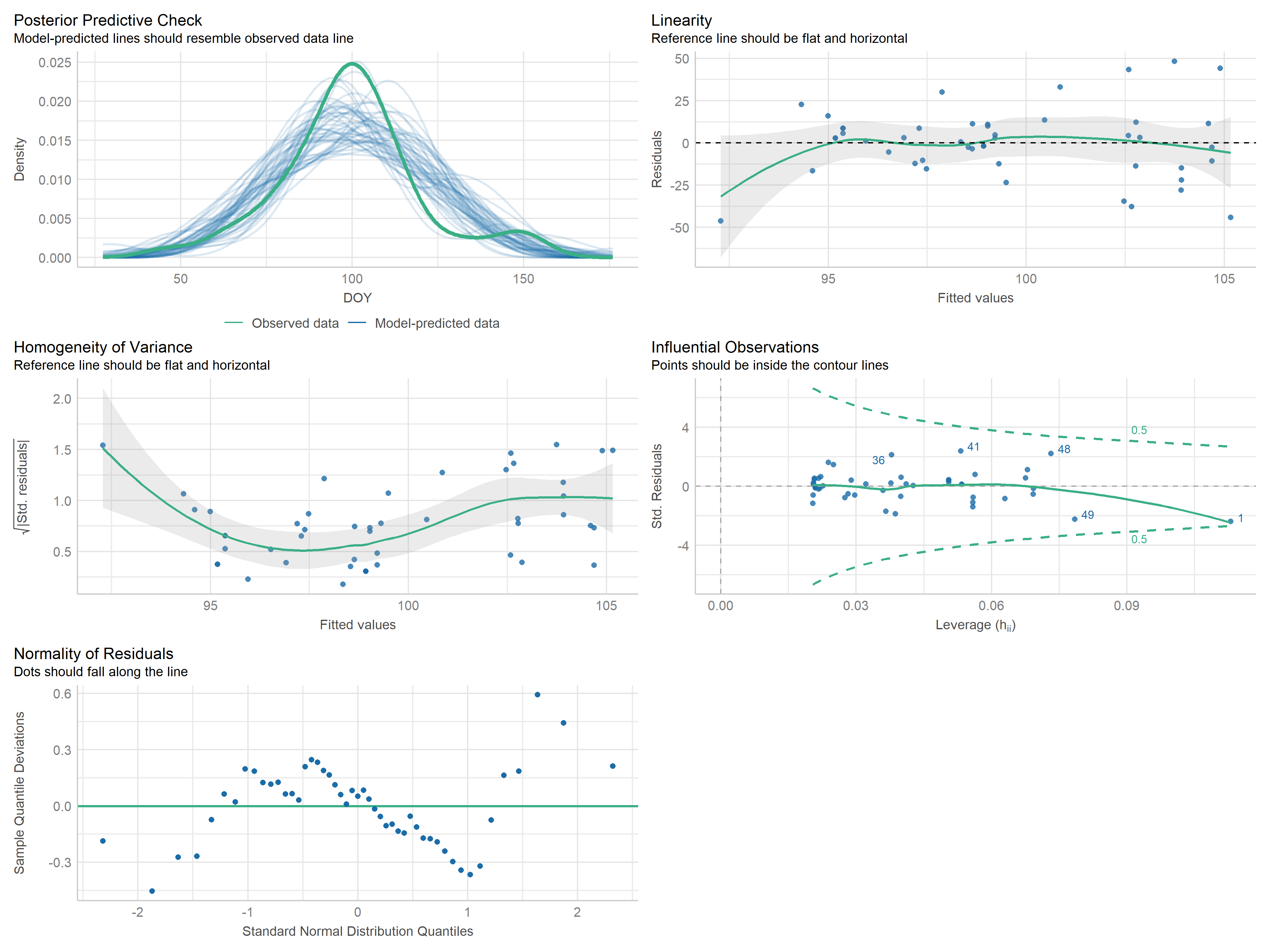

Supplement: Supplementary file 1 [file plants-14-00843-s001.zip › File S2-Species/S2.1-DOYvsYears/1_LM/Plots/Residuals_DVG_Amsinckia menziesii.png]

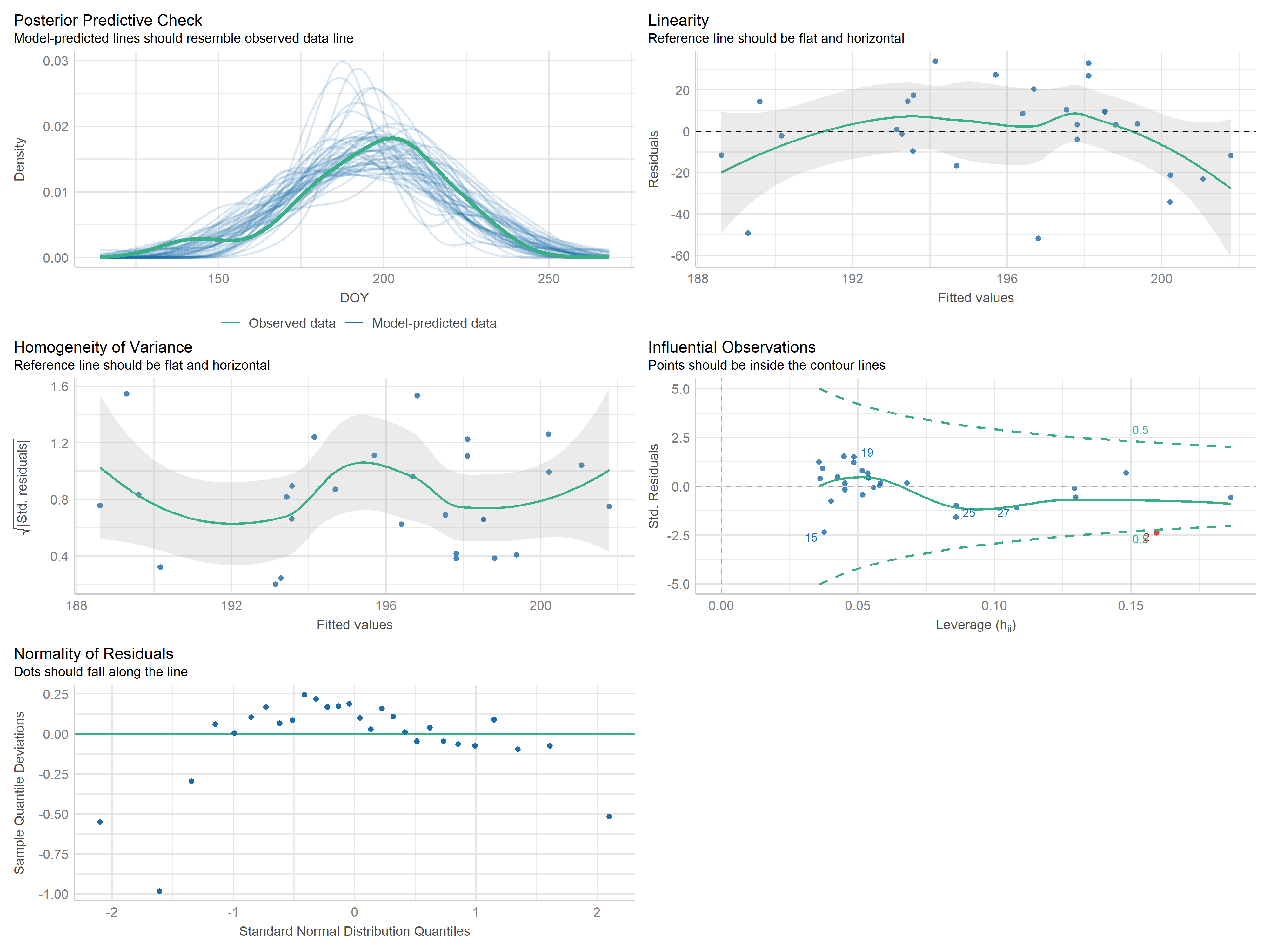

Supplement: Supplementary file 1 [file plants-14-00843-s001.zip › File S2-Species/S2.1-DOYvsYears/1_LM/Plots/Residuals_DVG_Arctostaphylos nevadensis.png]

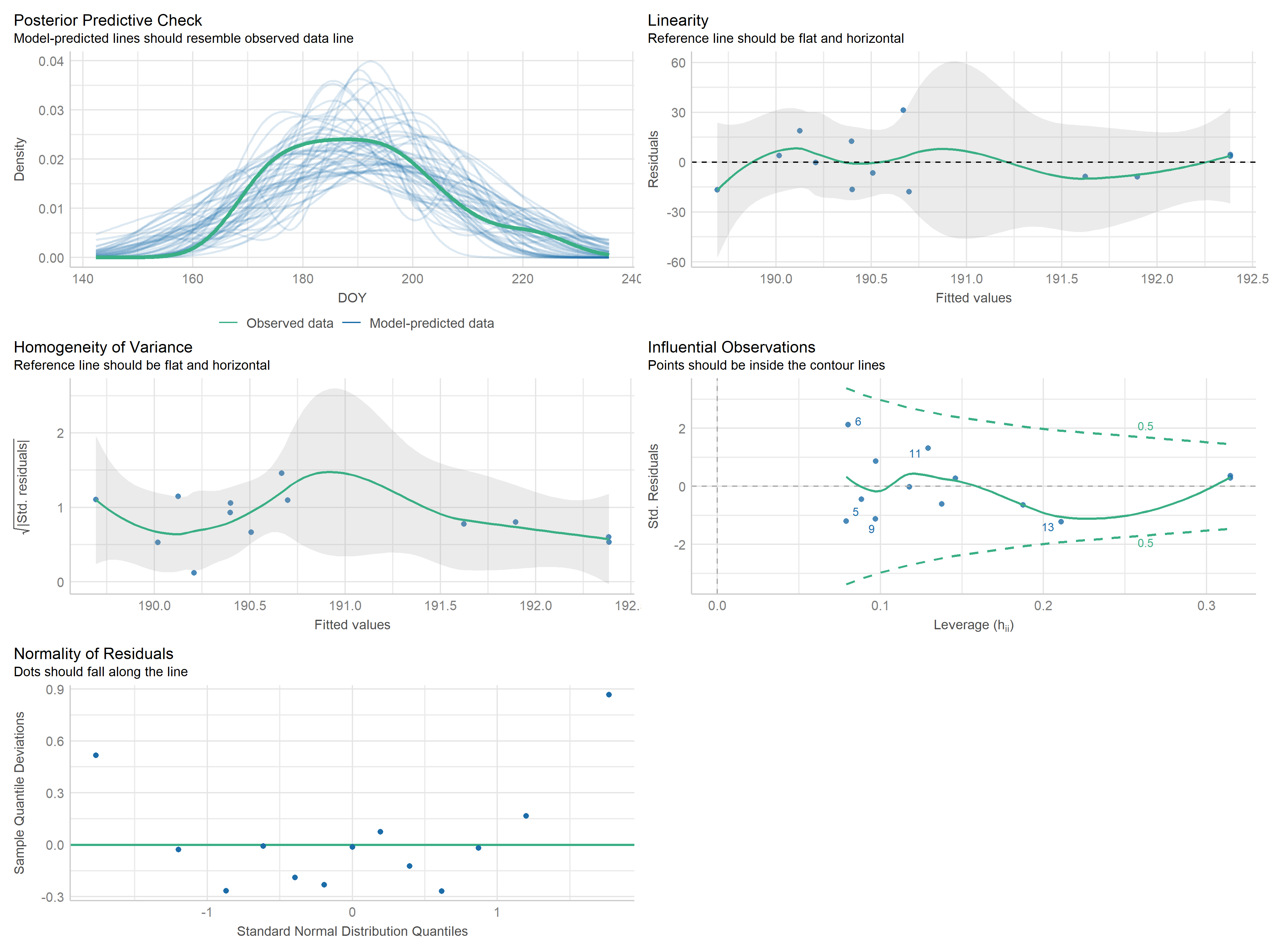

Supplement: Supplementary file 1 [file plants-14-00843-s001.zip › File S2-Species/S2.1-DOYvsYears/1_LM/Plots/Residuals_DVG_Arctostaphylos patula.png]

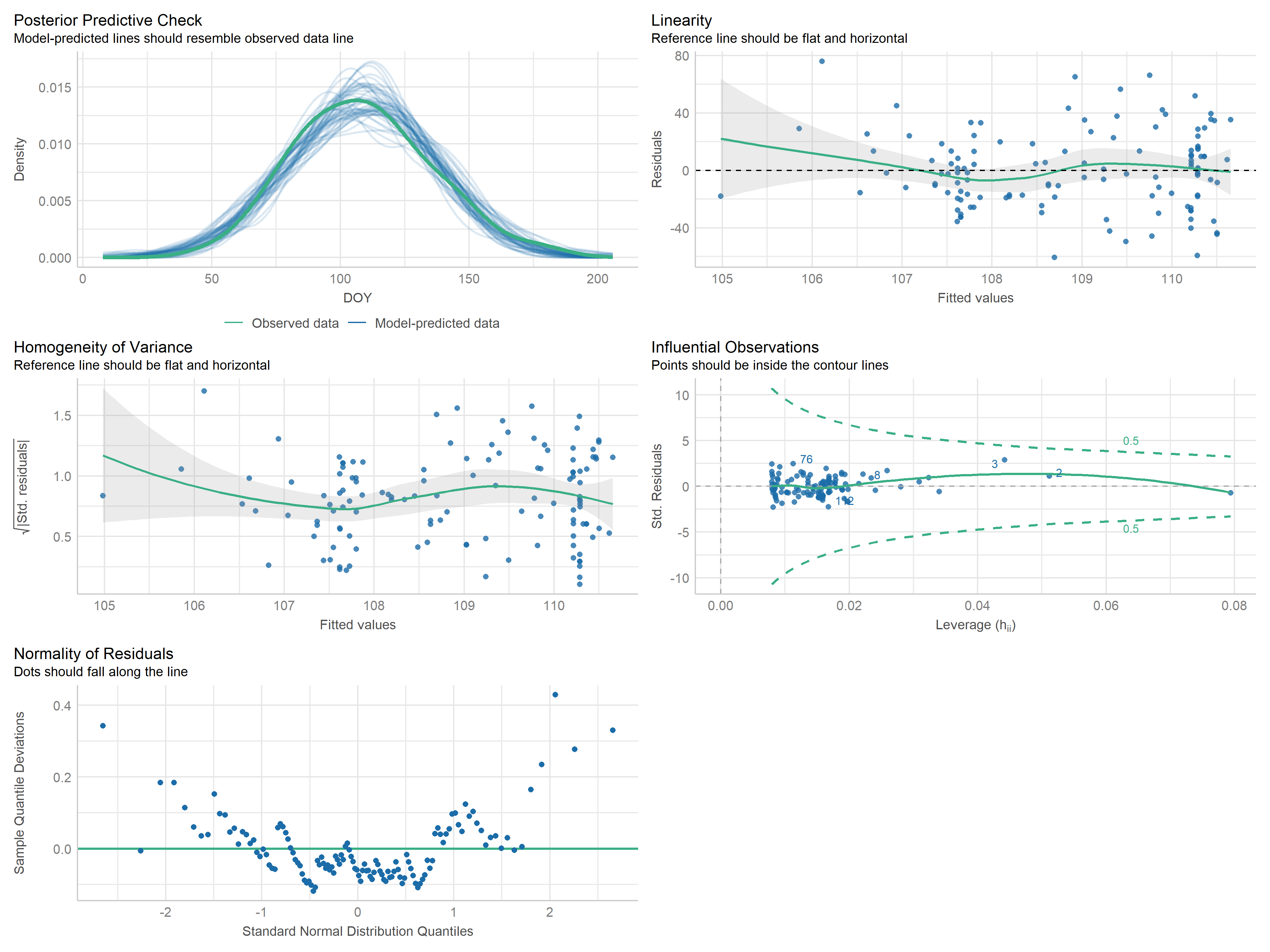

Supplement: Supplementary file 1 [file plants-14-00843-s001.zip › File S2-Species/S2.1-DOYvsYears/1_LM/Plots/Residuals_DVG_Ceanothus cuneatus.png]

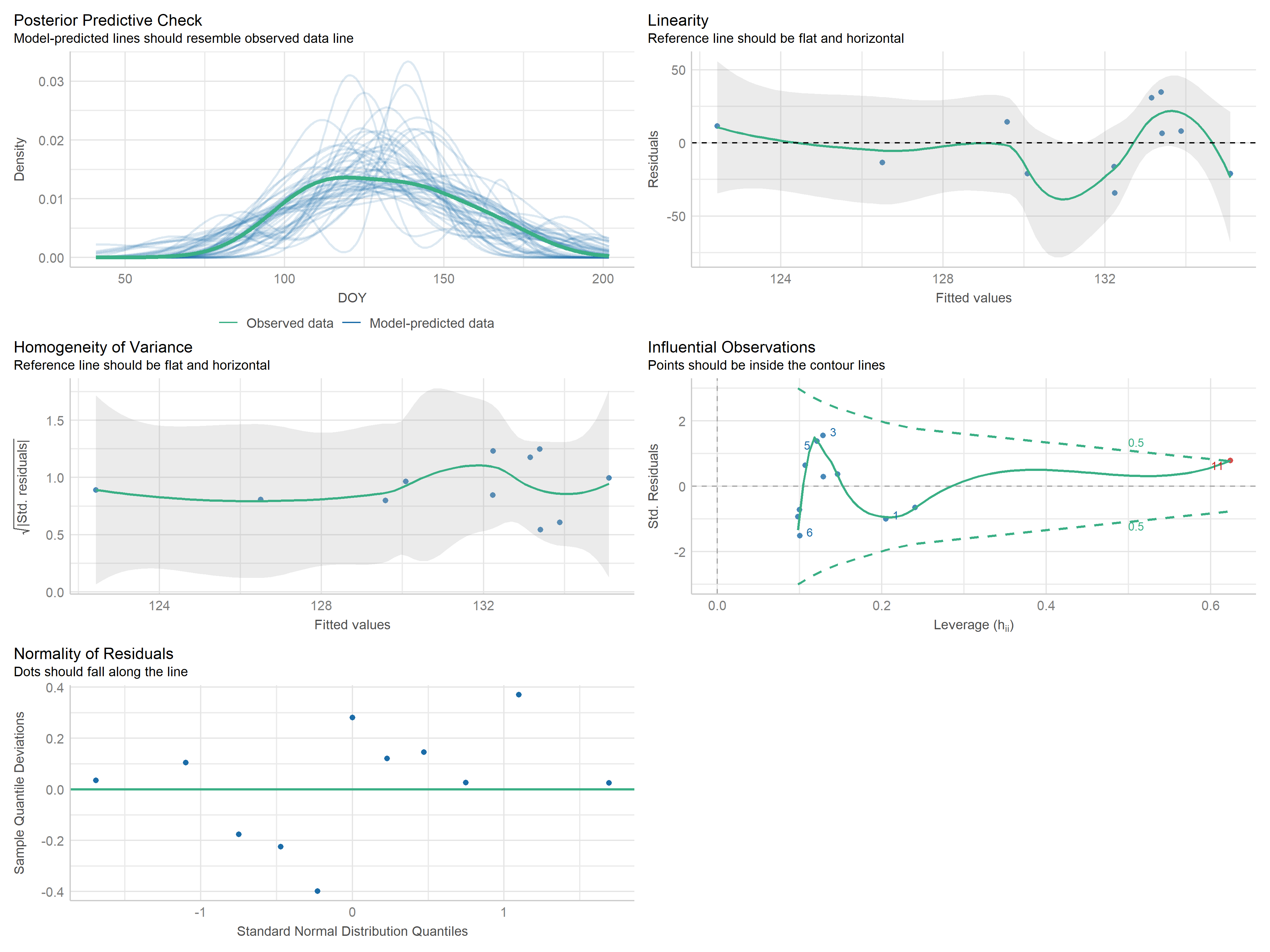

Supplement: Supplementary file 1 [file plants-14-00843-s001.zip › File S2-Species/S2.1-DOYvsYears/1_LM/Plots/Residuals_DVG_Dudleya cymosa.png]

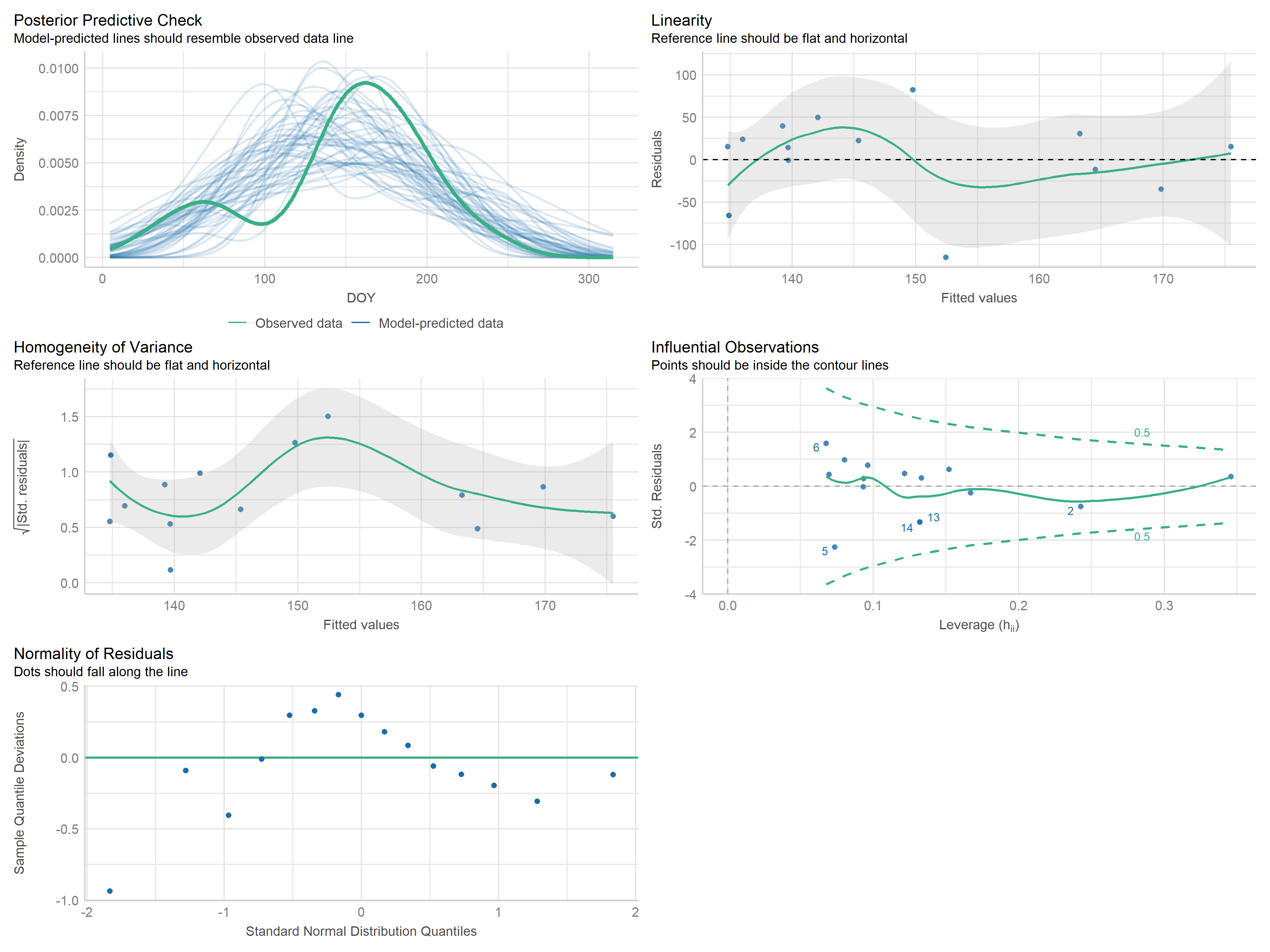

Supplement: Supplementary file 1 [file plants-14-00843-s001.zip › File S2-Species/S2.1-DOYvsYears/1_LM/Plots/Residuals_DVG_Heteromeles arbutifolia.png]

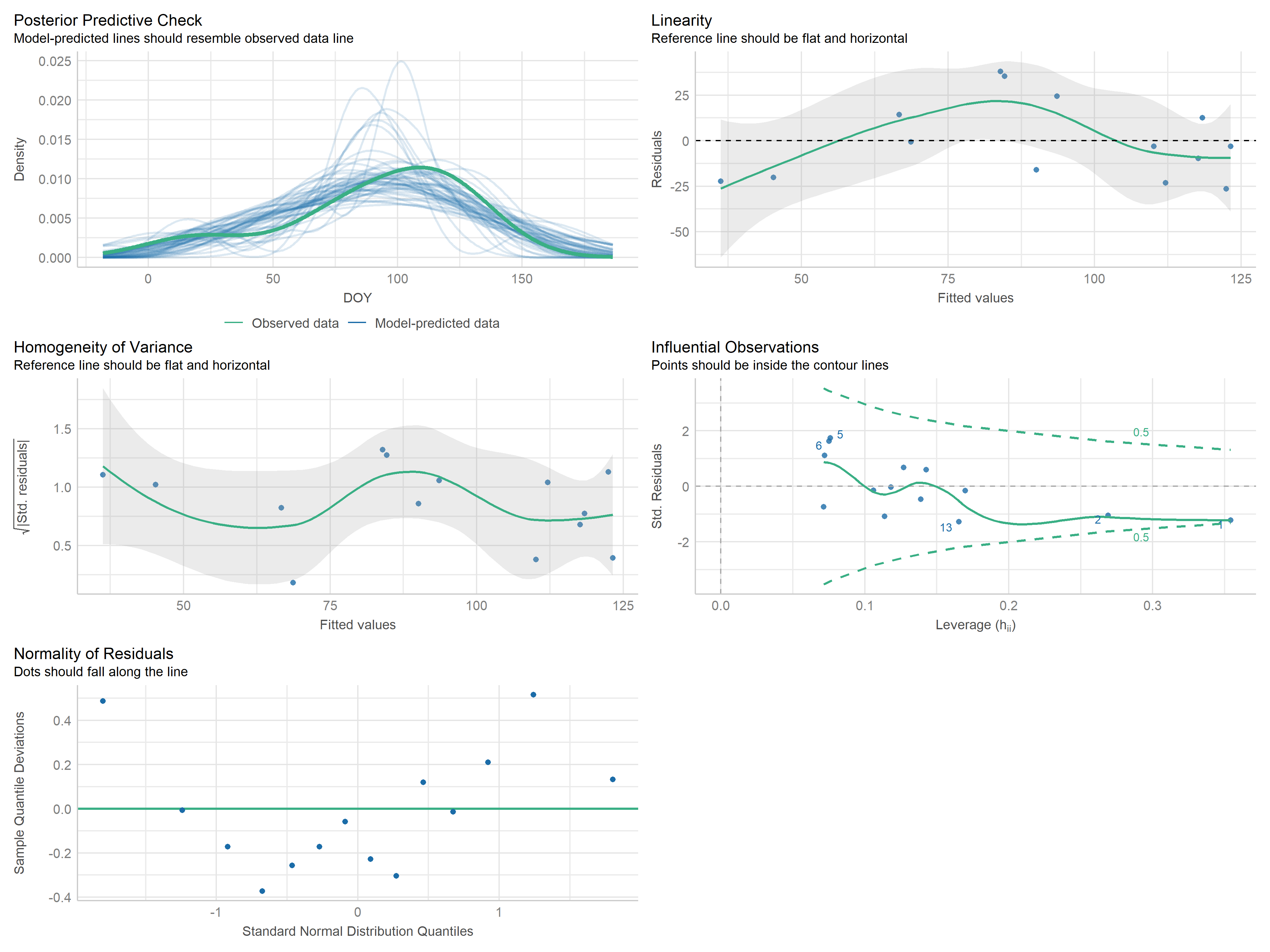

Supplement: Supplementary file 1 [file plants-14-00843-s001.zip › File S2-Species/S2.1-DOYvsYears/1_LM/Plots/Residuals_DVG_Nassella pulchra.png]

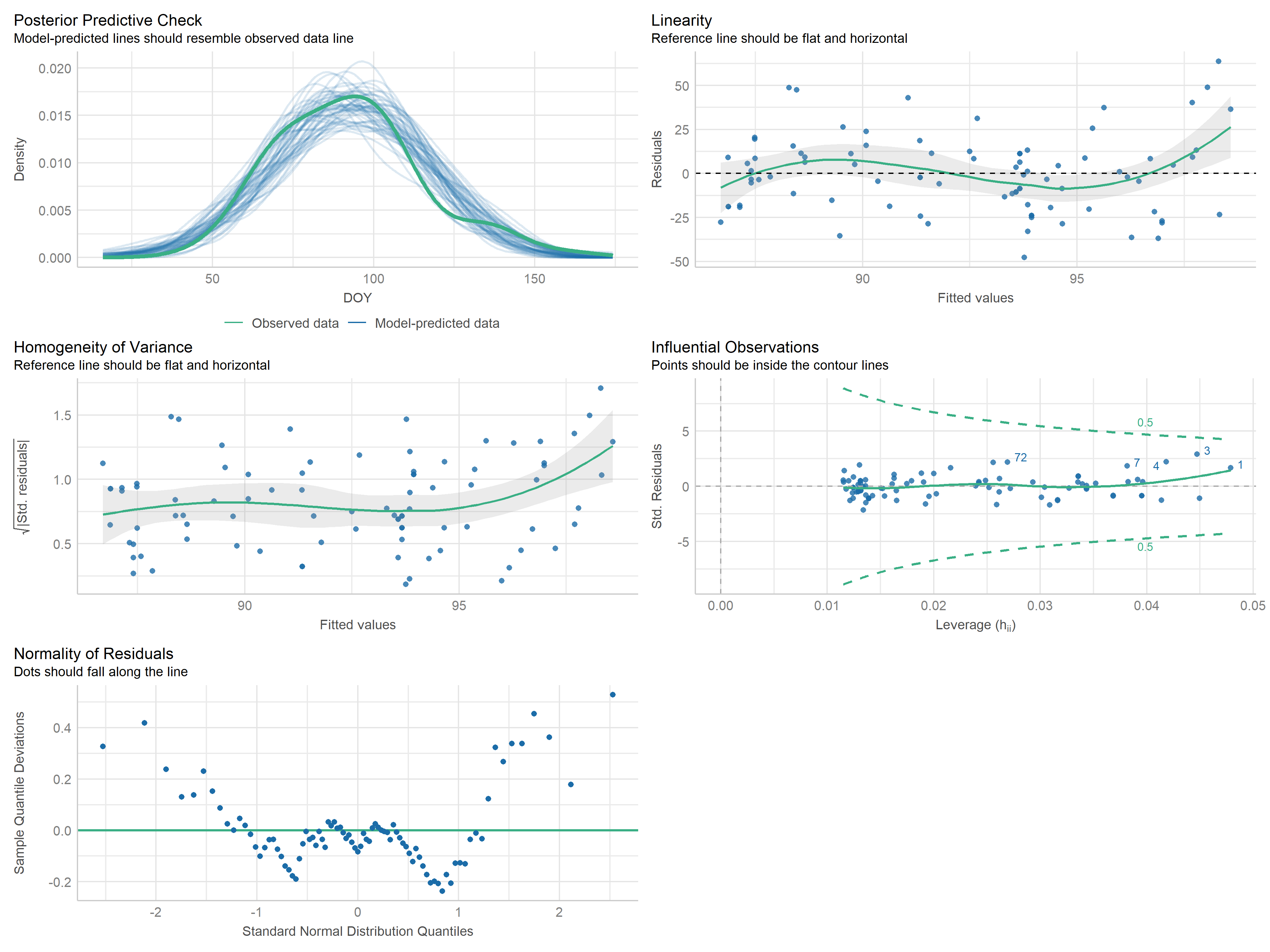

Supplement: Supplementary file 1 [file plants-14-00843-s001.zip › File S2-Species/S2.1-DOYvsYears/1_LM/Plots/Residuals_DVG_Plagiobothrys nothofulvus.png]

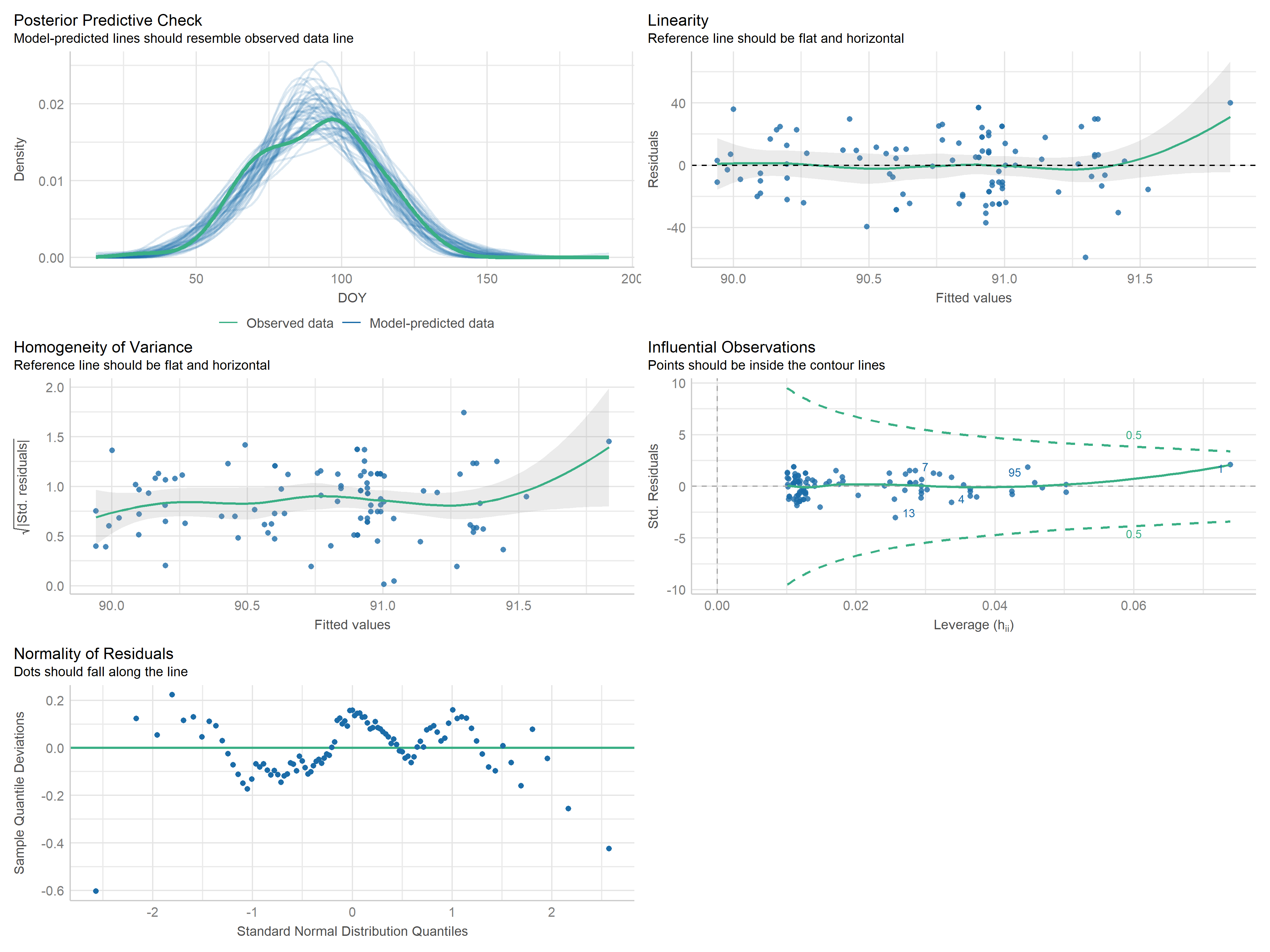

Supplement: Supplementary file 1 [file plants-14-00843-s001.zip › File S2-Species/S2.1-DOYvsYears/1_LM/Plots/Residuals_DVG_Quercus agrifolia.png]

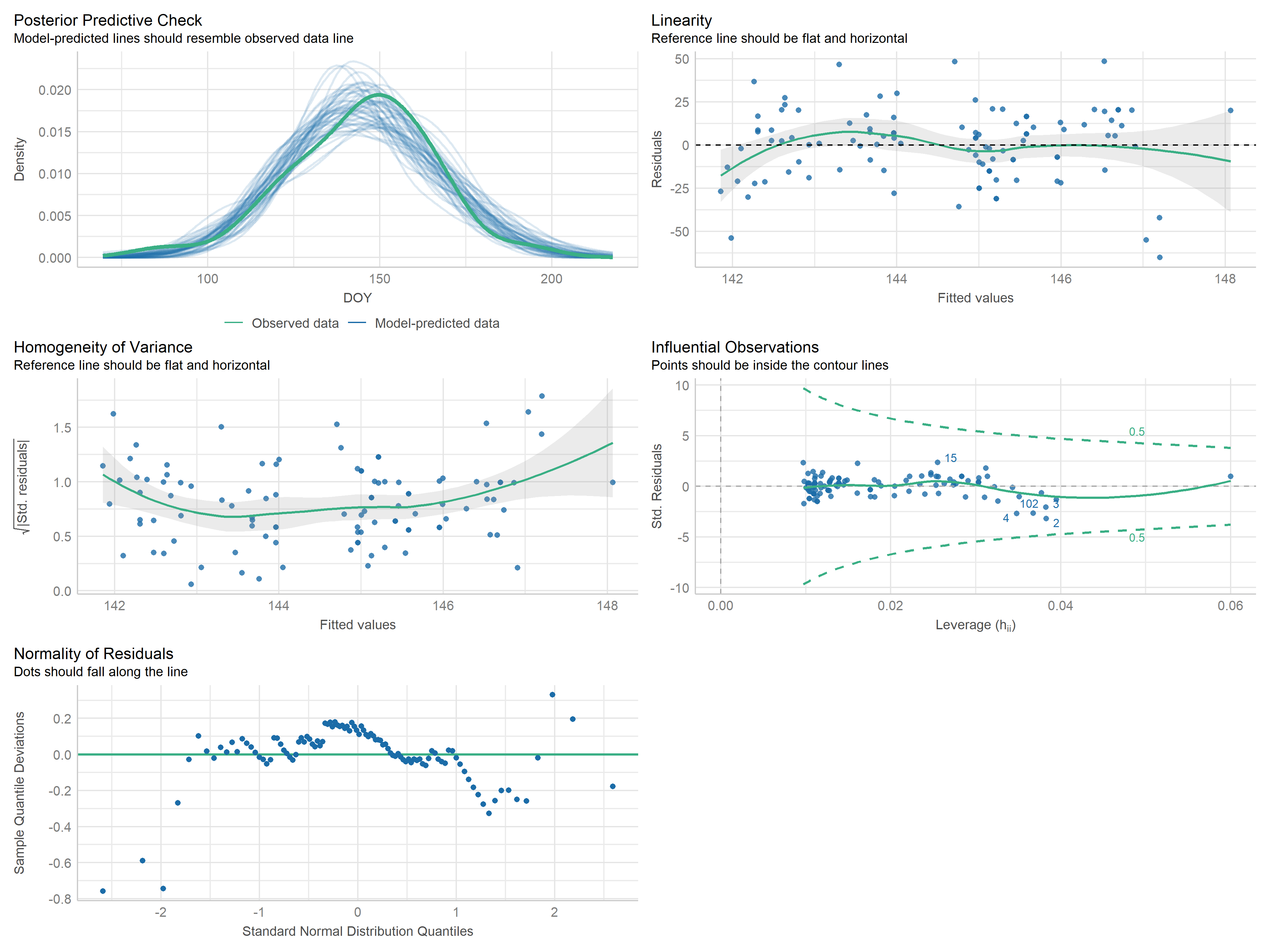

Supplement: Supplementary file 1 [file plants-14-00843-s001.zip › File S2-Species/S2.1-DOYvsYears/1_LM/Plots/Residuals_DVG_Quercus chrysolepis.png]

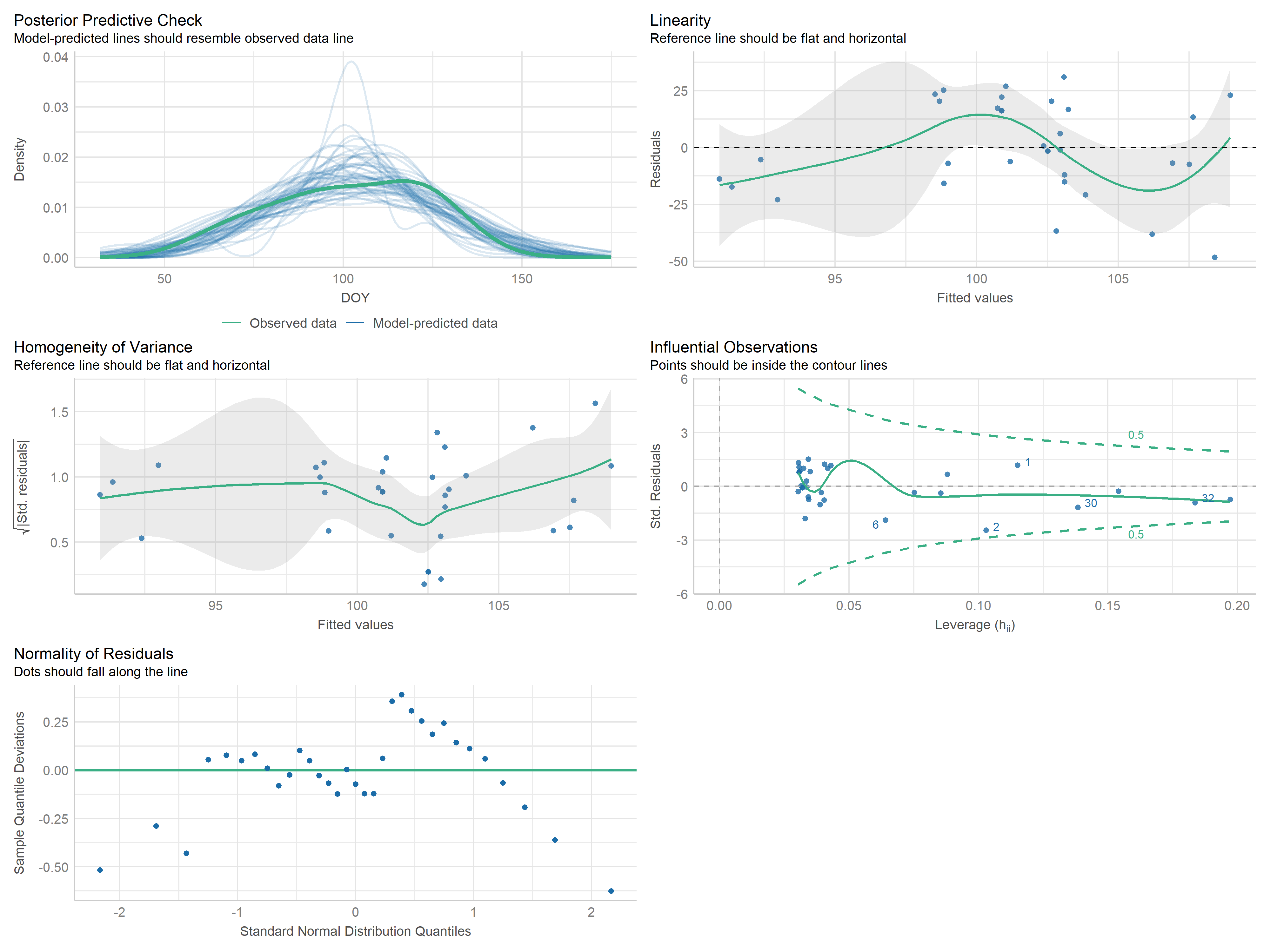

Supplement: Supplementary file 1 [file plants-14-00843-s001.zip › File S2-Species/S2.1-DOYvsYears/1_LM/Plots/Residuals_DVG_Quercus douglasii.png]

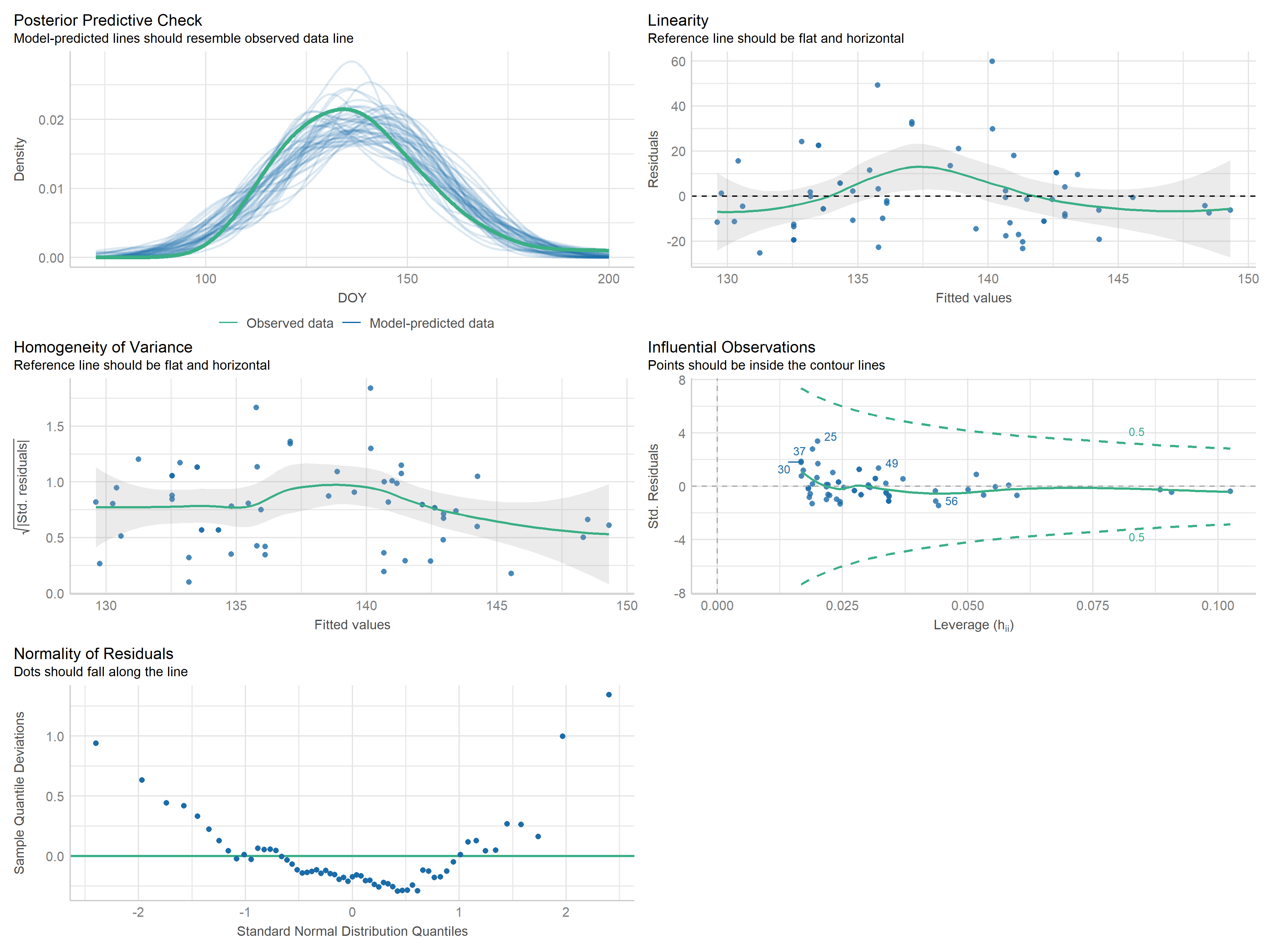

Supplement: Supplementary file 1 [file plants-14-00843-s001.zip › File S2-Species/S2.1-DOYvsYears/1_LM/Plots/Residuals_DVG_Quercus durata.png]

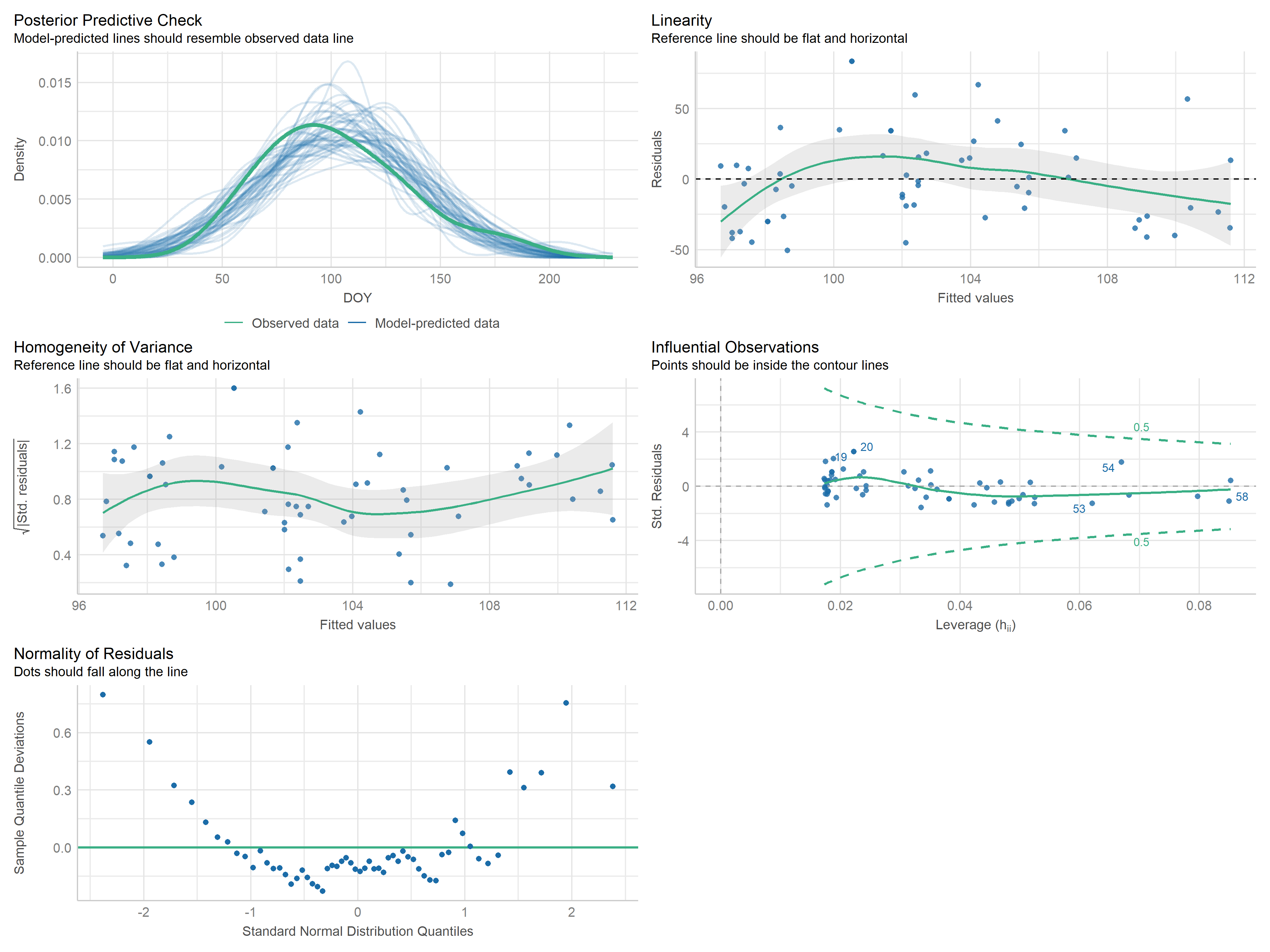

Supplement: Supplementary file 1 [file plants-14-00843-s001.zip › File S2-Species/S2.1-DOYvsYears/1_LM/Plots/Residuals_DVG_Quercus kelloggii.png]

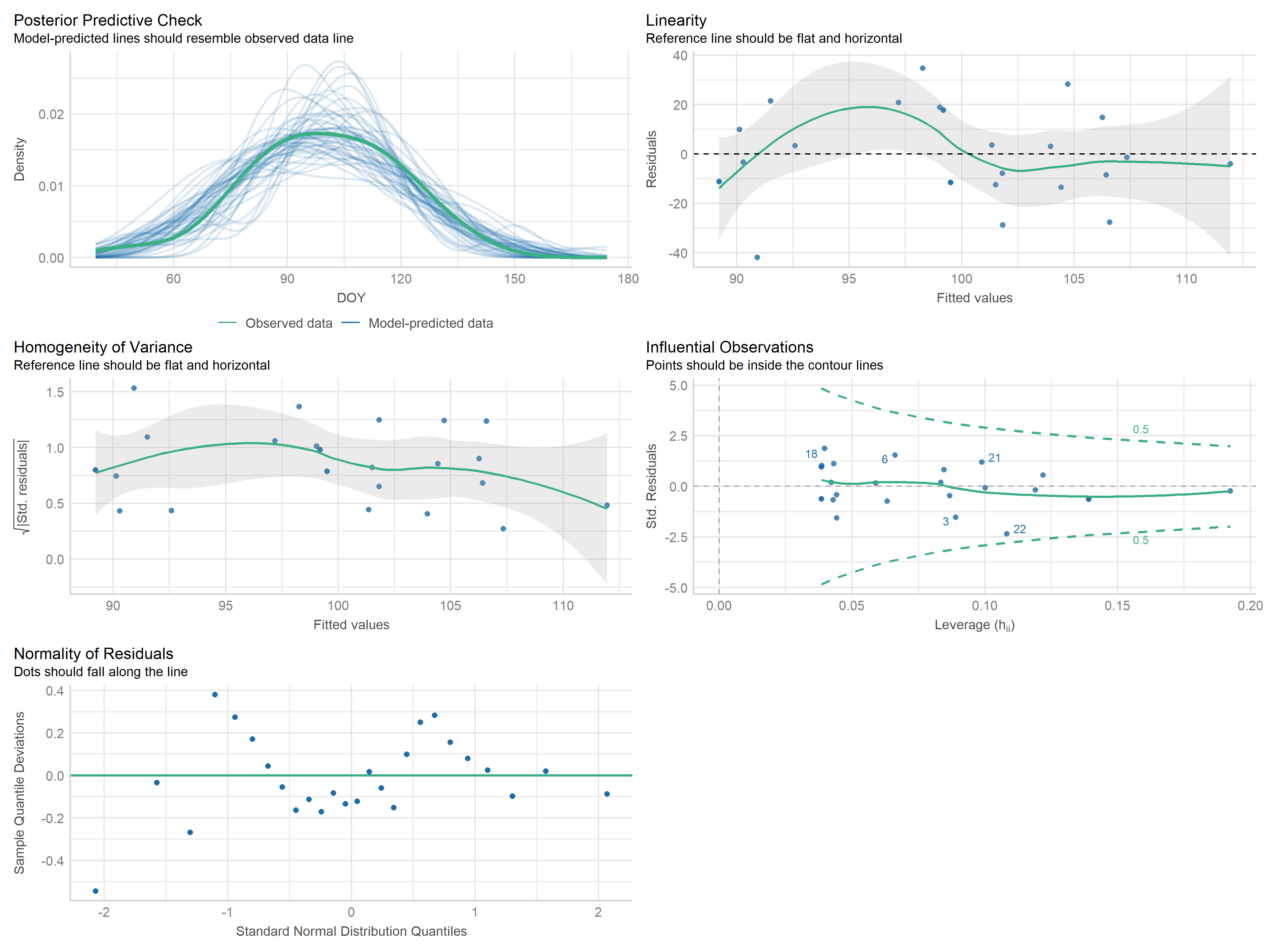

Supplement: Supplementary file 1 [file plants-14-00843-s001.zip › File S2-Species/S2.1-DOYvsYears/1_LM/Plots/Residuals_DVG_Quercus lobata.png]

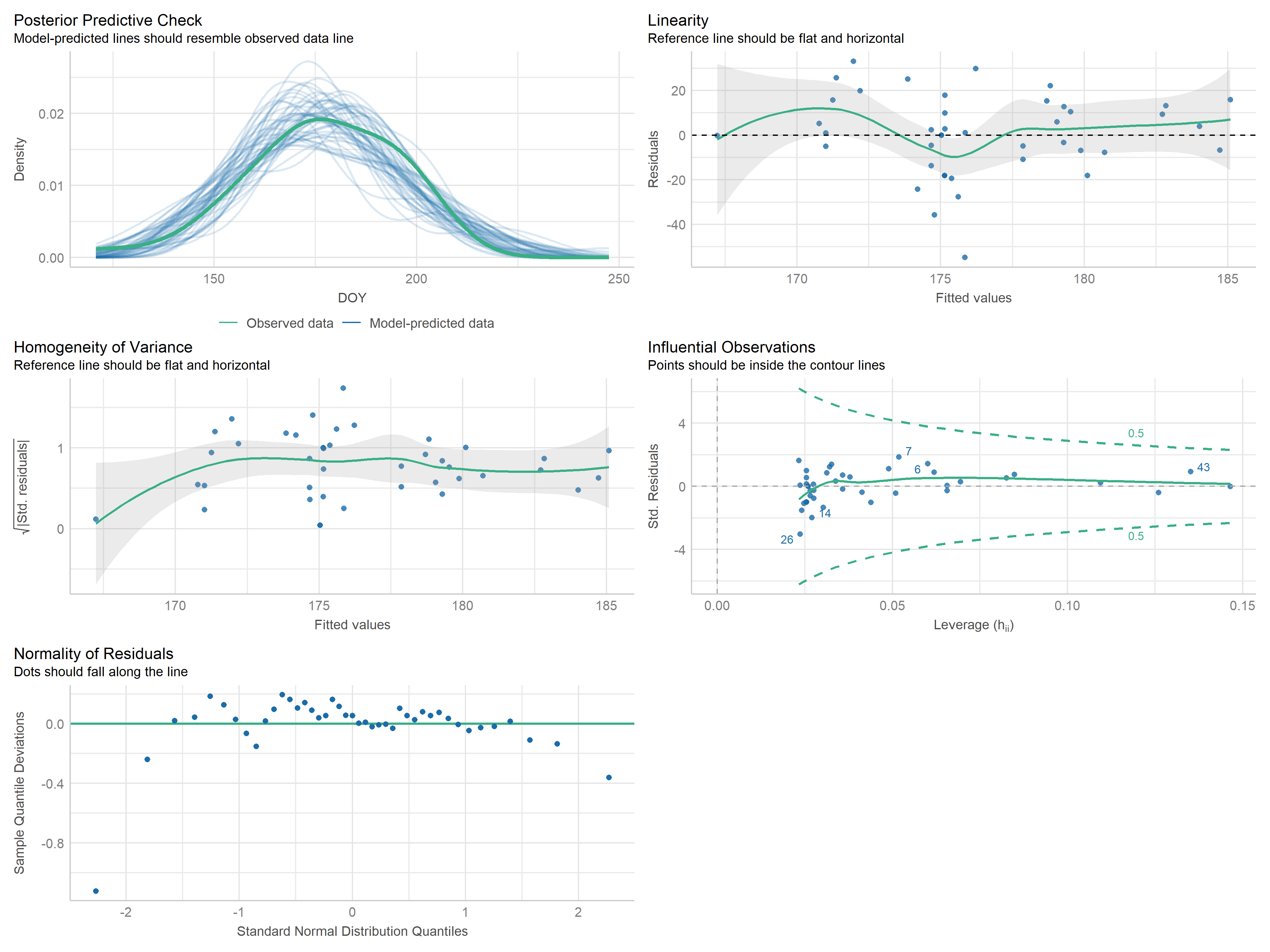

Supplement: Supplementary file 1 [file plants-14-00843-s001.zip › File S2-Species/S2.1-DOYvsYears/1_LM/Plots/Residuals_DVG_Quercus vaccinifolia.png]

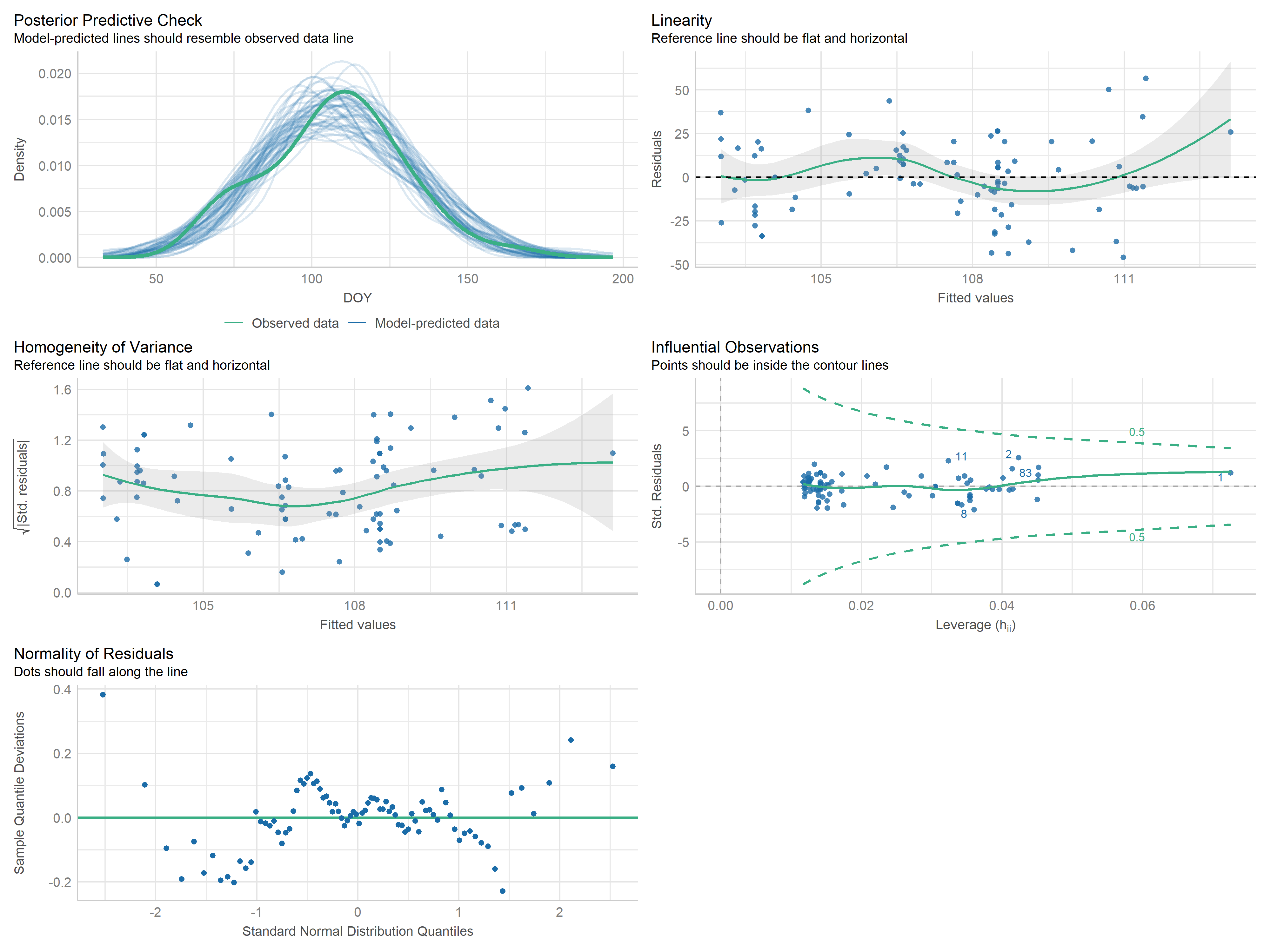

Supplement: Supplementary file 1 [file plants-14-00843-s001.zip › File S2-Species/S2.1-DOYvsYears/1_LM/Plots/Residuals_DVG_Quercus wislizeni.png]
