# Supplementary material for: Phenological Shifts Since 1830 in 29 Native Plant Species of California and Their Responses to Historical Climate Change
Source: Plants (Basel). 2025 Mar 7;14(6):843. doi: 10.3390/plants14060843 (PMC11945038; doi:10.3390/plants14060843)
Supplement: Supplementary file 1 [file plants-14-00843-s001.zip › File S3-Ecoregions/File S3-README.pdf]

Phenological shifts since 1830 in 29 native plant species of California and their responses to historical climate change

Andros Solakis-Tena [1]*, Noelia Hidalgo-Triana [1], Ryan Boynton [2] and James H. Thorne [2]

[1] Department of Botany and Plant Physiology (Botany Area), Faculty of Science, University of Málaga, Málaga 29010, Spain; andros@uma.es (A.S.T); nhidalgo@uma.es (N.H.T.)
[2] Department of Environmental Science and Policy, University of California, Davis 95616, USA; rmboynton@ucdavis.edu (R.B.); jhthorne@ucdavis.edu (J.H.T.)
* Correspondence: andros@uma.es

File S3

Models by Jepson ecoregion

File S3 contains the results of the models by Jepson ecoregion. Within the File S3 folder, there are three subfolders:

1. **S3.1-DOYvsYears**, which contains the results of the models comparing the day of year (DOY) with the time variable (Years). This folder includes the results of the linear models (1_LM), generalized linear models (2_Gaussian_Log), and generalized additive models (3_GAM), as well as an extra folder (4_Results_synthesis) with the synthesized results of DOY versus time for the final models. Each of the subfolders for the model types contains the results for each taxon and phenophase in separate and independent CSV files, showing the different models tested for selection based on the AIC criterion. In these subfolders, there are two CSV files: one that collects the selected results according to the type of model used (Results.csv), and another with the results of those models that did not meet the criteria for normality, linearity, or homoscedasticity (Results_NONORM.csv). These have been evaluated in the next folder, either by applying various GLM or GAM. Additionally, within each model subfolder, there is another subfolder called Plots, which contains graphs of the results (both significant and non-significant) of the models and their residual plots to validate the model. Each graph is a .png image named with the taxon and its analyzed phenophase. The structure is as follows: F_ecoregion_plot.png for the modeling graph, and Residuals_F_ecoregion.png for the residual analysis graphs. E.g., F_Great Valley_plot.png.

2. **S3.2-DOYvsClimate** contains the results of the models comparing the day of year (DOY) with the climatic variables. This folder includes the results in the same structure as in DOYvsYears, as well as the folder with the synthesized results of DOY versus the climatic variables for the final models.

3. **S3.3-SpecimensxEcoregion** contains the figures (Figure S3.3.1-S3.3.5) distribution across the state and by Jepson ecoregion of the used samples/preserved specimens of each species.
